# The Challenging Complete and Detailed ^1^H and ^13^C NMR Assignment for *ent*-Kaurenoic Acid, a Remarkable Natural Product

**DOI:** 10.1021/acsomega.5c09155

**Published:** 2025-11-20

**Authors:** Alexsandro Eurípedes Ferreira, Ana Carolina Ferreira Soares Rocha, Julian Carlos da Silva Pavan, Viviani Nardini Takahashi, Herbert Júnior Dias, Patrícia Mendonça Pauletti, Daiane Cristina Sass, Vladimir Constantino Gomes Heleno

**Affiliations:** † Núcleo de Pesquisas em Ciências Exatas e Tecnológicas, 92917 Universidade de Franca, Av. Dr. Armando de Salles Oliveira, 201 − Pq. Universitário, 14404-600, Franca, SP, Brazil; ‡ Departamento de Química, Faculdade de Filosofia, Ciências e Letras de Ribeirão Preto, USP − Av. Bandeirantes,3900 − Vila de Monte Alegre, 14040-900, Ribeirão Preto, SP, Brazil; § 119524 Instituto Federal Goiano, Campus Urutaí, Núcleo de Química, Rod. Geraldo Silva Nascimento, Km-2,5 - Zona Rural, 75790-000, Urutaí, GO, Brazil; ∥ Instituto de Biociências de Rio Claro, UNESP − Jardim Bela Vista, 13506-900, Rio Claro, SP, Brazil

## Abstract

In this work, we report a comprehensive structural assignment of the ^1^H and ^13^C nuclear magnetic resonance (NMR) data for kaurenoic acid (KA, *ent*-kaur-16-en-19-oic acid), a natural diterpene with diverse biological activities. To support this analysis, the methyl ester derivative (*ent*-methyl-kaur-16-en-19-oate) was also investigated, allowing comparison of chemical shift variations arising from subtle structural differences. The complete assignment was achieved through the analysis of multidimensional NMR spectra and accurate determination of ^1^H–^1^H coupling constants and signal multiplicities. Experiments including ^1^H NMR, ^13^C NMR {^1^H}, g-COSY, g-HSQC, g-HMBC, and J-resolved were employed, complemented by spectral simulations using FOMSC3 and SimEsp_NMR software. Measurements in different deuterated solvents further clarified overlapping regions and enhanced data reliability. This approach resulted in the most detailed and complete NMR data set to date for KA and its methyl ester. To our knowledge, no previous studies on KA have provided this level of spectroscopic detail or a step-by-step description of the data acquisition, underscoring the novelty and relevance of this work.

## Graphical abstract

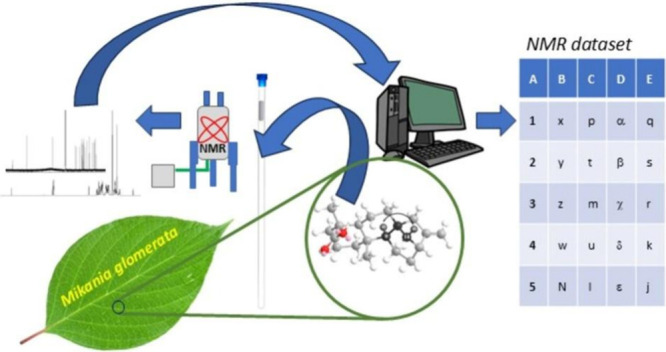

## Introduction

Diterpenes are specialized metabolites predominantly found in plants and marine organisms, although their occurrence has also been reported in other biological sources.^1,2^ This class of natural products (NP) exhibits remarkable structural diversity, encompassing skeletons such as kauranes, pimaranes, and abietanes.^3^ Beyond the diversity of core frameworks, the wide range of possible substituents generates an extensive variety of compounds.^4,5^ Many of these molecules display significant biological and ecological activities,^4,6^ including antiparasitic,^7,8^ phytotoxic,^9^ anti-inflammatory,^10^ antibacterial,^4,11^ fungicidal,^4,12^ and antiviral^4,13^ effects, among others.^4,5,14^

Several plant families, including Asteraceae,^15^ Lamiaceae,^16^ Araucariaceae,^17^ Flacourtiaceae,^18^ and Celastraceae,^19^ particularly Asteraceae,^20^ are known sources of diverse diterpenes. These compounds have attracted considerable attention as prototypes for the development of derivatives, which are often investigated for their biological properties.^4,7,10,13,15^

Among them, *ent*-kaur-16­(17)-en-19-oic acid (*ent*-kaurenoic acid, KA) (1) (Figure 1) stands out as a kaurane diterpene with a rigid tetracyclic skeleton. With more than eight hundred references retrieved in a SciFinder search, KA ranks among the most widely studied diterpenes and plays a central role in various areas of natural product research.^21–25^

**Figure 1:**
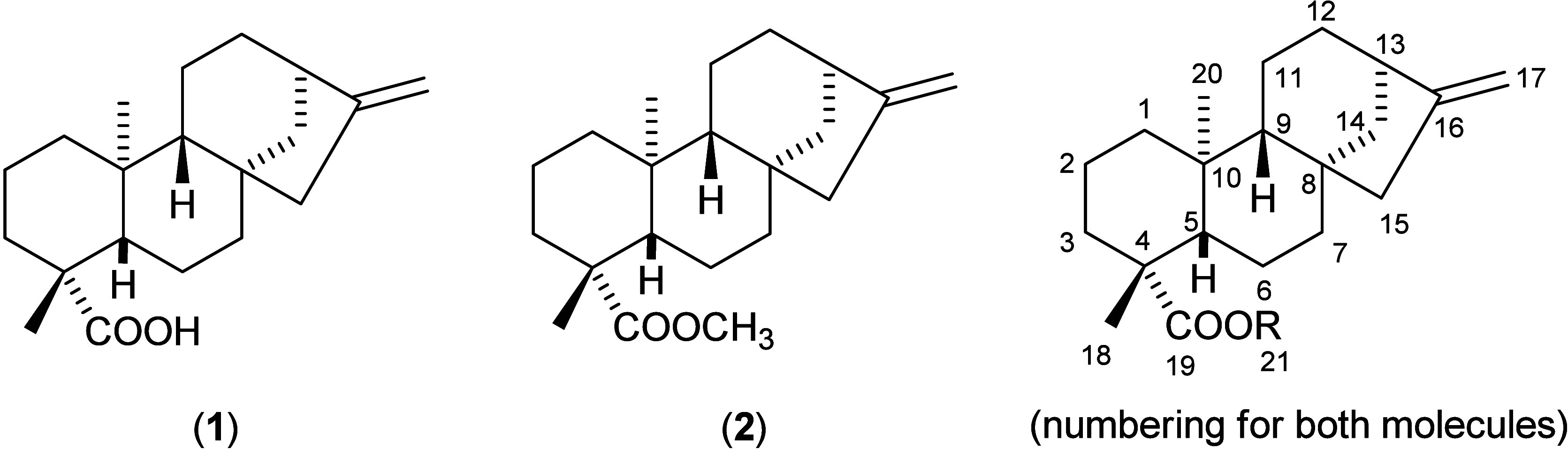
Chemical structures of *ent*-kaur-16­(17)-en-19-oic acid (kaurenoic acid, KA) (**1**) and methyl-*ent*-kaur-16-en-19-oate, its methyl ester, KAMe (**2**).

This compound can be isolated from numerous plant species native to Central and South America, including *Mikania glomerata* Sprengel and *Mikania laevigata* Schultz Bip. (Asteraceae), popularly known in Brazil as “Guaco”.^26^ It has also been isolated from many other plants and occurs as a major constituent in extracts or resins of several species.^25,27^ Extracts containing KA exhibit a broad spectrum of biological activities, such as antifungal,^28^ antiasthmatic,^29^ antimalarial, and antispasmodic^30^ effects. The pure compound itself has demonstrated notable anti-inflammatory,^31^ antibacterial,^32^ and antitumor^33^ activities.

Because of these properties, KA has become a major focus of research aimed at elucidating its pharmacological mechanisms and validating its biological potential.^7,15,24,25,27,31,34^ It is therefore considered an attractive structural prototype for the preparation of derivatives,^22,23^ supported by its consistent relevance in both traditional and scientific contexts.

Despite its importance, the NMR data available in the literature for natural products, including KA, often contain numerous unassigned hydrogen signals, limited multiplicity information, and incomplete coupling constant measurements. As a result, many publications report ambiguous, imprecise, or even incorrect data sets.^35–38^ For instance, Montiel-Ruiz et al.^39^ published in 2020 a study on the biological effects of natural products—including KA in the title and keywords—yet presented only four hydrogen chemical shifts, citing as reference a work that reports data solely for KA analogues.^39^ Their Supporting Information includes spectra but no tabulated NMR data. Additional examples of incomplete KA data sets are shown in the Supporting Information (page S4).

Such incomplete data are especially common for less functionalized structures, in which the extensive overlap of CH_2_–related signals creates major challenges for accurate interpretation. Consequently, many studies involving reisolated compounds rely solely on these limited data sets without further clarification.^35,40^ For KA, only six hydrogen signals—mainly olefinic, methyl, and bridgehead hydrogens—are frequently cited as structural references, and most structural confirmations depend primarily on ^13^C NMR rather than ^1^H NMR data.^41^ The scarcity of complete and reliable data sets, as highlighted by the number of works addressing NMR data corrections,^42–45^ significantly limits progress in the structural elucidation of diterpenes.

The issue of coupling constant determination is particularly critical in spectra with extensive signal overlap, as often seen for diterpenes, making unambiguous interpretation extremely challenging. Poorly functionalized molecules represent the most difficult cases: the fewer functional groups, the greater the overlap and the harder it becomes to fully resolve ^1^H NMR signals. Historically, compounds with complex spectra have rarely been reported with complete NMR assignments.

To overcome these difficulties, computational tools can assist in clarifying complex splitting patterns.^45^ Modern NMR instruments provide numerous efficient experimental options, but in many cases, simulated spectra are indispensable for confirming signal structures. For first-order ^1^H NMR signals (without distortions), simulations can be performed using the FOMSC3 program (First Order Multiplet Simulator/Checker), while second-order couplings are better addressed using the SimEsp_NMR software. Both programs were developed at the Laboratory of Organic Synthesis (FFCLRP/USP) and are freely available.^46^

In this work, we present the complete ^1^H and ^13^C NMR data assignments for KA, performed concurrently with those for its methyl ester, KAMe, to enhance the reliability and clarity of the process. The use of FOMSC3 and SimEsp_NMR software formed a central part of the methodology, previously validated for other diterpenes.^45^ Additionally, spectra were recorded in different deuterated solvents to reveal hidden features and to generate a more comprehensive and useful data set.

## Materials and Methods

### Isolation of *ent*-Kaurenoic Acid

The *ent*-kaurenoic acid sample used in this study was isolated from a commercial source, *Mikania glomerata* Spreng (Asteraceae), obtained from the Brazilian company Nutri Ervas, Ltd.a, as described in our previously published work.^15^

### NMR Experiments

^1^H NMR, ^13^C­{^1^H} NMR, DEPT-135, gCOSY, gHSQC, gHMBC, NOESY, and *J-resolved* experiments were performed. The NMR spectra were recorded on Bruker spectrometers models AVANCE DRX400, 9.4 T (^1^H SFO1 = 400.21 MHz and ^13^C SFO2 = 100.63 MHz) and AVANCE DRX500, 11.7 T (^1^H SFO1 = 500.13 MHz and ^13^C SFO2 = 125.76 MHz); a 5 mm inverse probe (BBI), operated at 27 °C (300 K), was employed. ^1^H NMR spectra were acquired with an SWH of 8.28 kHz, TD of 64K and NS of 16, providing a digital resolution of ca. 0.126 Hz (^1^H 30° pulse width = 8.5 μs). For ^13^C NMR spectra, an SWH of 23.98 kHz was used with a TD of 32K and NS of 1024, giving a digital resolution of ca. 0.732 Hz (^13^C 30° pulse width = 14.25 μs). DEPT (512 scans), ^1^H/^1^H and ^13^C/^1^H 2D chemical shift correlation experiments were performed using standard pulse sequences supplied by the spectrometer manufacturer. Long-range ^13^C/^1^H chemical shift correlations were obtained in experiments with delay values optimized for ^2^ *J*(C,H) = 8 Hz. The deuterated solvents used were chloroform-*d* (CDCl_3_), methanol-*d*_4_ (CD_3_OD), benzene-*d*_6_ (C_6_D_6_) and pyridine-*d*_5_ (C_5_D_5_N), all referenced with 0.03% TMS. All samples were prepared at concentrations ranging from 3.5 to 25 mg/mL, depending on the pulse sequence of the NMR experiment. When possible, less concentrated samples were prioritized to achieve higher resolution. The 2D experiments were conducted with pulse sequences provided by the spectrometer manufacturer.

### NMR Spectral Processing

The NMR experiments conducted were processed using the ACD/Spectrus Processor software through an academic version obtained free of charge from the Internet,^47^ with no usage restrictions. This program processes the acquired data, enhances spectral signals, and performs measurements in both 1D and 2D NMR experiments. In conjunction with the ACD/Spectrus Processor software, the SpinWorks program was also utilized for processing the obtained NMR analyses. This software is freely available and can be accessed via the Internet.^48^ For ^1^H NMR spectra, the processing parameters were TD = 65536; SW = 8503,40 Hz; NS = 64; LB = 0,00; GF = 0,00. For ^13^C NMR spectra, the processing parameters were TD = 65536; SW = 31446,54 Hz; NS = 5120; LB = 1,00; GF = 0,00.

### NMR Signal Simulations

The signals and spectra were simulated with the programs FOMSC3_rm_NB and NMR_MultSim, available free of charge,^46^ following our methodology recently published.^45^

### Data Set Comparison to Structure

Aiming to verify if there was a reliable consistency of data with structural features, all the assignment work was carried out in parallel with evaluation of physical molecular models and the use of simple 3D molecular modeling techniques to confirm some structural features hypothesis. For these purposes, the trial license of ChemDraw 3D software, included in the installation package of ChemDraw Professional was used as well as the ChemDraw NMR data calculations.^49^

## Results and Discussion

The first approach we employed was through the ^1^H NMR experiment using deuterated chloroform, which is the most affordable deuterated solvent and most commonly used for this type of compound. The ^1^H NMR spectrum of KA, in CDCl_3_, is shown on Figure 1.

**2:**
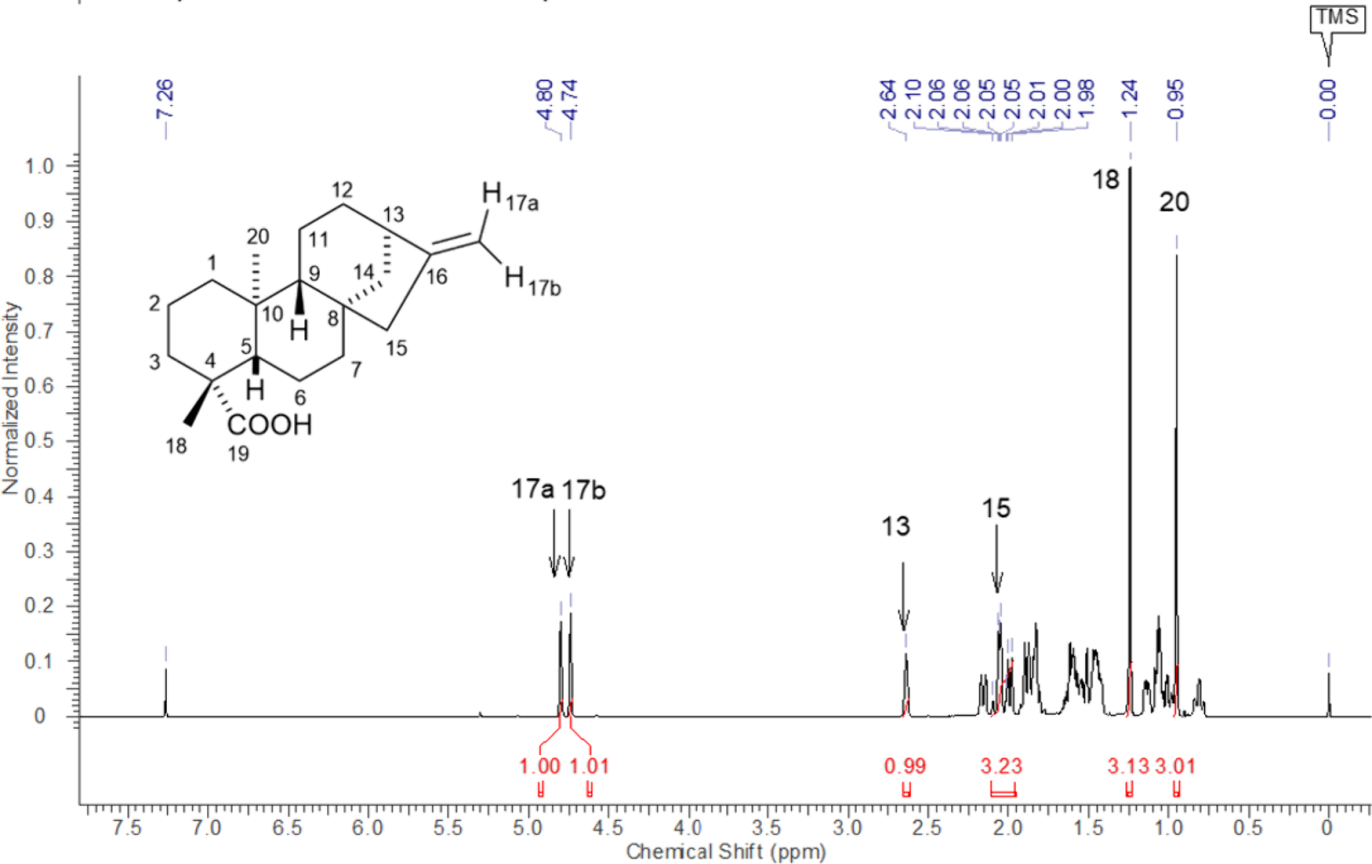
^1^H NMR spectrum of *ent-*kaurenoic acid (KA), CDCl_3_, 500 MHz.

The most readily assigned resonances corresponded to the methyl protons H-18 and H-20, the olefinic protons H-17a and H-17b, the bridgehead proton H-13, and the methylene protons H-15. These few signals are commonly used to confirm the KA scaffold, since ^13^C NMR signals are often prioritized^41^ because they are less complex and show less signal overlap. However, H-17a and H-17b are frequently reported only as an undifferentiated pair of olefinic protons; to obtain distinct assignments we therefore applied NOE experiments. As shown in Figures S111–S114 (Supporting Information), H-17a is unambiguously cis to C-13, in agreement with Enriquez and his co-workers.^50^

We then proceeded to fully assign the remaining resonances and to determine all relevant coupling constants (J values). Some J values could be measured directly from the ^1^H NMR spectra, while others required alternative approaches. The methodology previously developed in our group^45^ combines: (i) careful measurement of J values from high-resolution ^1^H spectra; (ii) J-resolved experiments when direct measurement is hindered by overlap; and (iii) simulation of NMR signals to extract unresolved J values.^46^

For direct measurement in ^1^H spectra, well-resolved signals are essential; consequently, spectra were recorded from highly diluted KA solutions in CDCl_3_. Expansion of the relevant spectral regions allowed measurement of most coupling constants. Based on prior assignments,^41,50^ H-15 resonances were expected around 2.05–2.09 ppm, although some reports list a single chemical shift for this methylene. This motivated a closer inspection of the ^1^H spectral region shown in Figure 3.

**Figure 3:**
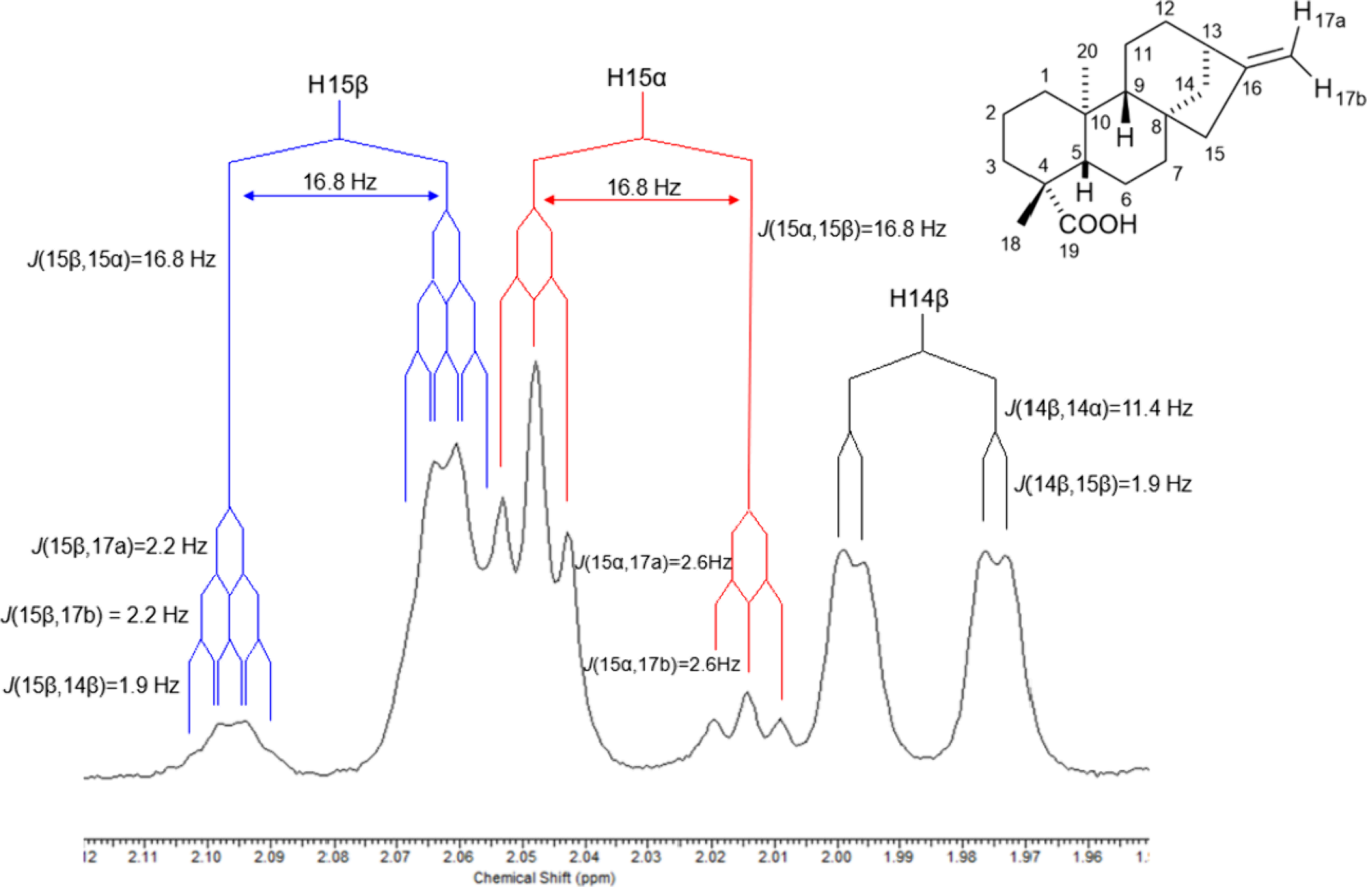
^1^H NMR spectrum expansion of H-14β, H15α, and H15β signals from *ent-*kaurenoic acid (KA), CDCl_3_, 500 MHz, exemplifying the *J* value measurement.

At first inspection, the group of peaks slightly above 2.0 ppm may appear as a single resonance with an overall intensity of two protons. Consequently, this region is often assigned to H-15, assuming both hydrogens are chemically equivalent. However, the asymmetry of this multiplet demonstrates that it actually corresponds to two distinct signals. A closer examination at higher intensity reveals the second component of each resonance, separated by 16.8 Hz, which corresponds to the geminal coupling.

In this region, the J values of H-15α and H-14β can be clearly measured, whereas not all coupling constants of H-15β can be accurately determined from the ^1^H NMR spectrum alone. Even though precise measurement of all J values was not possible, the spectral pattern indicated an inconsistency: H-15α and H-15β displayed different splitting behaviors. The latter exhibited four couplings, while the former showed only three. This is unusual for two protons bound to the same carbon atom, which at this stage were still considered as H-15a and H-15b, without stereochemical distinction. Nevertheless, such inequality is consistent with the rules of homonuclear spin–spin coupling.

This observation emphasized the importance of performing the ^1^H and ^13^C NMR assignments in conjunction with a careful evaluation of the molecule’s three-dimensional structure. The analysis was carried out using physical molecular models and simple 3D structural calculations available in ChemDraw3D software.^49^ For the hydrogens at position 15, a W-type coupling between one H-15 and one H-14 proton was identified. This coupling allowed us to assign this hydrogen as H-15β, which in turn enabled the identification of H-14β, as illustrated in Figure 4. This finding guided the subsequent assignment of the relative stereochemistry of other methylene hydrogens in the molecule. Three additional ^4^ *J*_W_ couplings were identified, as shown in Figures S6, S11, S14, and S40 of the Supporting Information.

**Figure 4:**
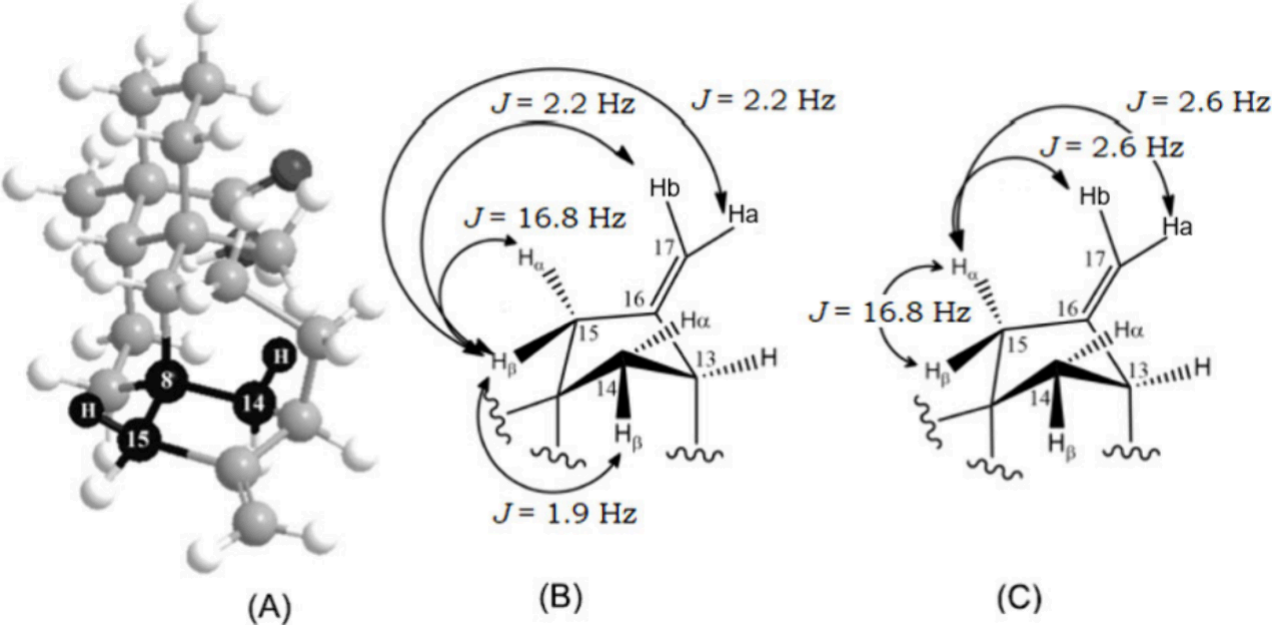
Example of the use of 3D models to track assignment with (A) the 3D image showing ^4^ *J*_w_ (15β,14β) and all expected couplings for H15β (B) and H15α (C).

Prior to the determination of coupling constants for the H-15 hydrogens, the presence of two distinct resonances near 2.06 ppm and their correct attribution to the H-15 protons were confirmed by analysis of the g-HSQC spectrum (Figure 5). All subsequent assignments were performed following the same methodology.

**Figure 5:**
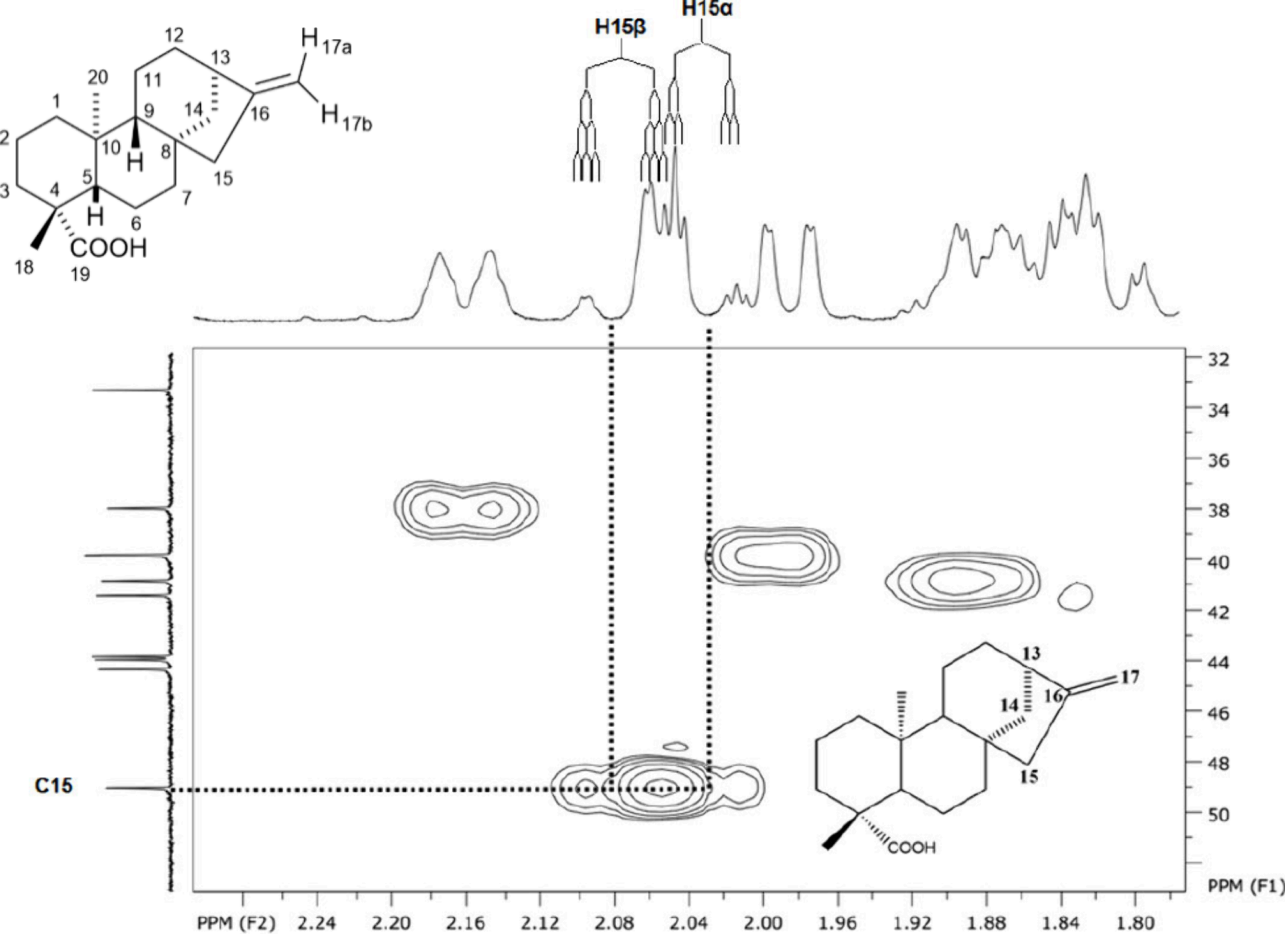
Expansion of the g-HSQC spectrum from *ent-*kaurenoic acid.

As previously discussed, it was essential to compare experimental measurements with 3D molecular geometries, either from physical models or computational structures—at every stage of the analysis. Dihedral angles (related to ^3^ *J*_HH_ values) were continuously evaluated using freely available software.^51^

In contrast to the H-15 case, closer examination of the H-13 resonance revealed a situation where almost none of the coupling constants could be directly measured from the ^1^H NMR signal (Figure 6).

**Figure 6:**
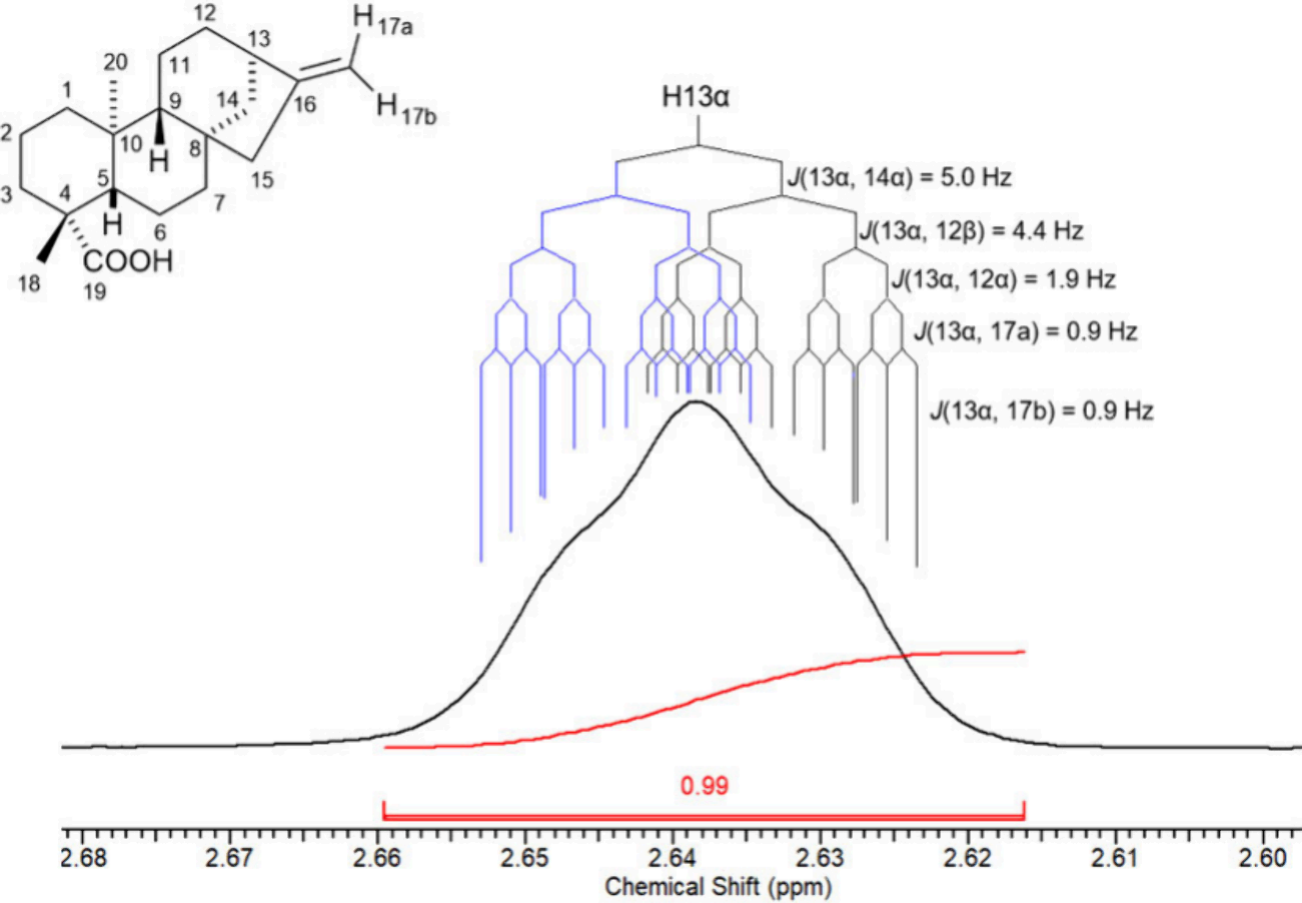
^1^H NMR spectrum expansion of the H-13 signal from *ent-*kaurenoic acid (KA), CDCl_3_, 500 MHz.

The H-13 proton is expected to couple with H-12α, H-12β, H-14α, and H-14β, and possibly to exhibit long-range interactions with H-17a and H-17b. However, despite the high resolution and detailed spectral expansion, none of these couplings produced discernible peak splittings in the H-13 resonance. The superposition of multiple *J* values resulted in a signal shape that prevented direct measurements.

Such cases required an alternative strategy involving simulated spectra and comparison between calculated and experimental line shapes. For these analyses, g-COSY experiments were essential to identify which coupling constants should be considered in the simulations.

To determine all coupling constants in complex signals, the first step was to measure every possible homonuclear ^1^H–^1^H coupling directly from the ^1^H NMR spectra. In some cases, the H_A_–H_B_ coupling constant could not be measured in the H_A_ resonance but was clearly observable in the H_B_ signal. Because the frequency difference (in Hz) between the first and last peak of a multiplet equals the sum of all contributing *J* values, one or two coupling constants could often be inferred even when not directly measurable.

Once most of the *J* values were experimentally obtained or estimated, the accuracy of these data was verified using signal-simulation software such as FOMSC3 and NMR_MultSim. Comparison between simulated and experimental spectra confirmed the reliability of the assignments, as evidenced by the excellent agreement between the calculated and observed line shapes and chemical shift spacings.^45^

In many cases, however, severe signal overlap in the ∧1H NMR spectrum (recorded in CDCl_3_) hindered the complete and unambiguous determination of all coupling constants. To overcome this limitation, spectra were recorded in alternative solvents. Because solvent effects alter chemical shifts but leave J values essentially unchanged, this approach allowed clarification of true signal multiplicities. The resulting nonuniform chemical-shift variations—some signals shifting upfield and others downfield—reduced or even eliminated overlapping in previously congested regions. This improvement enabled isolation of specific resonances and facilitated accurate measurement of homonuclear coupling constants. Figure 7 illustrates the variation in chemical shifts observed across different solvents.

**Figure 7:**
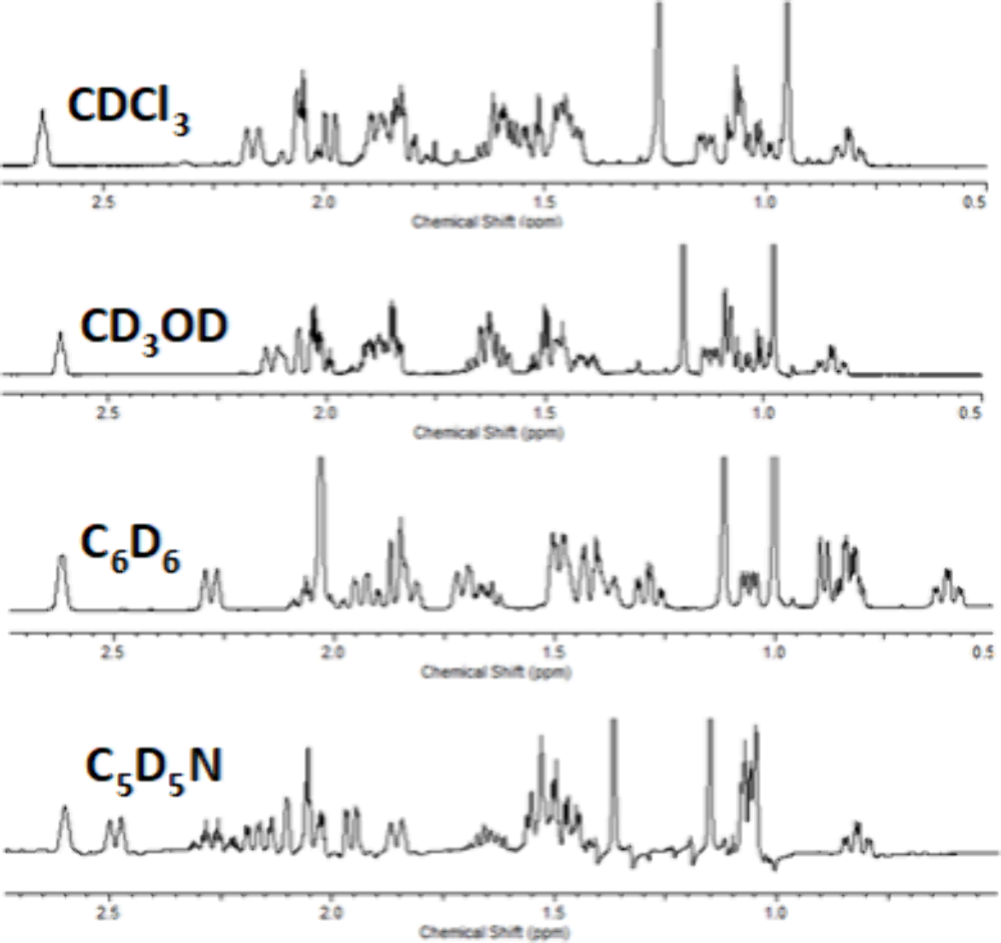
KA ^1^H NMR spectra in different solvents for comparison. Methyl signals were cut to fit the figure.

Comparison of spectra recorded in different solvents revealed variations in the chemical shifts of several signals, ranging from minor to pronounced. For instance, the CH_3_–20 resonance appeared at 0.95 ppm in CDCl_3_ and at 1.15 ppm in pyridine, representing a significant solvent-induced shift. In contrast, the same signal showed only a slight difference when spectra in chloroform and methanol were compared.

These variations in chemical shifts facilitated the isolation of signals that were previously overlapped, enabling measurement of several homonuclear coupling constants that had been hidden in the CDCl_3_ spectrum. This improvement allowed a detailed analysis of each region of the ^1^H NMR spectrum of KA. The signal analyses were performed with the aid of freely available software, FOMSC3 and MultiSim_NMR.^46^

Despite the increased signal resolution, the intrinsic spectral complexity still prevented complete and fully precise measurements. The aforementioned software packages are specifically designed to address this challenge, accurately simulating ^1^H NMR spectra from input parameters such as chemical shifts and coupling constants. When uncertainty remained regarding a given coupling constant, spectra were simulated with two or three closely related J values to determine the one that best reproduced the experimental data. The final assignments were established through comparison of simulated and experimental spectra, where agreement in peak positions and overall line shapes confirmed the correctness of the parameters. An example of this procedure is presented in Figure 8.

**Figure 8:**
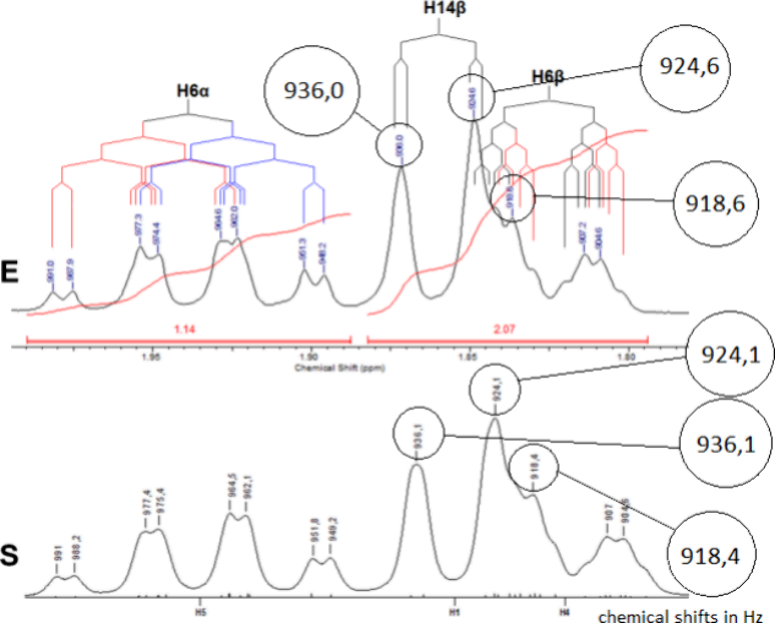
Experimental (E) versus simulated (S) spectra for hydrogens H-6α and H-14β. The chemical shifts (Hz) are shown magnified in larger circles, to enable reading.

The close resemblance between the simulated and experimental signal shapes is evident. The individual peak positions also show excellent agreement, with a maximum deviation of only 0.5 Hz. This confirms the accuracy of the assigned chemical shifts and homonuclear coupling constants. These procedures were performed for every proton, using the most suitable solvent in each case, yielding the data set summarized in Table 1.

**Table 1:**
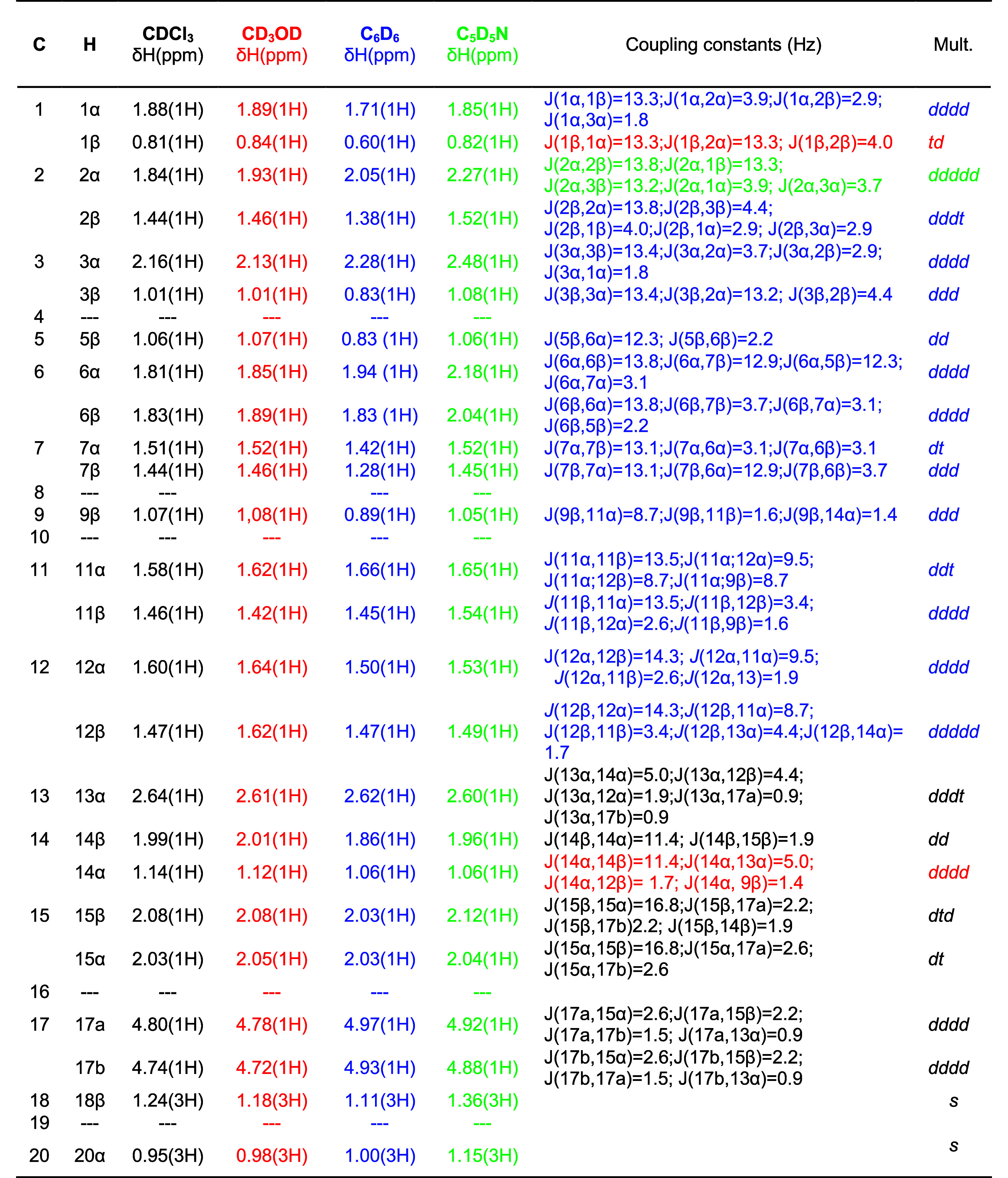
Complete ^1^H NMR Data for *ent*-Kaurenoic Acid (**1**) (500 MHz)^a^

a Font colors indicate which solvent used in experiment provided the data.

A complete comparison between experimental and simulated spectra for all cases is provided in the Supporting Information (Figures S5–S35 and S38–S42). This material also includes several 3D computational representations illustrating verified couplings for each signal. Furthermore, it contains a 2D NMR data set section (Tables S1–S5), an extensive spectral section and data comparison segment (Tables S6–S16), and a summary of results. Finally, a tutorial section is provided, offering step-by-step guidance on the use of the employed software.

Once the data set was established, we aimed to compare it with NMR data reported in the literature. A search using the keywords “kaurenoic acid” and “NMR” was performed in SciFinder and Web of Science. Fourteen relevant articles published over the past three decades were identified (references 3–16 in the Supporting Information). Most of these works are relatively recent, with 80% published within the last 14 years and 57% within the past decade. For organizational reasons, they are cited as a group in the Supporting Information, where all their reported ^1^H NMR data are compiled in Tables S9–S13. Each table presents the data side by side with the corresponding values obtained in this work.

Among these 14 references, only one provides a data set that could be considered reasonably complete; even in this case, only few signal multiplicities were reported [ref. S13]. In fact, none of the studies presented complete multiplicity assignments or measured coupling constants. Between the remaining 13 articles, including some very recent ones, five of them omit between 12 and 22 proton chemical shifts [refs. S4, S5, S6, S8, S14]. One of the data set with fewest omissions also contains one assignment error [ref. S5], while six others show inverted or duplicated chemical shifts for equivalent hydrogens in CH_2_ groups [refs. S3, S7, S9–S11, S16]. One article appears to suffer from calibration issues, as several values deviate significantly from all others [ref.S9].

Therefore, the ^1^H NMR data set reported here represents the most complete and detailed analysis of kaurenoic acid available to date. For ^13^C NMR assignments, most cases could be confidently clarified using g-HSQC and DEPT-135 (Figure 9) experiments, as described below.

**Figure 9:**
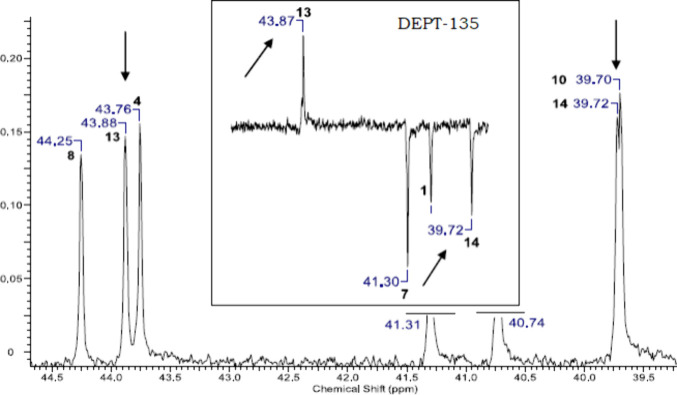
Example of the use of DEPT-135 experiments.

The pairs C-4/C-13 and C-10/C-14, which display the closest chemical shift values in the ^13^C NMR spectrum, were unexpectedly not problematic. They were easily distinguished using the DEPT-135 experiment, which also served to confirm several other carbon assignments.

The use of g-HSQC experiments (previously exemplified in Figure 5) was further improved by recording spectra in different solvents. Correlations that could not be observed in the g-HSQC spectrum of one solvent were clearly verified in another, enabling the assignment of most ^13^C NMR signals.

For quaternary carbons and other more challenging cases, g-HMBC experiments were required. One of the most intricate examples was the correct assignment of C-7 and C-12. Although some studies do not report ^13^C NMR data for KA,^41^ and others—despite focusing on structure elucidation—assign these signals incorrectly,^50^ a careful inspection of the g-HMBC spectrum in this work provided sufficient information for an unambiguous assignment, as illustrated in Figure 10.

**Figure 10:**
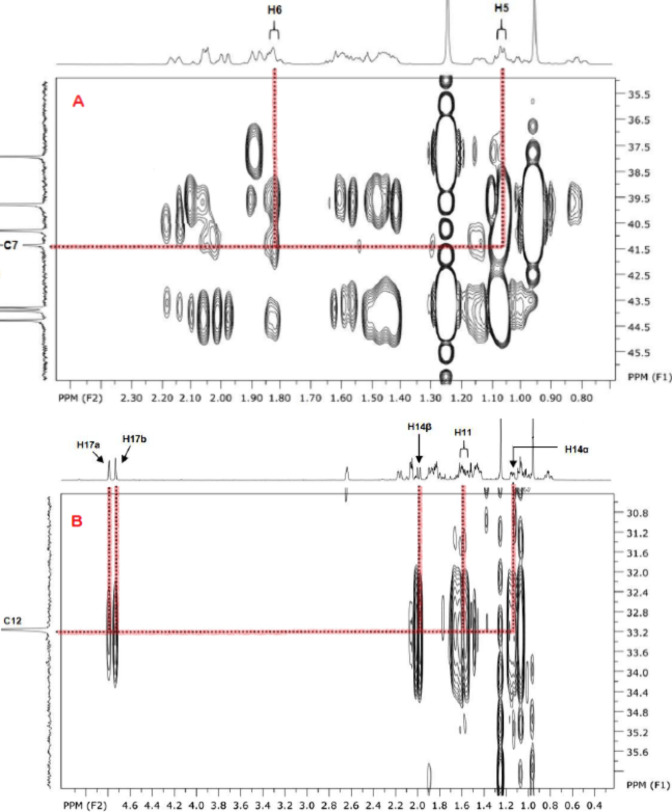
Application of g-HMBC experiments to clarify the assignment of quaternary carbons. The cases of C-7 (A) and C-12 (B) are highlighted.

After a thorough assignment and meticulous data verification using the ^13^C NMR and 2D-NMR spectra, a comprehensive ^13^C NMR data table was compiled (Table 2).

**Table 2:**
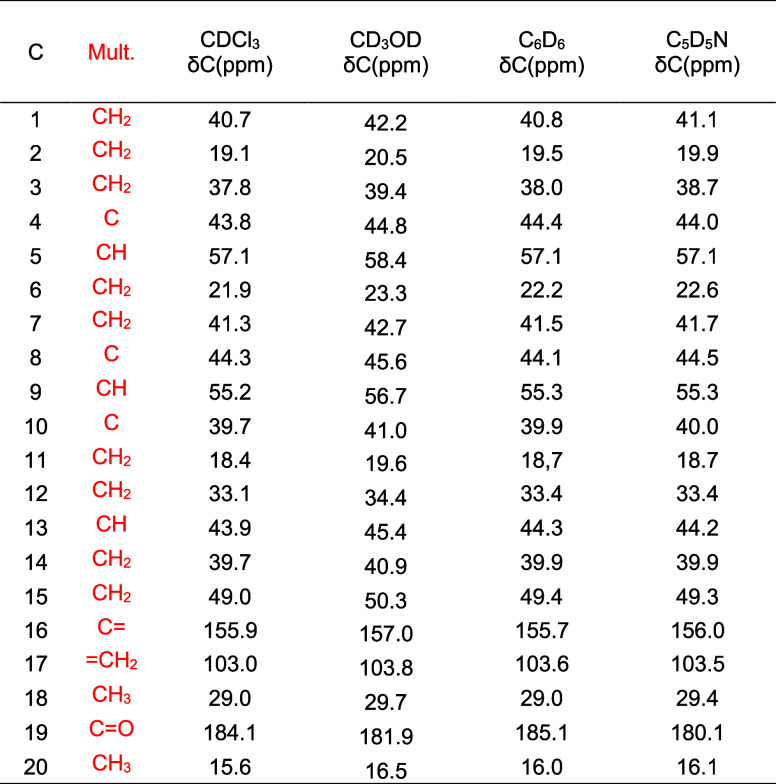
Complete ^13^C NMR Data for *ent*-Kaurenoic Acid (**1**) (500 MHz)

The comparison of ^13^C NMR data was conducted with the same group of references used for ^1^H NMR and is also available in Supporting Information (Tables S12–S15). In the case of carbon NMR data, more complete data sets are available due to the inherent simplicity of the data and spectra. Carbon NMR data is more commonly utilized as a reference for structural identification than proton NMR data for certain natural products. Of the 13 references that present ^13^C NMR data for KA, eight are considered to contain complete and well-assigned data. However, one relatively recent reference provides minimal data, with only four carbon assignments. Additionally, two references contain one unassigned carbon, and three others feature erroneous assignment, including inverted values. Therefore, the complete and unequivocal ^13^C NMR data assignment for KA presented in this work can be regarded as a significant contribution to the literature. All assignments were corroborated by multiple experiments and ^13^C NMR chemical shifts were carefully determined across four different solvents. All 2D-NMR data for kaurenoic acid (1) are presented on Tables S1 to S4 in Supporting Information. The same methodology was applied to methyl-ent-kaur-16-en-19-oate, KAMe (2). The idea of using a very similar structure along with KA was to gradually assign both structure signals and to verify differences such as coupling constants values, conformation, and dihedral angles. The plan was to carry out the assignments by comparing both diterpenes. Clearly KA is much more studied and utilized as a research target, which is why it was presented first. Furthermore, KA was the main focus of this work. As found for kaurenoic acid, KAMe also has just a few 1H-NMR signals commonly assigned. Some papers describe only six hydrogen assignments, as shown in Figure 11.

**Figure 11:**
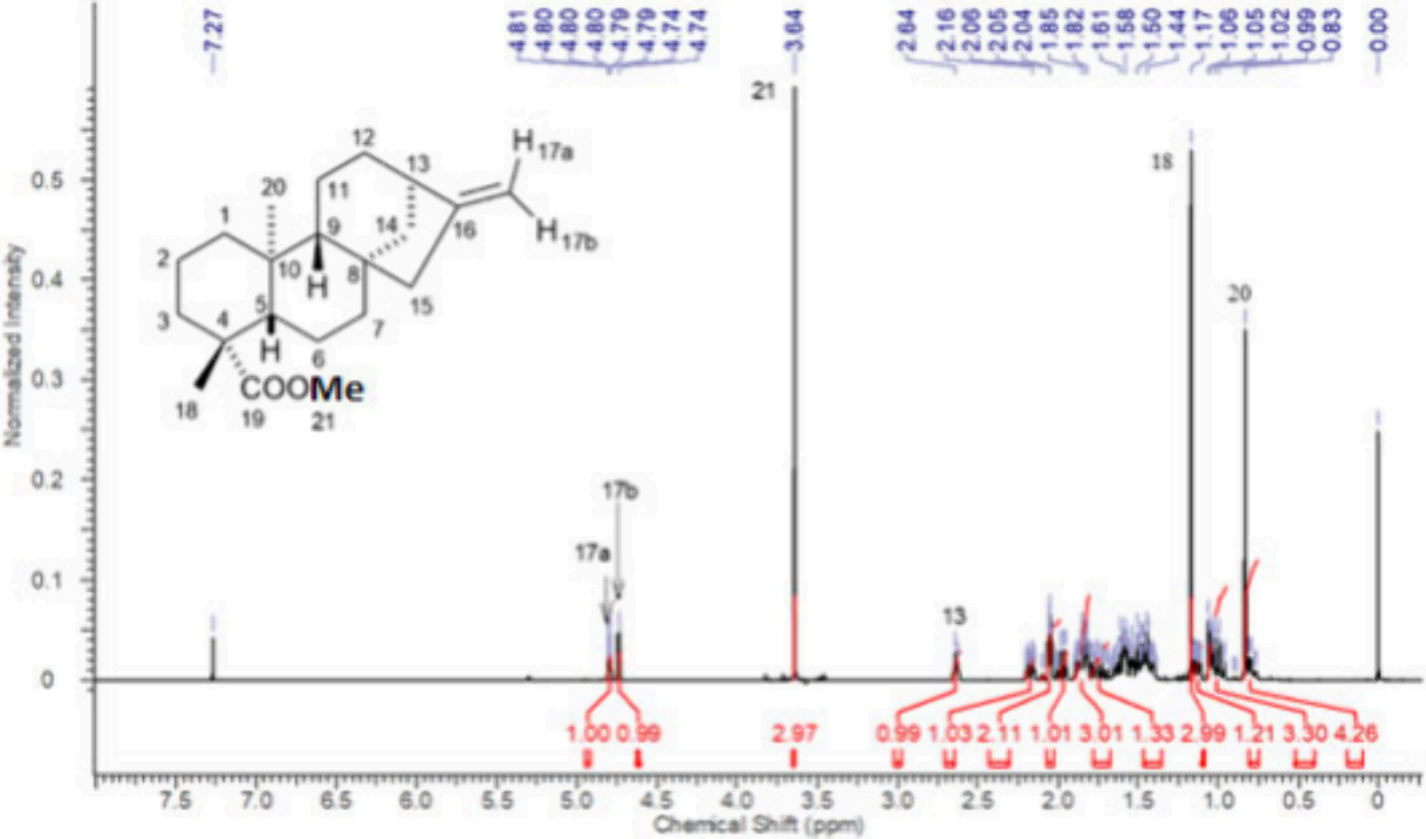
^1^H NMR spectrum of *ent-*kaurenoic acid methyl ester (KAMe), CDCl_3_, 400 MHz.

With the same attention to detail as carried out for KA, the search for the detailed analysis of ^1^H and ^13^C NMR data for kaurenoic acid methyl ester (KAMe) was conducted. All hydrogen signals were expanded and meticulously analyzed aiming to reach the multiplicity determination and the homonuclear hydrogen coupling constants measurement. This search is exemplified in Figure 12.

**Figure 12:**
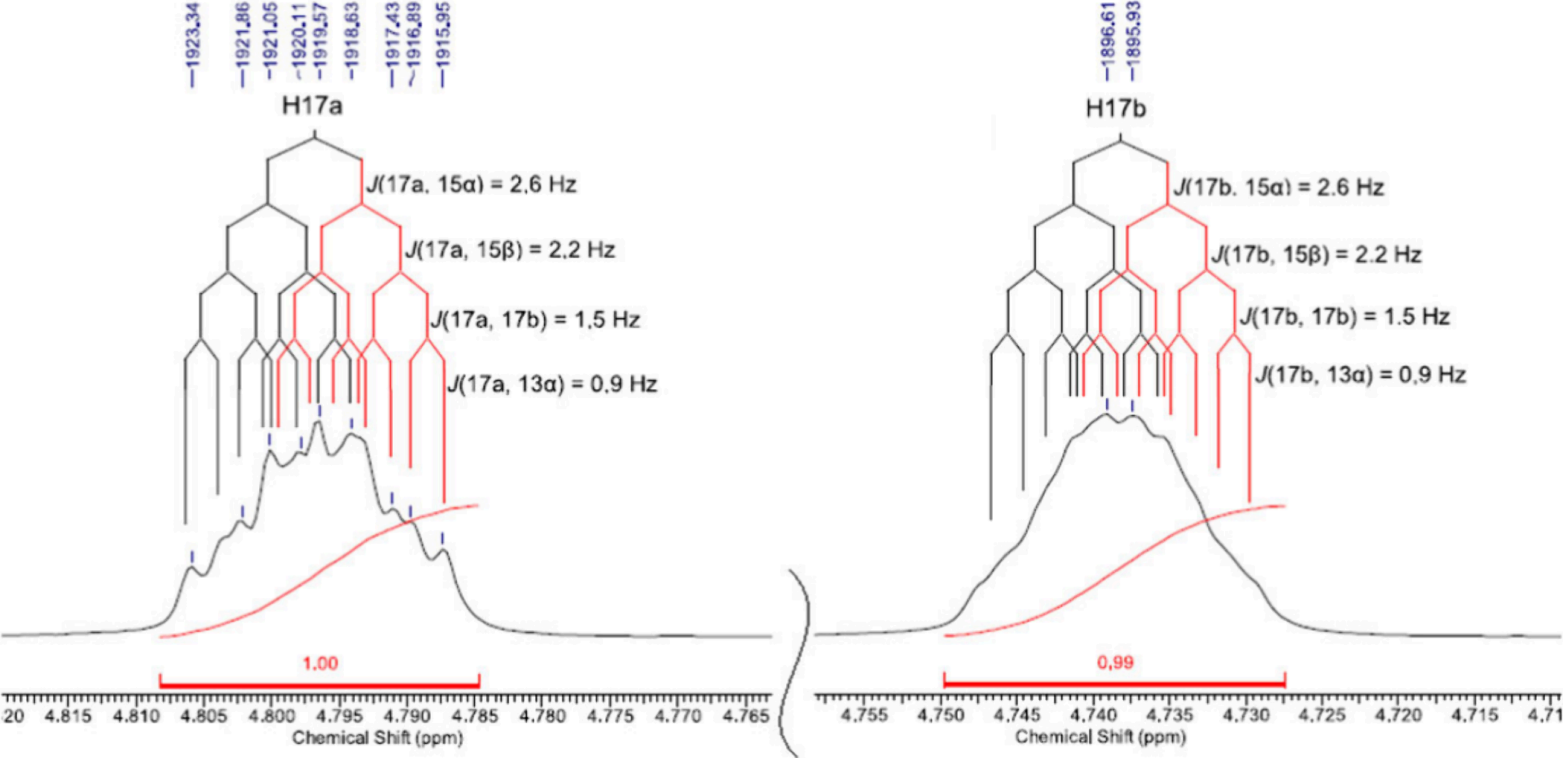
Expansions of ^1^H NMR signals H-17a and H-17b of *ent-*kaurenoic acid methyl ester (KAMe), CDCl_3_, 400 MHz.

The double bond between C-16 and C-17 enables some long-range couplings, which make H-17a and H-17b signals exhibit a dddd multiplicity. Achieving these *J* values was done by the same methodology as described for KA. More simulated and experimental signal plots can be seen on Supporting Information (Figures S5–S35 and S38–S42). This case was also conducted with the assistance of the software, and different solvents were used as well. The difference is that for KAMe only CDCl_3_ and C_6_D_6_ were used, but the thoroughness was the same as the previous case. All the ^13^C NMR signals were assigned using DEPT-135, g-HSQC and g-HMBC for assignments and verification. In the Supporting Information there is a spectral section for KAMe (2), as was done for KA, where a complete set of spectra can be viewed. The ^1^H and ^13^C NMR data obtained for this substance are organized in Tables 3 and 4. The data set presented in this table is also the most complete and detailed set of NMR data for methyl kaurenoate (KAMe), including data obtained in two different solvents, a complete set of hydrogen homonuclear coupling constants values, and the clarification of the entire multiplicity.

**Table 3:**
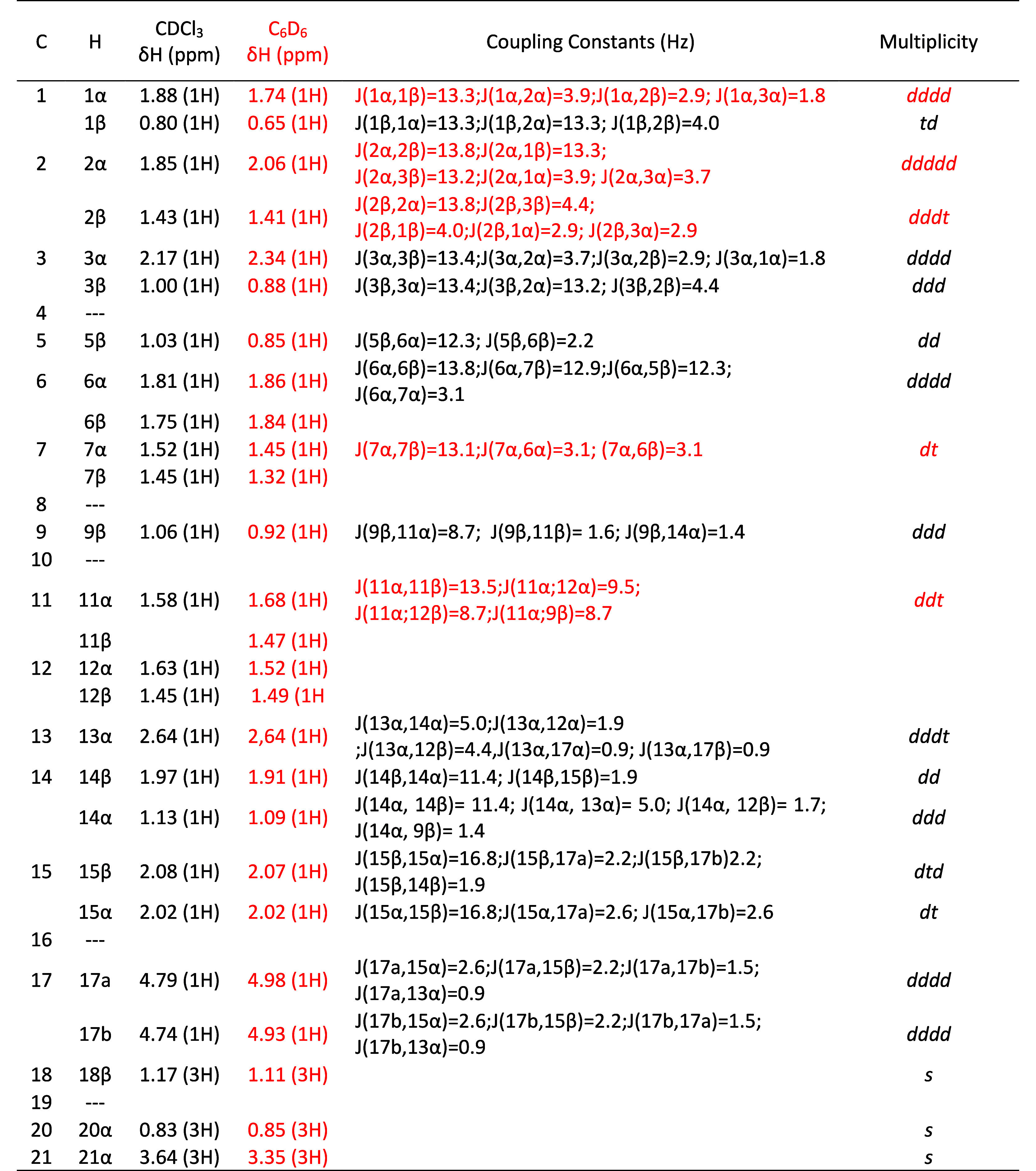
Complete ^1^H NMR Data for *ent*-Kaurenoic Acid Methyl Ester (**2**) (500 MHz)^a^

a Font colors indicate which solvent used in experiment provided the data.

**Table 4:**
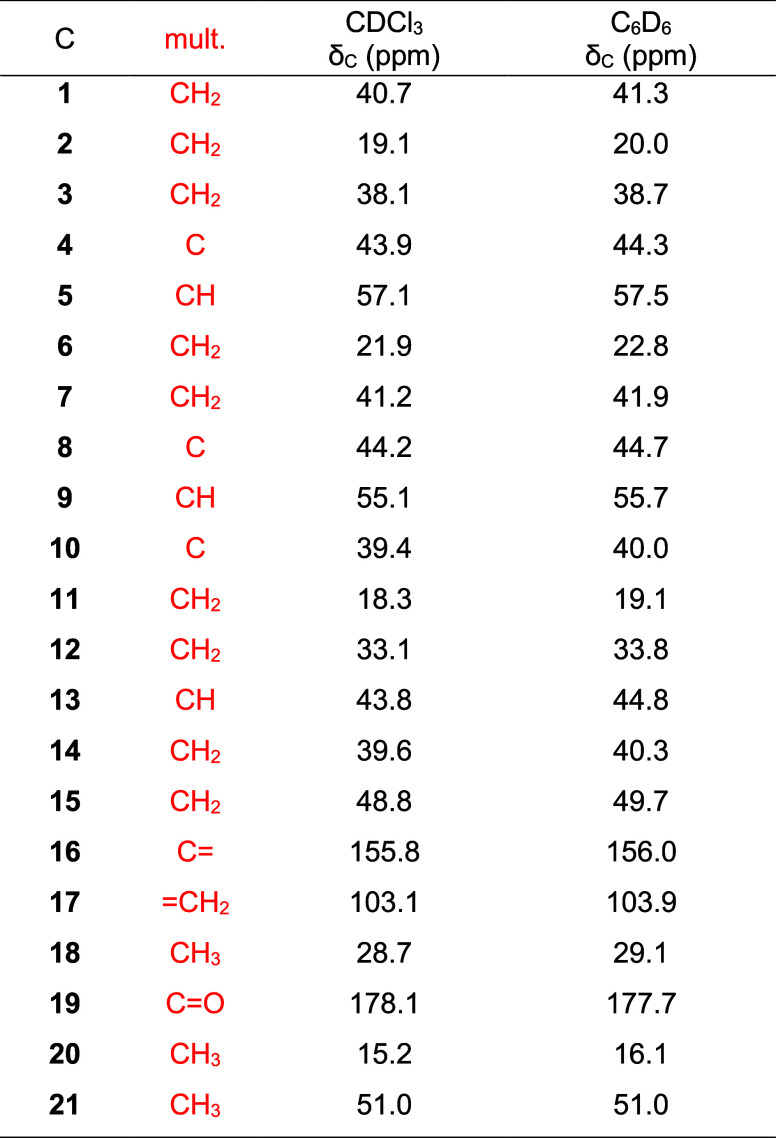
Complete ^13^C NMR Data for *ent*-Kaurenoic Acid Methyl Ester (**2**) (500 MHz)

## Conclusions

The complete assignment of ^1^H and ^13^C NMR data was achieved for two analogous diterpenes – kaurenoic acid (KA) and its methyl ester (KAMe). The methodology, developed and continuously refined within our research group, proved to be a robust approach for the detailed elucidation of ^1^H and ^13^C NMR spectra of poorly functionalized molecules. The NMR data of KA and KAMe represented one of the most challenging assignments due to their structural features. For both compounds, all hydrogen and carbon chemical shifts were identified, including individual assignments of each diastereotopic hydrogen. In addition, all ^1^H–^1^H coupling constants were determined, and the multiplicities of every proton resonance were unambiguously established. No previous studies on KA in the literature provide this level of spectral detail or describe the step-by-step procedures adopted here, underscoring the novelty of this work. These results also highlight the potential of this methodology to be applied to other natural products with scarce NMR data, enabling more comprehensive and accurate spectroscopic analyses.

## Supplementary Material



## Notes

Structures, IUPAC name, and 3D figures for the studied compounds; complete spectral section, including spectra expansions for both compounds; examples of comparison between calculated and experimental spectra; complete comparison of the data obtained in the present work with literature available data for KA; full tutorial section for the use of FOMSC3 and NMR_MultiSim programs (PDF)
