# Supplementary material for: The Challenging Complete and Detailed 1H and 13C NMR Assignment for ent-Kaurenoic Acid, a Remarkable Natural Product
Source: ACS Omega. 2025 Nov 20;10(48):59500–12. doi: 10.1021/acsomega.5c09155 (PMC12771156; doi:10.1021/acsomega.5c09155)
Supplement: Supplementary file 1 [file ao5c09155_si_001.pdf]

Supplementary Material

The challenging complete and detailed ^{1}H and ^{13}C NMR assignment for *ent*-kaurenoic acid, a remarkable natural product

Alexsandro Eurípedes Ferreira, Ana Carolina Ferreira Soares Rocha, Julian Carlos da Silva Pavan, Viviani Nardini Takahashi, Herbert Júnior Dias, Patrícia Mendonça Pauletti, Daiane Cristina Sass and Vladimir Constantino Gomes Heleno

This Supplementary Material contains: **page**

IUPAC Nomenclature and structure of kaurenoic acid.	**2**
3D Figures of kaurenoic acid.	**3**
Examples of data scarcity for kaurenoic acid even with varied NMR techniques available	**4**
I. Examples of comparison between experimental and simulated signals for kaurenoic acid.	**5**
IUPAC Nomenclature and structure of methyl kaurenoate.	**36**
3D Figures of methyl kaurenoate.	**37**
I. Examples of comparison between experimental and simulated signals.	**38**
II. A 2D NMR data set (Tables).	**43**
III. Spectral section.	**48**
IV. A data comparison section with tables showing the present data and previously published data.	**95**
V. Summary of Results.	**107**
VI. Tutorials Section	**112**
REFERENCES	**137**

IUPAC NOMENCLATURE FOR *ENT*-KAURENOIC ACID

***Ent*-kaurenoic acid (*Ent*-kaur-16(17)-en-19-oic acid):**

(1S,4S,5R,9S,10R,13R)-5,9-dimethyl-14-methylidenetetracyclo[11.2.1.01,10.04,9]hexadecane-5-carboxylic acid

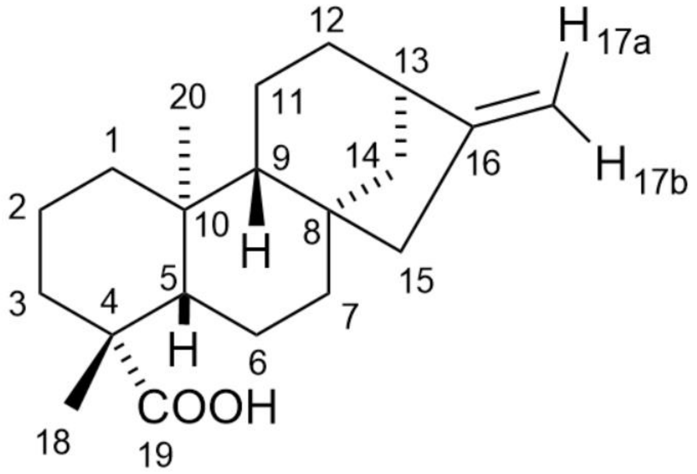

Figure S1. Structure with detailed numbering of *ent*-kaurenoic acid

Source: HIPOLITO *et al.*, 2011 [1]

3D figures of *ent*-kaurenoic acid

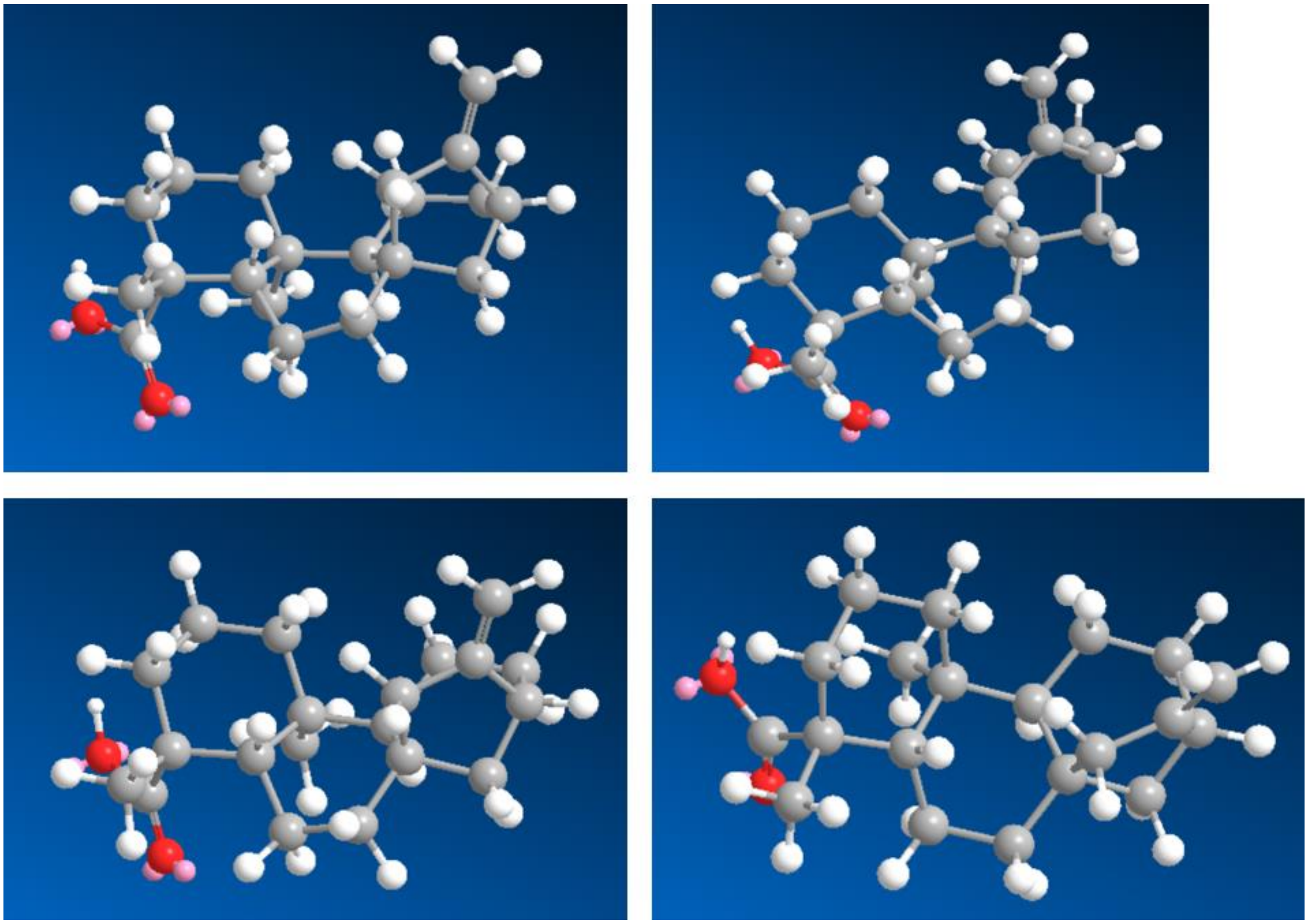

Source: Chem3D – MM2.

Examples of data scarcity for kaurenoic acid even with varied NMR techniques available*

*Section to exemplify the first four lines of the seventh paragraph of the article.

This Section is about the scarcity of KA NMR data in literature in several publications and its incomplete set of NMR data presented, even in articles published at a time of full development of NMR techniques. The habitual use of “data comparison with literature” in each work carried out with KA (or any other NP with complex spectra) causes this situation.

As a first example, one can observe reference 41 in the article, a paper from 2001, which refers to kaurene diterpenes in its title, but present for kaurenoic acid only 6 hydrogen signals, none of which with coupling constants (see ref. 41). This paper was commonly used as reference for NMR data comparison for KA identification. Other examples are the Montiel-Ruiz and coworkers’ paper, cited in the article as reference 39, and the examples of Chen and coworkers[1], Móricz and coworkers[2], and Santos and coworkers[3] papers, all from 2018. The paper cited in the article as reference 39 cites only four hydrogen chemical shifts, besides not presenting any tabulated data in the supplementary material, only spectra. Chen’s work cites the same four chemical shifts plus the H13 chemical shift, but as a broad simplet (without coupling constants), even though the spectrum was recorded in a 600 MHz spectrometer. Móricz’s work has the best NMR dataset for KA of all five references. The only limitations in this work were: to assign only one signal for both H-11; to provide only 3 coupling constants values; and to use the solvent as reference. Finally, Santos’ work cites only 5 hydrogen and four carbons chamical shifts. On the other hand, it cites a reference to which a comparison was made. Nevertheless, this reference presents only chemical shifts also without coupling constants.

---

[1]Chen, Q.; Lin, H.; Wu, X.; Song, H.; Zhu, X. Preparative separation of six terpenoids from *Wedelia prostrata* Hemsl.by two-step high-speed counter-current chromatography. *J. Liq. Chromatogr. Relat. Technol.*, **2018**, *41*, 408-414.
[2]Móricz, A.M.; Ott, P.G.; Yüce, I.; Darcsi, A.; Béni, S. Effect-directed analysis via hyphenated high-performance thin-layer chromatography for bioanalytical profiling of sunflower leaves. *J. Chromatogr. A*, **2018**, *1533*, 213-220.
[3]Santos, J.S.; Escher, G.B.; Pereira, J.M.S.; Marinho, M.T.; Prado-Silva, L.; Sant’Ana, A.S.; Dutra, L.M.; Barison, A.; Granato, D. ^{1}H-NMR combined with chemometrics tools for rapid characterization of edible oils and their biological properties. *Ind. Crops Prod.* **2018**, *116*, 191-200.

I. SIMULATED AND EXPERIMENTAL SIGNALS

In this section, the simulated signals are compared with the experimental signals to confirm the assigned data.

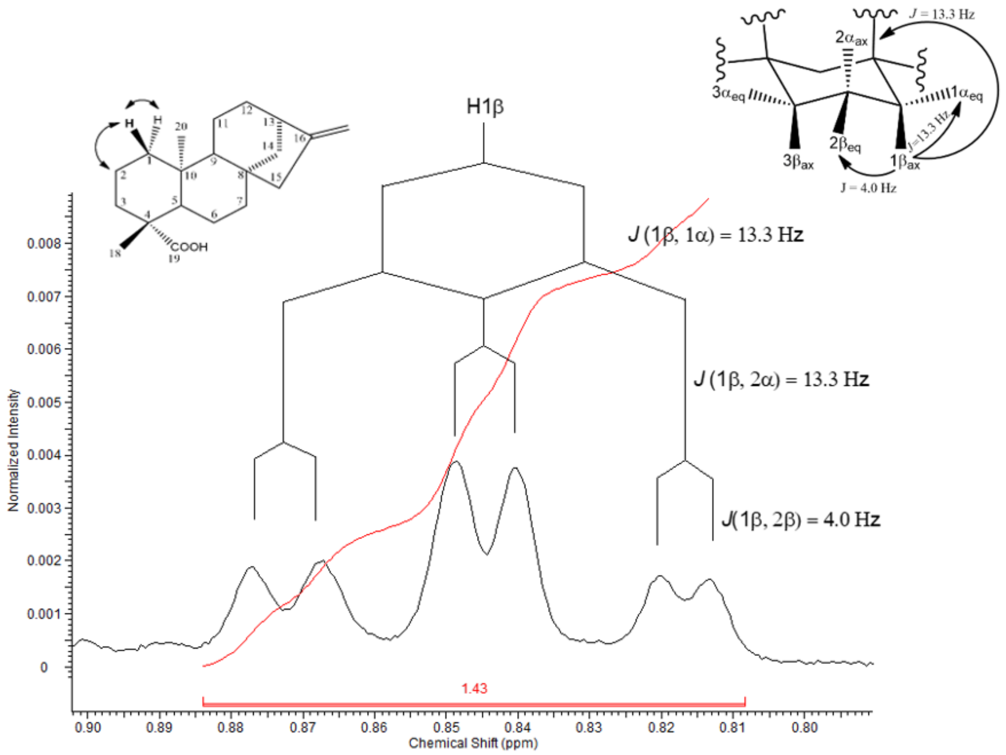

Figure S2. Signal of H1β in the ^{1}H NMR spectrum (500.13 MHz) of kaurenoic acid in CD_3OD.

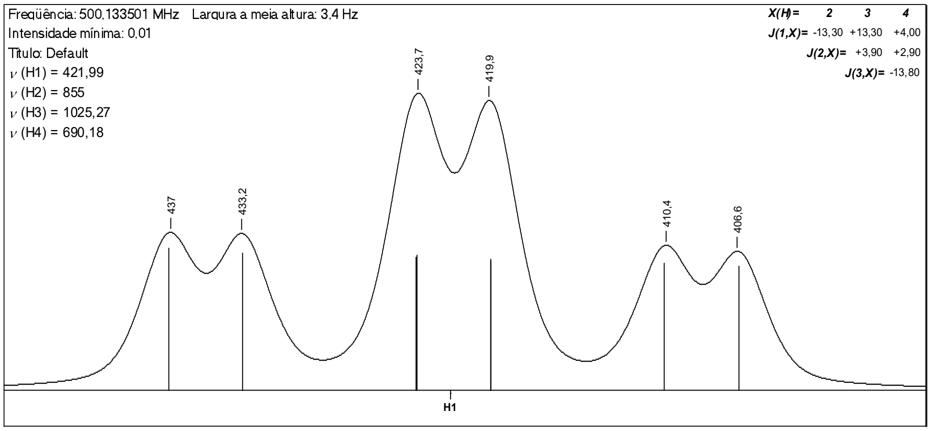

Figure S3. Dataset for the simulation of the H1β signal in the NMR_MultSim program.

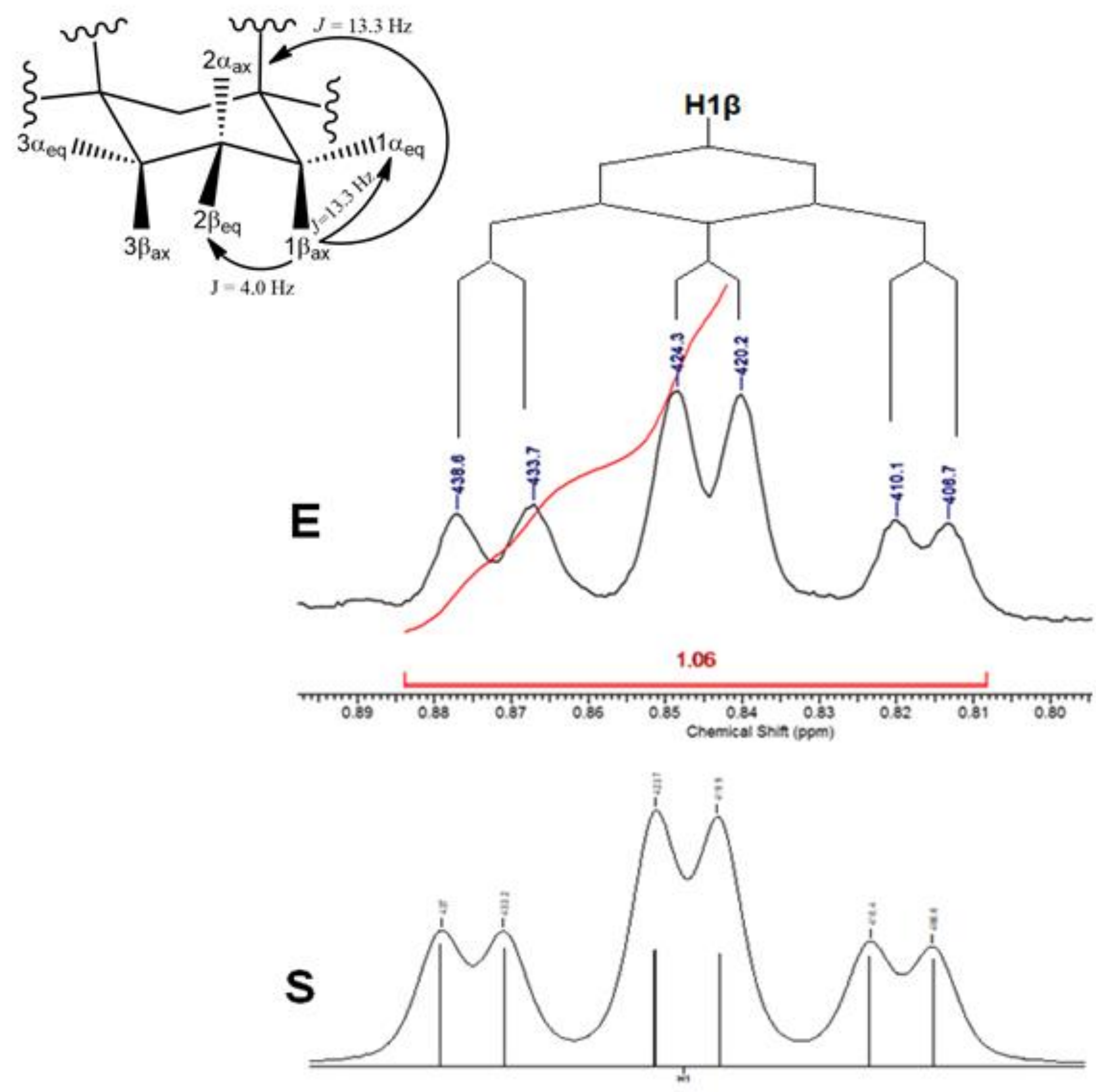

Figure S4. Experimental (**E**) and simulated (**S**) ^{1}H NMR signal H1β of *ent*-kaurenoic acid (CD_3OD).

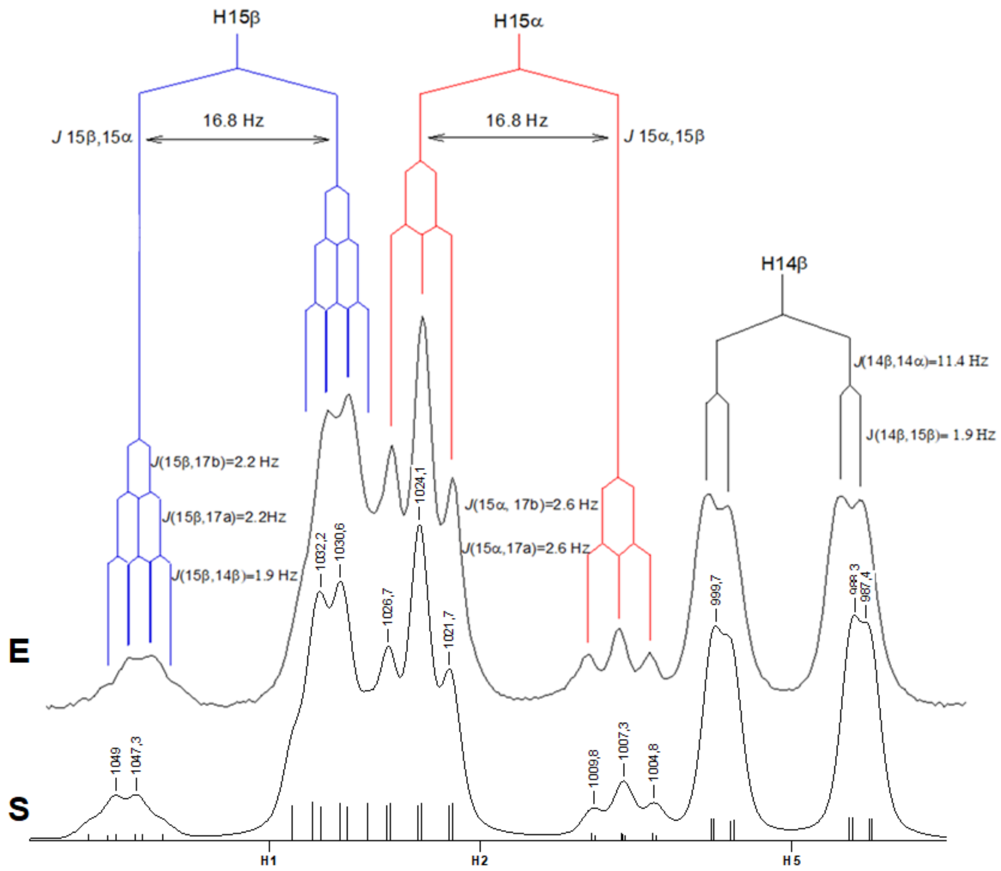

Figure S5. Experimental (**E**) and simulated (**S**) ^{1}H NMR signals H15β, H15α and H14β of *ent*-kaurenoic acid ($CDCl_3$).

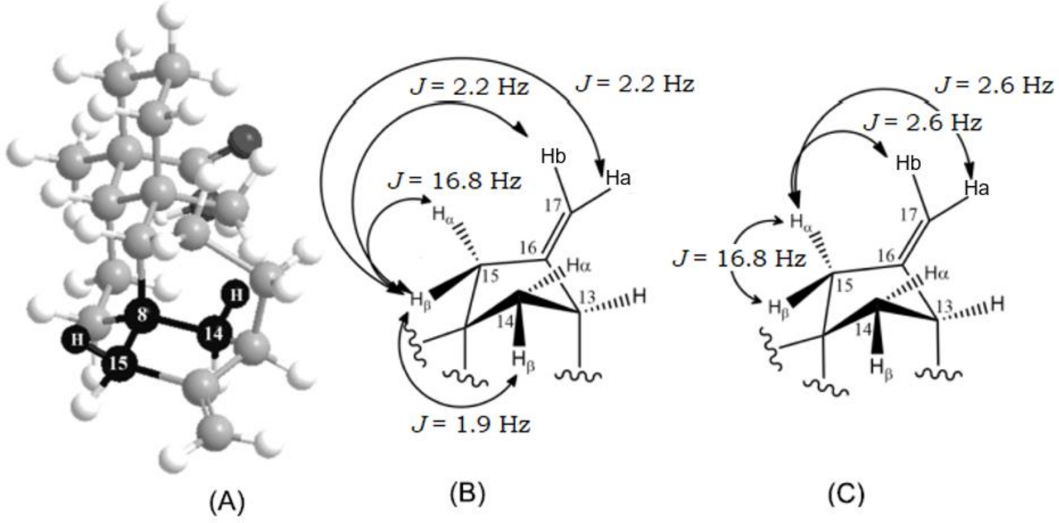

Figure S6. (A) 3D image demonstrating $^{4}J_{W}(15β,14β)$=1.9 Hz. (B) Spatial conformation with the correlations for H15β and (C) H15α.

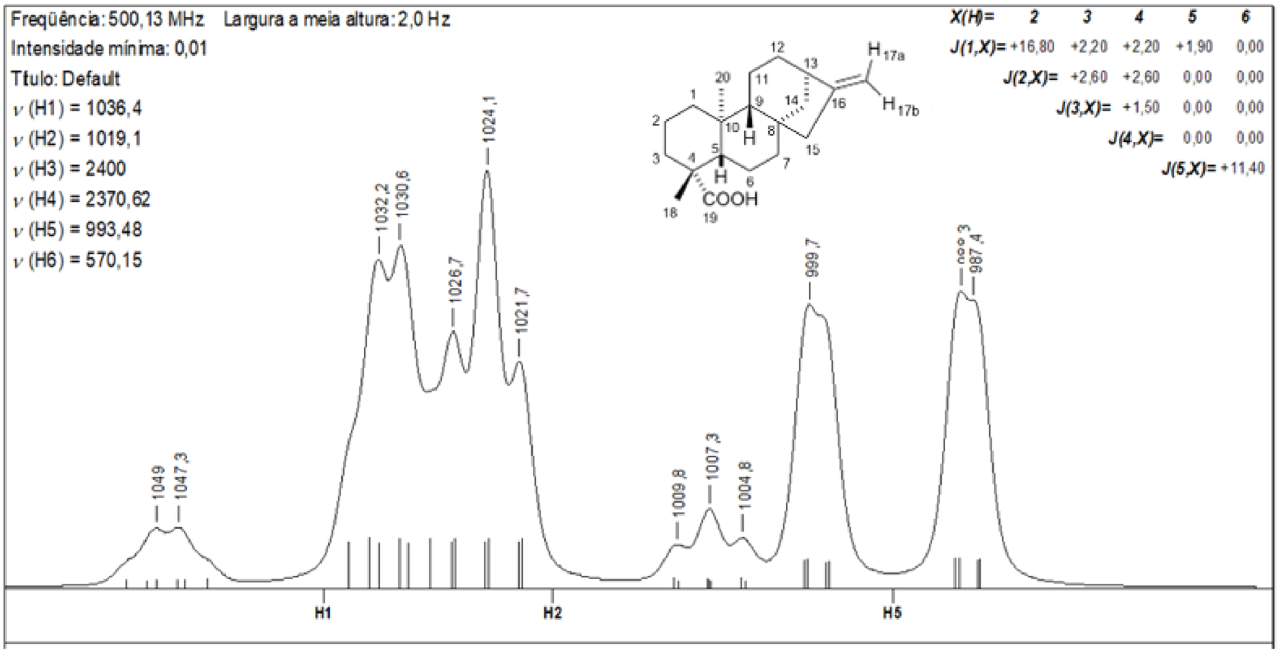

Figure S7. Dataset for the simulation of H15β, H15α, and H14β signals in the NMR_MultSim program.

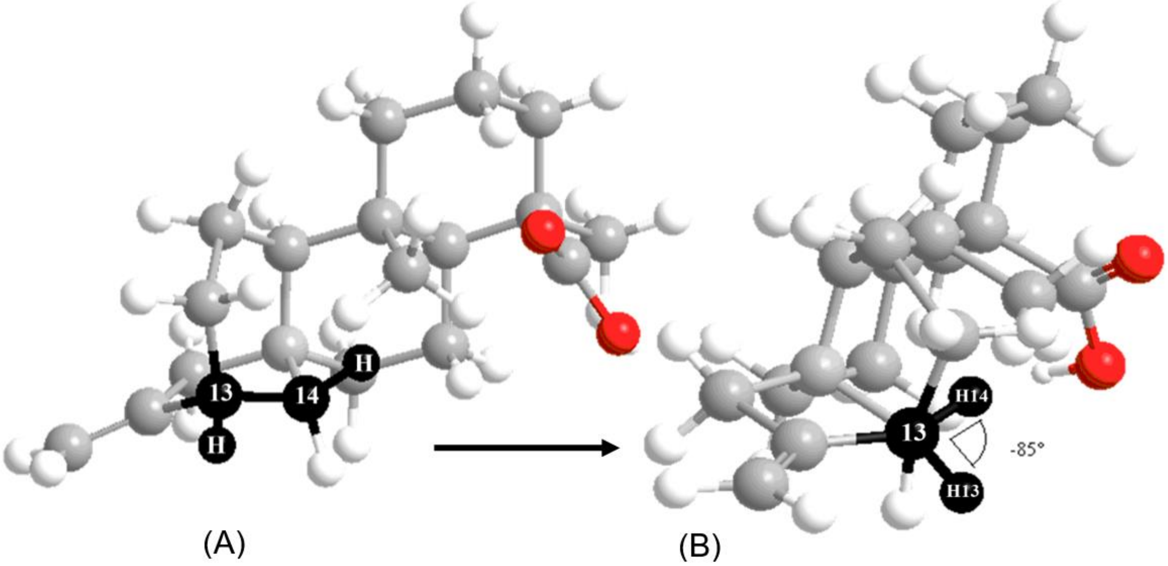

Figure S8. (A) 3D image demonstrating $^4J_W(15\beta,14\beta)$=1.9 Hz. (B) Spatial conformation with the correlations for H15β and (C) H15α.

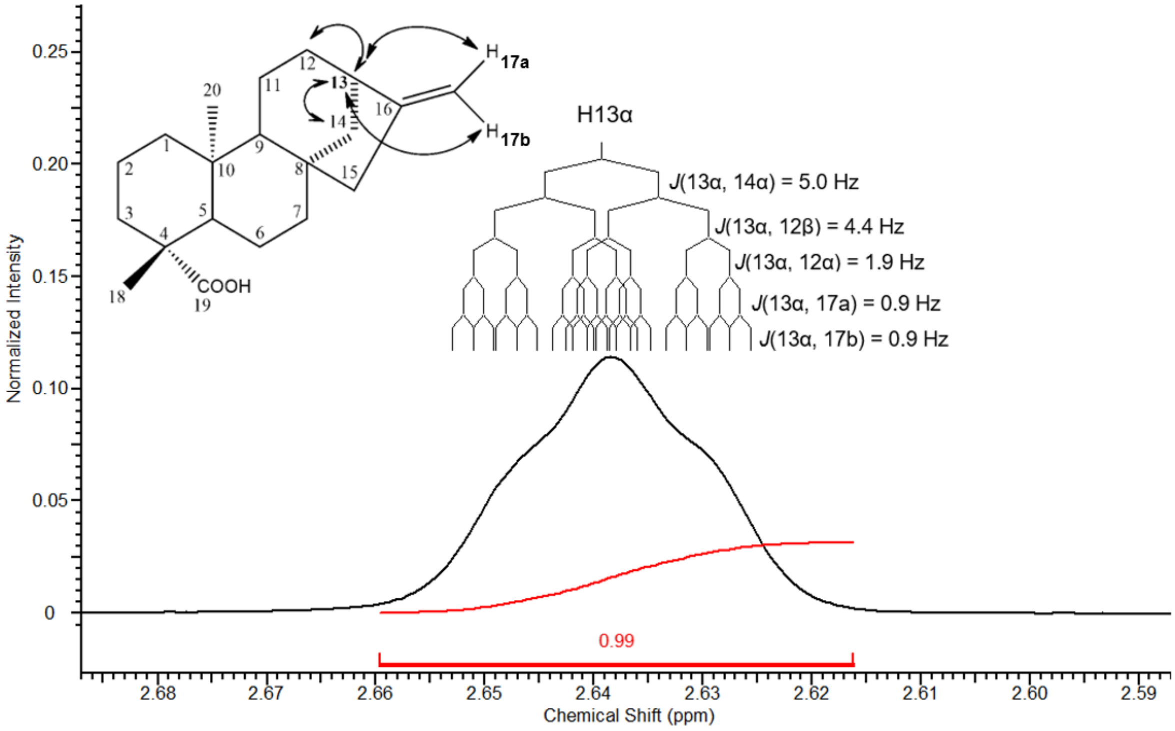

Figure S9. Signal of H13α in the ^{1}H NMR spectrum (500.13 MHz) of kaurenoic acid in $CDCl_3$.

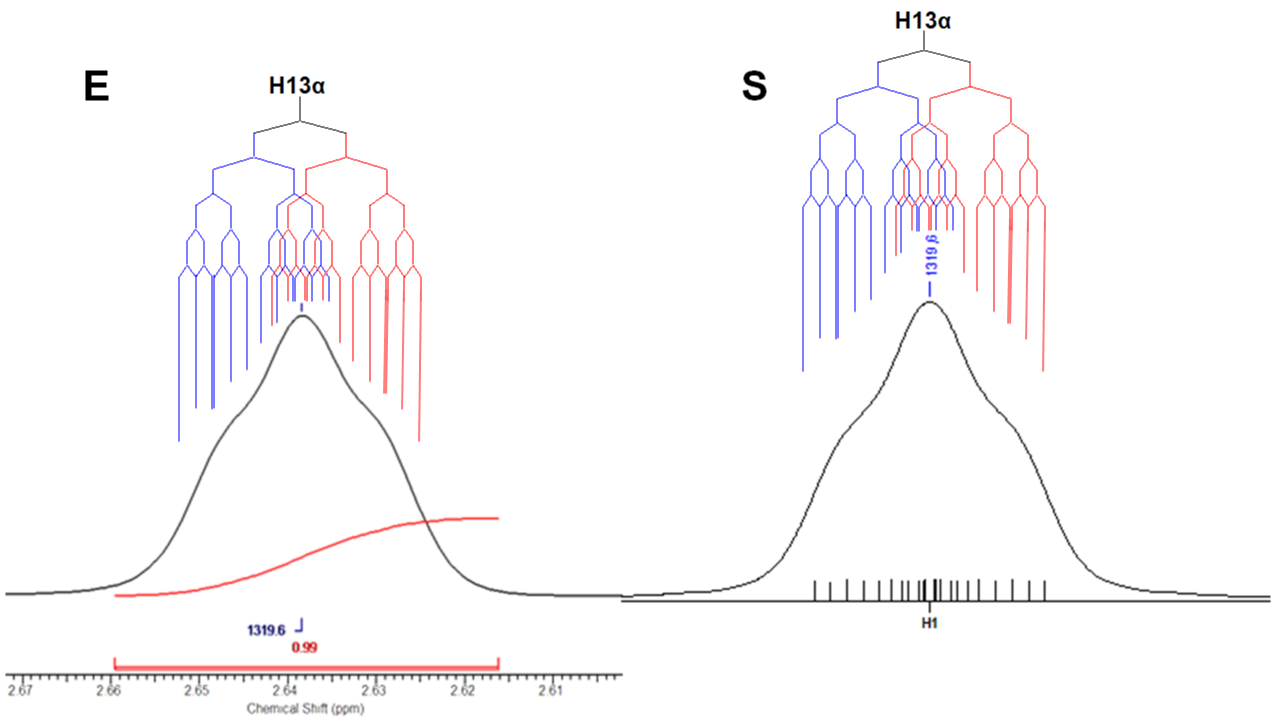

Figure S10. Experimental (**E**) and simulated (**S**) ^{1}H NMR signal H13α of *ent*-kaurenoic acid ($CDCl_3$).

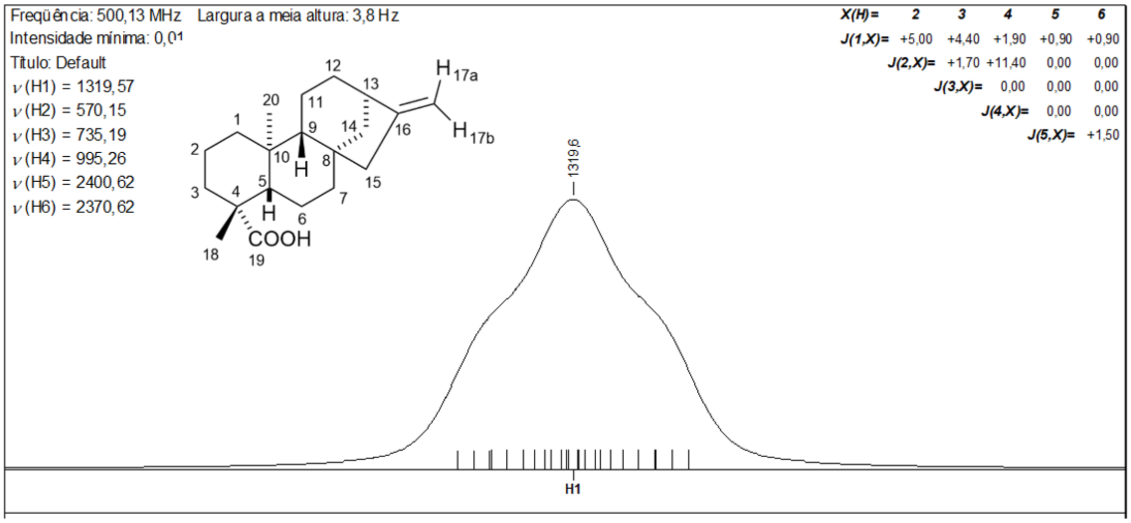

Figure S11. Dataset for the simulation of the H13α signal in the NMR_MultSim program.

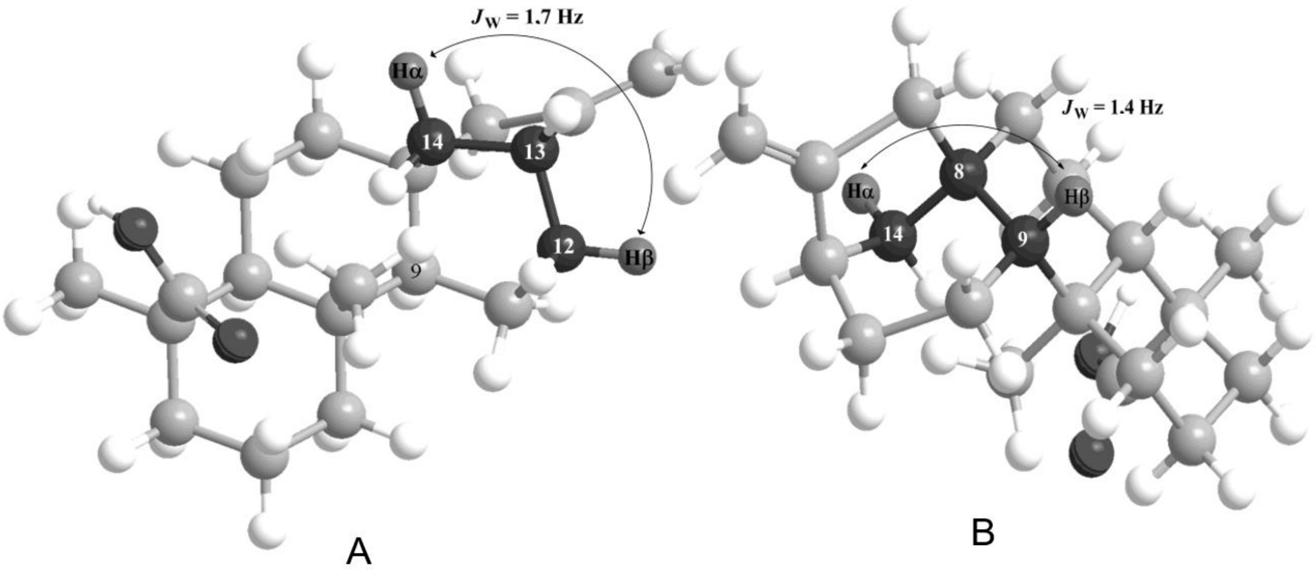

Figure S12. (A) 3D image demonstrating $^4J_W(14\alpha,12\beta)$=1.7 Hz and (B) $^4J_W(14\alpha,9\beta)$=1.4.

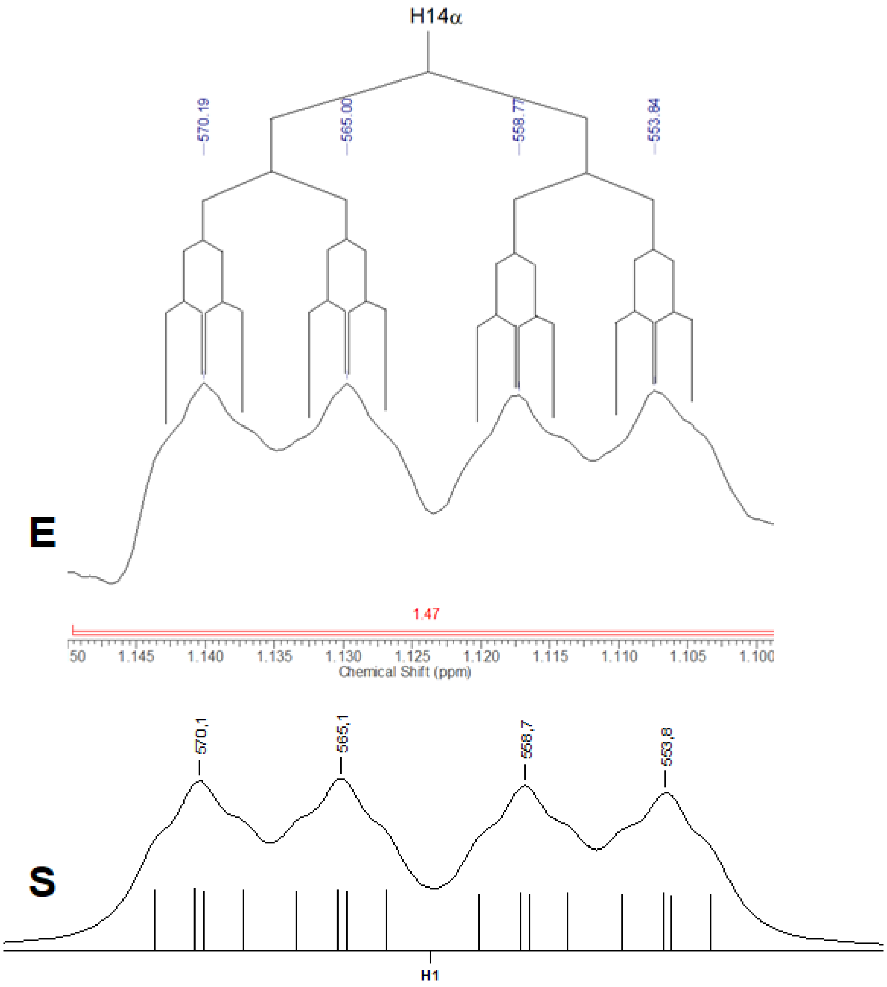

Figure S13. Experimental (**E**) and simulated (**S**) ^{1}H NMR signals H14α of *ent*-kaurenoic acid (CD_3OD).

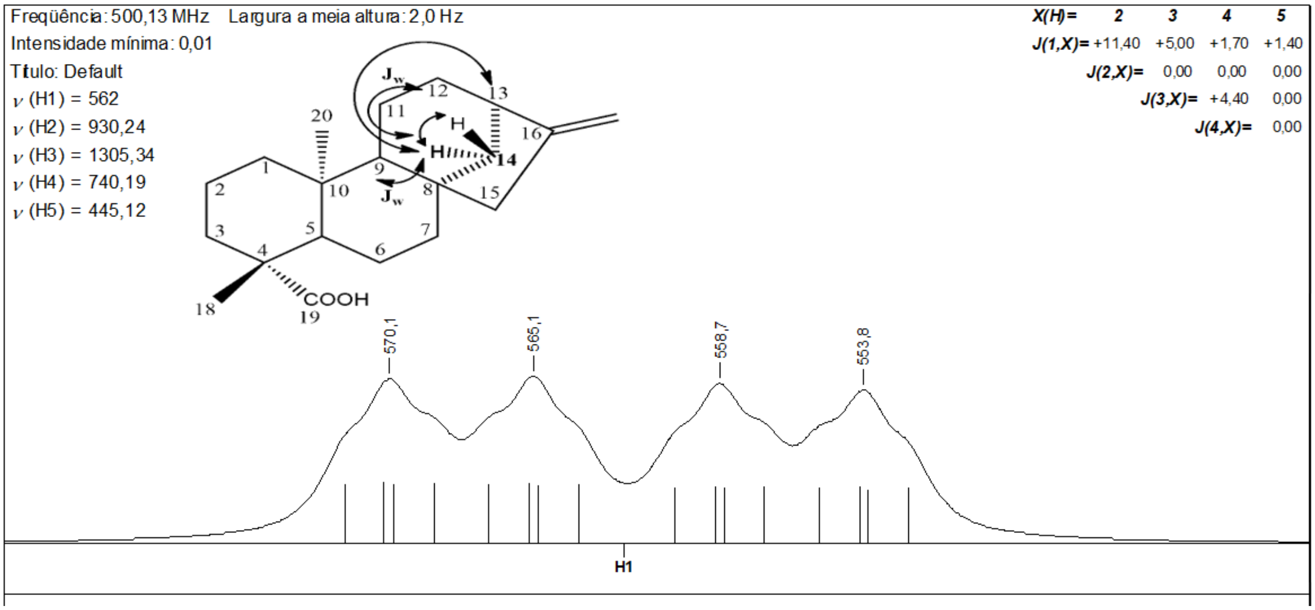

Figure S14. Dataset for the simulation of the H14α signal in the NMR_MultSim program.

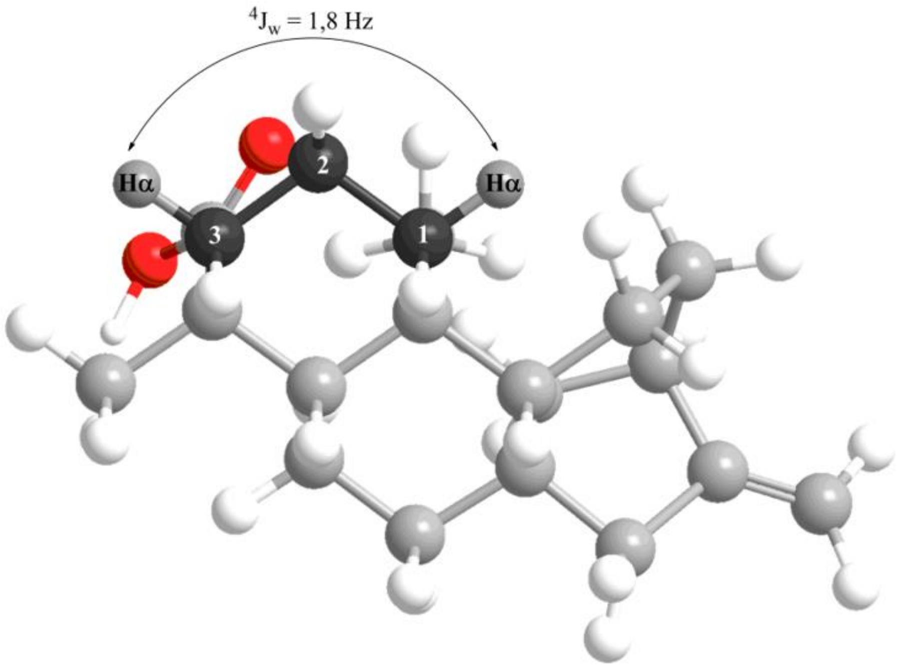

Figure S15. 3D image demonstrating $^4J_w(3\alpha,1\alpha)$ = 1.8 Hz.

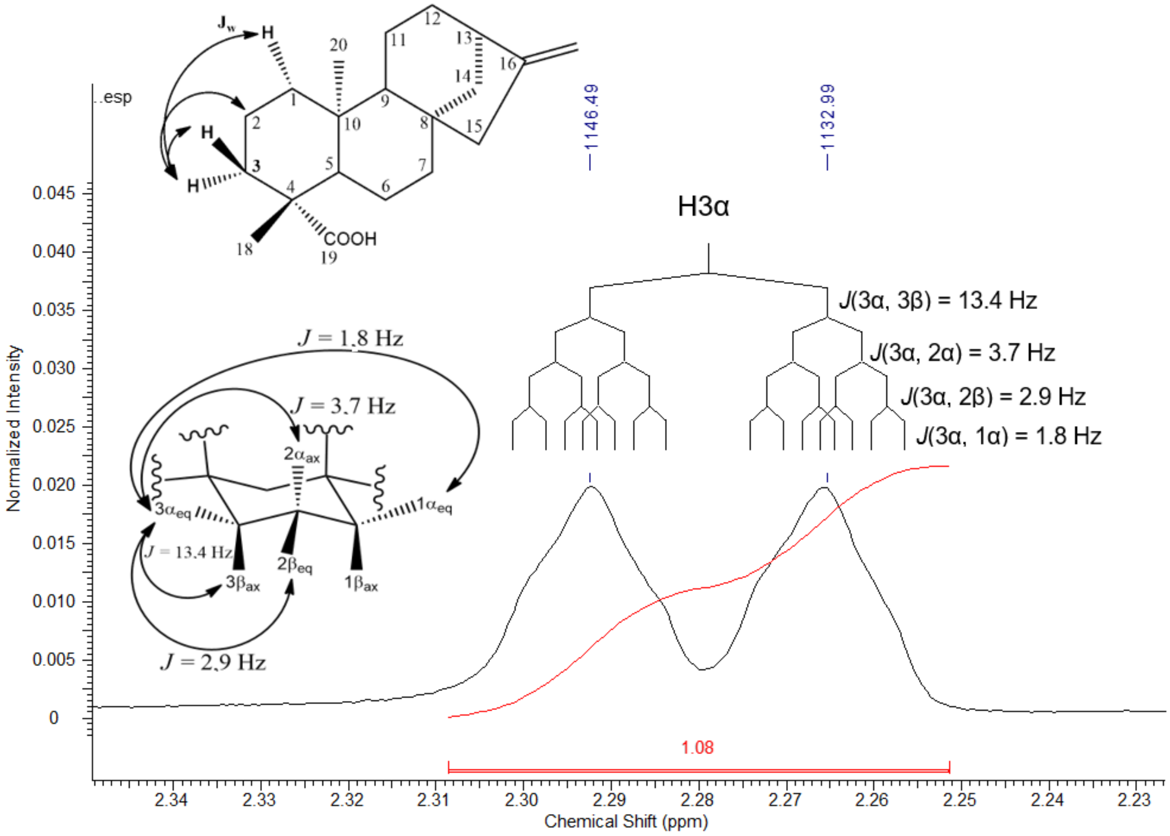

Figure S16. Signal of H3α in the ^{1}H NMR spectrum (500.13 MHz) of kaurenoic acid in C_6D_6.

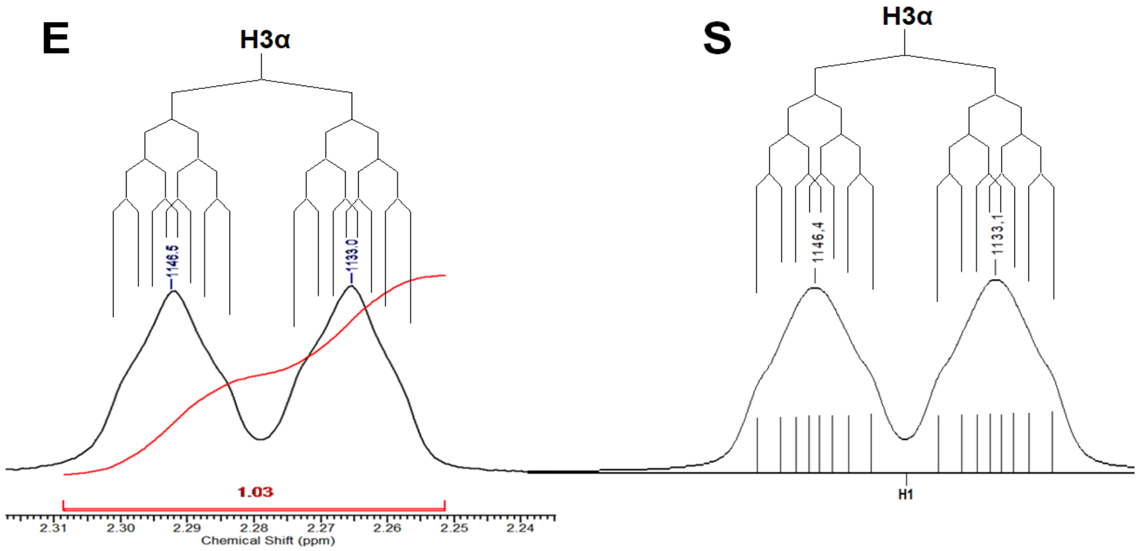

Figure S17. Experimental (**E**) and simulated (**S**) ^{1}H NMR signal H3α of *ent*-kaurenoic acid (C_6D_6).

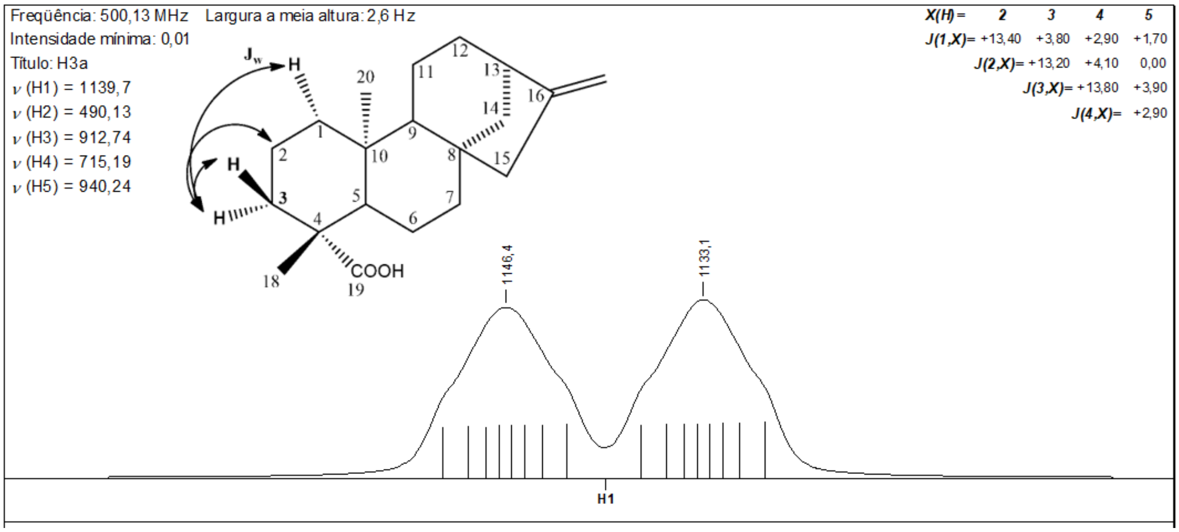

Figure S18. Dataset for the simulation of the H3α signal in the NMR_MultSim program.

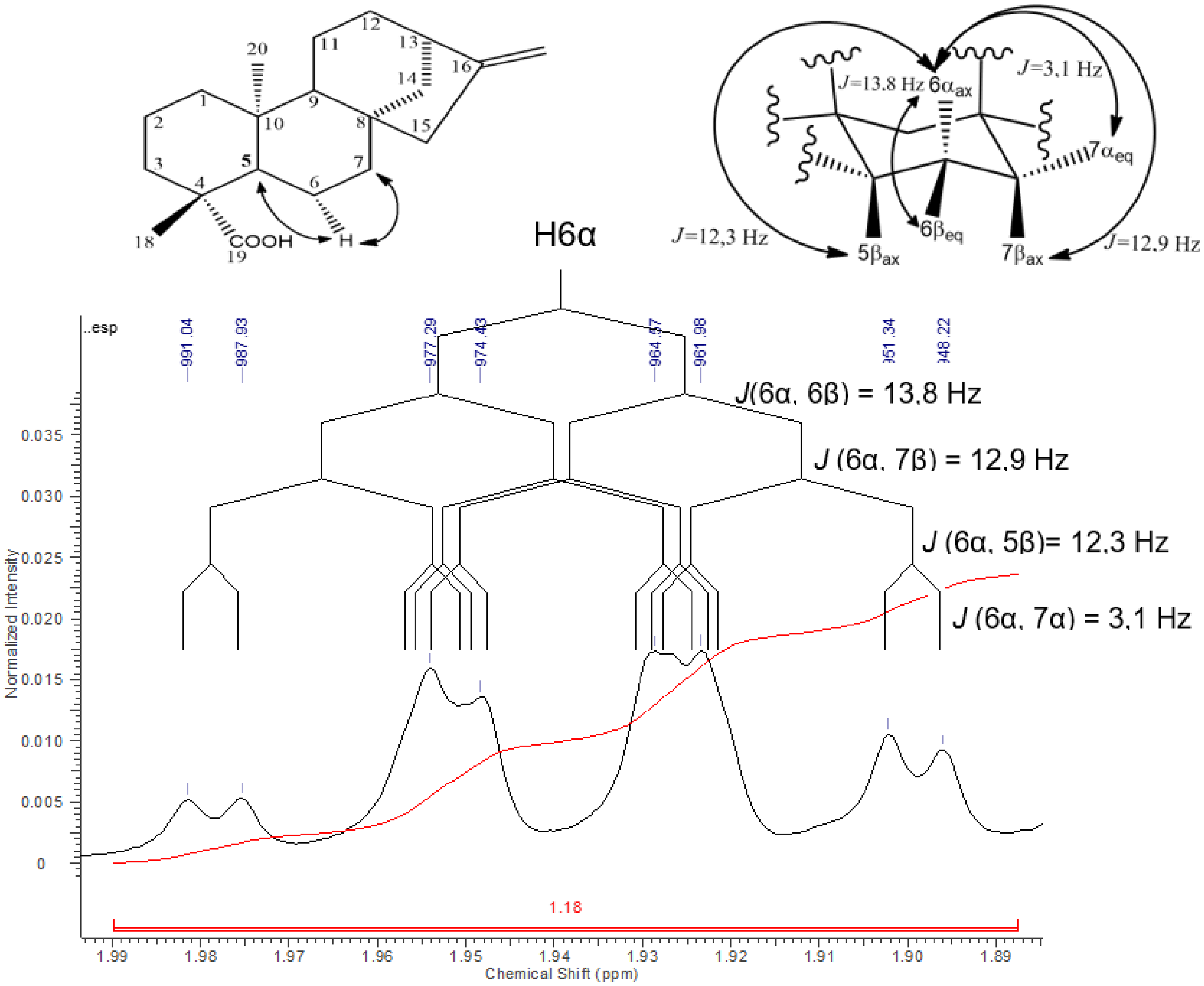

Figure S19. Signal of H6α in the ^{1}H NMR spectrum (500.13 MHz) of kaurenoic acid in C_6D_6.

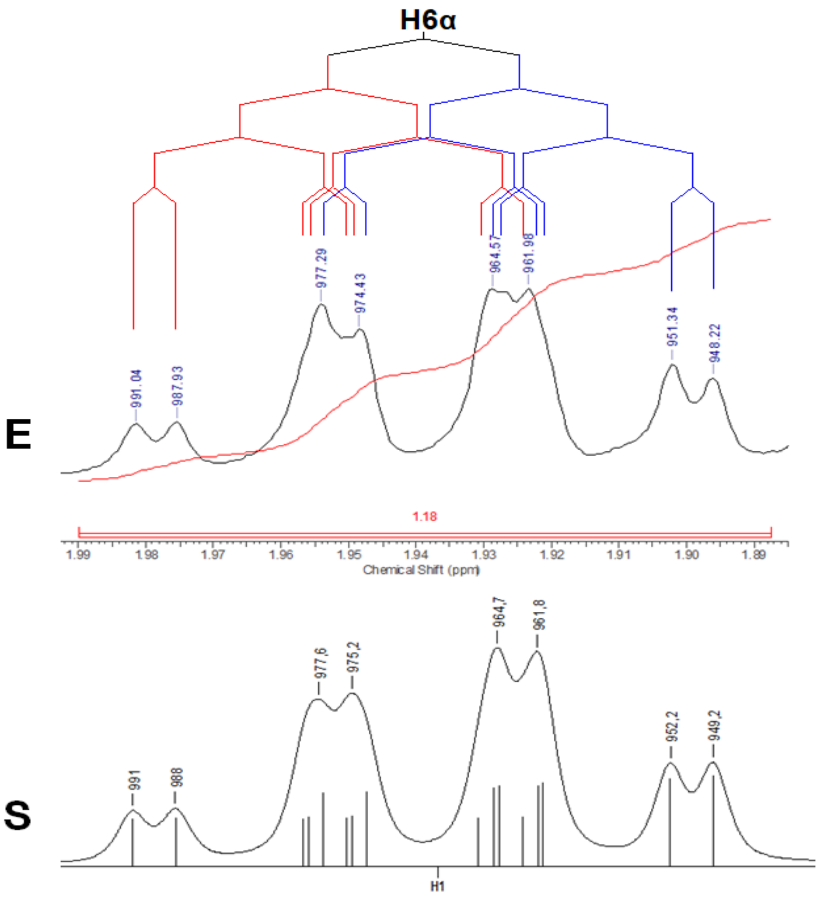

Figure S20. Experimental (**E**) and simulated (**S**) ^{1}H NMR signal H6α of *ent*-kaurenoic acid (C_6D_6).

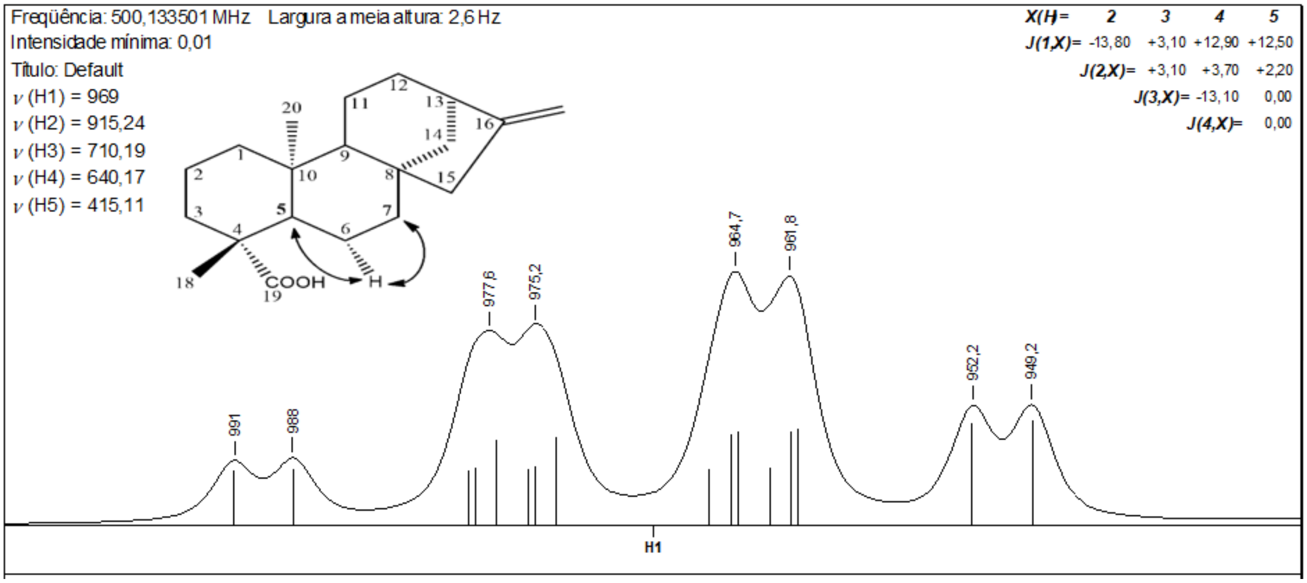

Figure S21. Dataset for the simulation of the H6α signal in the NMR_MultSim program.

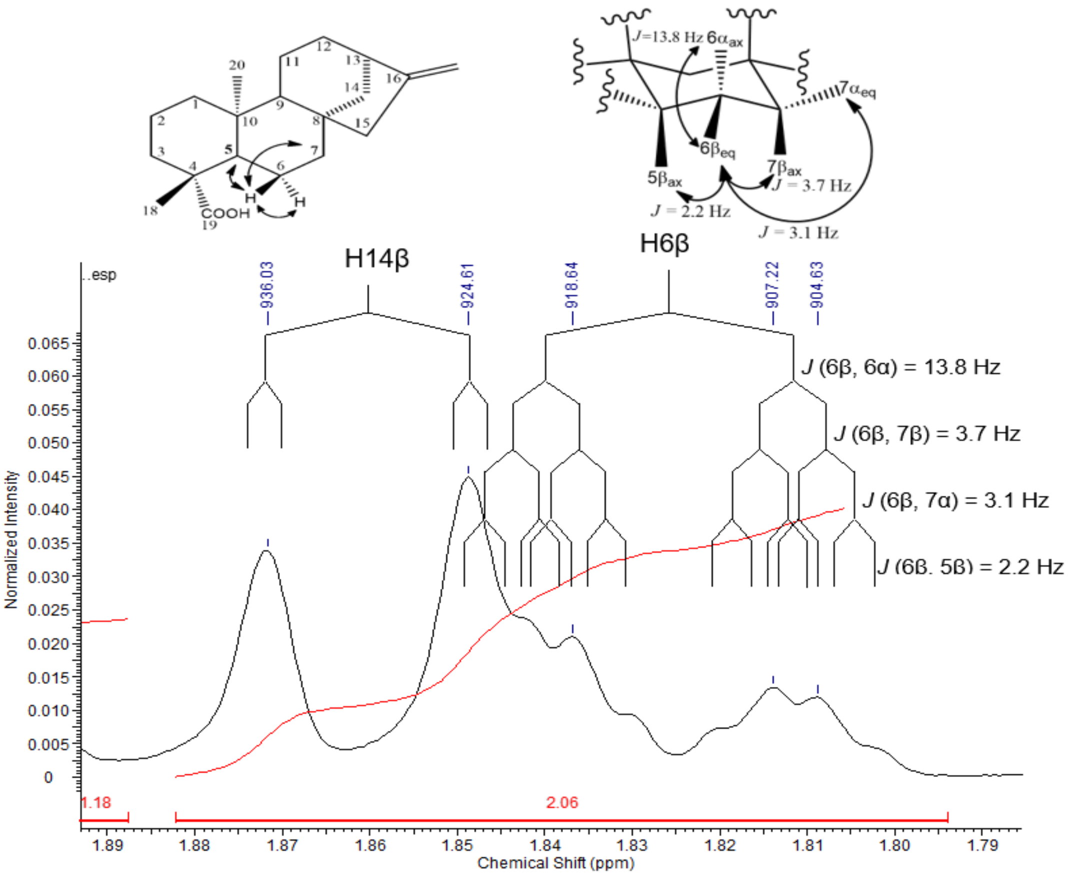

Figure S22. Signal of H14β and H6β in the ^{1}H NMR spectrum (500.13 MHz) of kaurenoic acid in C_6D_6.

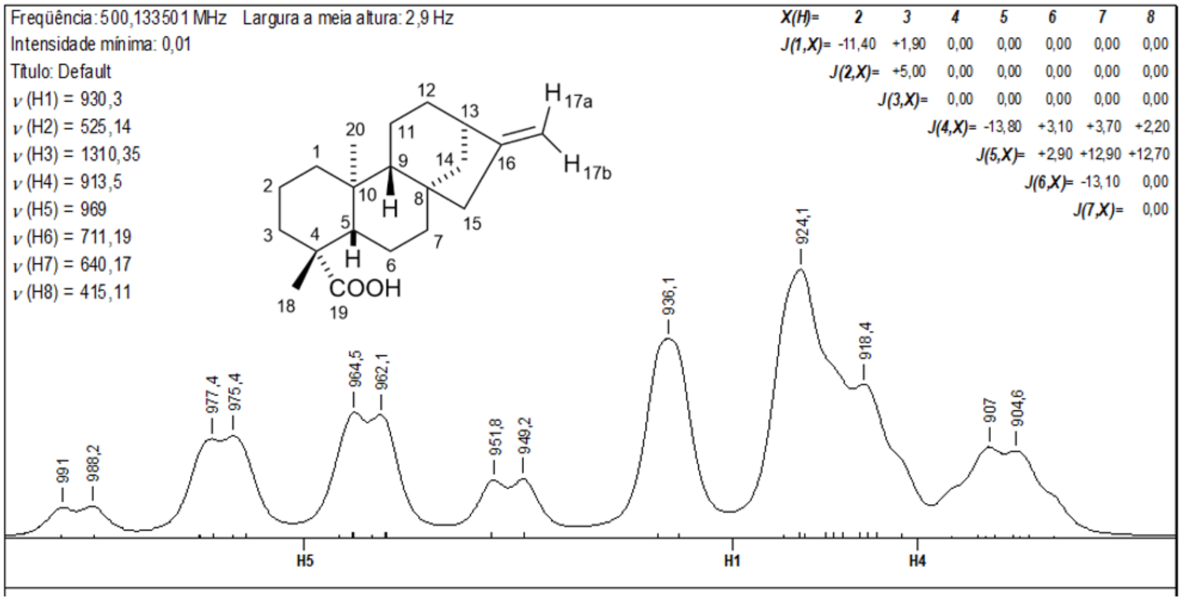

Figure S23. Dataset for the simulation of the H6α, H14β, and H6β signals in the NMR_MultSim program.

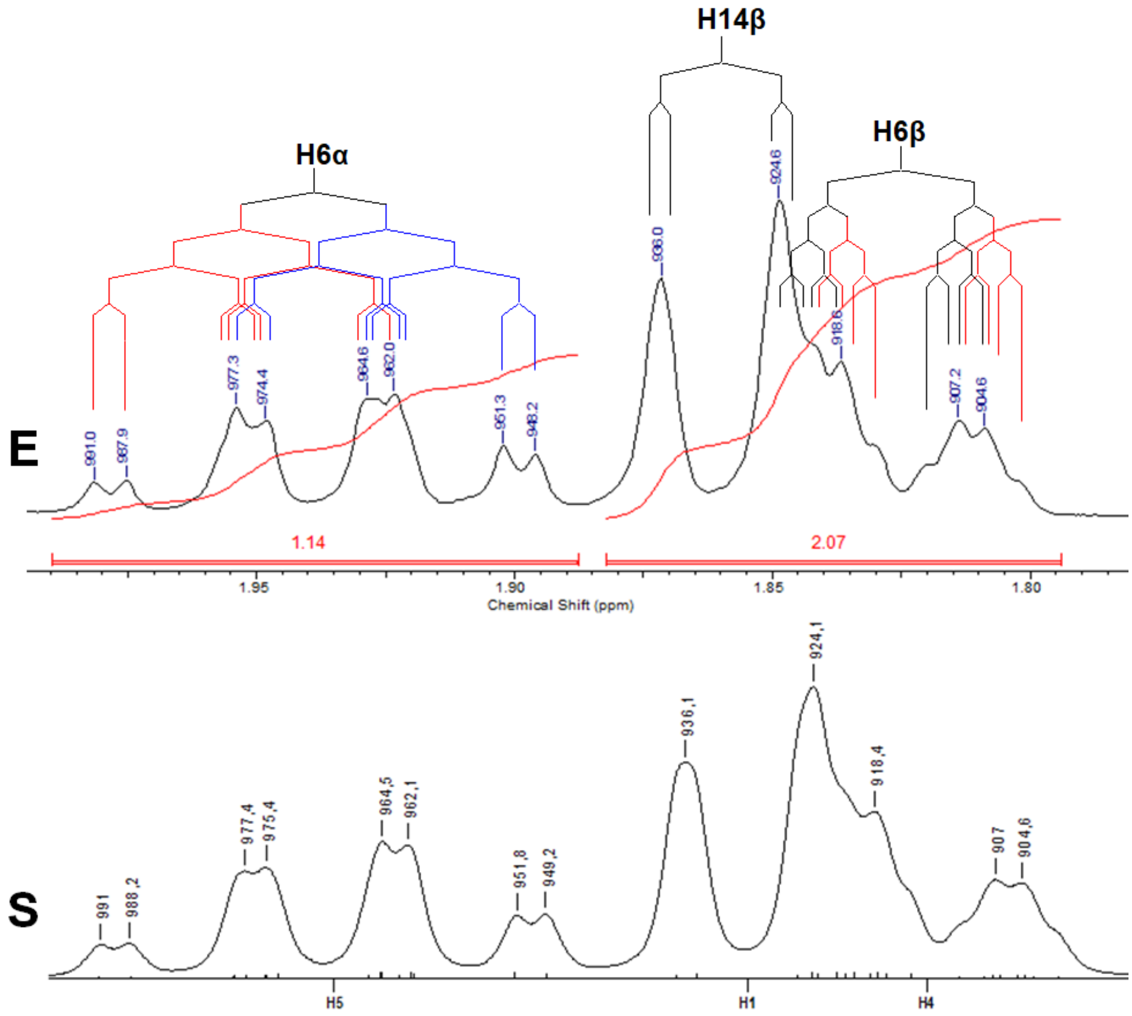

Figure S24. Experimental (**E**) and simulated (**S**) ^{1}H NMR signals H6α, H14β and H6β of *ent*-kaurenoic acid (C_6D_6).

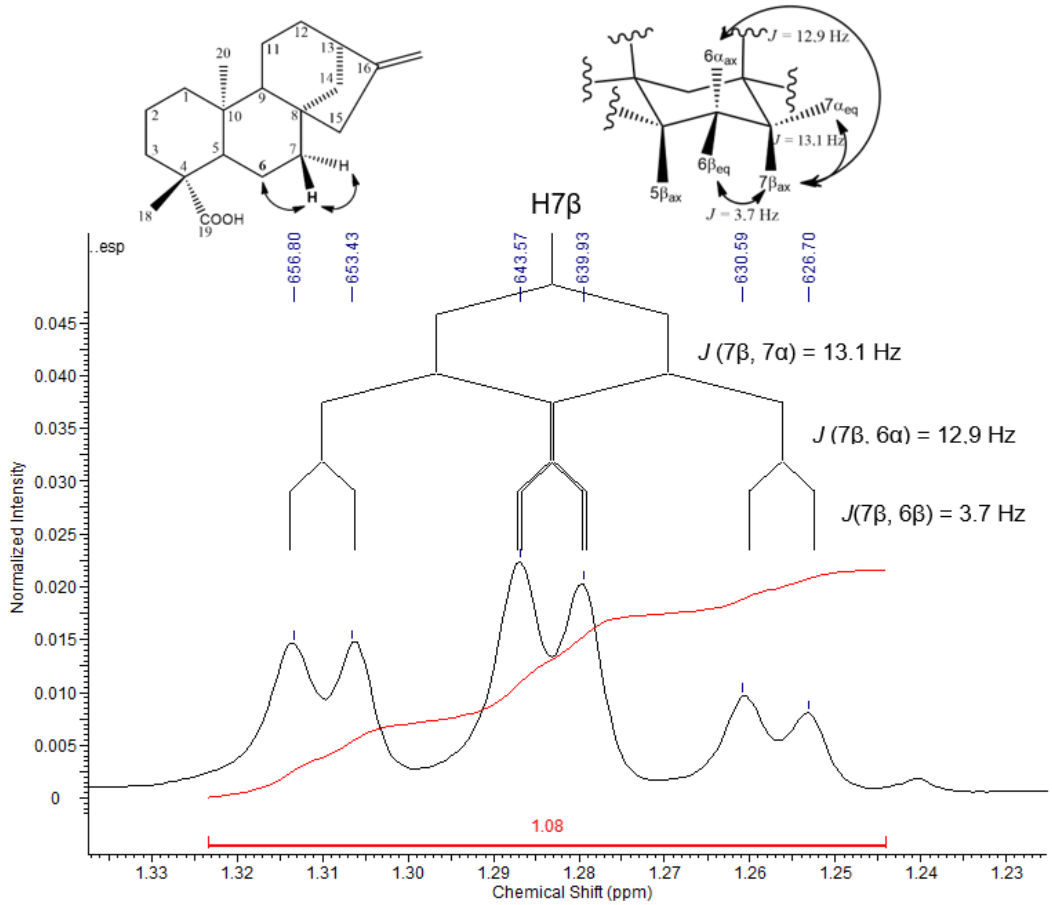

Figure S25. Signal of H7β in the ^{1}H NMR spectrum (500.13 MHz) of kaurenoic acid in C_6D_6.

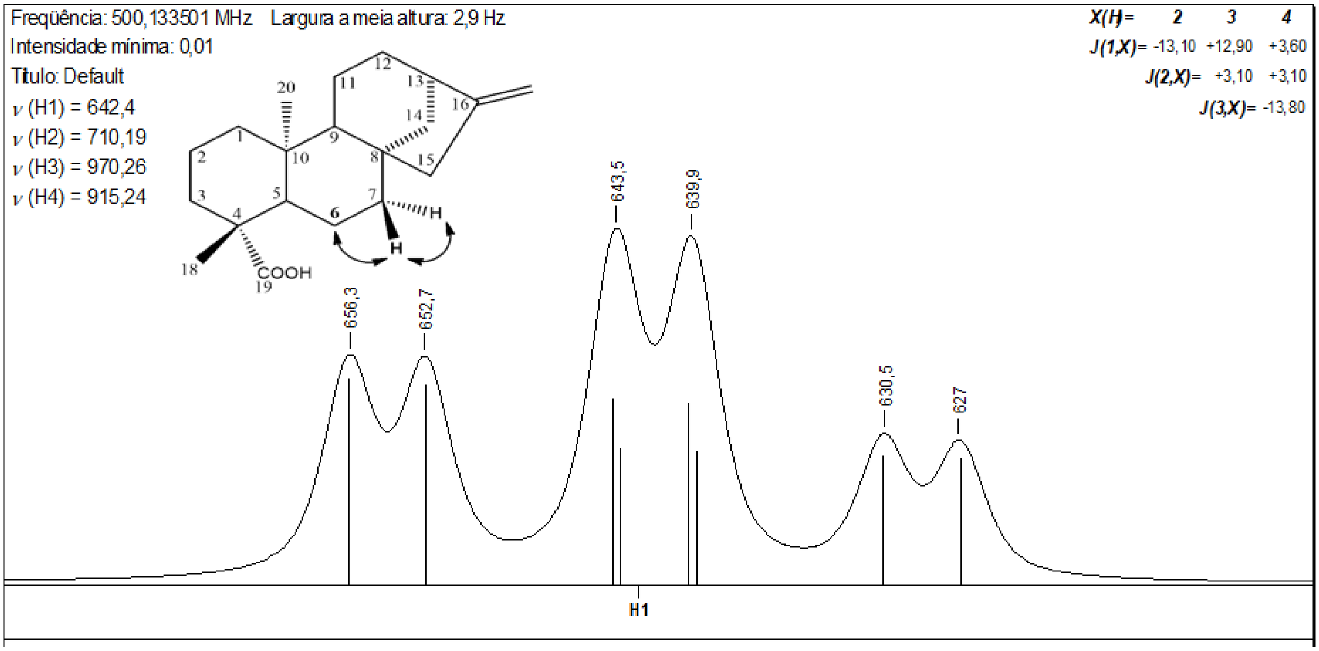

Figure S26. Dataset for the simulation of the H7β signal in the NMR_MultSim program.

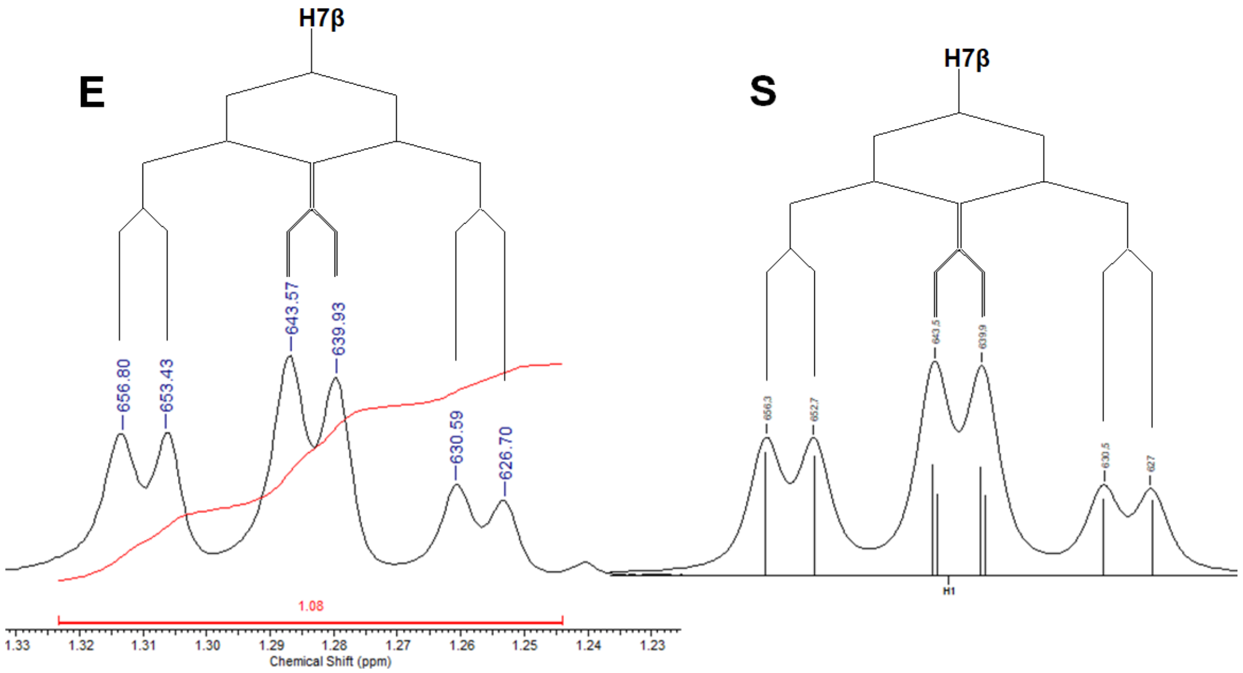

Figure S27. Experimental (**E**) and simulated (**S**) ^{1}H NMR signal H7β of *ent*-kaurenoic acid (C_6D_6).

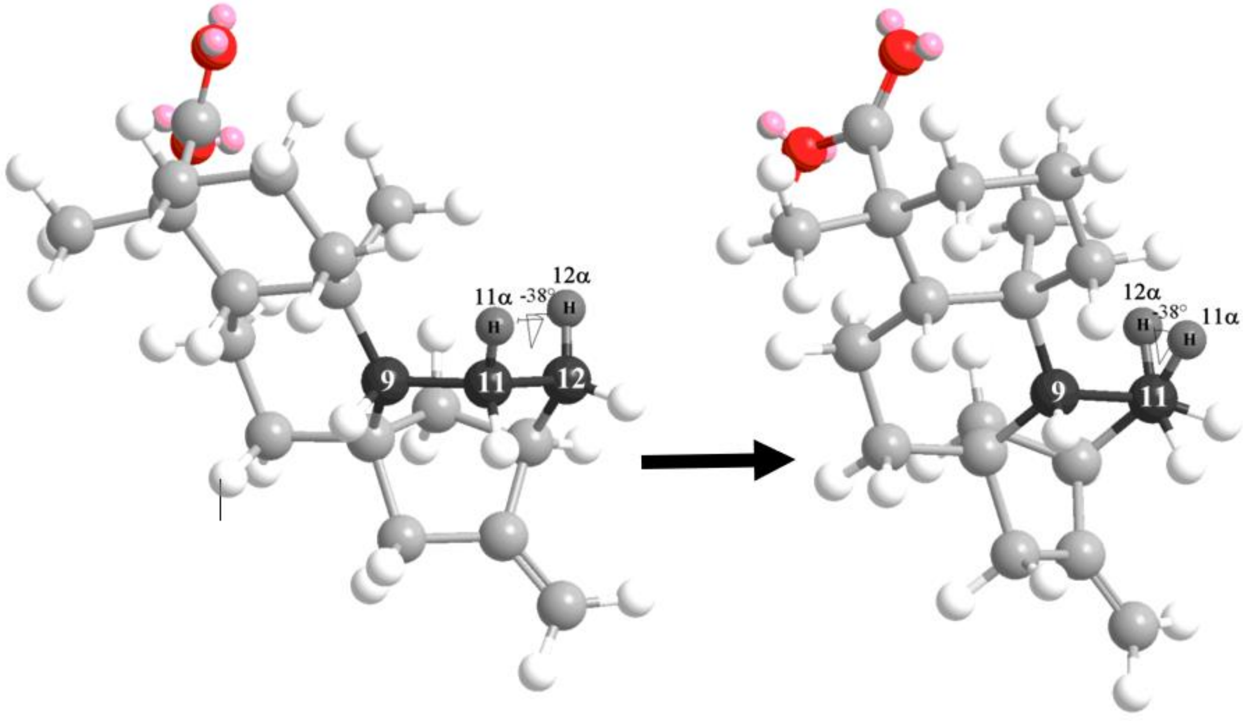

Figure S28. 3D image representing the possible dihedral angle (38º) between H11α and H12α.

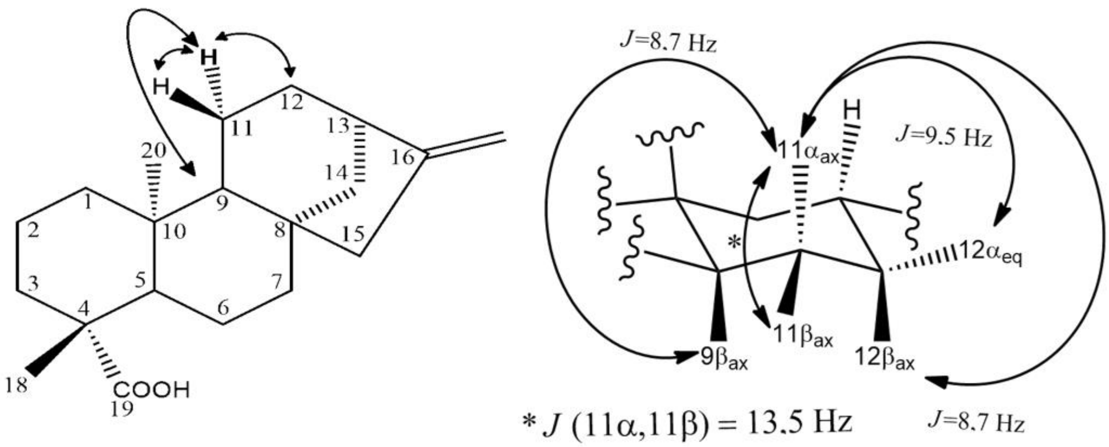

Figure S29. Spatial conformation of the ring region of the kaurenoic acid structure showing possible correlations for H11α.

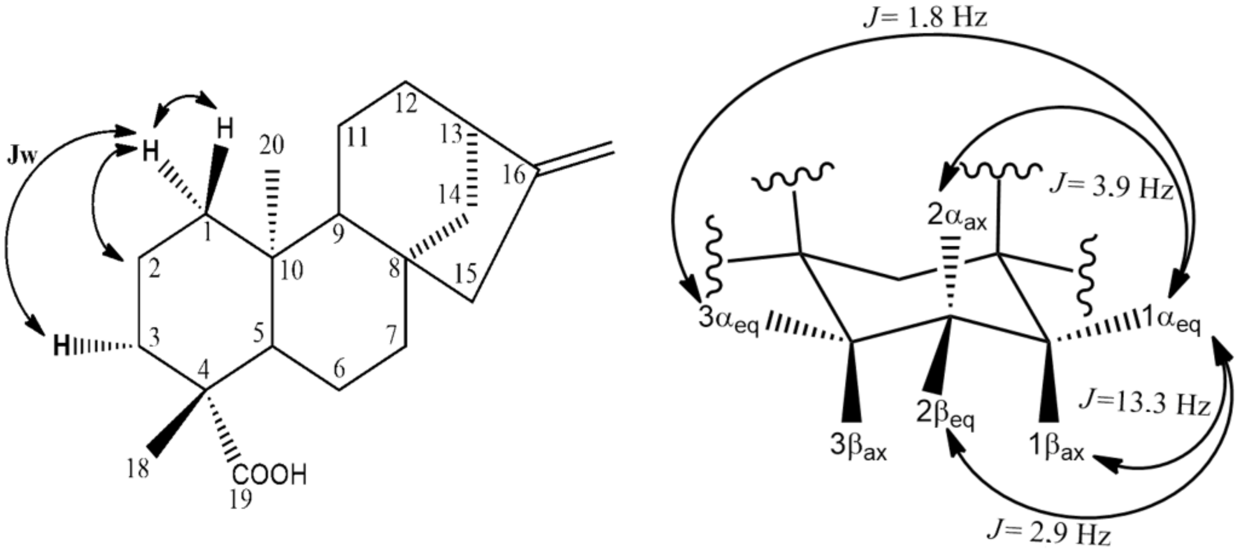

Figure S30. Spatial conformation of the ring region of the kaurenoic acid structure showing possible correlations for H1α.

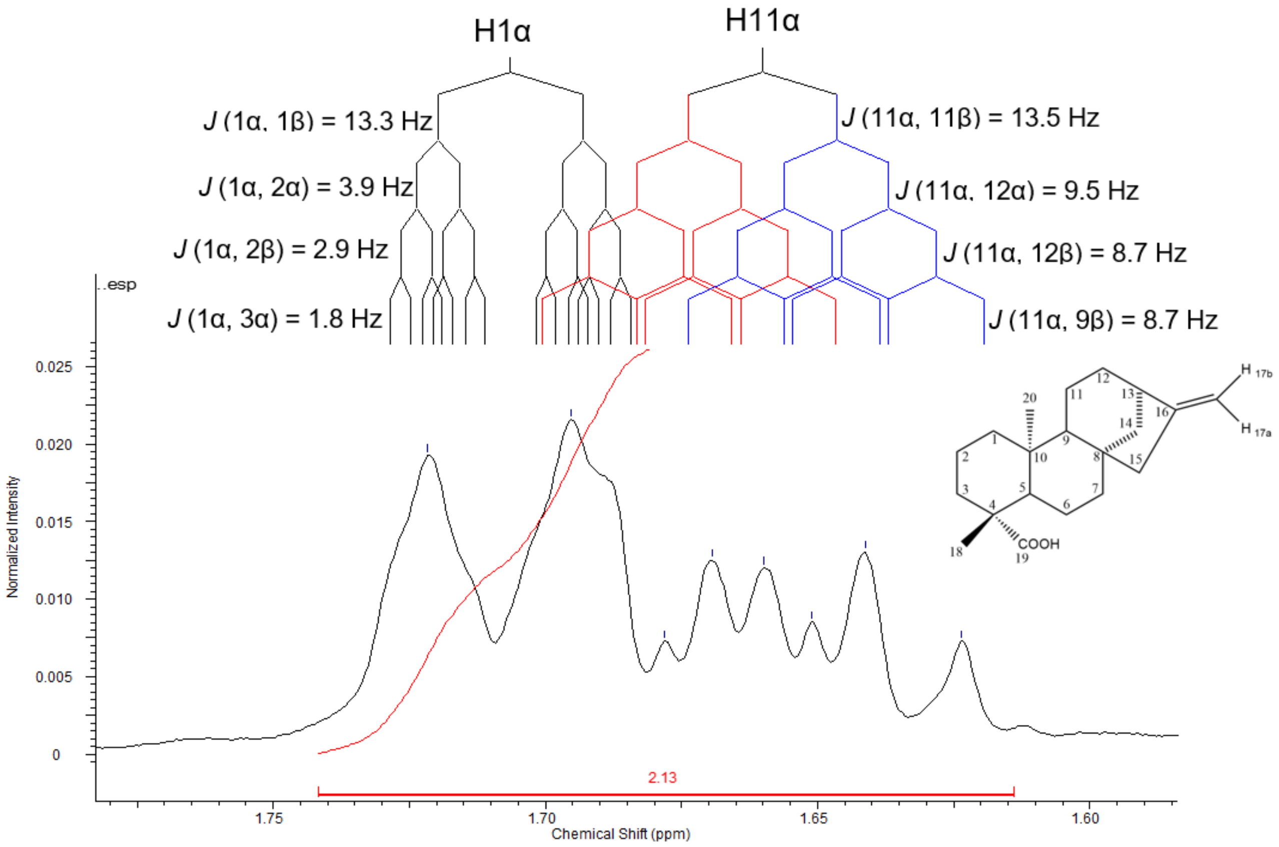

Figure S31. Signal of H1α and H11α in the ^{1}H NMR spectrum (500.13 MHz) of kaurenoic acid in C_6D_6.

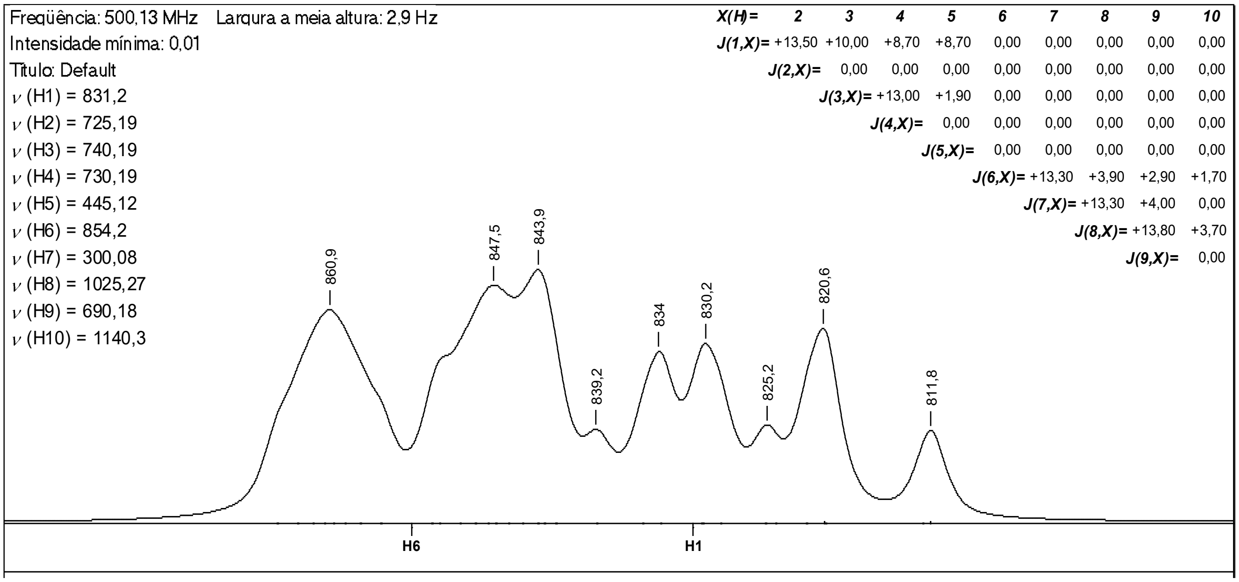

Figure S32. Dataset for the simulation of the H1α and H11α signals in the NMR_MultSim program.

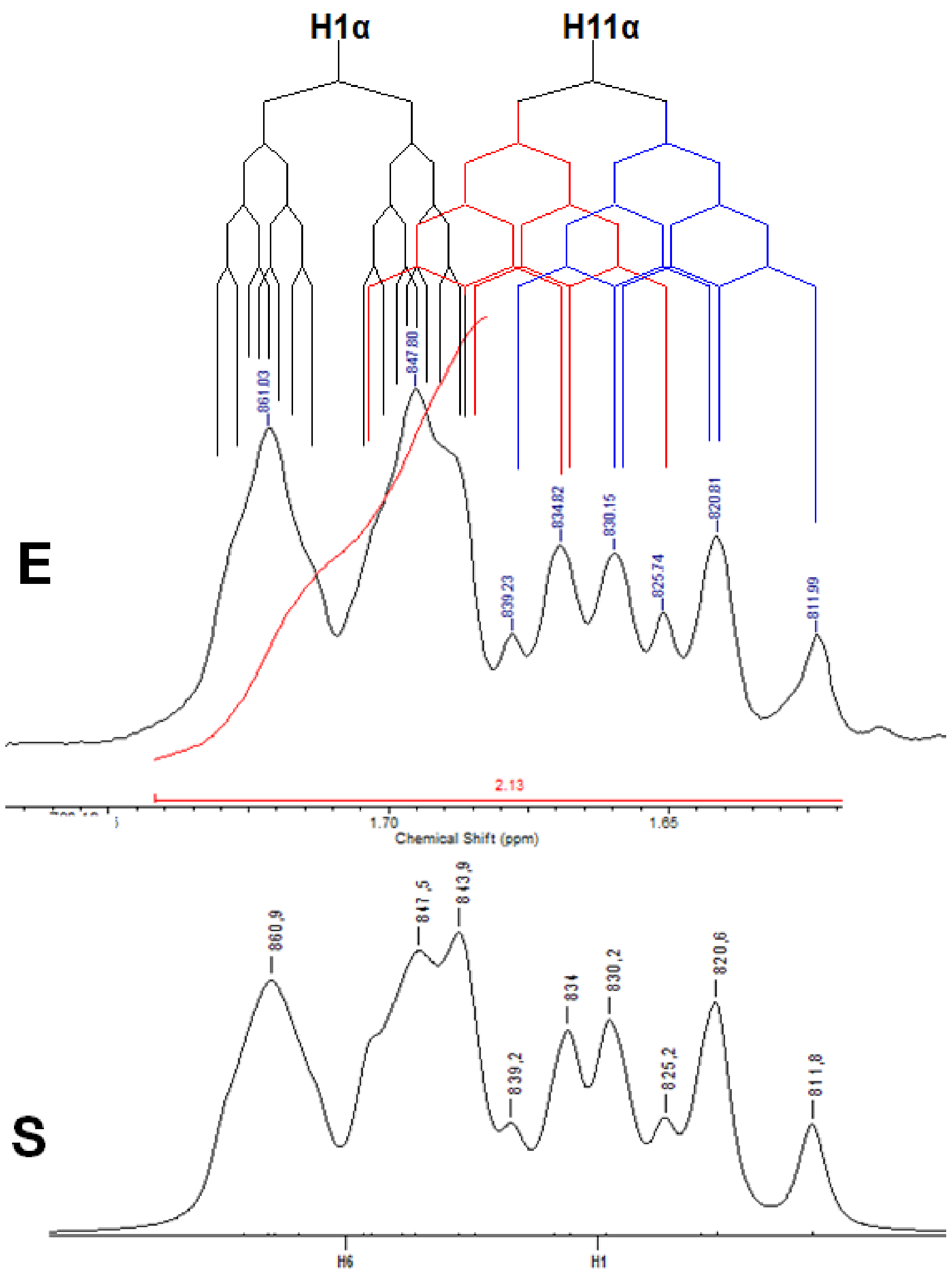

Figure S33. Experimental (**E**) and simulated (**S**) ^{1}H NMR signals H1α and H11α of *ent*-kaurenoic acid (C_6D_6).

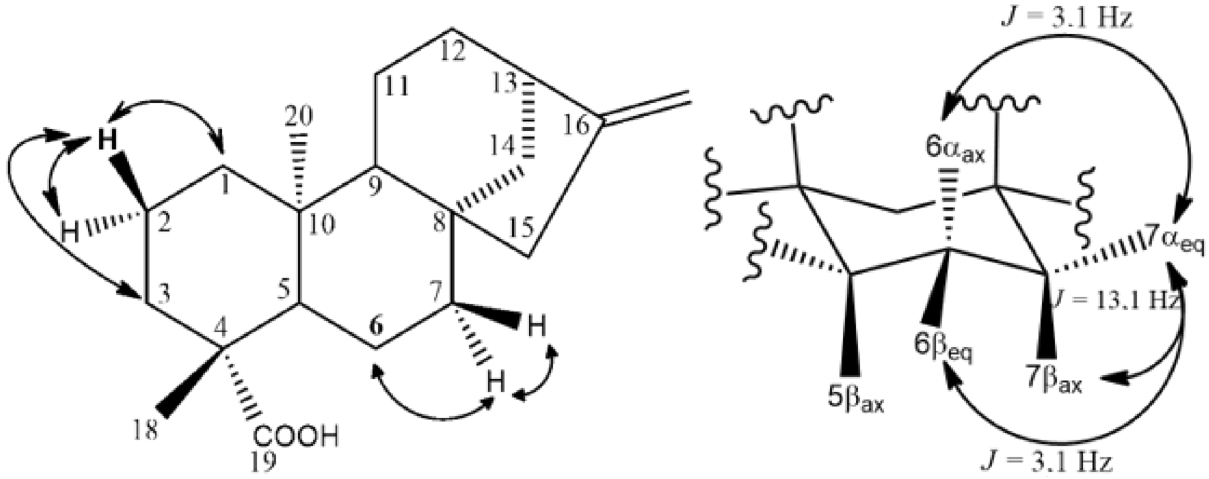

Figure S34. Spatial conformation of the ring region of the kaurenoic acid structure showing possible correlations for H7α.

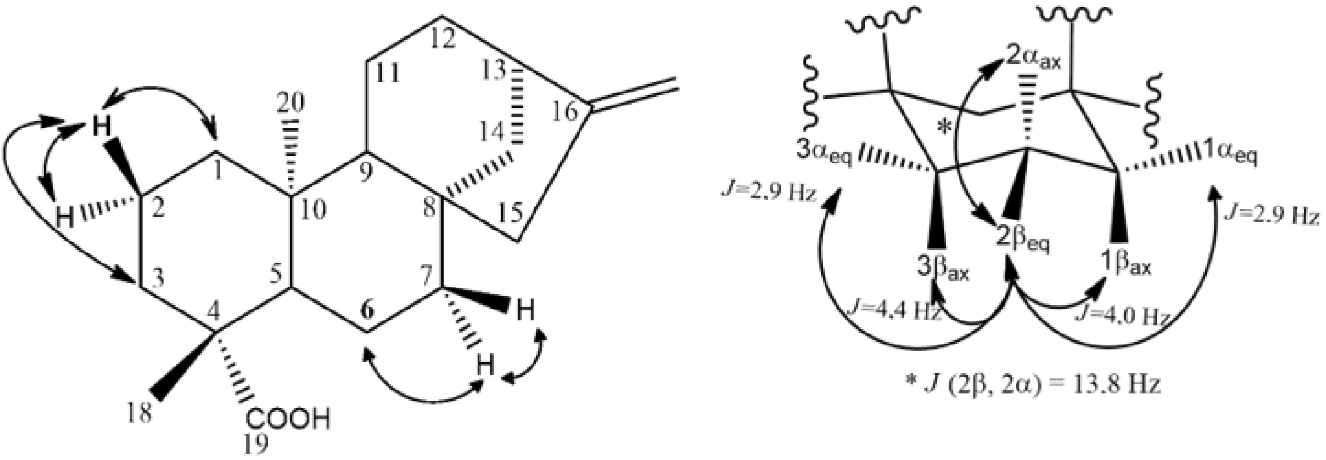

Figure S35. Spatial conformation of the ring region of the kaurenoic acid structure showing possible correlations for H2β.

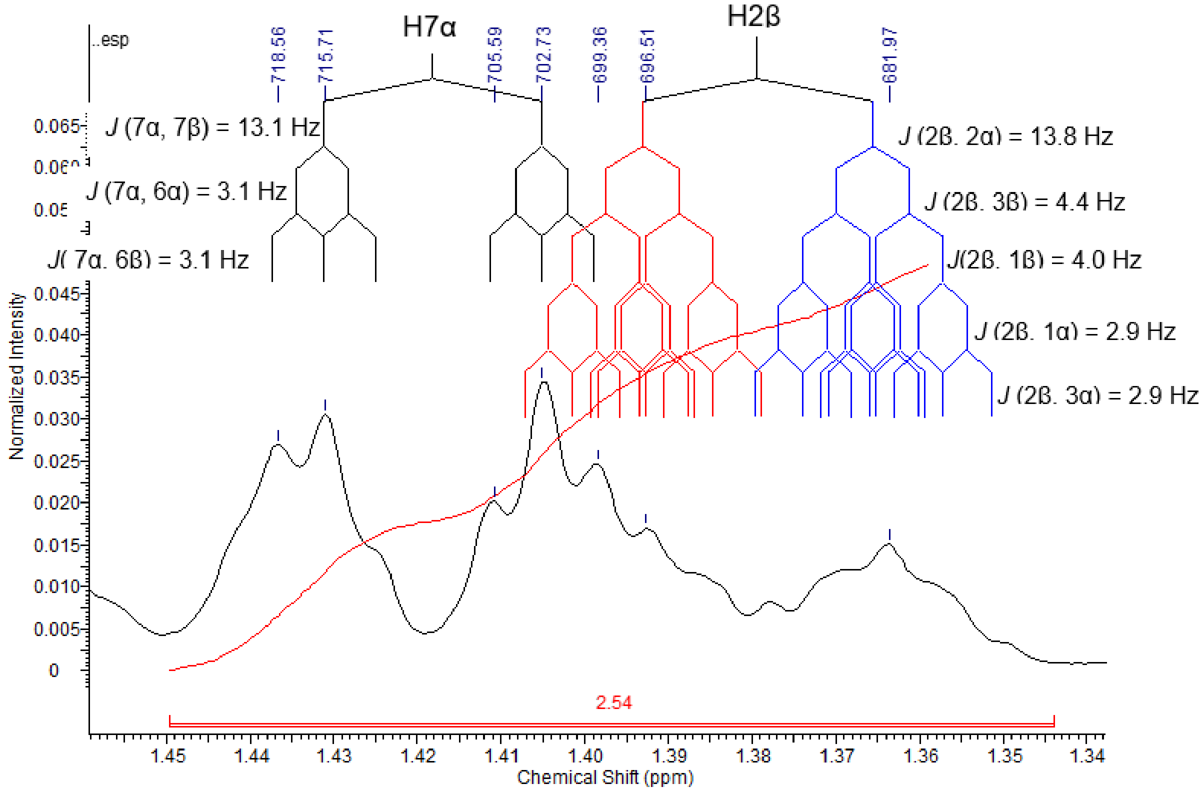

Figure S36. Signal of H7α and H2β in the ^{1}H NMR spectrum (500.13 MHz) of kaurenoic acid in C_6D_6.

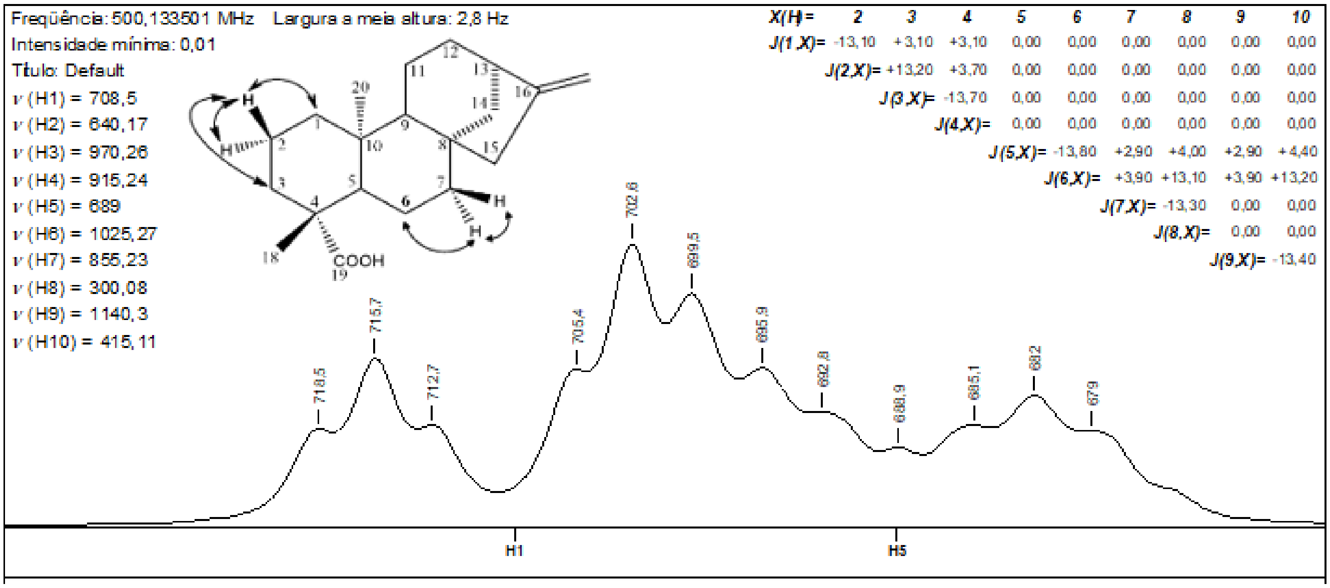

X(H)=	2	3	4	5	6	7	8	9	10
J(1,X)=	-13,10	+3,10	+3,10	0,00	0,00	0,00	0,00	0,00	0,00
J(2,X)=		+13,20	+3,70	0,00	0,00	0,00	0,00	0,00	0,00
J(3,X)=			-13,70	0,00	0,00	0,00	0,00	0,00	0,00
J(4,X)=				0,00	0,00	0,00	0,00	0,00	0,00
J(5,X)=					-13,80	+2,90	+4,00	+2,90	+4,40
J(6,X)=						+3,90	+13,10	+3,90	+13,20
J(7,X)=							-13,30	0,00	0,00
J(8,X)=								0,00	0,00
J(9,X)=									-13,40

Figure S37. Dataset for the simulation of the H7α and H2β signals in the NMR_MultSim program.

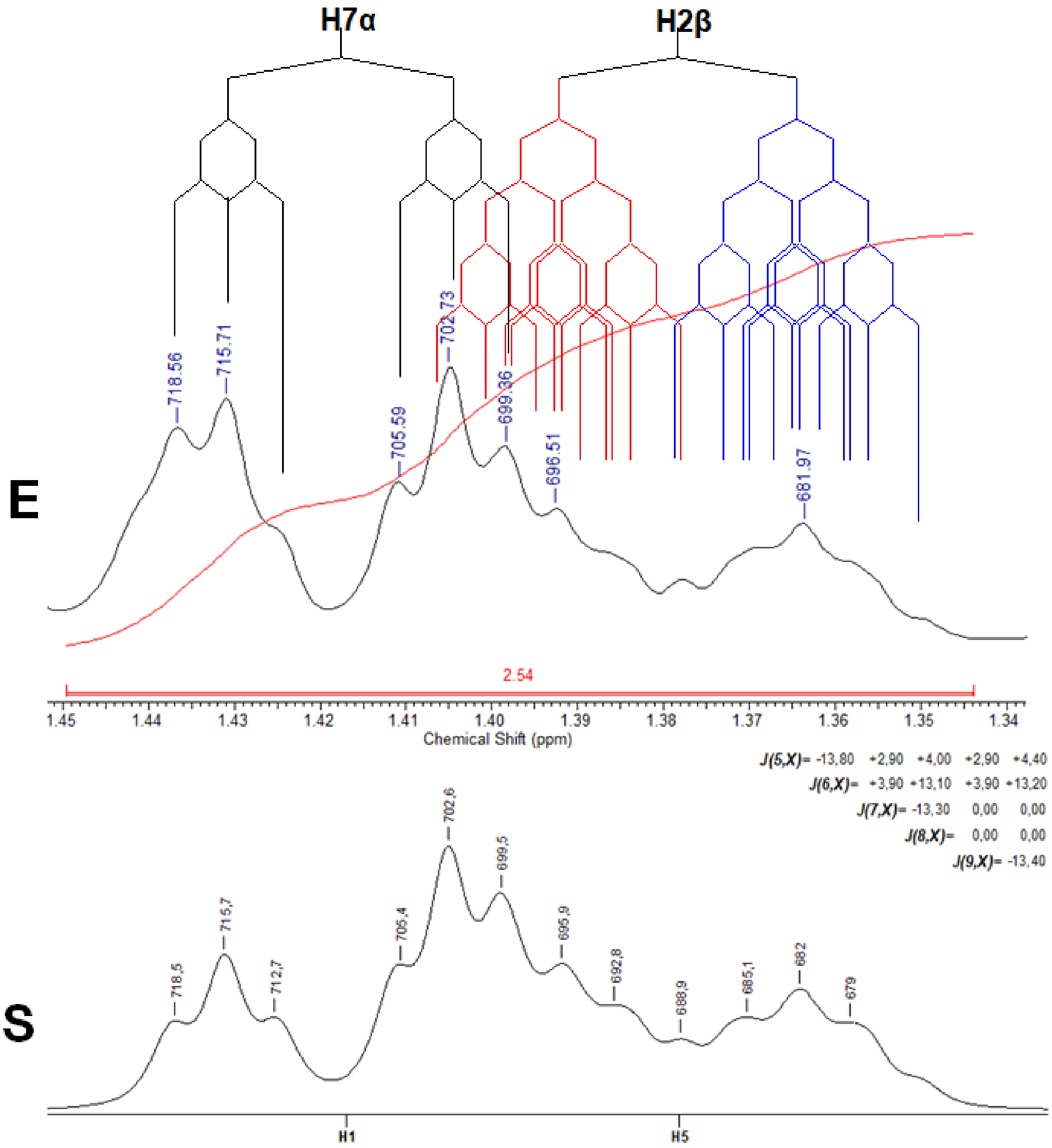

Figure S38. Experimental (**E**) and simulated (**S**) ^{1}H NMR signals H7α and H2β of *ent*-kaurenoic acid (C_6D_6).

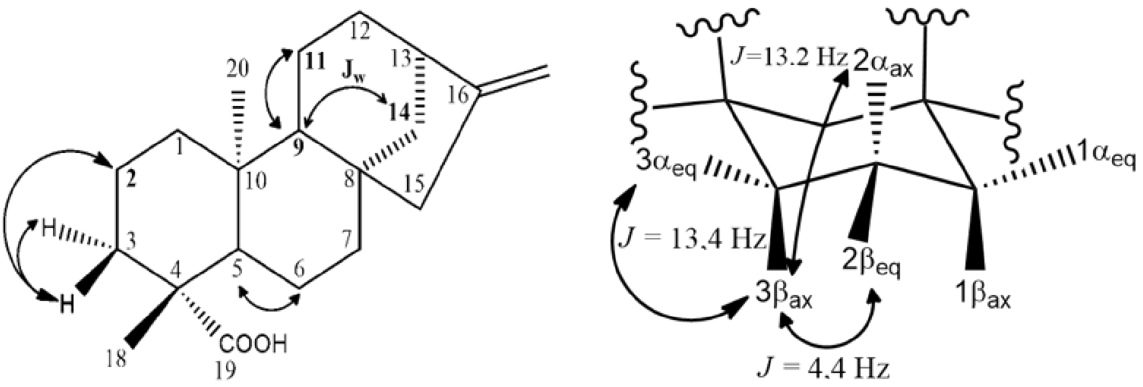

Figure S39. Spatial conformation of the ring region of the kaurenoic acid structure showing possible correlations for H3β.

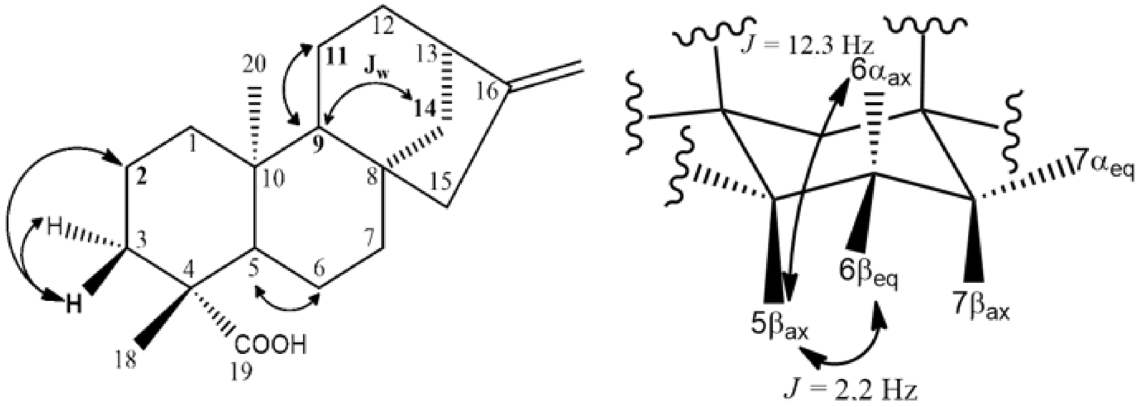

Figure S40. Spatial conformation of the ring region of the kaurenoic acid structure showing possible correlations for H5β.

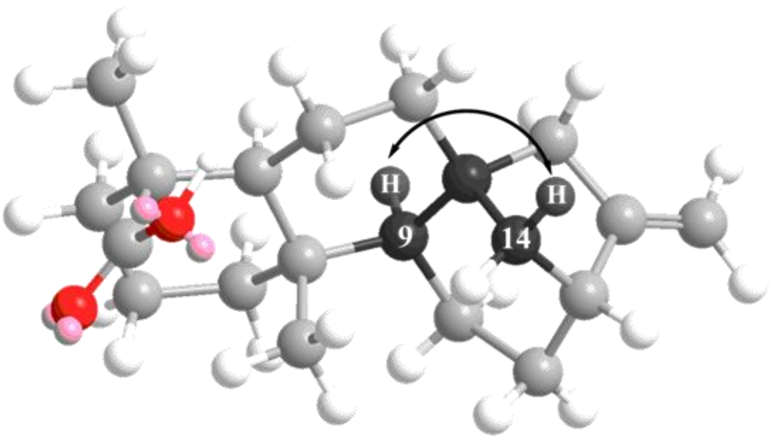

Figure S41. 3D image demonstrating $^{4}J_{W}(9\beta,14\alpha)$= 1.4 Hz.

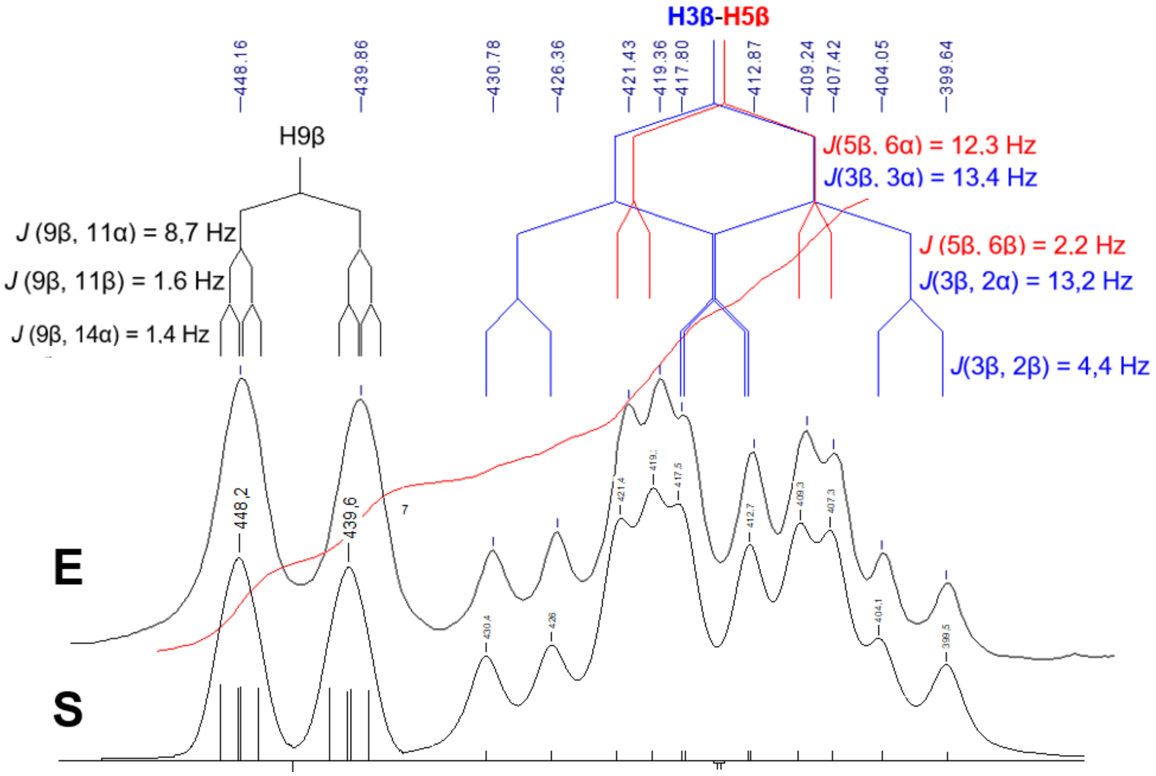

Figure S42. Experimental (**E**) and simulated (**S**) ^{1}H NMR signals H9β, H3β and H5β of *ent*-kaurenoic acid (C_6D_6).

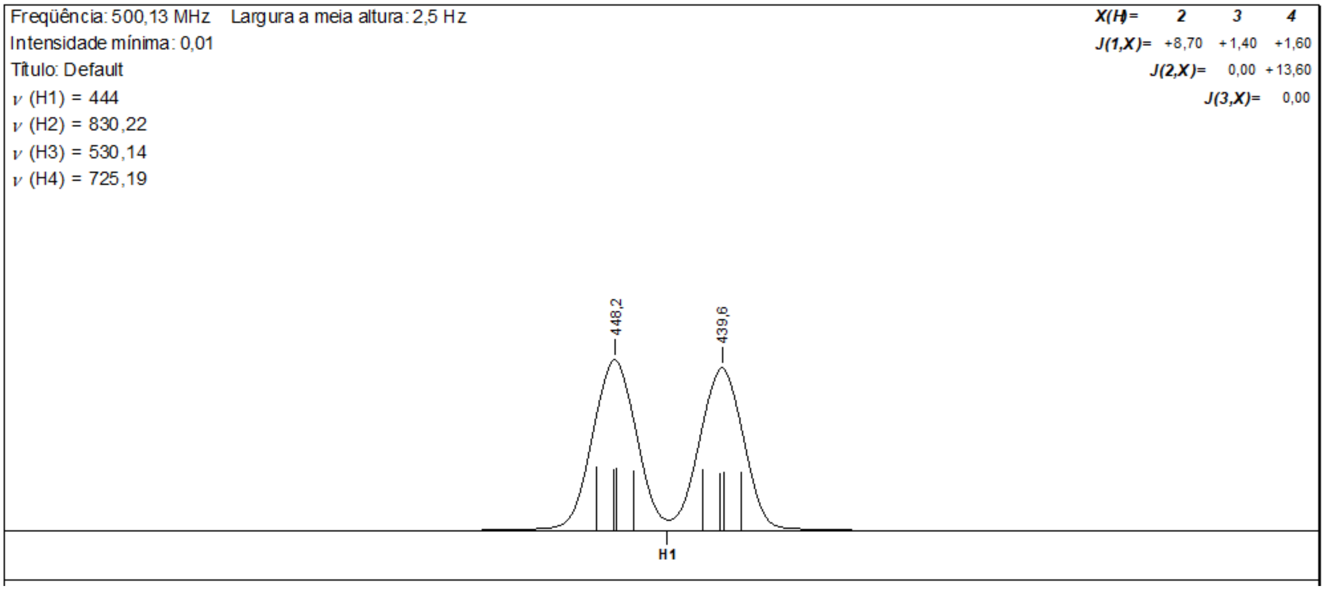

Figure S43. Dataset for the simulation of the H9β signal in the NMR_MultSim program.

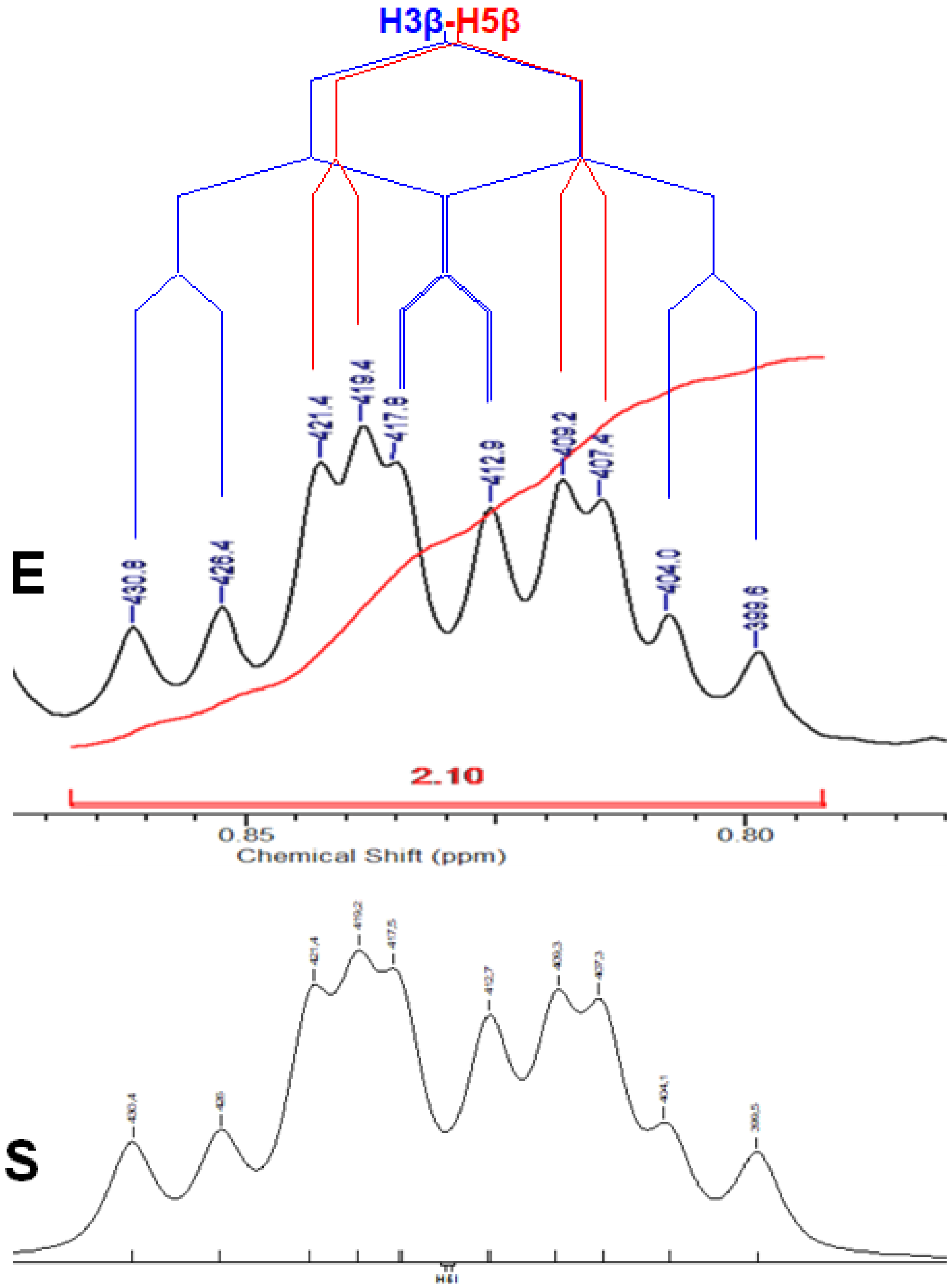

Figure S44. Experimental (**E**) and simulated (**S**) ^{1}H NMR signals H3β and H5β of *ent*-kaurenoic acid (C_6D_6).

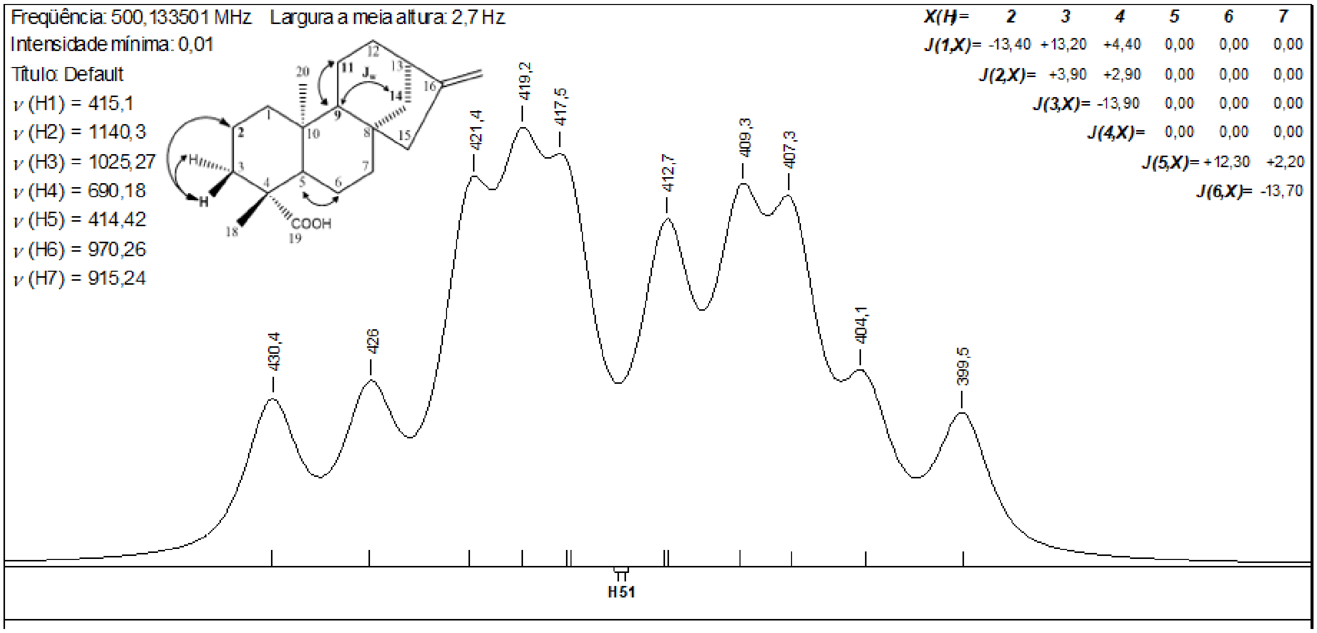

Figure S45. Dataset for the simulation of the H5β and H3β signals in the NMR_MultSim program.

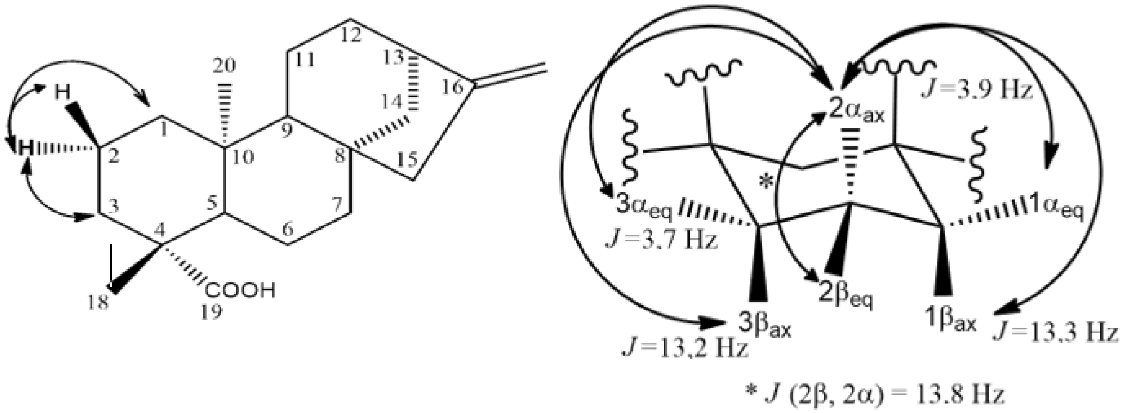

Figure S46. Spatial conformation of the ring region of the kaurenoic acid structure showing possible correlations for H2α.

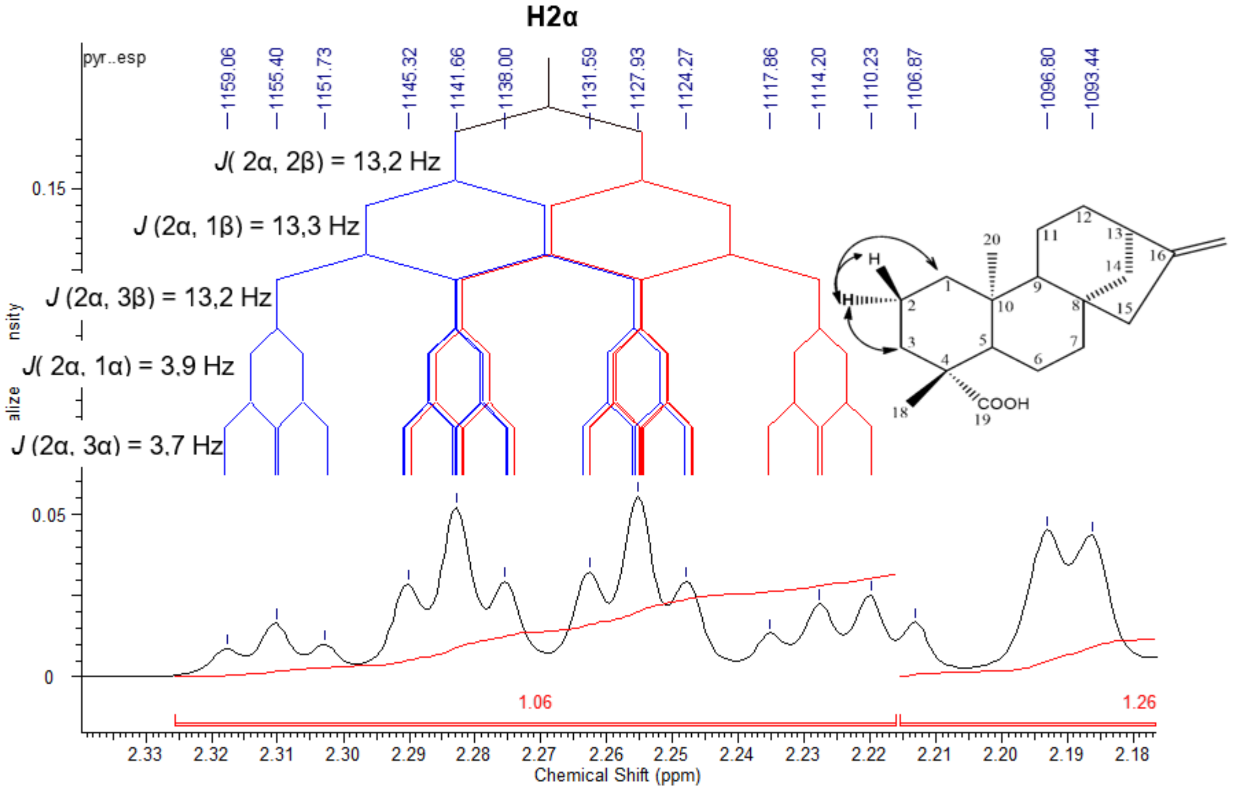

Figure S47. Signal of H2α in the ^{1}H NMR spectrum (500.13 MHz) of kaurenoic acid in C_5D_5N.

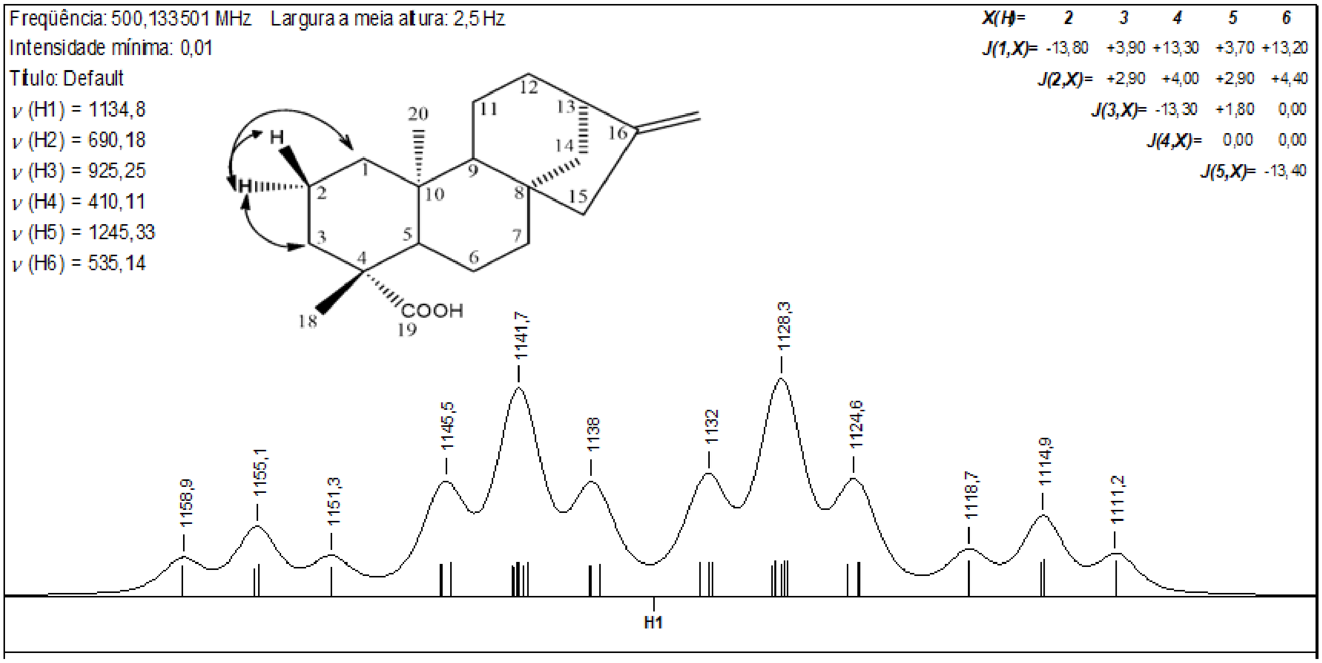

Figure S48. Dataset for the simulation of the H2α signal in the NMR_MultSim program.

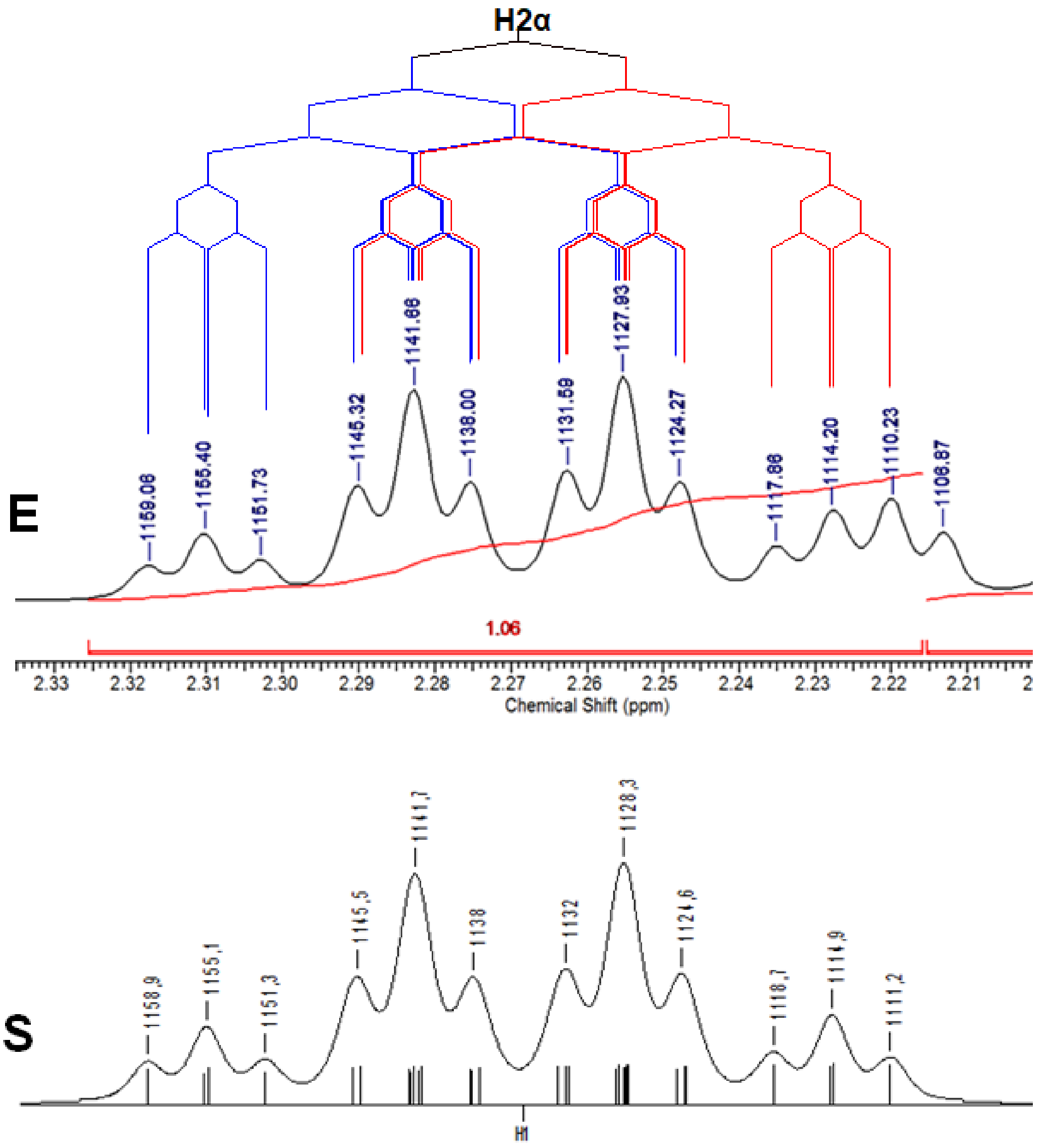

Figure S49. Experimental (**E**) and simulated (**S**) ^{1}H NMR signal H2α of *ent*-kaurenoic acid (C_5D_5N).

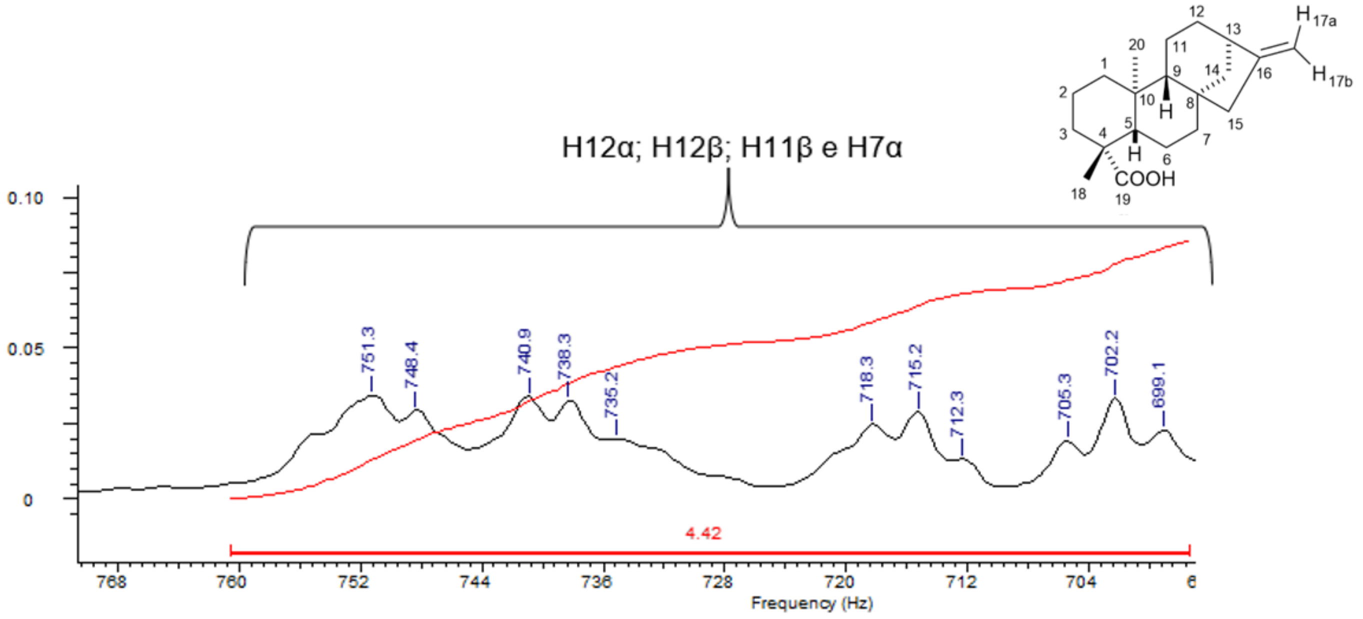

Figures S50. Signals of H12α, H12β, H11β, and H7α in the ^{1}H NMR spectrum (500.13 MHz) of kaurenoic acid in C_6D_6.

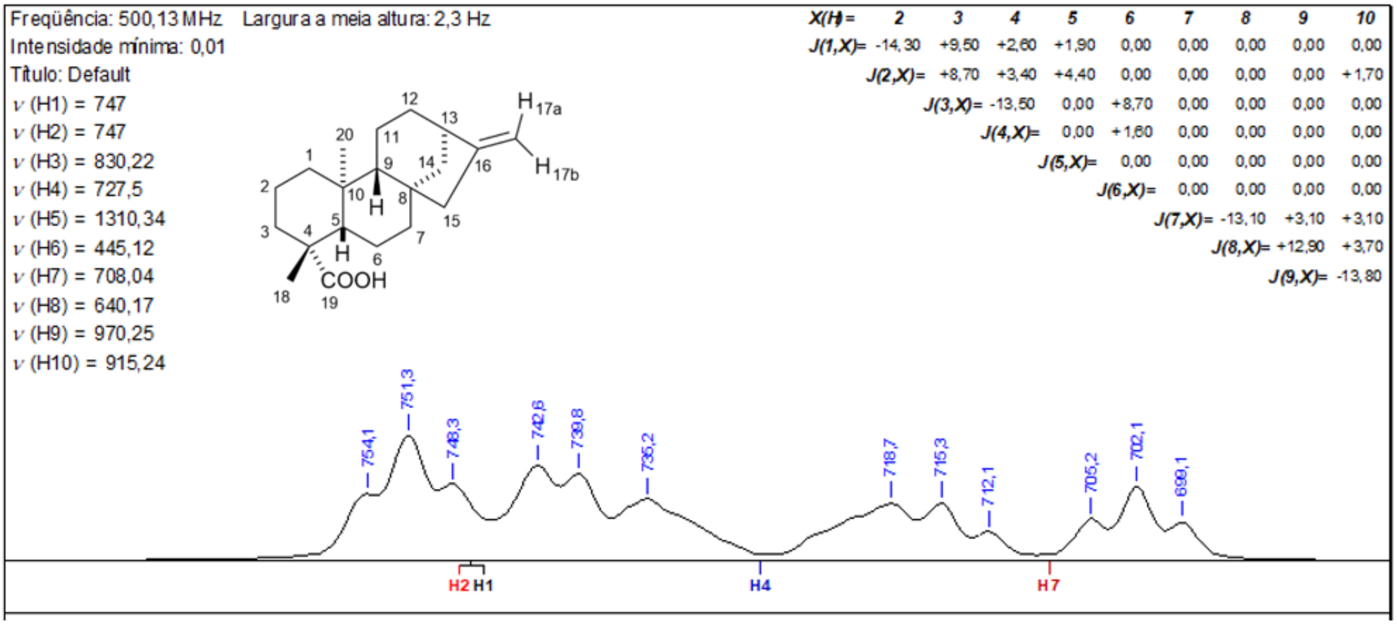

Figures S51. Dataset for the simulation of the H12α, H12β, H11β, and H7α signals in the NMR_MultSim program.

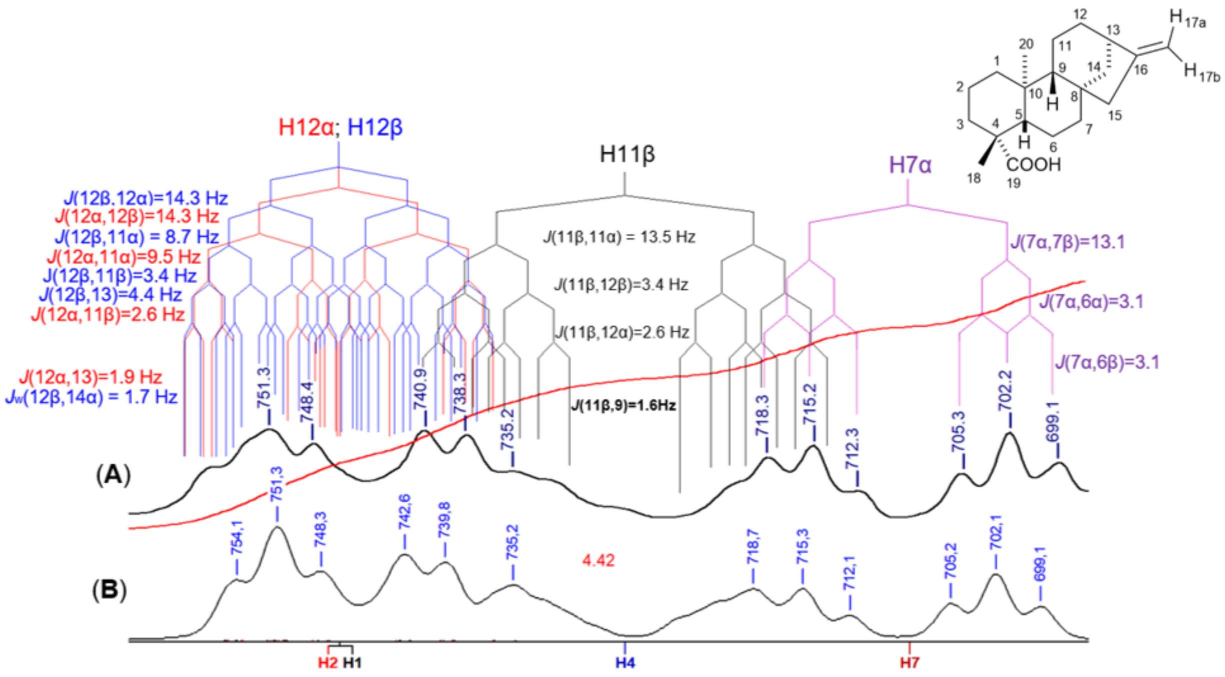

Figure S52. Expansion (A) and Simulation (B) of the signals of H12α, H12β, H11β, and H7α in the NMR_MultSim program.

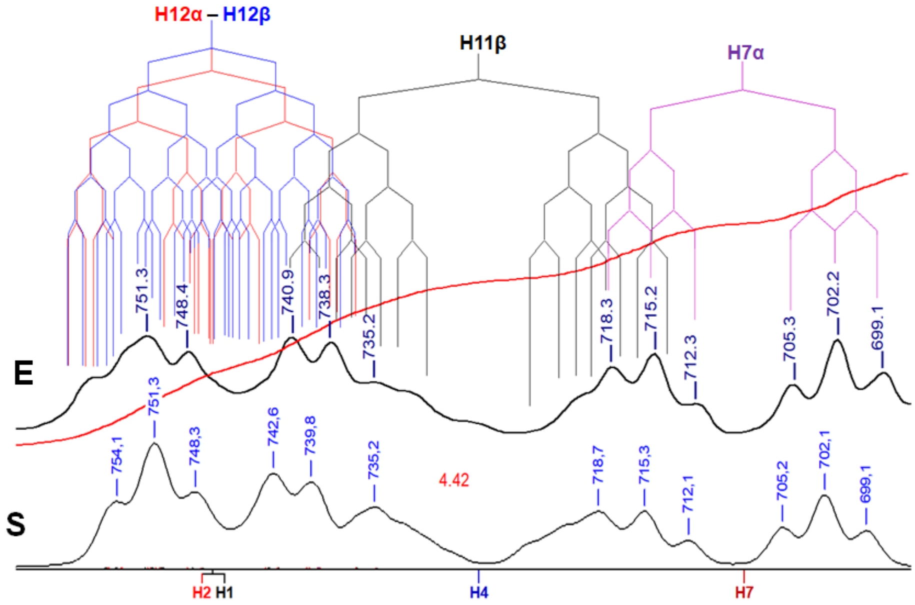

Figure S53. Experimental (**E**) and simulated (**S**) ^{1}H NMR signals H12α, H12β and H7α of *ent*-kaurenoic acid (C_6D_6).

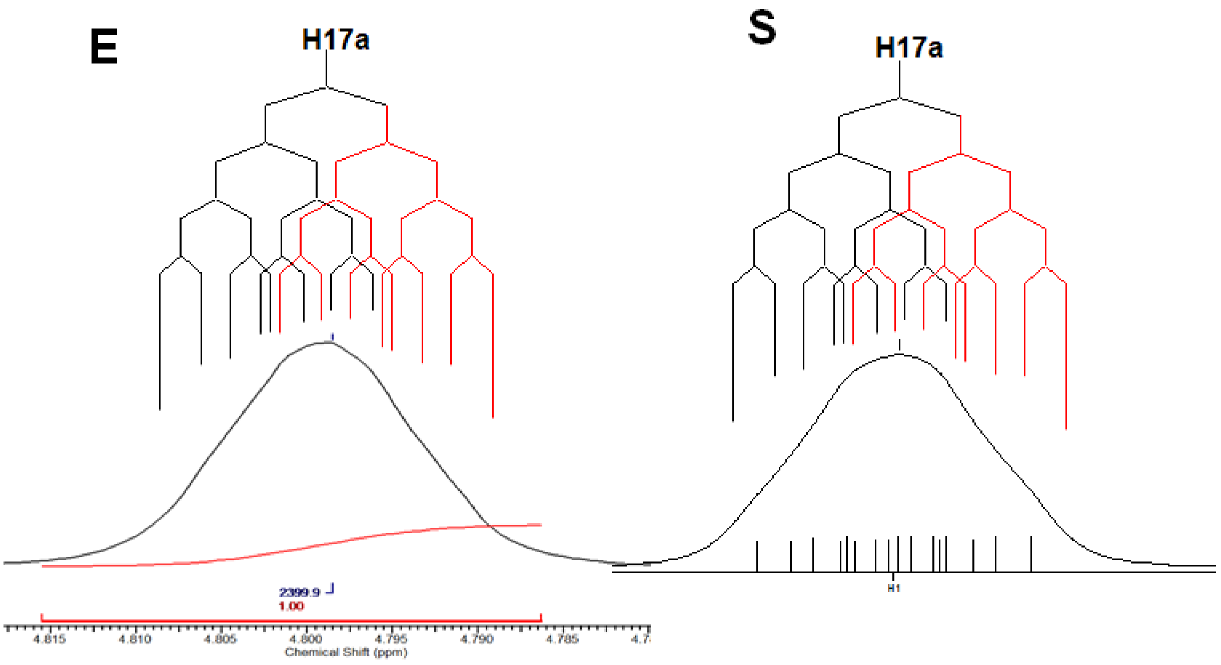

Figure S54. Experimental (**E**) and simulated (**S**) ^{1}H NMR signal H17a of *ent*-kaurenoic acid ($CDCl_3$).

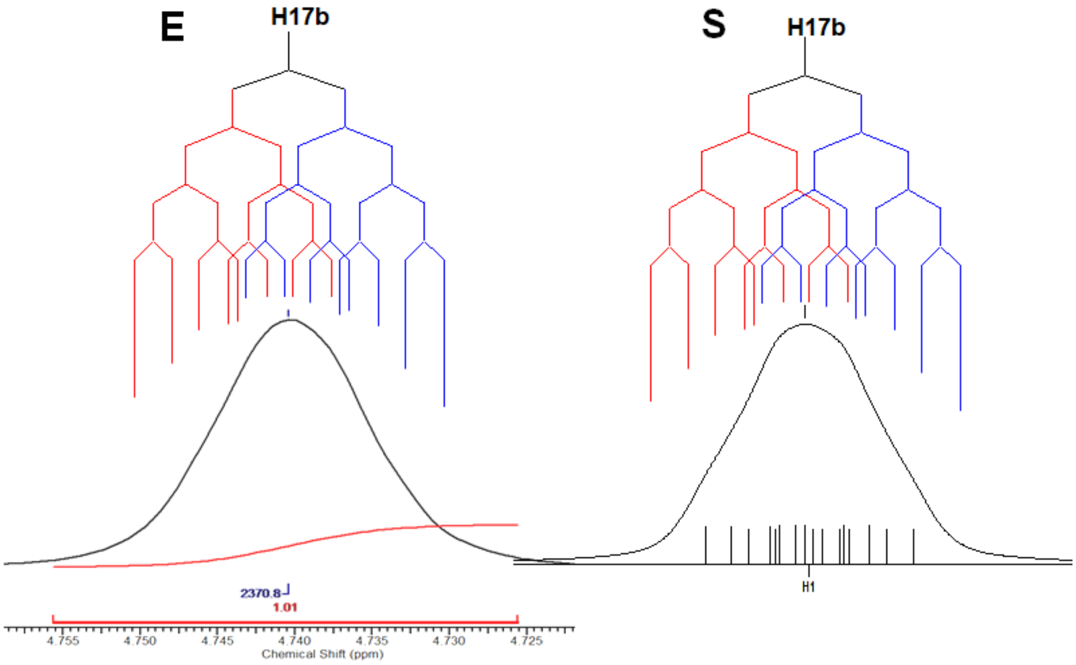

Figure S55. Experimental (**E**) and simulated (**S**) ^{1}H NMR signal H17b of *ent*-kaurenoic acid ($CDCl_3$).

IUPAC NOMENCLATURE FOR METHYL ENT-KAUR-16-EN-19-OATE

Methyl ent-kaur-16-en-19-oate:

methyl 5,9-dimethyl-14-methylidenetetracyclo[11.2.1.0.0]hexadecane-5-carboxylate

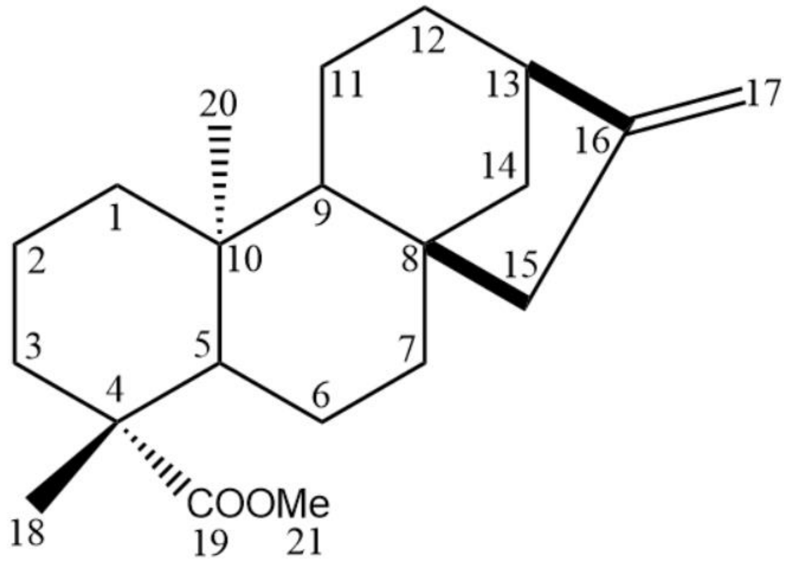

Figure S56. Detailed structure with numbering of Methyl ent-kaur-16-en-19-oate

Source: VIEIRA *et al*., 2002 [2]

3D figures of Methyl ent-kaur-16-en-19-oate

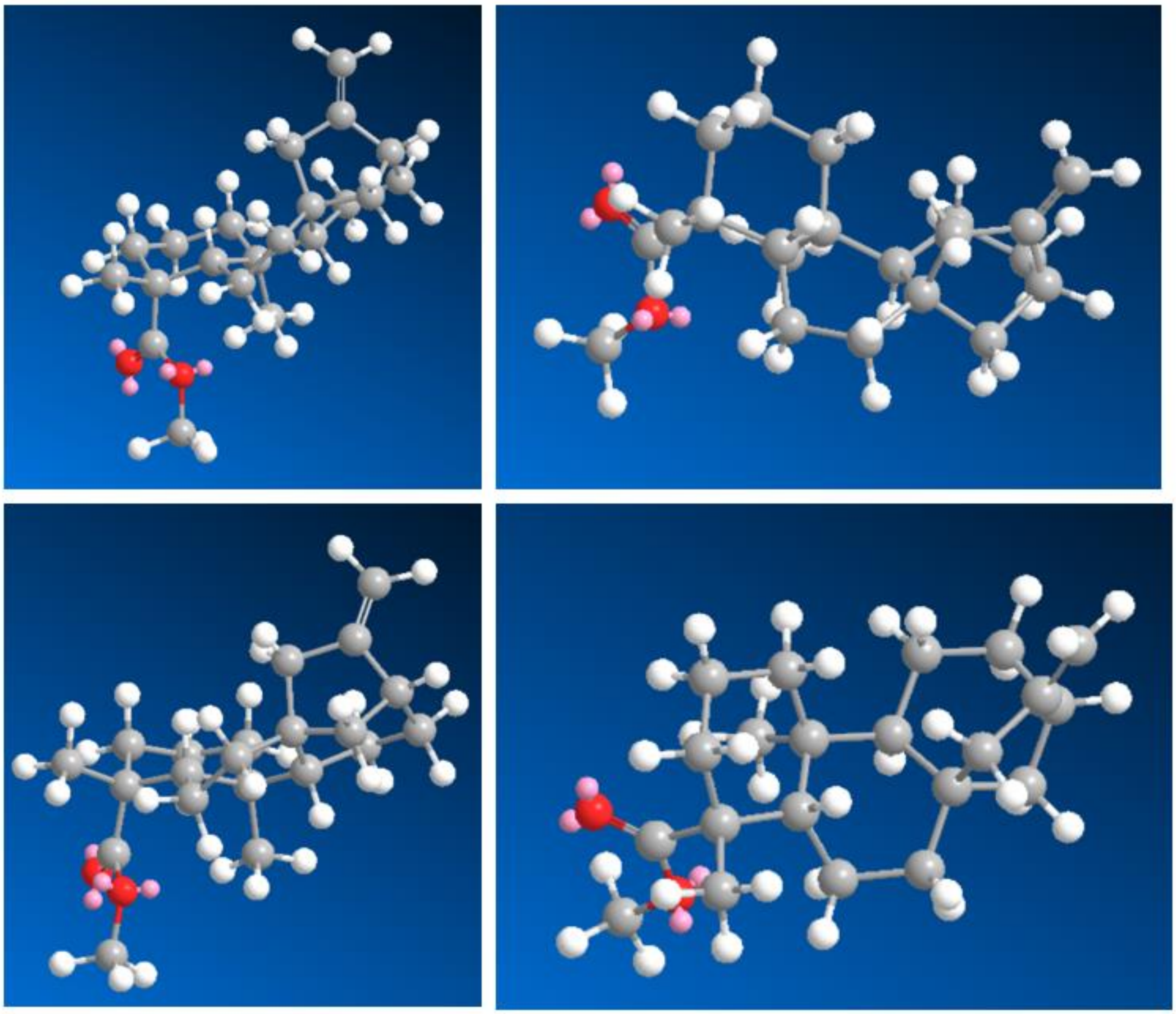

Source: Chem3D – MM2

I. SIMULATED AND EXPERIMENTAL SIGNALS

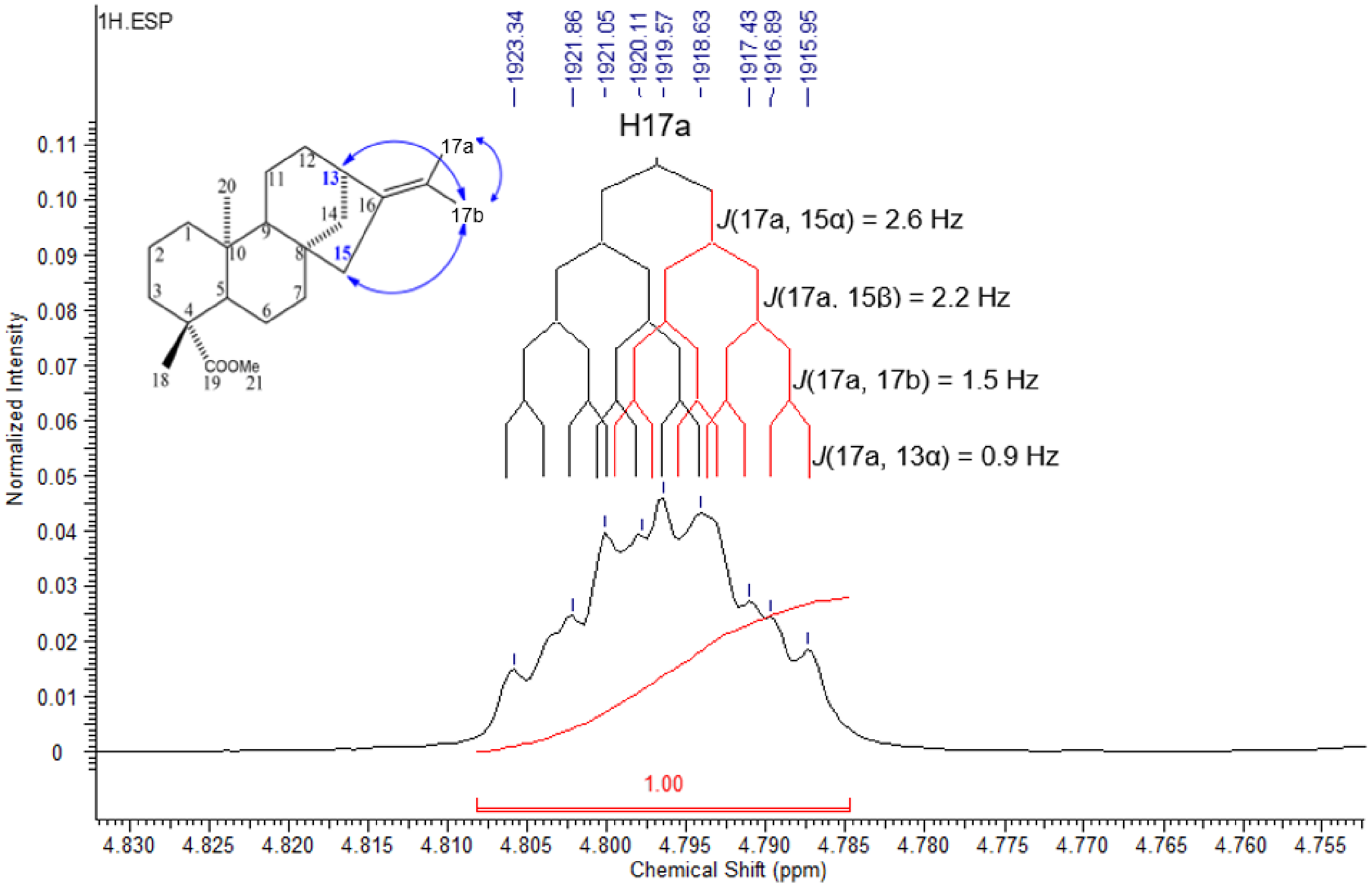

Figure S57. Signal of H17a in the ^{1}H NMR spectrum (400.21 MHz) of the methyl ent-kaur-16-en-19-oate.

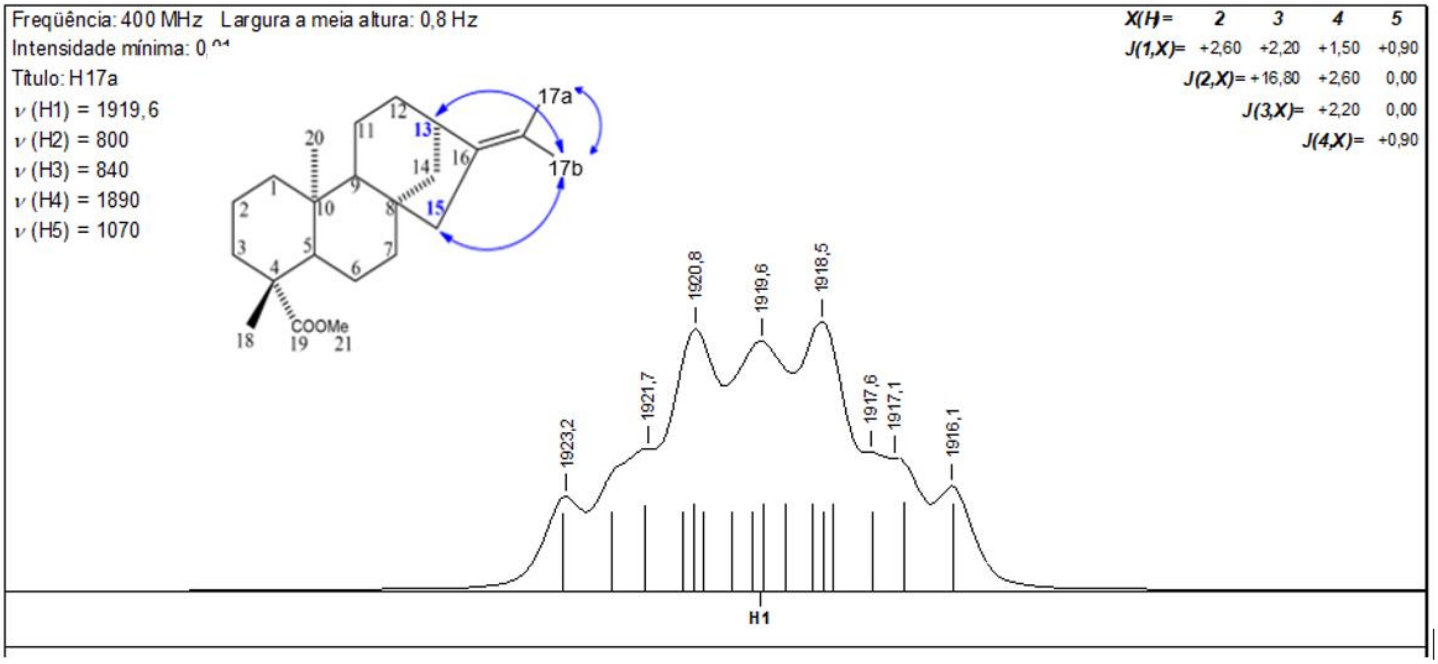

Figure S58. Dataset for the simulation of the H17a signal in the NMR_MultSim program.

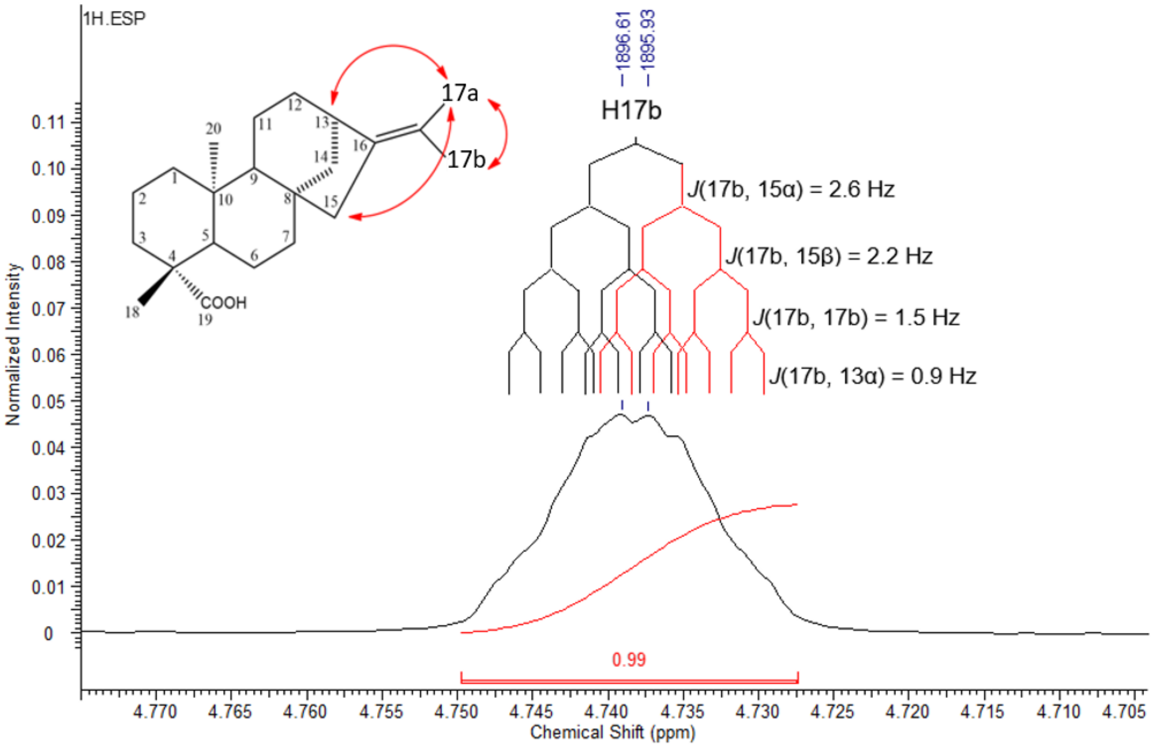

Figure S59. Signal of H17b in the ^{1}H NMR spectrum (400.21 MHz) of the methyl ent-kaur-16-en-19-oate.

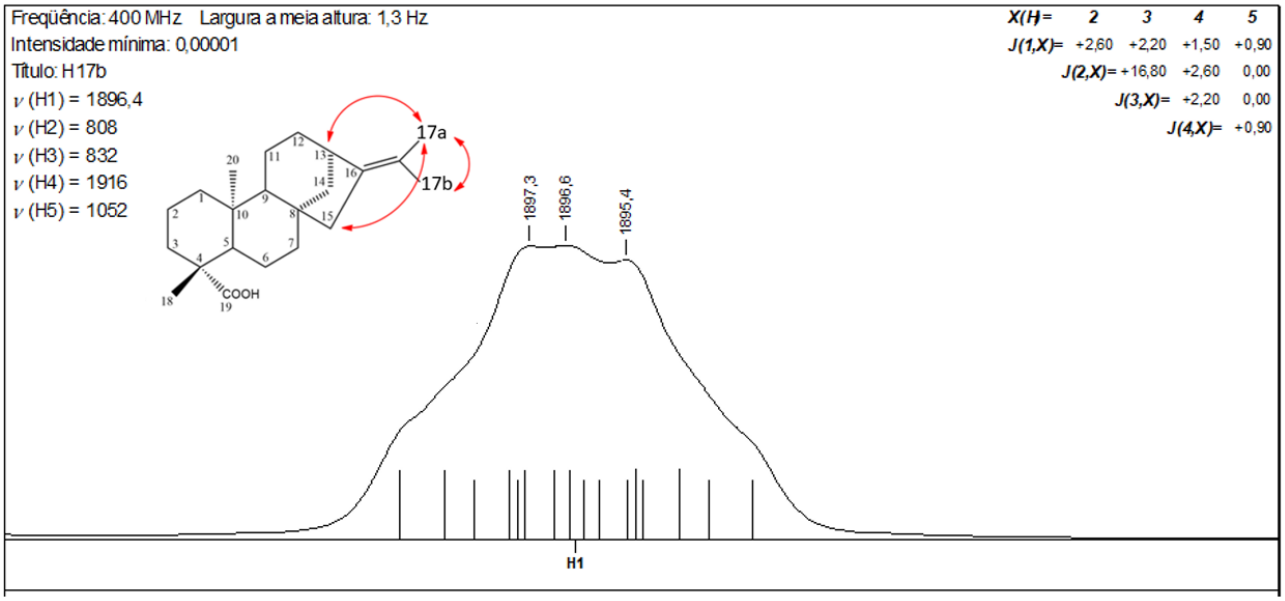

Figure S60. Dataset for the simulation of the H17b signal in the NMR_MultSim program.

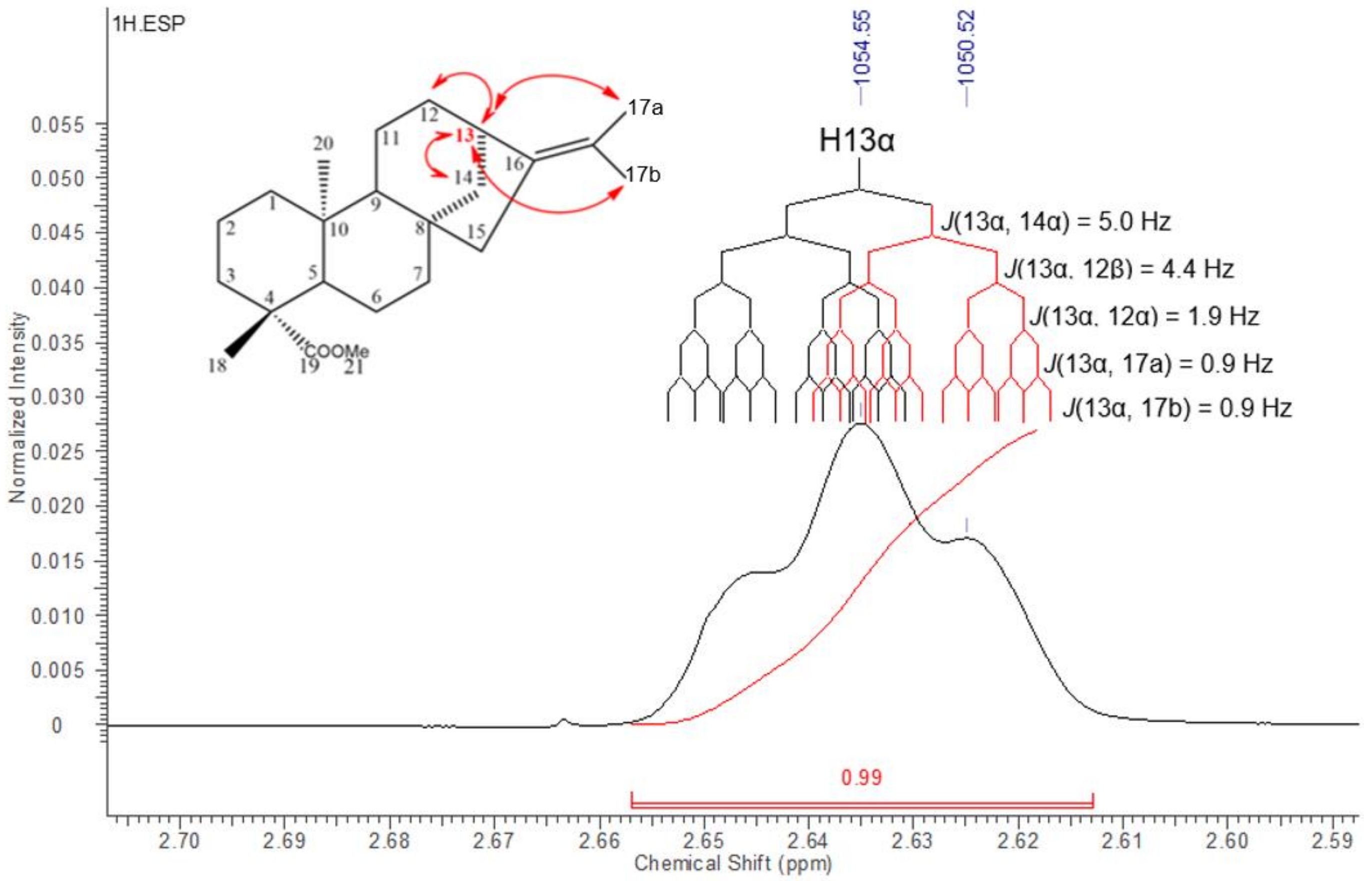

Figure S61. Signal of H13α in the ^{1}H NMR spectrum (400.21 MHz) of the methyl ent-kaur-16-en-19-oate.

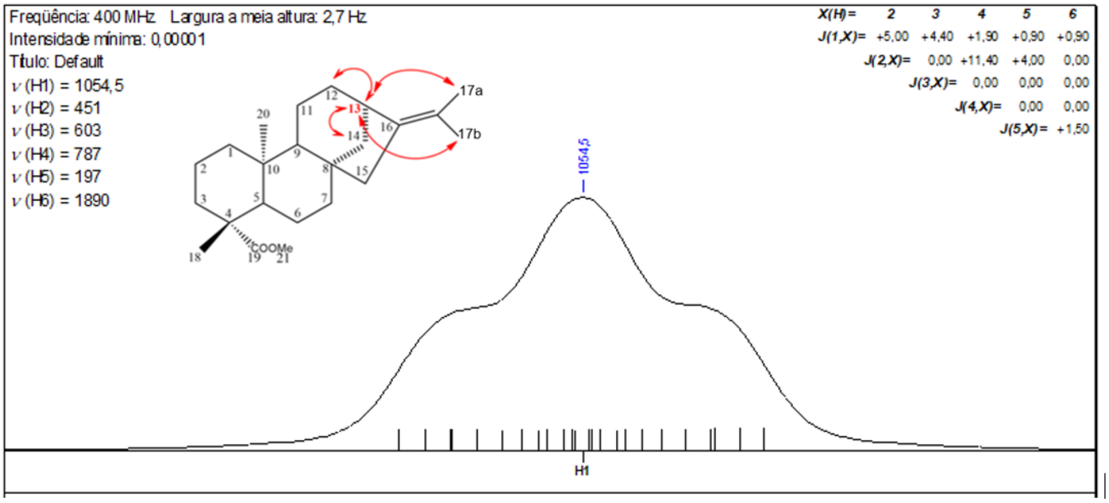

Figure S62. Dataset for the simulation of the H13α signal in the NMR_MultSim program.

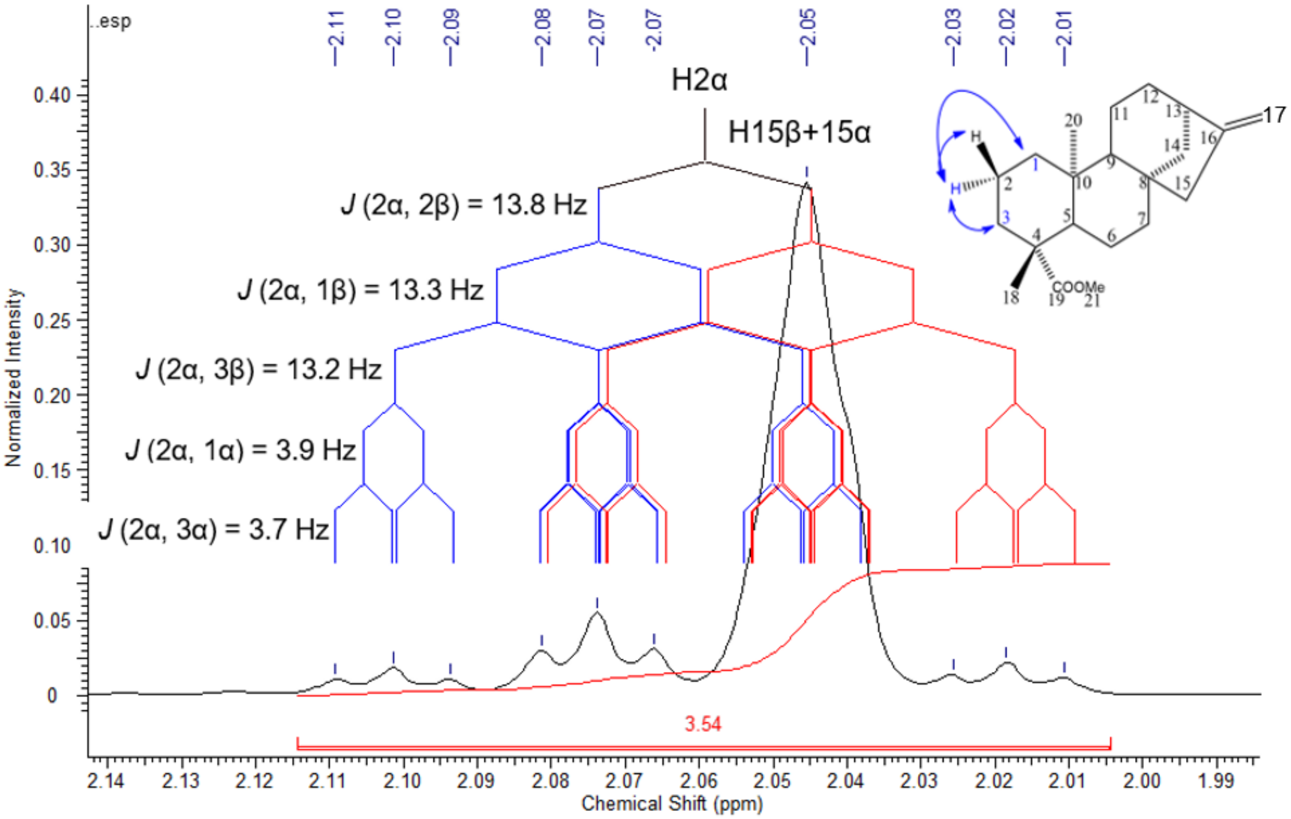

Figure S63. Signal of H2α in the ^{1}H NMR spectrum (500.13 MHz) of the methyl ester of kaurenoic acid in C_6D_6.

II. 2D-NMR DATA SECTION

In this section, the 2D NMR data set for *ent*-kaurenoic acid.

Table S1. 2D NMR data for *ent*-kaurenoic acid – $CDCl_3$, 500 MHz.

C	H	HMBC	COSY	HSQC
1	1α	H-3α,H-20α	H-1β,H-2β	H-1α, H-1β
	1β		H-1α,H-2α, H-2β	
2	2α	H-1α, H-1β,H-3α,H-3β	H-2β,H-3β	H-2α, H-2β
	2β		H-1β,H-3β	
3	3α	H-1α, H-2α, H-18β	H-2β, H-3β	H-3α, H-3β
	3β		H-2β, H2α, H-3α	
4	---	H-3α, H-3β,H-5β,H-18β		
5	5β	H-1β,H-1α,H-3α,H-3β,H-6α,H-6β,H-9β,H-18β,H-20α	H-6α, H-6β	H-5β
6	6α	H-5β,H-7α,H-7β	H-5β, H-7α, H7β	H-6α, H-6β
	6β		H-5β, H-7α, H7β	
7	7α	H-5β, H-6α, H-6β	H-6α, H-6β	H-7α, H-7β
	7β		H-6α, H-6β	
8	---	H-7α, H-7β, H-9β, H-14β, H-15α, H-15β		
9	9β	H-1α, H-11, H-12, H-14, H-15	H-11α	H-9β
10	---	H-1α, H-1β, H-5β, H-9β, H-20α		
11	11β	H-9β, H-12α,H-12β,H-13α	H-9β	H-11α,H11β
	11α		H-9β	
12	12α	H-11α, H-14α, H-14β, H-17a, H-17b	H-13α	H-12α,H-12β
	12β		H-13α	
13	13α	H-14α, H-14β, H-17a, H-17b	H-12β, H-14α	H-13α
14	14α	H-11α, H-11β, H-12α, H-12β	H-13α, H-14β	H-14α,H14β
	14β		H-14α	
15	15α	H-7β, H-13α, H-14α, H-14β, H-17a, H-17b	H-17a, H-17b	H-15α,H15β
	15β		H-17a, H-17b	
16	---	H-11α,H-11β,H-12α,H-12β,H-13α,H-14β,H-15α,H-15β, H-17a,H-17b		
17	17a	H-13α, H-14β, H-15α, H-15β	H-15α, H-15β	H-17a, H-17b
	17b		H-15α, H-15β	
18	18β	H-3β, H-5β		H-18β
19	---	H-3α, H-3β, H-5β, H-18β		
20	20α	H-1α, H-1β, H-5β, H-9β		H-20α

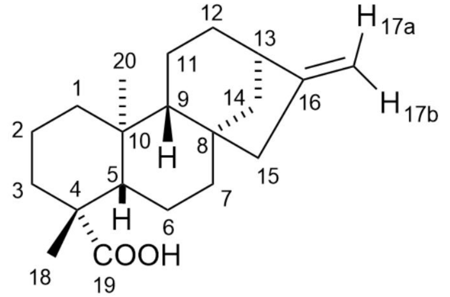

ent-kaurenoic acid

Table S2. 2D NMR data for *ent*-kaurenoic acid – CD_3OD, 600 MHz.

C	H	HMBC	COSY	HSQC
1	1α	H-3α,H-20α	H-1β	H-1α, H-1β
	1β		H-1α,H-2α, H-2β	
2	2α	H-1β,H-3α,H-3β	H-3β	H-2α, H-2β
	2β		H-1β, H-2α	
3	3α	H-1 β, H-18β	H-3β	H-3α, H-3β
	3β		H-2β, H-3α	
4	---	H-3α, H-5β,H-18β		
5	5β	H-1β, H-3α, H-9β,H-18β,H-20α	H-6α, H-6β	H-5β
6	6α	H-5β, H-7β	H-5β, H-7α	H-6α, H-6β
	6β		H-5β, H-7α	
7	7α	H-5β	H-6α, H-6β	H-7α, H-7β
	7β		H-6α, H-6β	
8	---	H-7β, H-9β, 13α, H-14β, H-15α, H-15β		
9	9β	H-1β, H-14α, H-14β, H-15α, H-15β	H-11α	H-9β
10	---	H-1β, H-5β, H-9β, H-20α		
11	11β	H-9β ,H-13α	H-9β	H-11α,H11β
	11α		H-9β	
12	12α	H-13α, H-14α, H-14β	H-13α	H-12α,H-12β
	12β		H-13α	
13	13α	H-14α, H-14β, H-17a, H-17b	H-14α	H-13α
14	14α	H-11α, H-11β, H-12α, H-12β	H-13α, H-14β	H-14α,H14β
	14β		H-14α	
15	15α	H-13α, H-14α, H-14β, H-17a, H-17b	H-17a, H-17b	H-15α,H15β
	15β		H-17a, H-17b	
16	---	H-12α,H-12β,H-13α,H-14β,H-15α,H-15β,H-17a,H-17b		
17	17a	H-13α, H-14β, H-15α, H-15β	H-13α,H-15α,H-15β	H-17a, H-17b
	17b		H-13α,H-15α,H-15β	
18	18β	H-3α, H-5β		H-18β
19	---	H-3α, H-3β, H-5β, H-18β		
20	20α	H-1β, H-5β, H-9β		H-20α

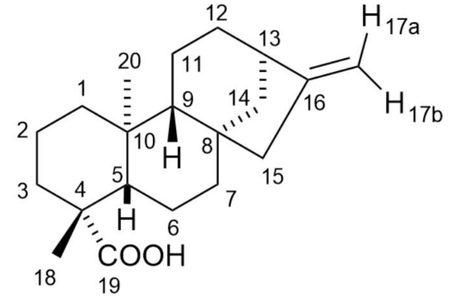

ent-kaurenoic acid

Table S3. 2D NMR data for *ent*-kaurenoic acid – C_6D_6, 500 MHz.

C	H	HMBC	COSY	HSQC
1	1α	H-3α, H-20α	H-1β, H-2α, H-2β, H-3α	H-1α, H-1β
	1β		H-1α, H-2α, H-2β	
2	2α	H-1α, H-1β,H-3α, H-3β	H-1α, H-1β, H-2β, H3α, H-3β	H-2α, H-2β
	2β		H-1α, H-1β, H-2α, H3α, H-3β	
3	3α	H-1α,H-1β, H-2α, H-2β, H-18β	H-1α, H-2α, H-2β, H-3β	H-3α, H-3β
	3β		H-2α, H2β, H-3α	
4	---	H-3α, H-3β, H-5β, H-18β		
5	5β	H-1β, H-1α, H-3α, H-3β, H-6α, H-6β, H-7α, H-7β H-9β,H-18β, H-20α	H-6α, H-6β	H-5β
6	6α	H-5β, H-7α, H-7β	H-5β, H-6β H-7α, H-7β	H-6α, H-6β
	6β		H-5β, H-6α H-7α, H-7β	
7	7α	H-5β, H-6α, H-6β	H-6α, H6β, H-7β	H-7α, H-7β
	7β		H-6α, H6β, H-7α	
8	---	H-7α, H-7β, H-9β, H-14β, H-15α, H-15β		
9	9β	H-1α,H-1β,H-5β,H-11α,H-11β,H-12α,H-12β,H-14α, H-14β, H-20α	H-11α	H-9β
10	---	H-1α, H-1β, H-5β, H-9β, H-20α		
11	11β	H-9β, H-12α,H-12β, H-13α	H-11α	H-11α,H11β
	11α		H-9β, H-11β,H-12α, H-12β	
12	12α	H-11α, H11β, H13α, H-14α, H-14β, H-17a, H-17b	H-11α, H-13α	H-12α,H-12β
	12β		H11α, H-13α	
13	13α	H11β, H-12α, H-12β,H-14α,H-14β,H-17a, H-17b	H12α,H-12β, H-14α	H-13α
14	14α	H-11α, H-11β, H-12α, H-12β, H-15α, H-15β	H-13α, H-14β	H-14α,H14β
	14β		H-14α	
15	15α	H-7β, H-9β, H-13α, H-14α, H-14β, H-17a, H-17b	H-17a, H-17b	H-15α,H15β
	15β		H-17a, H-17b	
16	---	H-11α,H-11β,H-12α,H-12β,H-13α,H-14α,H-14β,H-15α, H-15β, H-17a, H-17b		
17	17a	H-13α, H-14β, H-15α, H-15β	H-13α,H-15α,H-15β	H-17a, H-17b
	17b		H-13α,H-15α,H-15β	
18	18β	H-3α, H-3β, H-5β		H-18β
19	---	H-3α, H-3β, H-5β, H-18β		
20	20α	H-1α, H-1β, H-5β, H-9β		H-20α

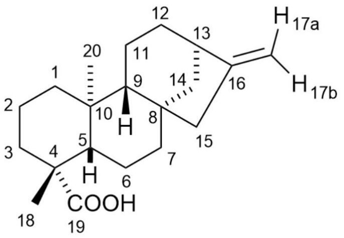

ent-kaurenoic acid

Table S4. 2D NMR data for *ent*-kaurenoic acid – C_5D_5N, 500 MHz.

C	H	HMBC	COSY	HSQC
1	1α	H-3α, H-20α	H-1β, H-2α, H-2β	H-1α, H-1β
	1β		H-1α, H-2α, H-2β	
2	2α	H-1α, H-1β,H-3α, H-3β	H-1α, H-1β, H-2β, H-3β	H-2α, H-2β
	2β		H-1β, H-3β	
3	3α	H-1α,H-1β, H-2α, H-18β	H-2α, H-2β, H-3β	H-3α, H-3β
	3β		H-3α	
4	---	H-3α, H-3β, H-5β, H-18β		
5	5β	H-1β, H-1α, H-3α, H-3β, H-6α, H-7α, H-7β H-9β,H-18β, H-20α	H-6α, H-6β	H-5β
6	6α	H-5β, H-7α, H-7β	H-5β, H-7β	H-6α, H-6β
	6β		H-5β, H-7α	
7	7α	H-5β, H-6α, H-6β	H-6α, H-7β	H-7α, H-7β
	7β		H-6α, H6β, H-7α	
8	---	H-7α, H-7β, H-9β, H-14β, H-15α, H-15β		
9	9β	H-1β,H-5β,H-11α,H-11β,H-12α,H-12β,H-14β,H-20α	H-11α	H-9β
10	---	H-1α, H-1β, H-5β, H-9β, H-11α, H-20α		
11	11β	H-9β, H-12α,H-12β	H-11α	H-11α,H11β
	11α		H-9β	
12	12α	H-11α, H11β,H-14α, H-14β, H-17a, H-17b	H-13α	H-12α,H-12β
	12β		H-13α	
13	13α	H11α, H-12α, H-12β,H-14β,H-17a, H-17b	H12α,H-12β, H-14α	H-13α
14	14α	H-11α, H-11β, H-12α, H-12β, H-15α, H-15β	H-13α, H-14β	H-14α,H14β
	14β		H-14α	
15	15α	H-9β, H-13α, H-14α, H-14β, H-17a, H-17b	H-17a, H-17b	H-15α,H15β
	15β		H-17a, H-17b	
16	---	H-11α,H-11β,H-12α,H-12β,H-14β,H-15α,H-15β, H-17a, H-17b		
17	17a	H-15α, H-15β	H-13α,H-15α, H-15β	H-17a, H-17b
	17b		H-13α,H-15α, H-15β	
18	18β	H-3β, H-5β		H-18β
19	---	H-3α, H-3β, H-5β, H-18β		
20	20α	H-1α, H-1β, H-5β, H-9β		H-20α

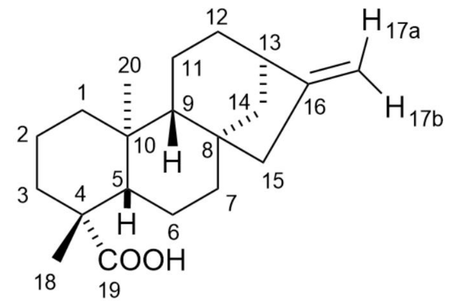

ent-kaurenoic acid

Table S5. 2D NMR data for methyl ent-kaur-16-en-19-oate – C_6D_6, 500 MHz.

C	H	HMBC	COSY	HSQC
1	1α	H-2α, H-3α, H-20α	H-1β, H-2α	H-1α, H-1β
	1β		H-1α, H-2α, H-2β	
2	2 α	H-1α, H-1β,H-3α, H-3β	H-1β, H-2β, 3β	H-2α, H-2β
	2 β		H-2α, H-1α, H-1β, H-3α,H-3β	
3	3 α	H1-α, H-2α, H-2β,H-18β	H-2α, H-2β, H-3β	H-3α, H-3β
	3 β		H-2β, H2α, H-3α	
4	---	H-2α, H-2β, H-3α, H-3β, H-5β, H-18β		
5	5β	H-1β, H-1α,H-3α, H-3β, H-6α, H-6β H-9β,H-18β,H-20α	H-6	H-5β
6	6α	H-5β, H-7α, H-7β	H-5β, H-6, H-7α, H-7β	H-6α, H-6β
	6β		H-5β, H-6, H-7α	
7	7α	H-5β,H-6α, H-6β	H-6	H-7α, H-7β
	7β		H-6	
8	---	H-7α, H-7β, H-9β, H-14α, H-14β, H-15α, H-15β, H-17a, H-17b		
9	9β	H-1β, H-11α, H-12, H-15, H-20α	H-11α	H-9β
10	---	H-1α, H-1β, H-5β, H-9β, H-20α		
11	11α	H-9β, H-12, H-13α	H-9, H-11β	H-11α,H-11β
	11β		H-9, H-11α	
12	12α	H-11α, H-11β,H-13α, H-14α, H-14β	H-13α	H-12α,H-12β
	12β		H-13α	
13	13α	H-14α,H-17a, H-17b	H-12α, H-12β, H-14α	H-13α
14	14β	H-11α,H-12, H-13α	H-14α	H-14α,H-14β
	14α		H-13α, H-14β	
15	15β	H-7α, H-7β,H-13α, H-14α, H-14β, H-17a, H-17b	H-17a, H-17b	H-15α,H-15β
	15α		H-17a, H-17b	
16	---	H-14β, H-15α, H-15β, H-17a, H-17b		
17	17a	H-13α, H-14β, H-15α, H-15β	H-15α, H-15β	H-17a,H-17b
	17b		H-15α, H-15β	
18	18β	H-3α, H-3β, H-5β		H-18β
19	---	H-3α, H-5β, H-18β		
20	20α	H-1α, H-1β, H-5β, H-9β		H-20α
21	21α			H-21α

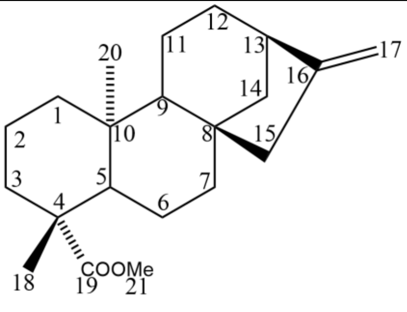

methyl ent-kaur-16-en-19-oate

III. SPECTRAL SECTION

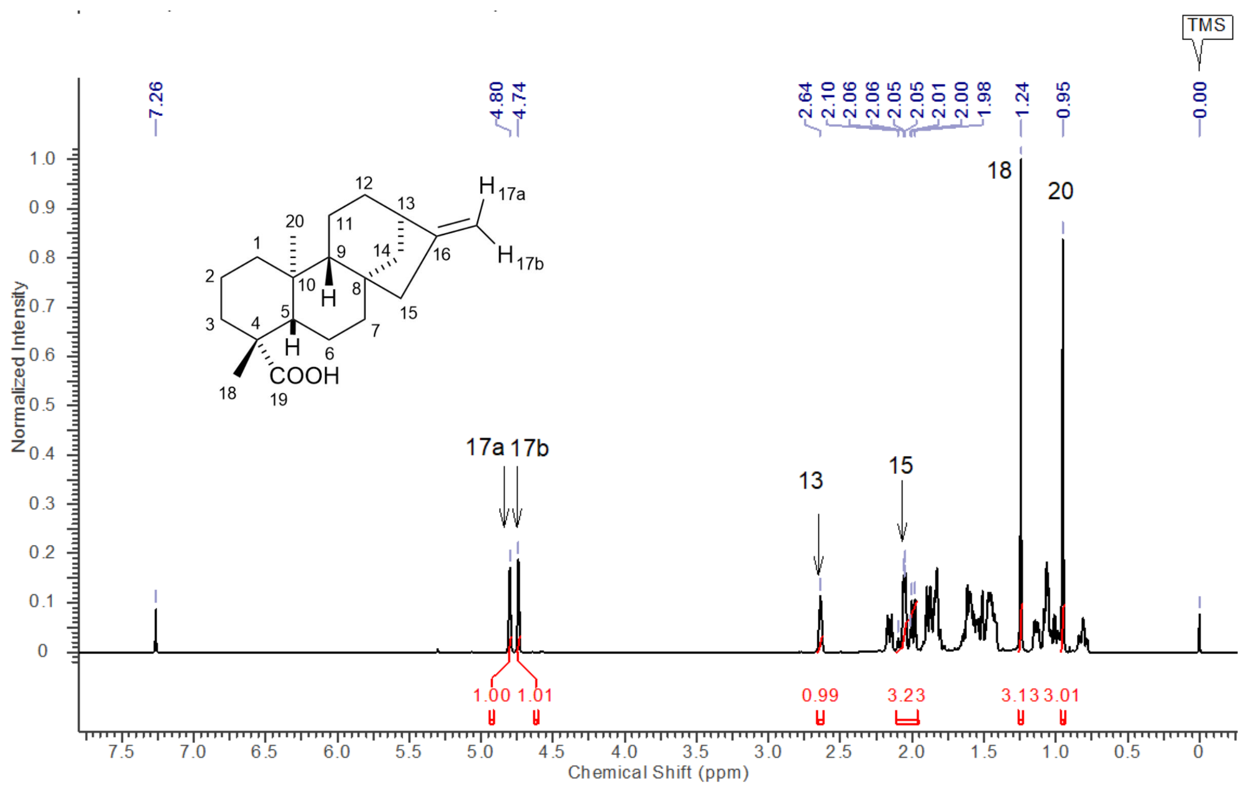

Figure S64. ^{1}H NMR spectrum of *ent*-kaurenoic acid – $CDCl_3$, 500 MHz.

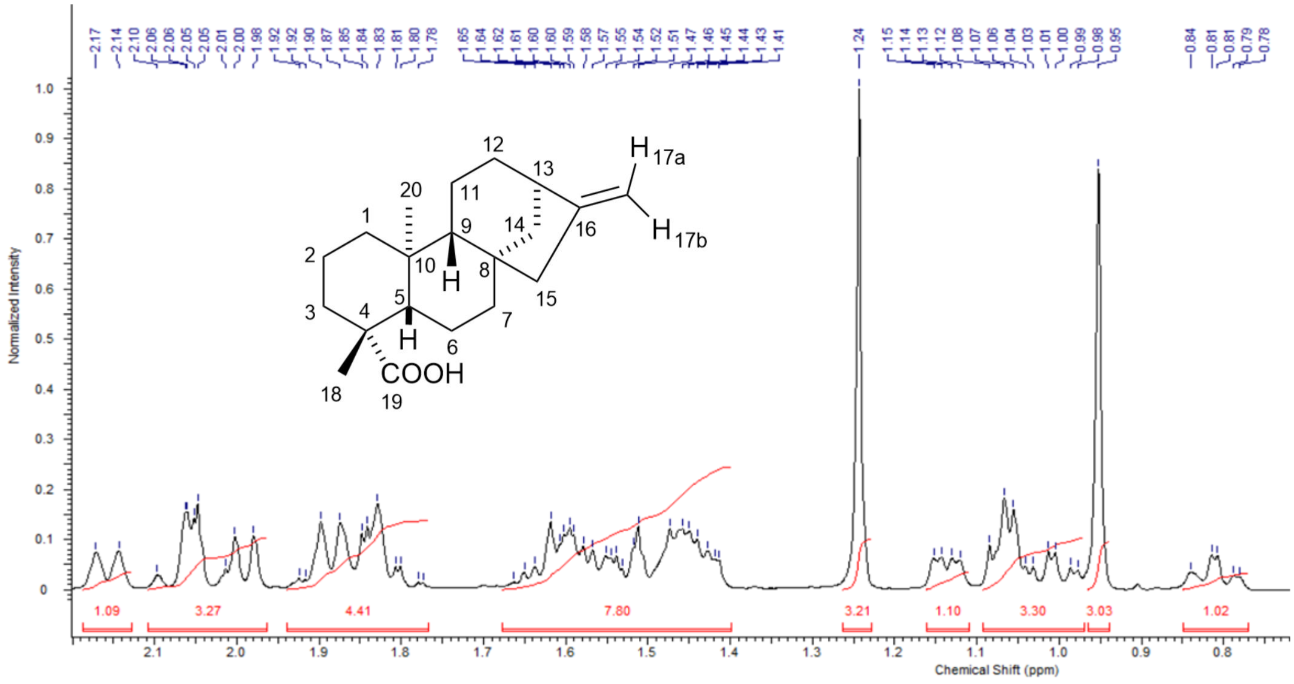

Figure S65. ^{1}H NMR spectrum of *ent*-kaurenoic acid – $CDCl_3$, 500 MHz – Expansion 1.

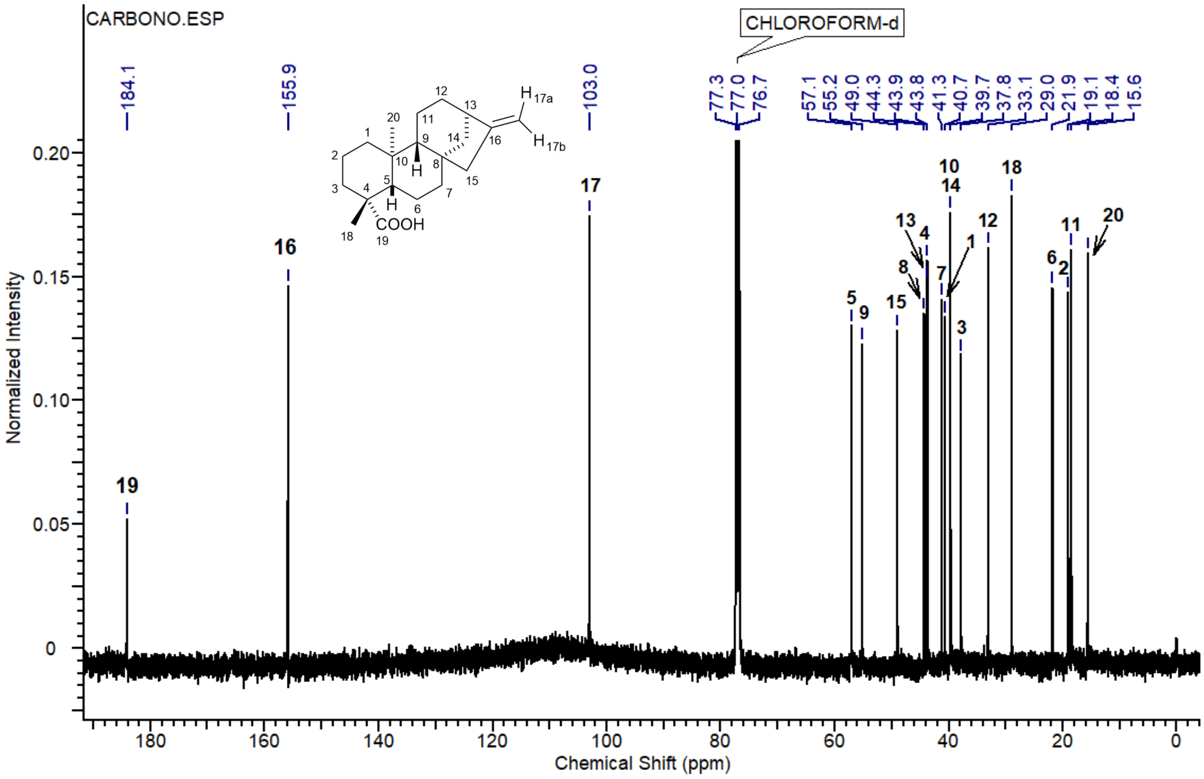

Figure S66. ^{13}C {^{1}H } NMR spectrum of *ent*-kaurenoic acid – $CDCl_3$, 125 MHz.

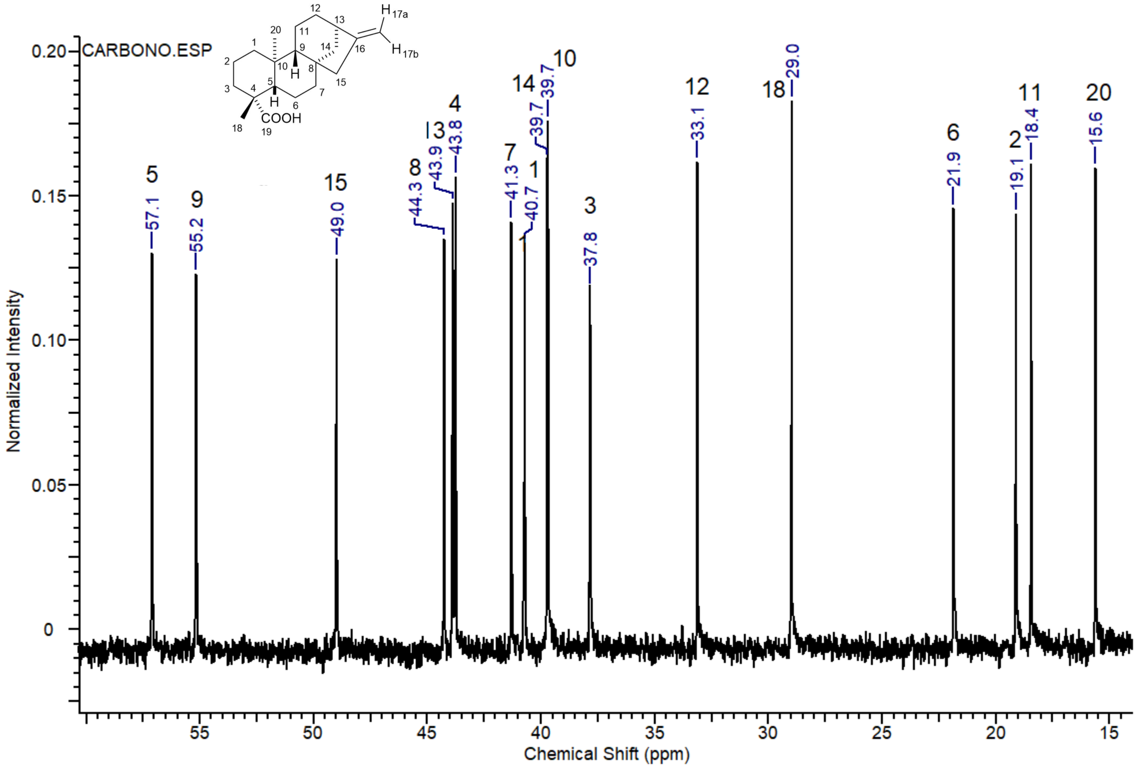

Figure S67. ^{13}C {^{1}H } NMR spectrum of *ent*-kaurenoic acid – $CDCl_3$, 125 MHz – Expansion 1.

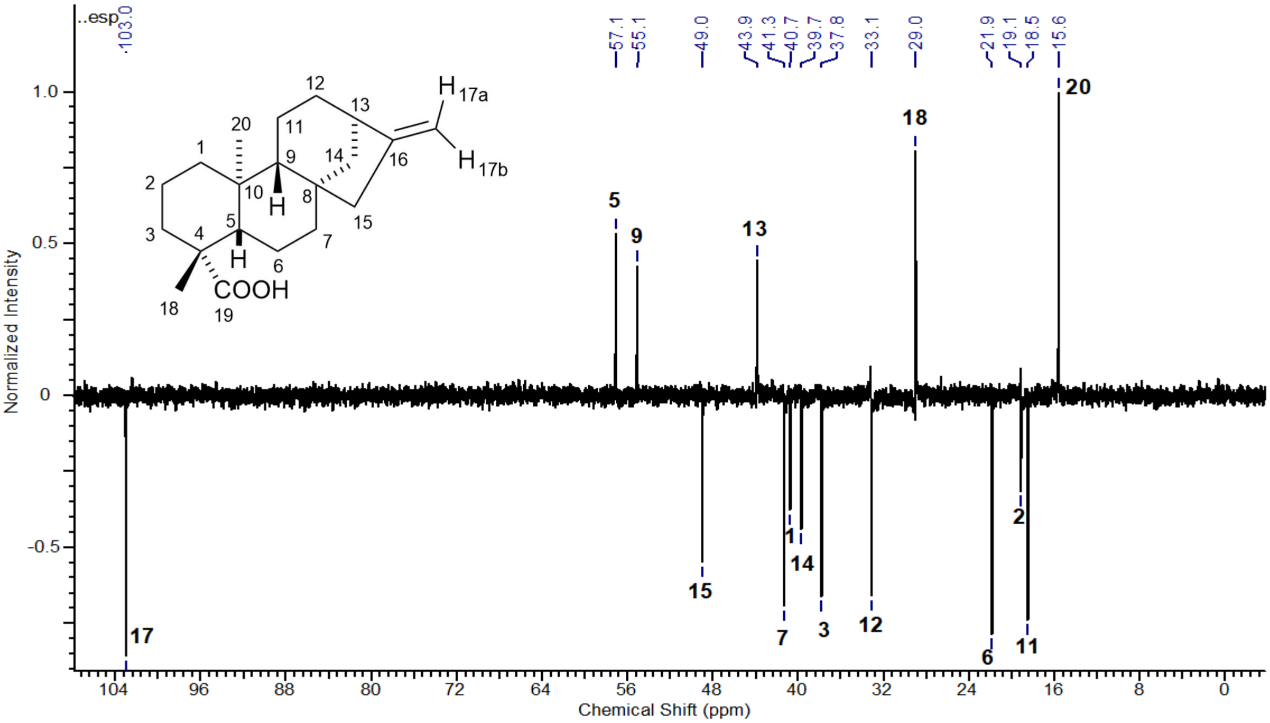

Figure S68. DEPT-135 spectrum of *ent*-kaurenoic acid – $CDCl_3$, 125 MHz.

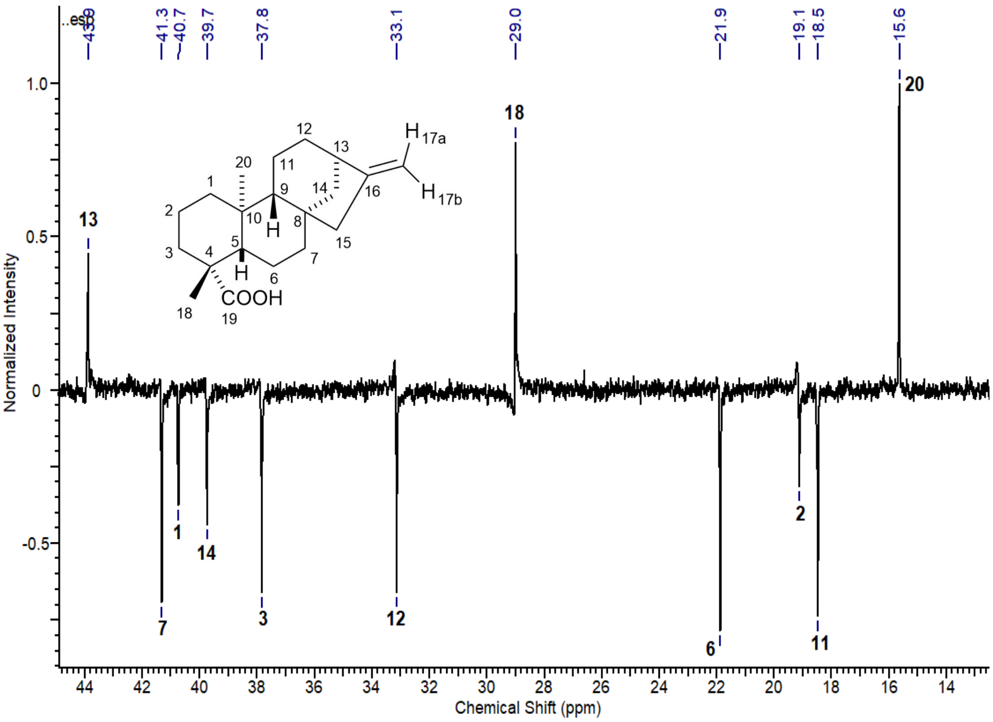

Figure S69. DEPT-135 spectrum of *ent*-kaurenoic acid – $CDCl_3$, 125 MHz – Expansion 1.

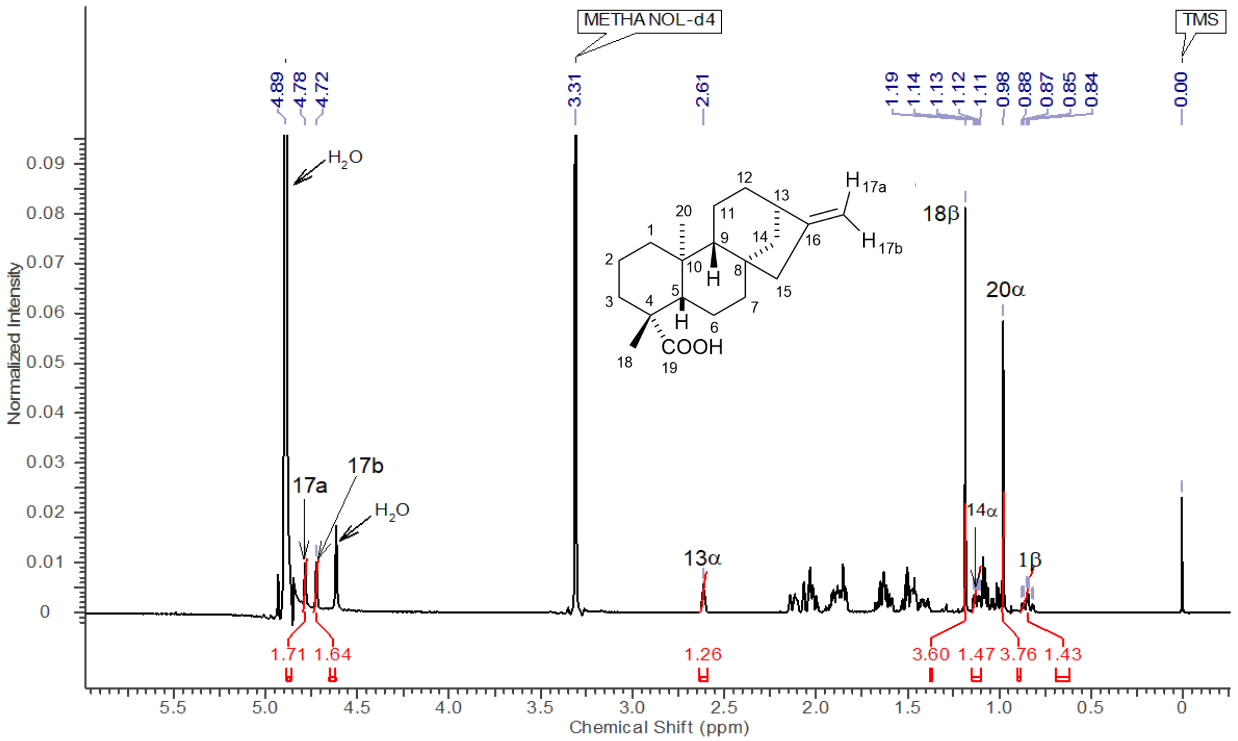

Figure S70. ^{1}H NMR spectrum of *ent*-kaurenoic acid – CD3OD, 500 MHz.

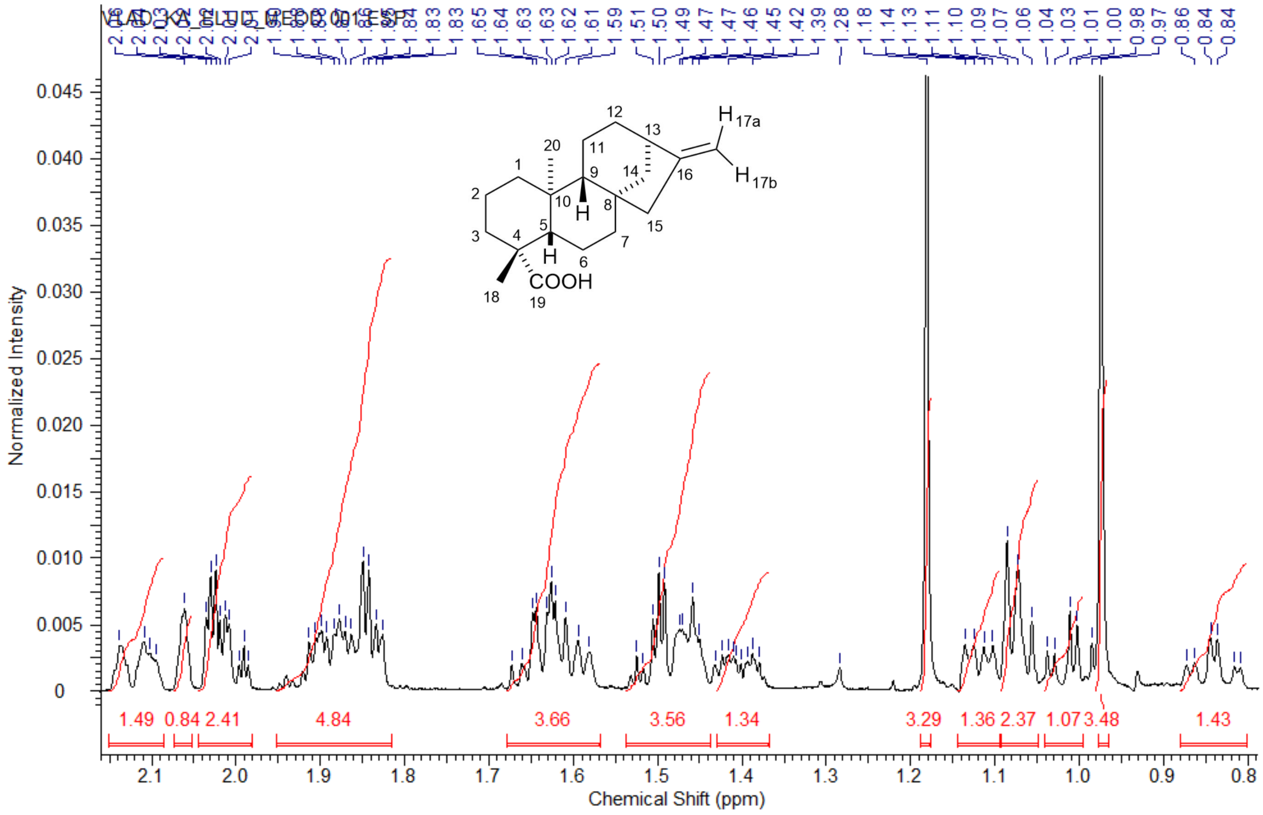

Figure S71. ^{1}H NMR spectrum of *ent*-kaurenoic acid – CD3OD, 500 MHz – Expansion 1.

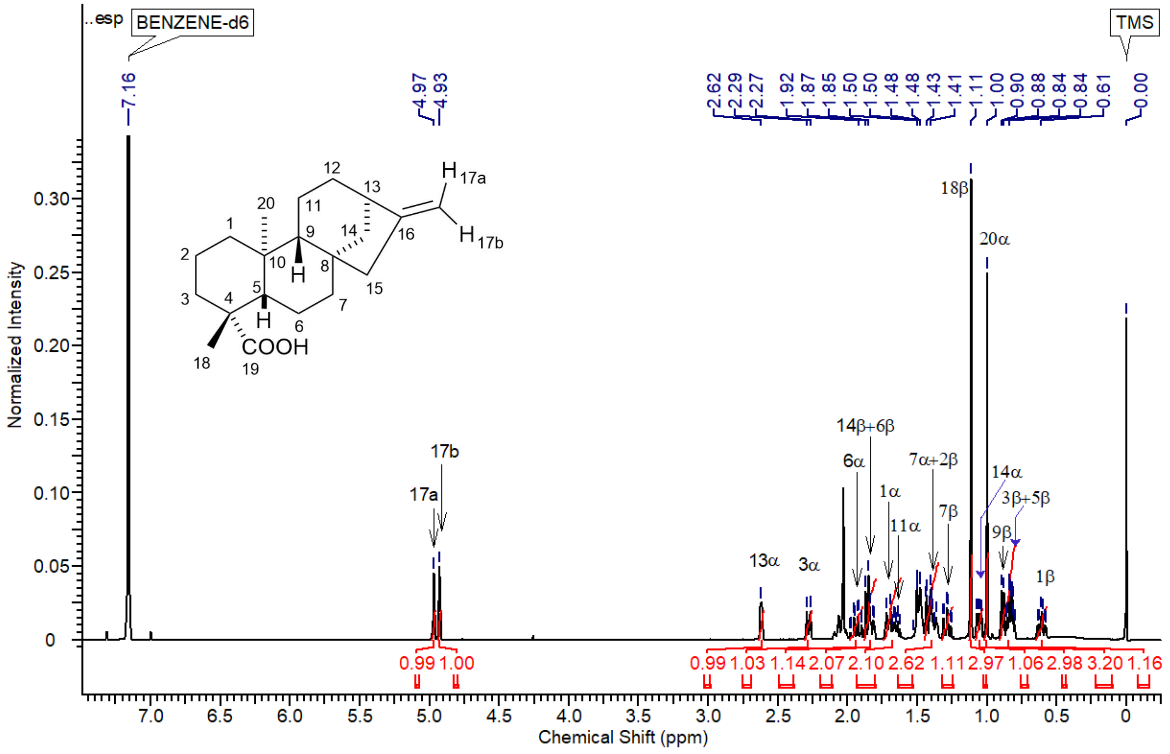

Figure S72. ^{1}H NMR spectrum of *ent*-kaurenoic acid – C_6D_6, 500 MHz.

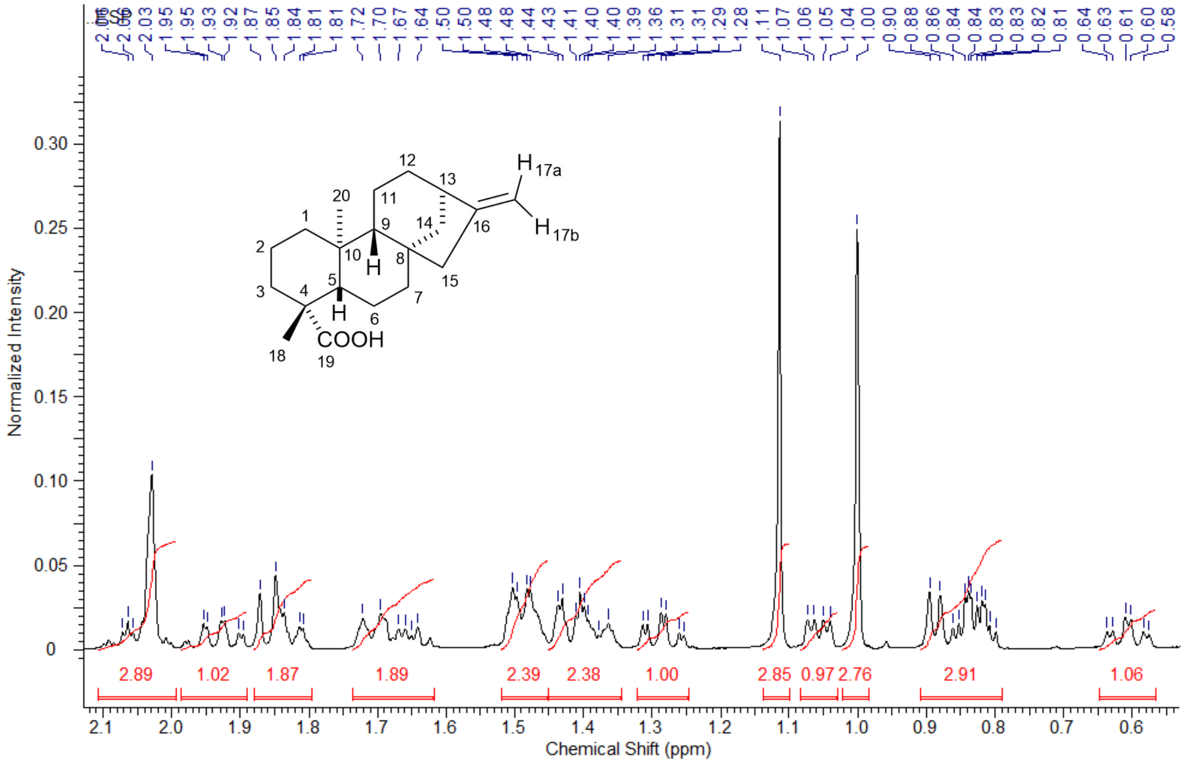

Figure S73. ^{1}H NMR spectrum of *ent*-kaurenoic acid – C_6D_6, 500 MHz - Expansion 1.

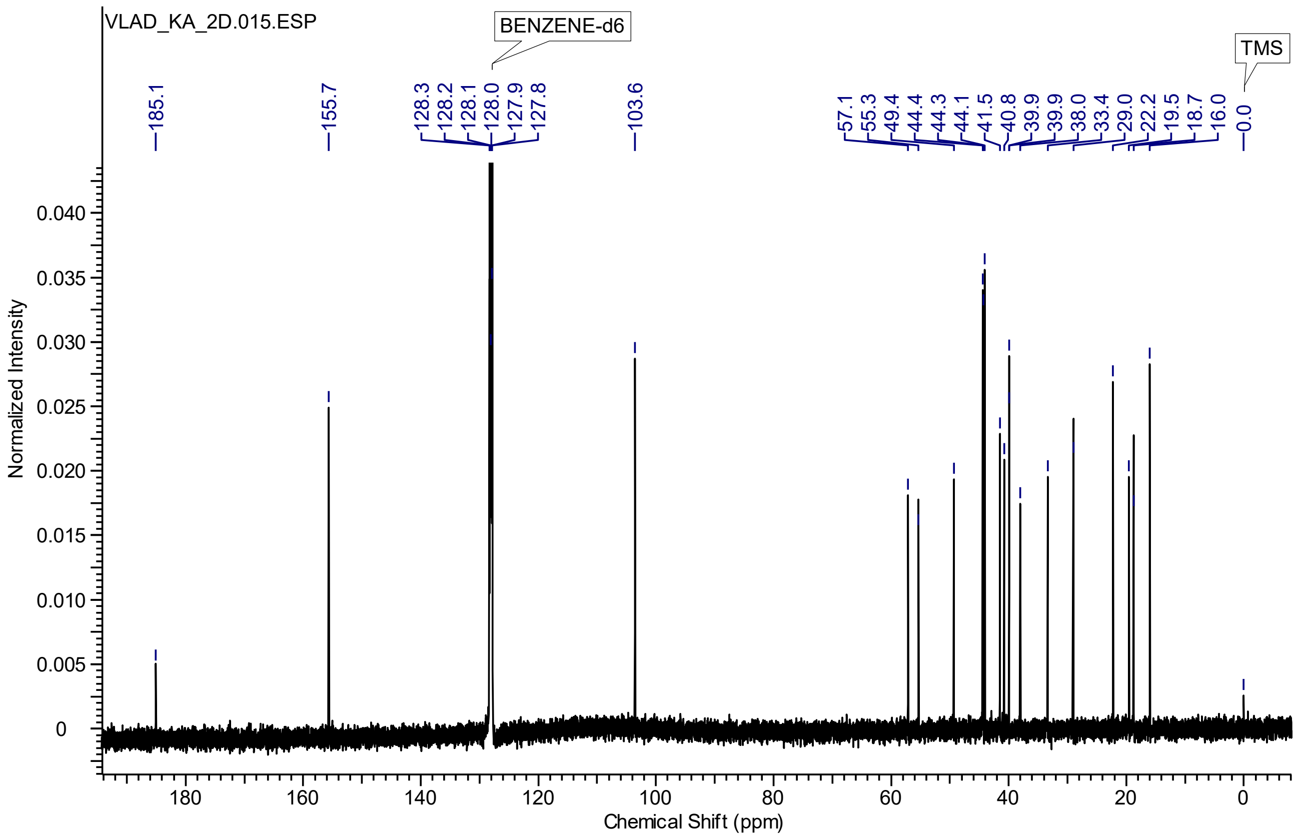

Figure S74. ^{13}C {^{1}H } NMR spectrum expansion of *ent*-kaurenoic acid – C_6D_6, 125 MHz.

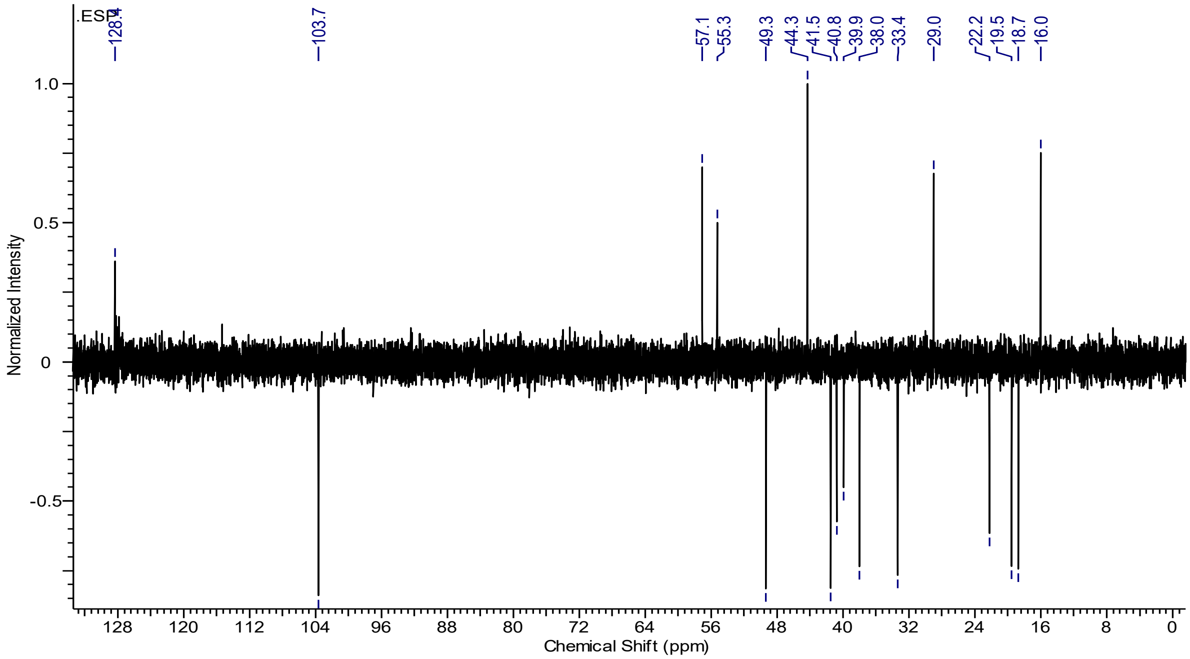

Figure S75. DEPT-135 spectrum of *ent*-kaurenoic acid – C_6D_6, 125 MHz.

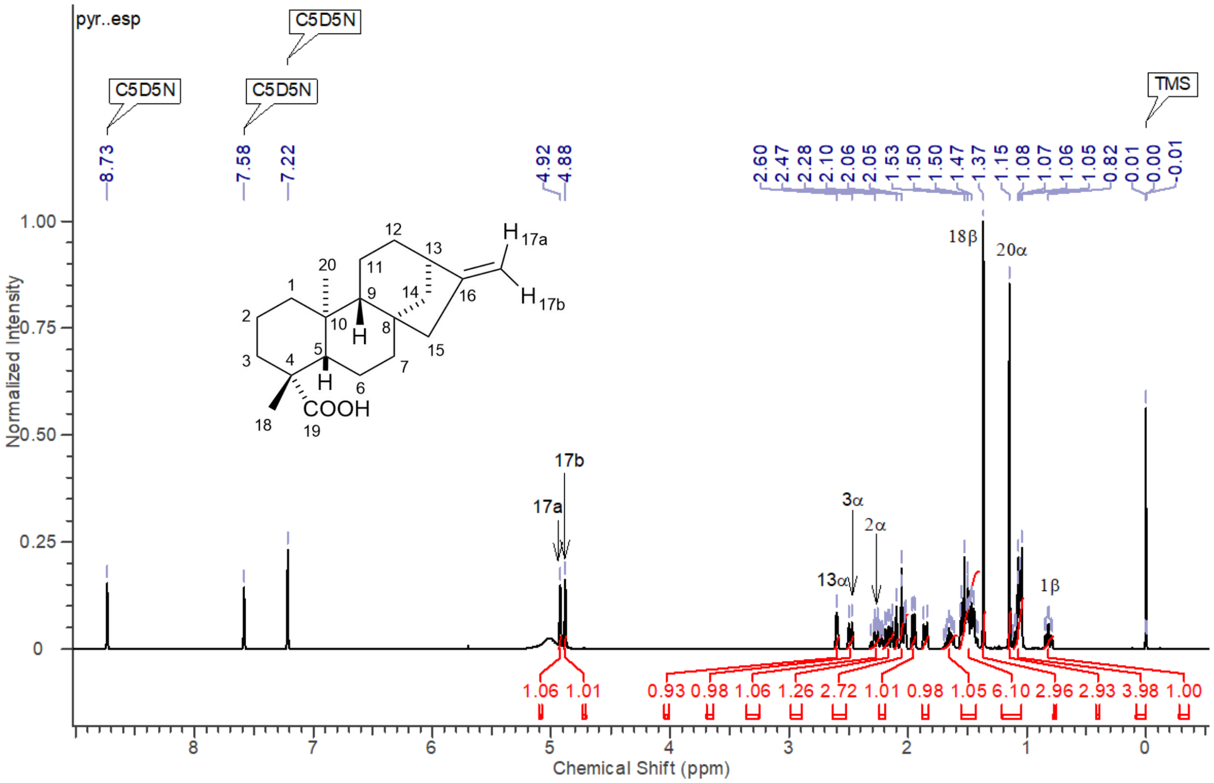

Figure S76. ^{1}H NMR spectrum of *ent*-kaurenoic acid – C_5D_5N, 500 MHz.

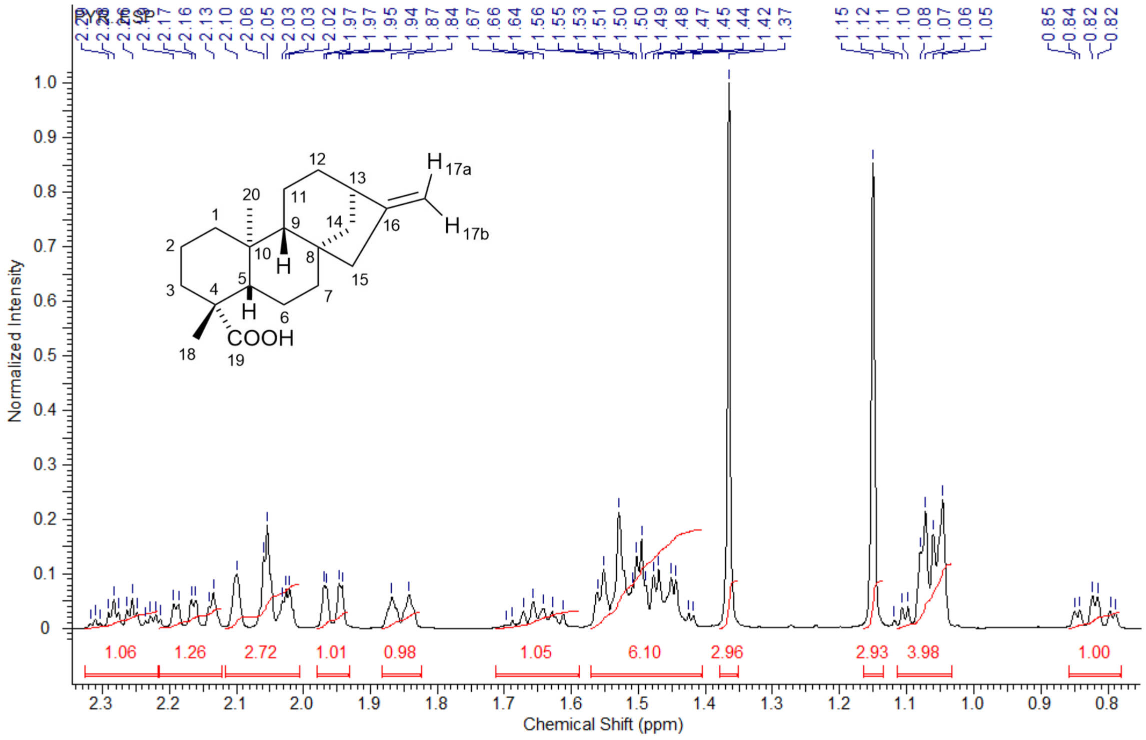

Figure S77. ^{1}H NMR spectrum of *ent*-kaurenoic acid – C_5D_5N, 500 MHz – Expansion 1.

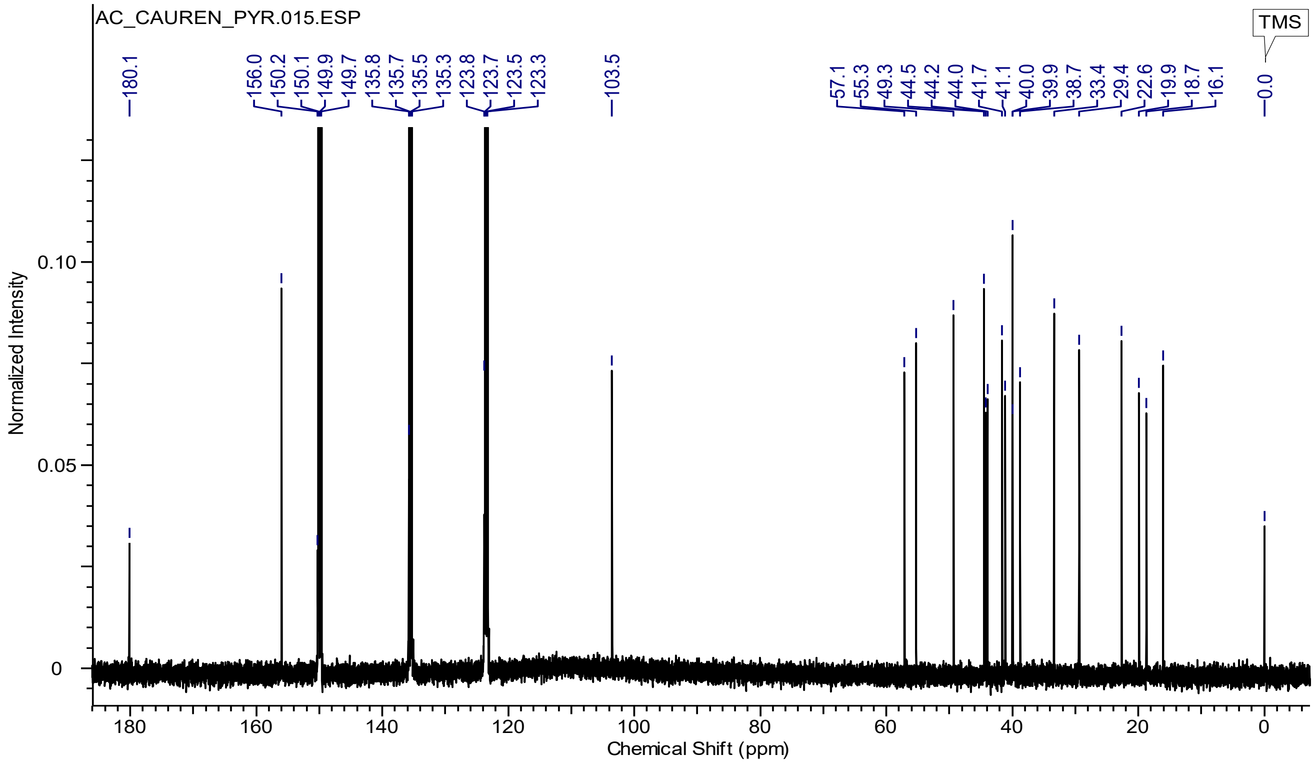

Figure S78. ^{13}C {^{1}H } NMR spectrum expansion of *ent*-kaurenoic acid – C_5D_5N, 125 MHz

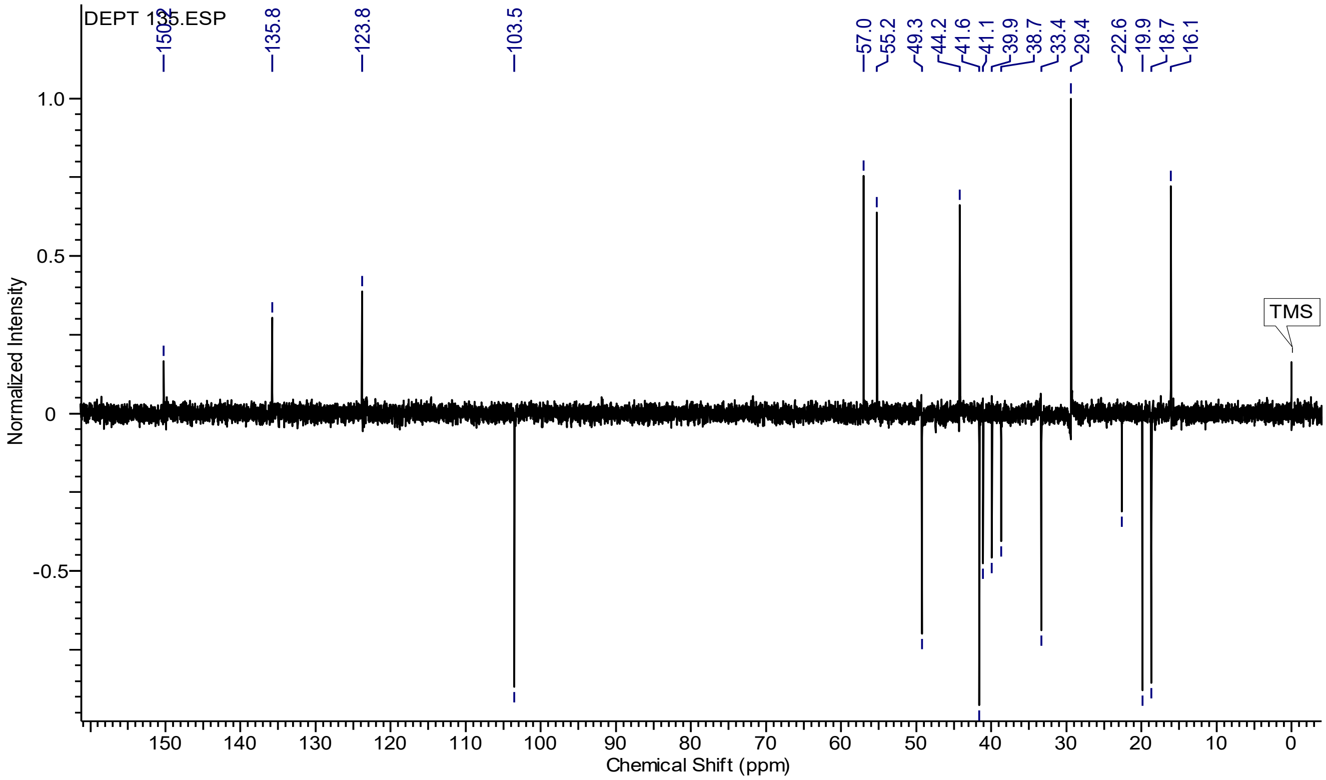

Figure S79. DEPT-135 spectrum of *ent*-kaurenoic acid – C_5D_5N, 125 MHz

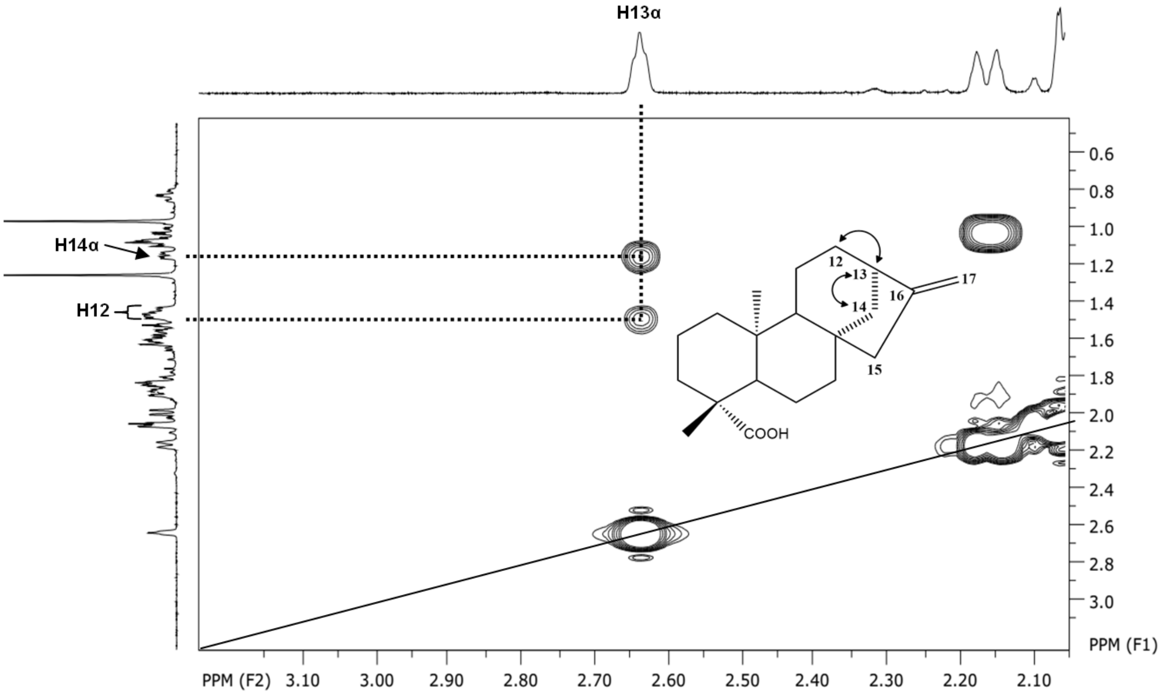

Figure S80. Correlation between H13α, H14α and H12 - COSY (^{1}H: 500 MHz) of *ent*-kaurenoic acid in $CDCl_3$.

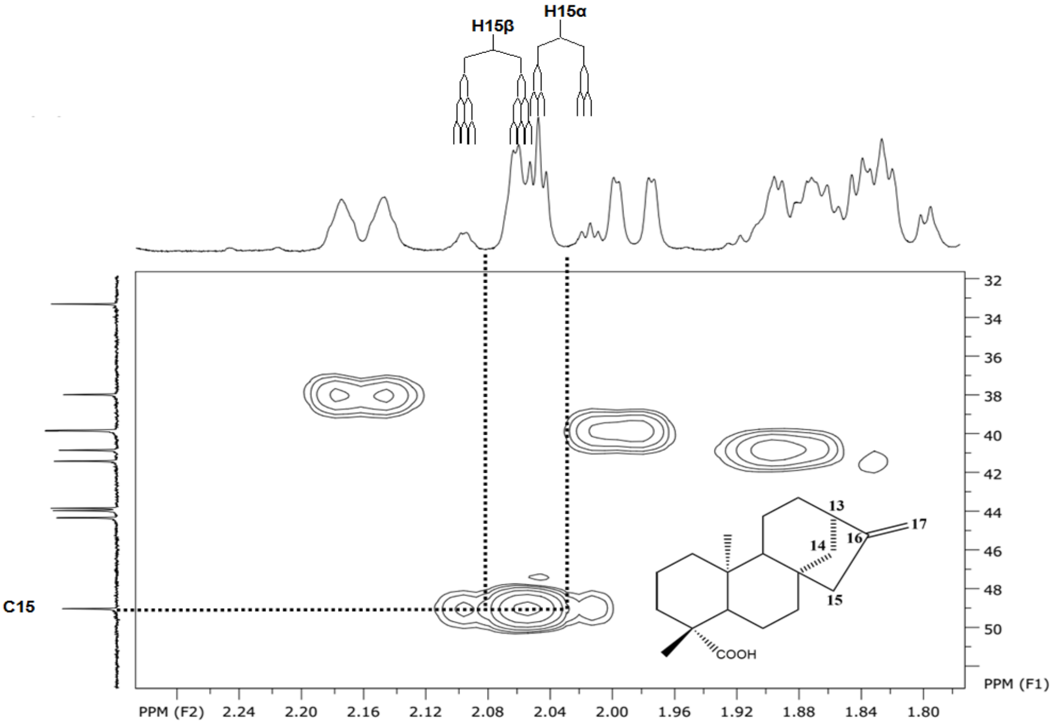

Figure S81. Spectrum expansion of HSQC (^{13}C: 125 MHz, ^{1}H: 500 MHz) correlation between C15-H15 - *ent*-kaurenoic acid in $CDCl_3$.

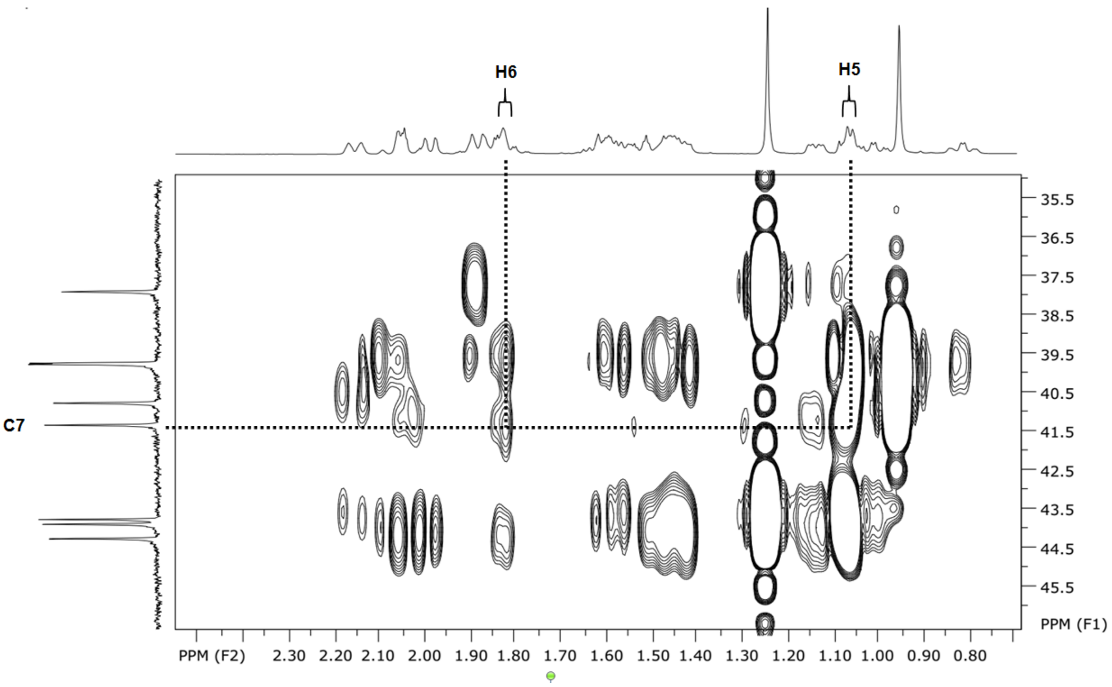

Figure S82. Correlation between C7-H6/H5 - HMBC (^{13}C: 125 MHz, ^{1}H: 500 MHz) of *ent*-kaurenoic acid in $CDCl_3$.

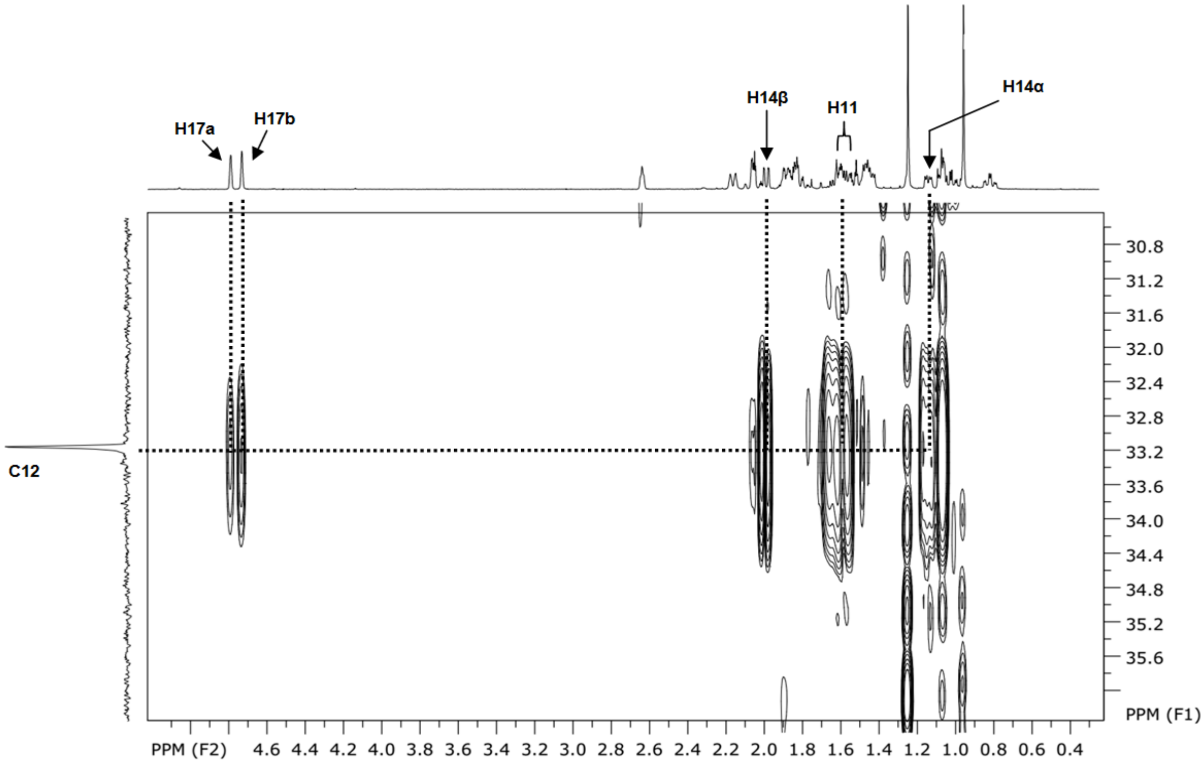

Figure S83. Correlation between C12-H17/H14/H11 - HMBC (^{13}C: 125 MHz, ^{1}H: 500 MHz) of *ent*-kaurenoic acid in $CDCl_3$.

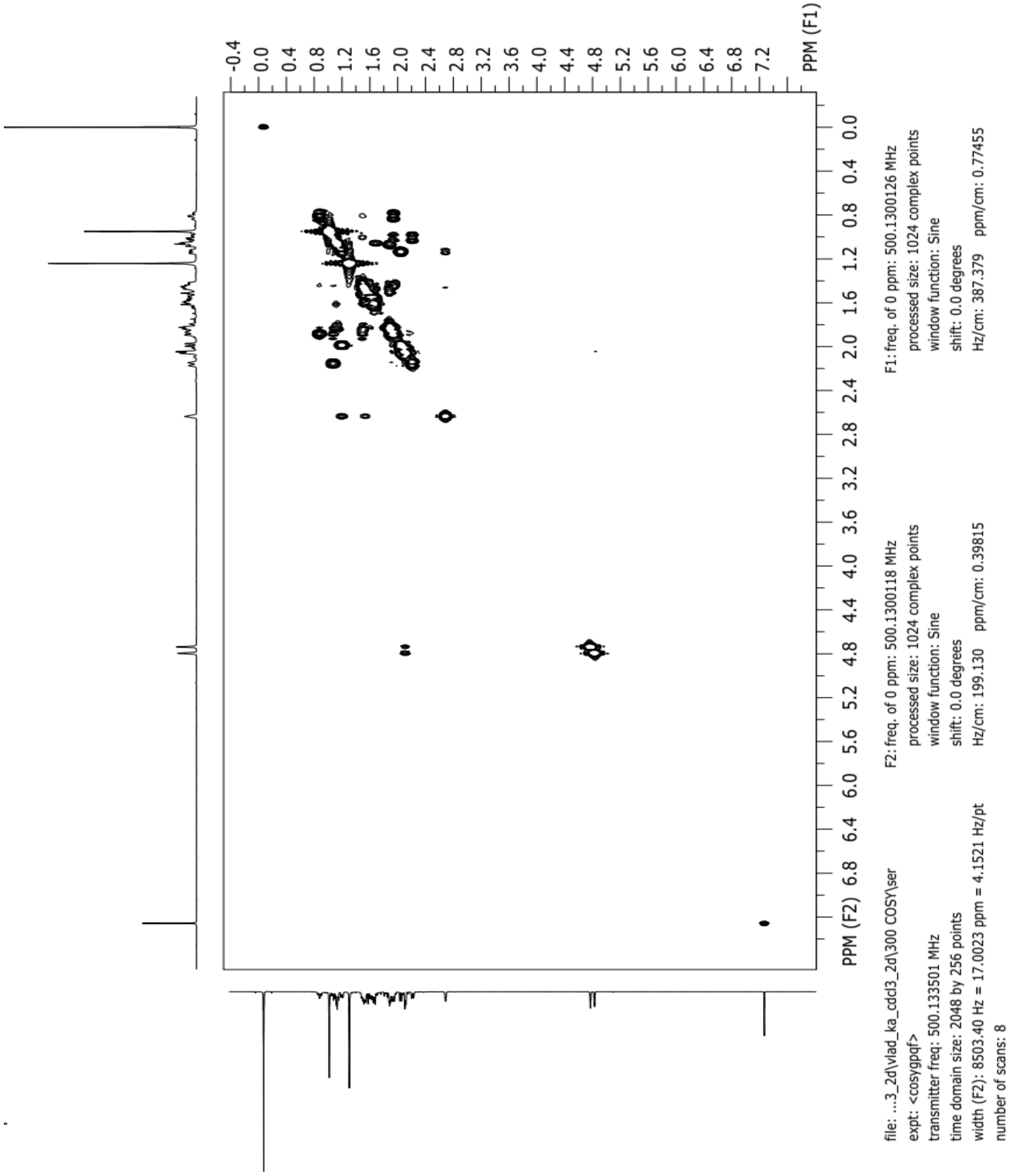

Figure S84. COSY spectrum of *ent*-kaurenoic acid - $CDCl_3$ (^1H: 500 MHz).

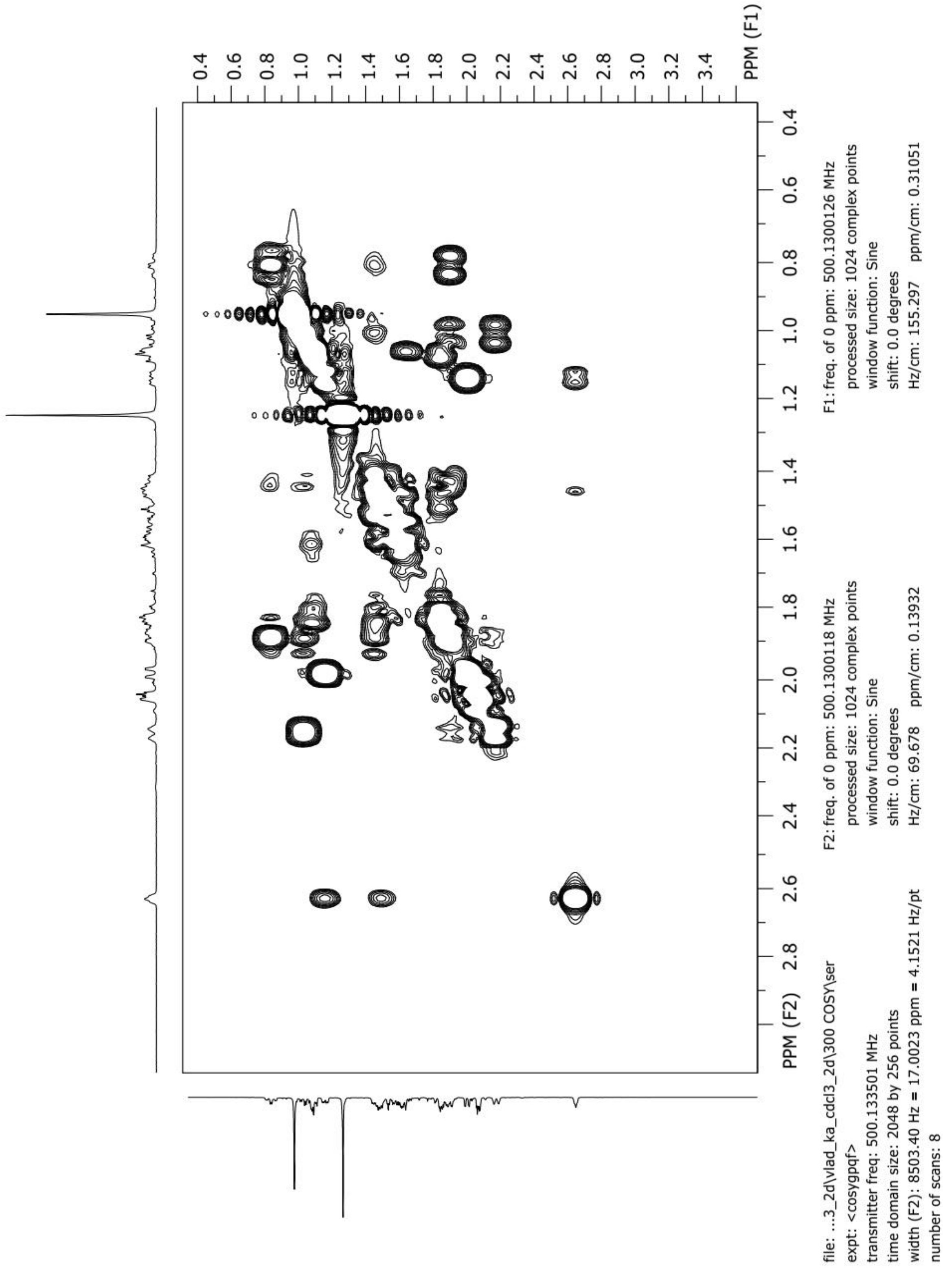

Figure S85. COSY expansion spectrum of *ent*-kaurenoic acid - $CDCl_3$ (500 MHz).

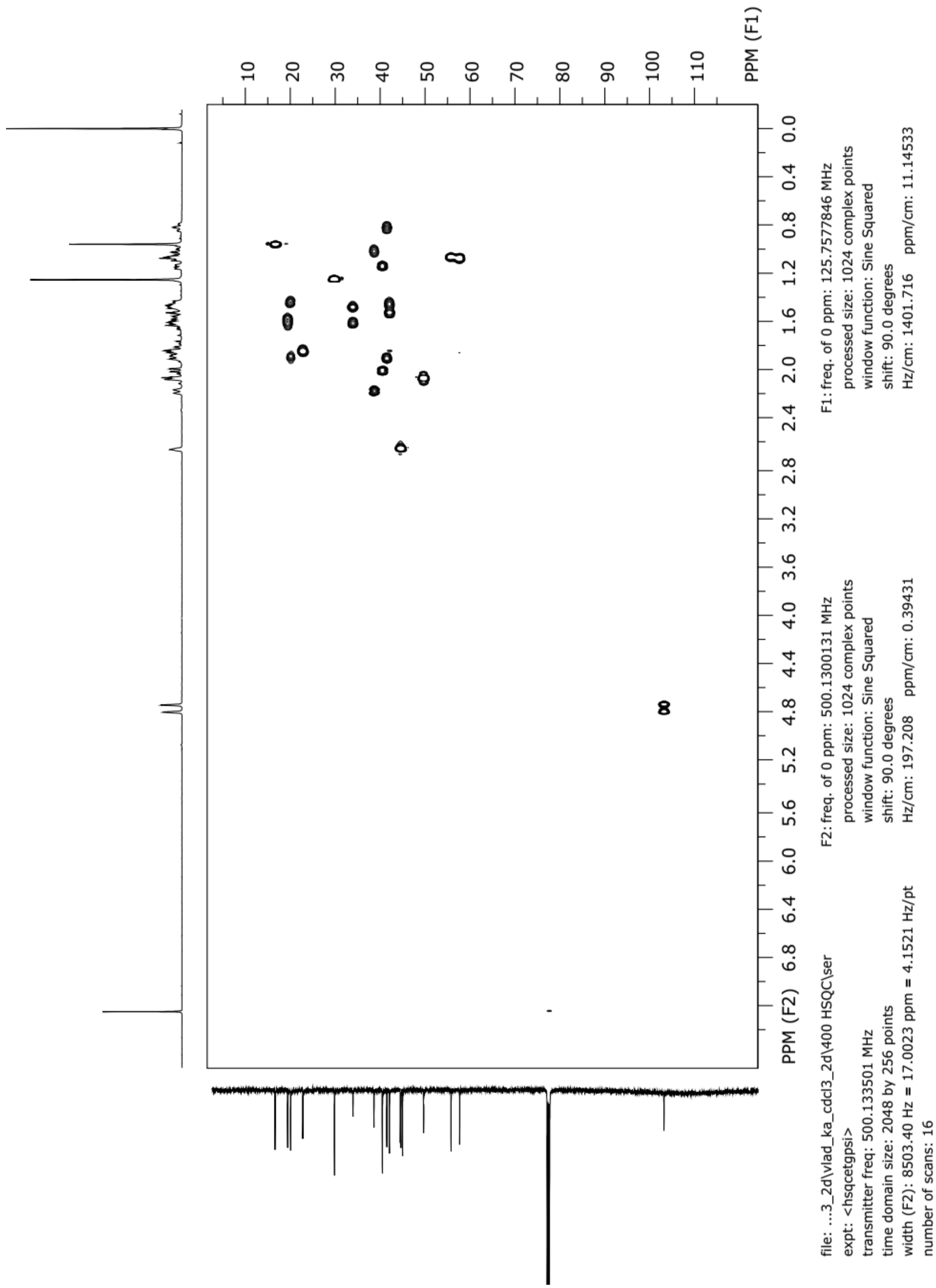

Figure S86. HSQC spectrum of *ent*-kaurenoic acid - $CDCl_3$ (^{13}C: 125 MHz, ^{1}H: 500 MHz).

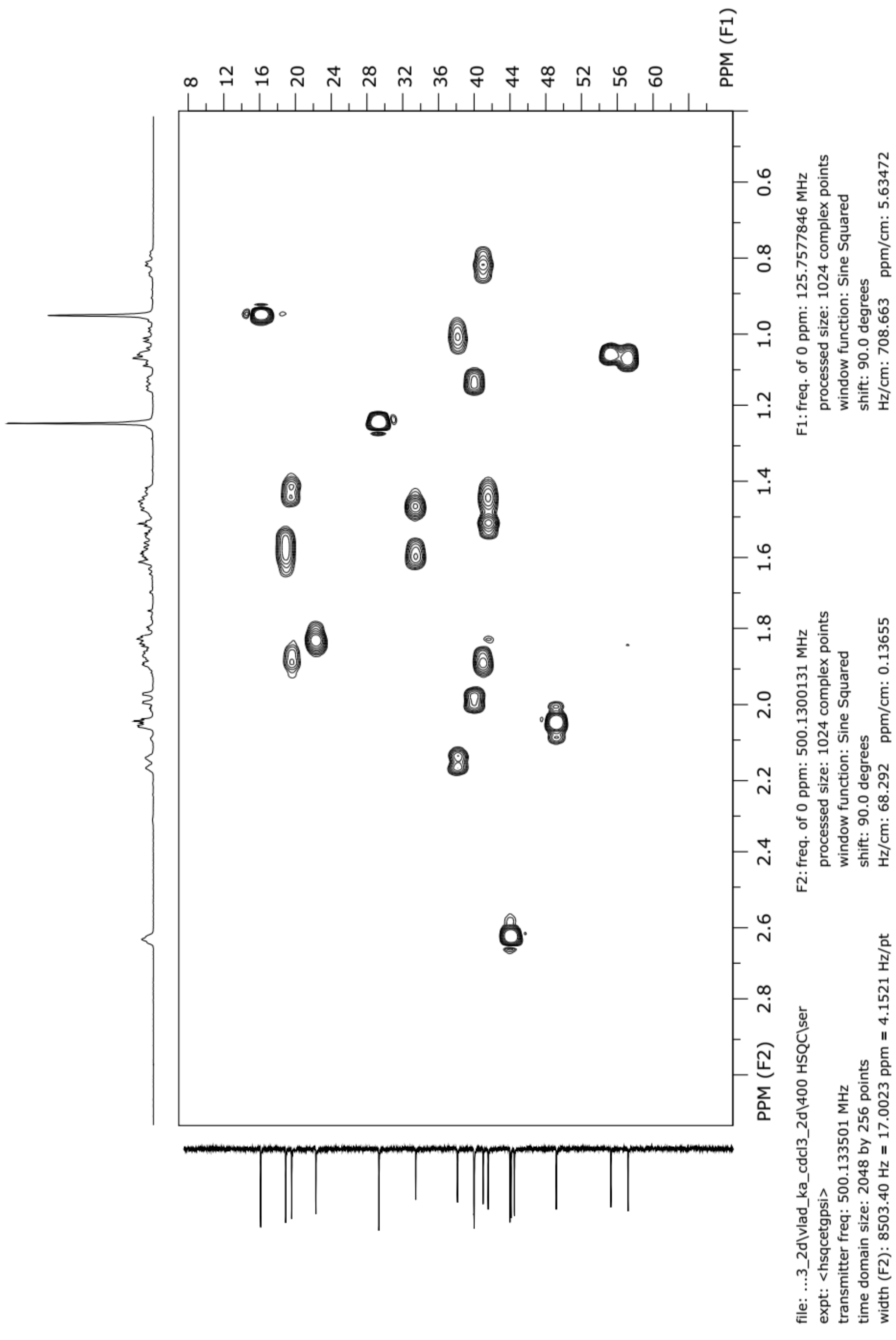

Figure S87. HSQC expansion spectrum of *ent*-kaurenoic acid - $CDCl_3$ (^{13}C: 125 MHz, ^{1}H: 500 MHz).

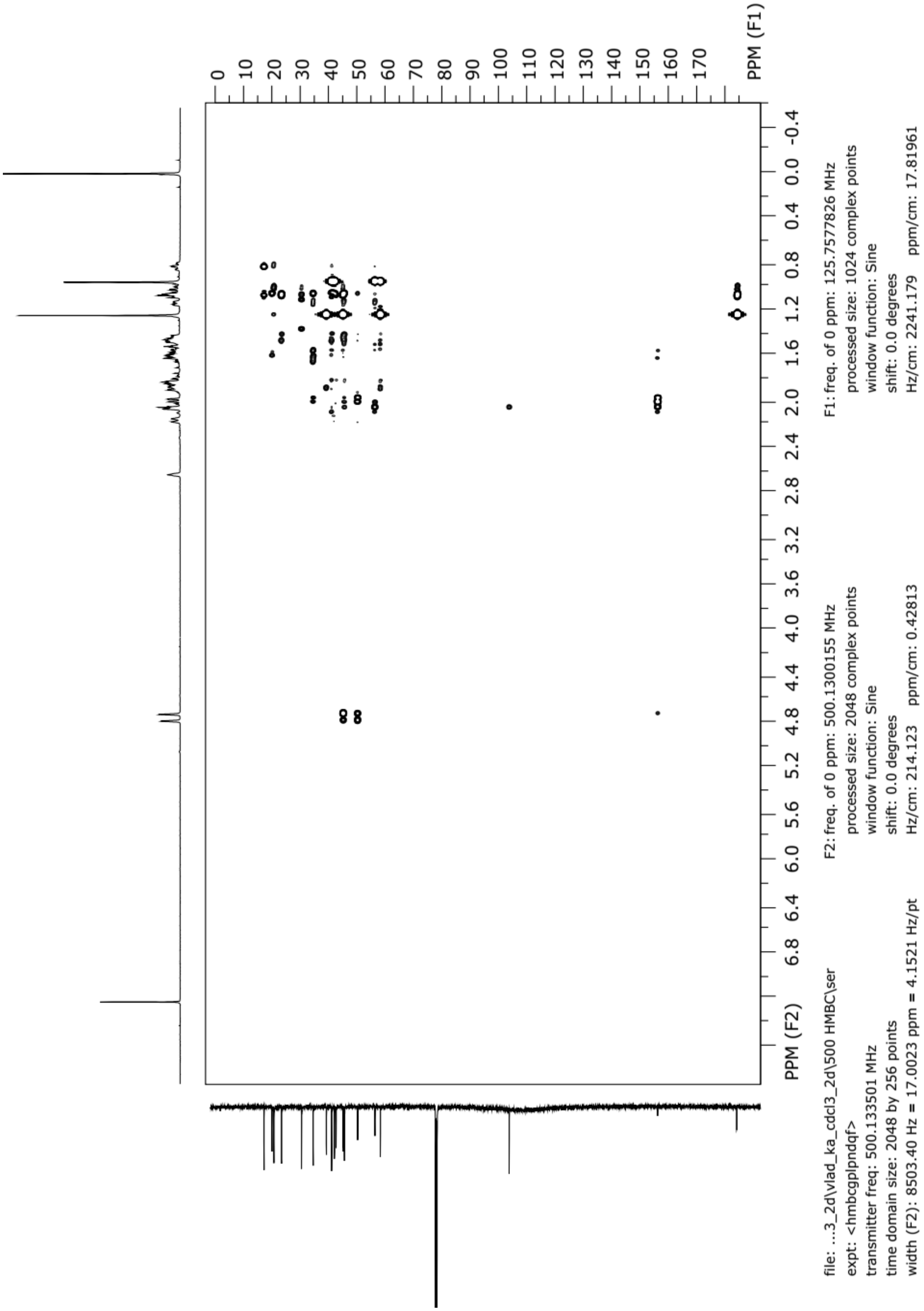

Figure S88. HMBC spectrum of *ent*-kaurenoic acid - $CDCl_3$ (^{13}C: 125 MHz, ^{1}H: 500 MHz).

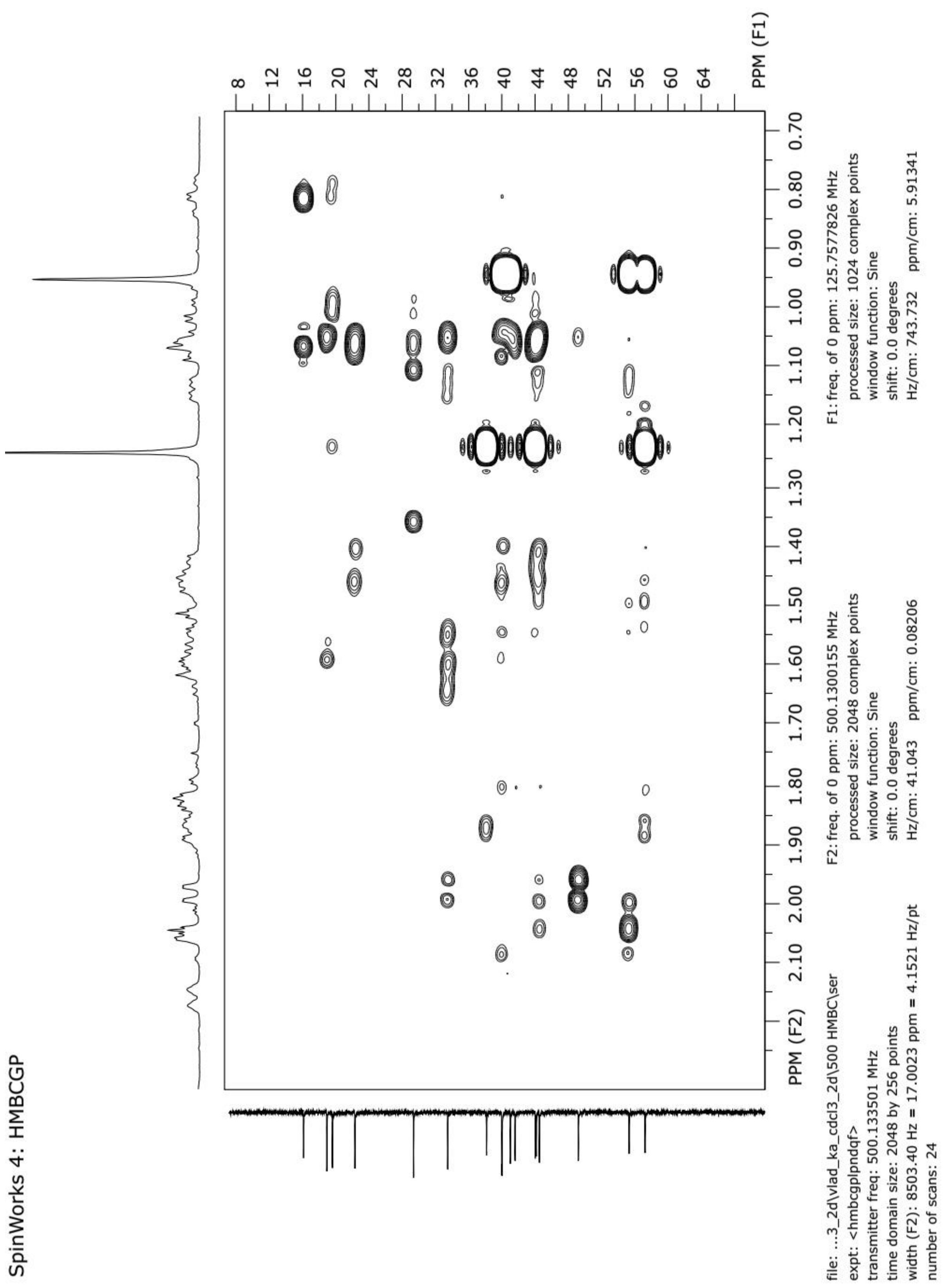

Figure S89. HMBC expansion spectrum of *ent*-kaurenoic acid - $CDCl_3$ (^{13}C: 125 MHz, ^{1}H: 500 MHz).

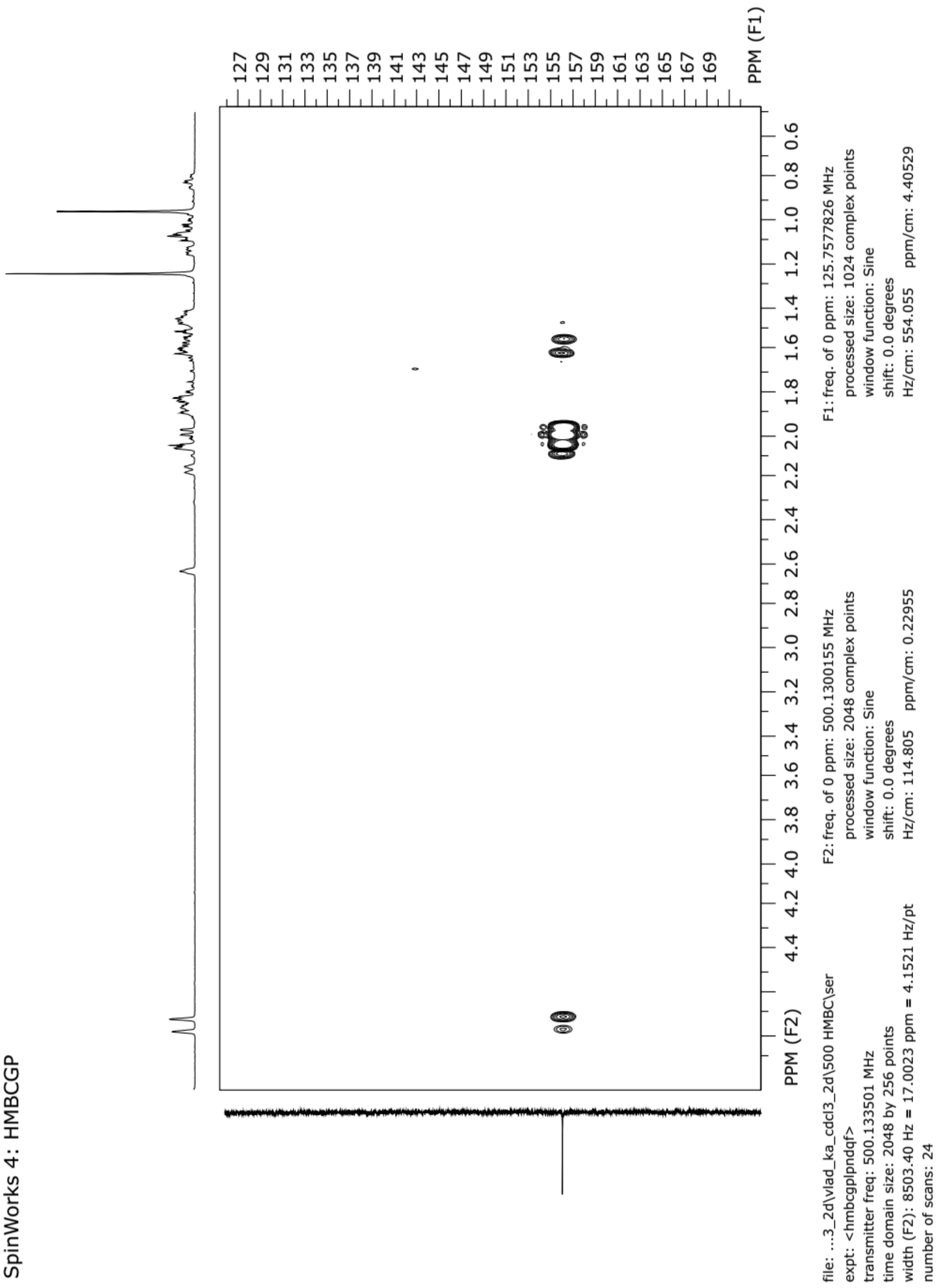

Figure S90. HMBC expansion spectrum of *ent*-kaurenoic acid - $CDCl_3$ (^{13}C: 125 MHz, ^{1}H: 500 MHz).

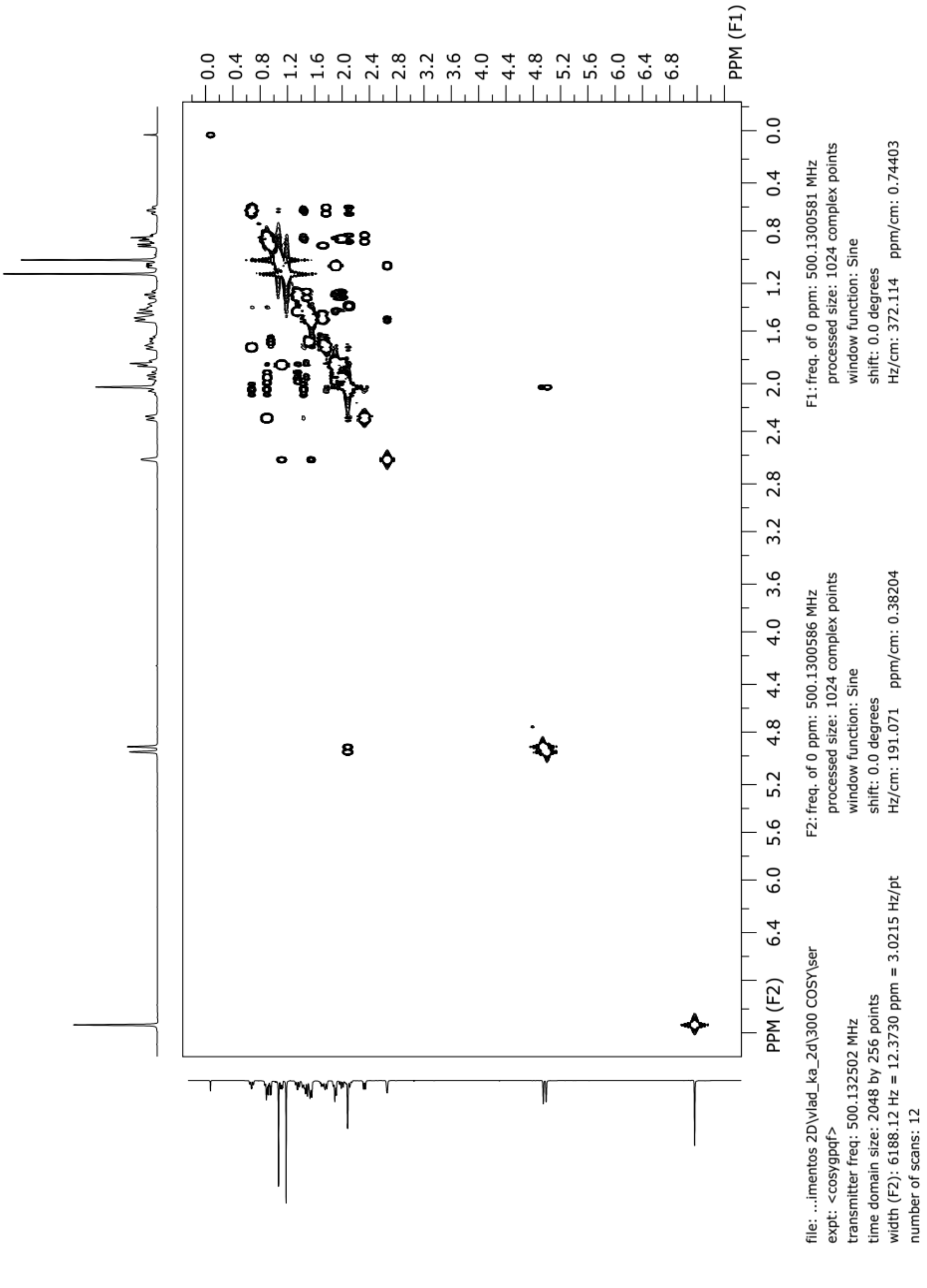

Figure S91. COSY spectrum of *ent*-kaurenoic acid – C_6D_6 (500 MHz).

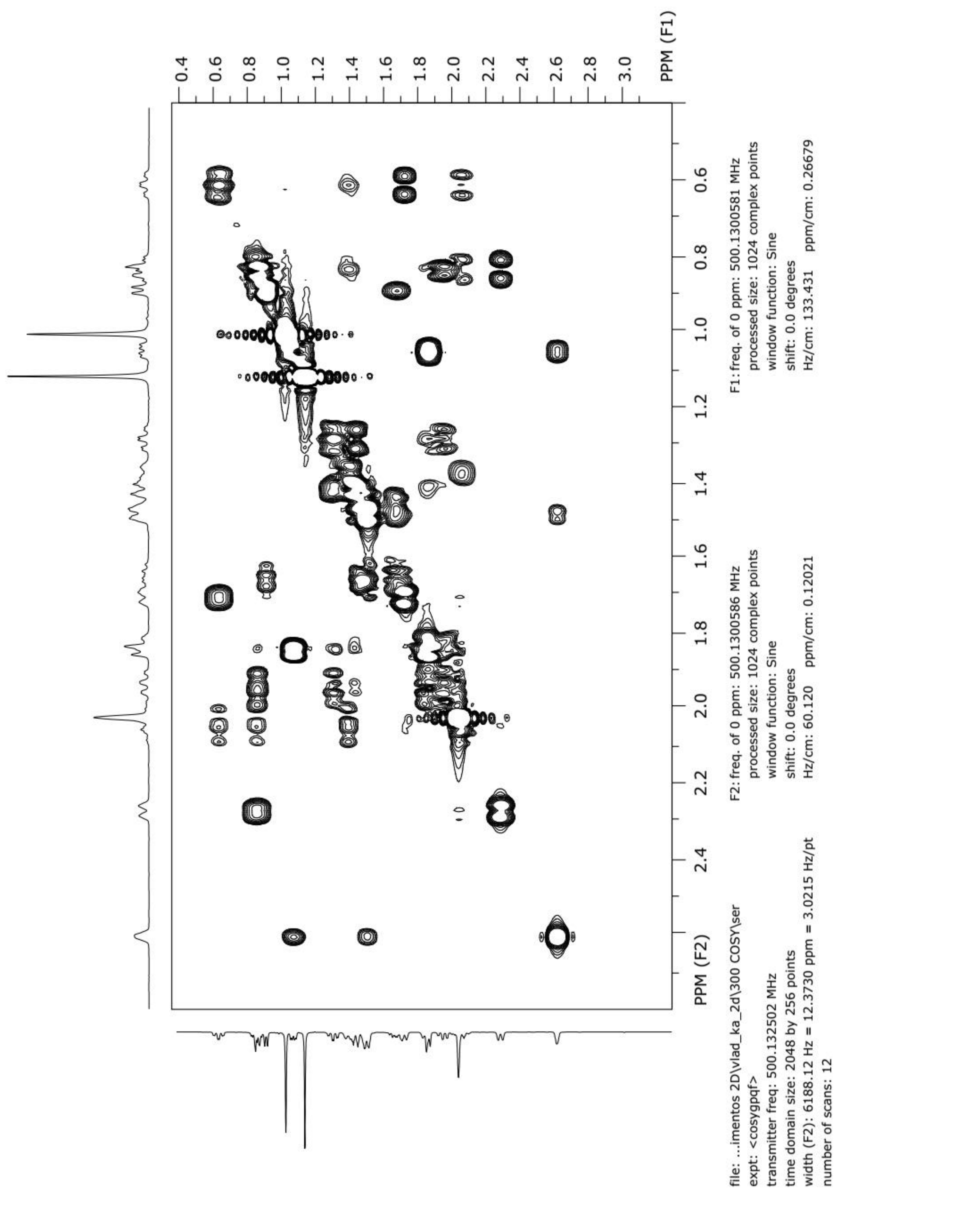

Figure S92. COSY expansion spectrum of *ent*-kaurenoic acid – C_6D_6 (500 MHz).

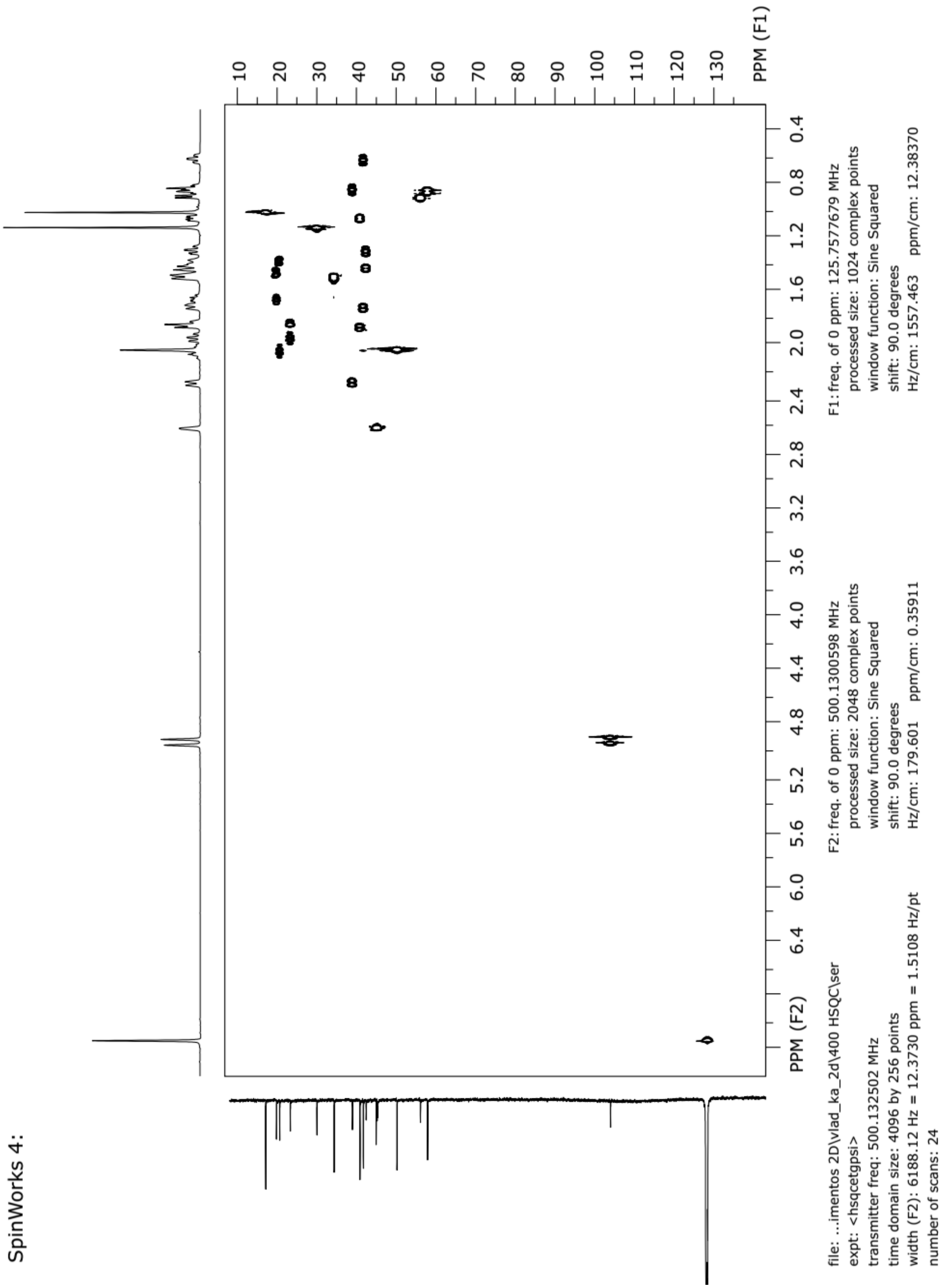

Figure S93. HSQC spectrum of *ent*-kaurenoic acid – C_6D_6 (^{13}C: 125 MHz, ^{1}H: 500 MHz).

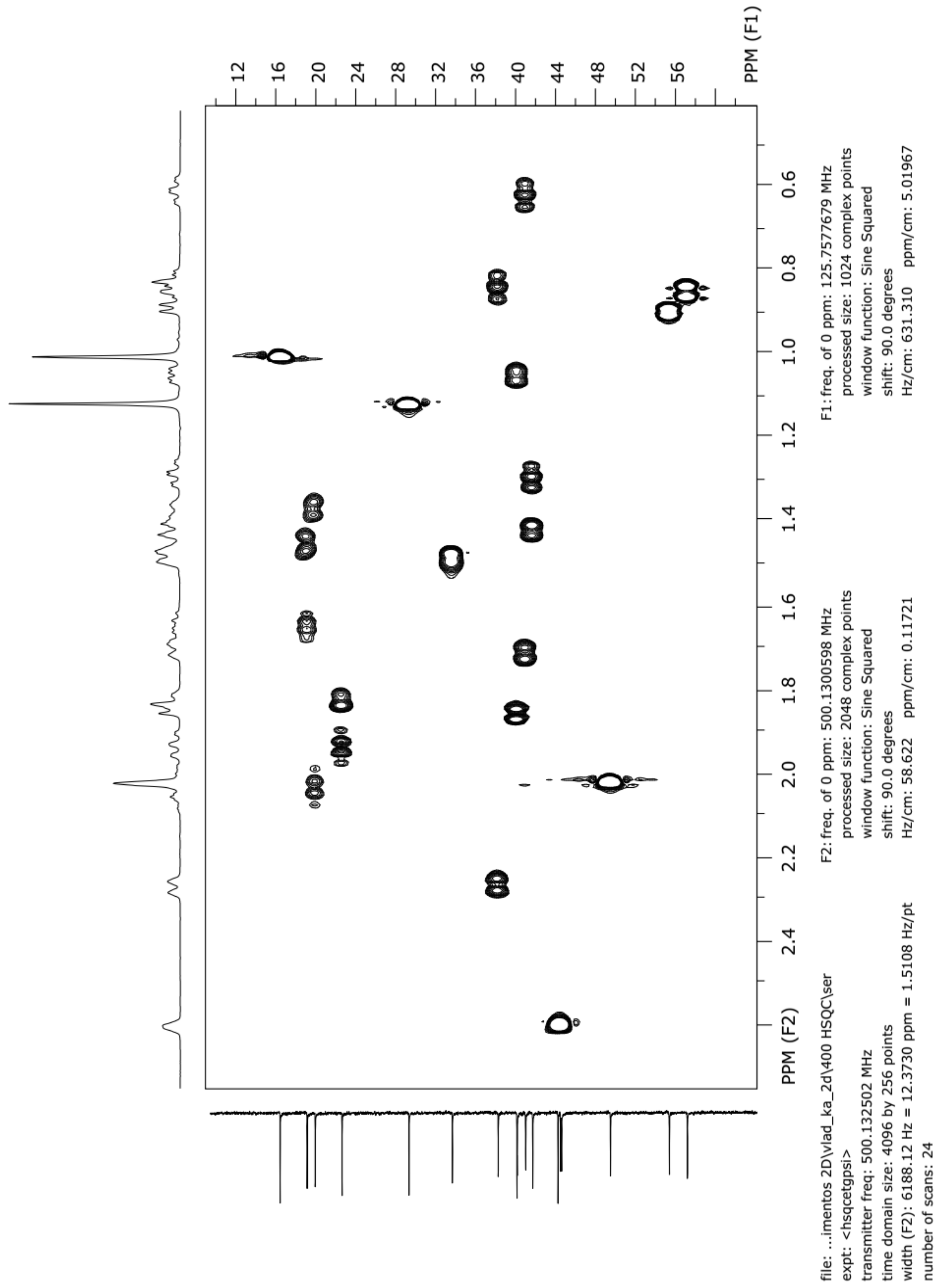

Figure S94. HSQC expansion spectrum of *ent*-kaurenoic acid – C_6D_6 (^{13}C: 125 MHz, ^{1}H: 500 MHz).

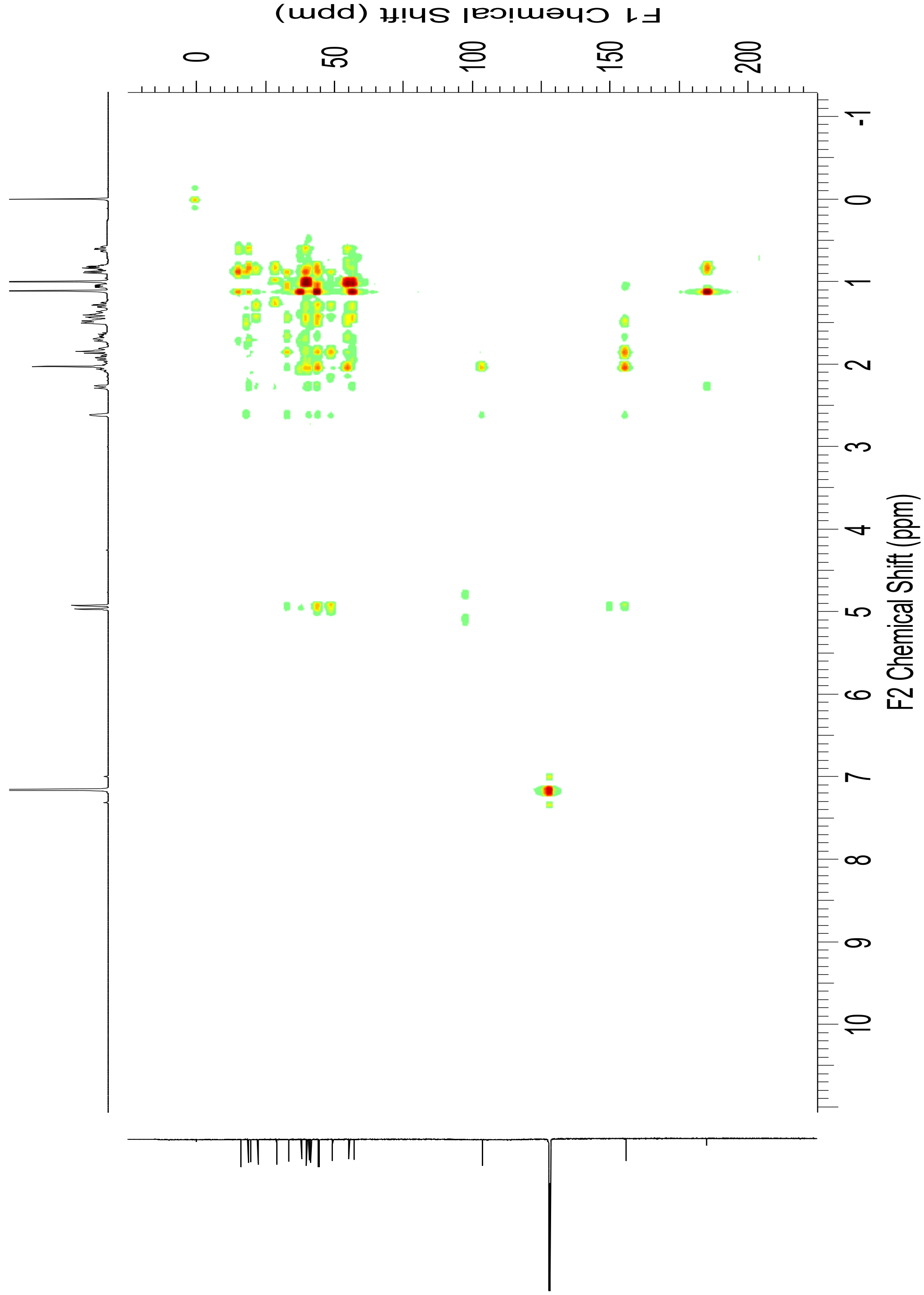

Figure S95. HMBC spectrum of *ent*-kaurenoic acid – C_6D_6 (^{13}C: 125 MHz, ^{1}H: 500 MHz).

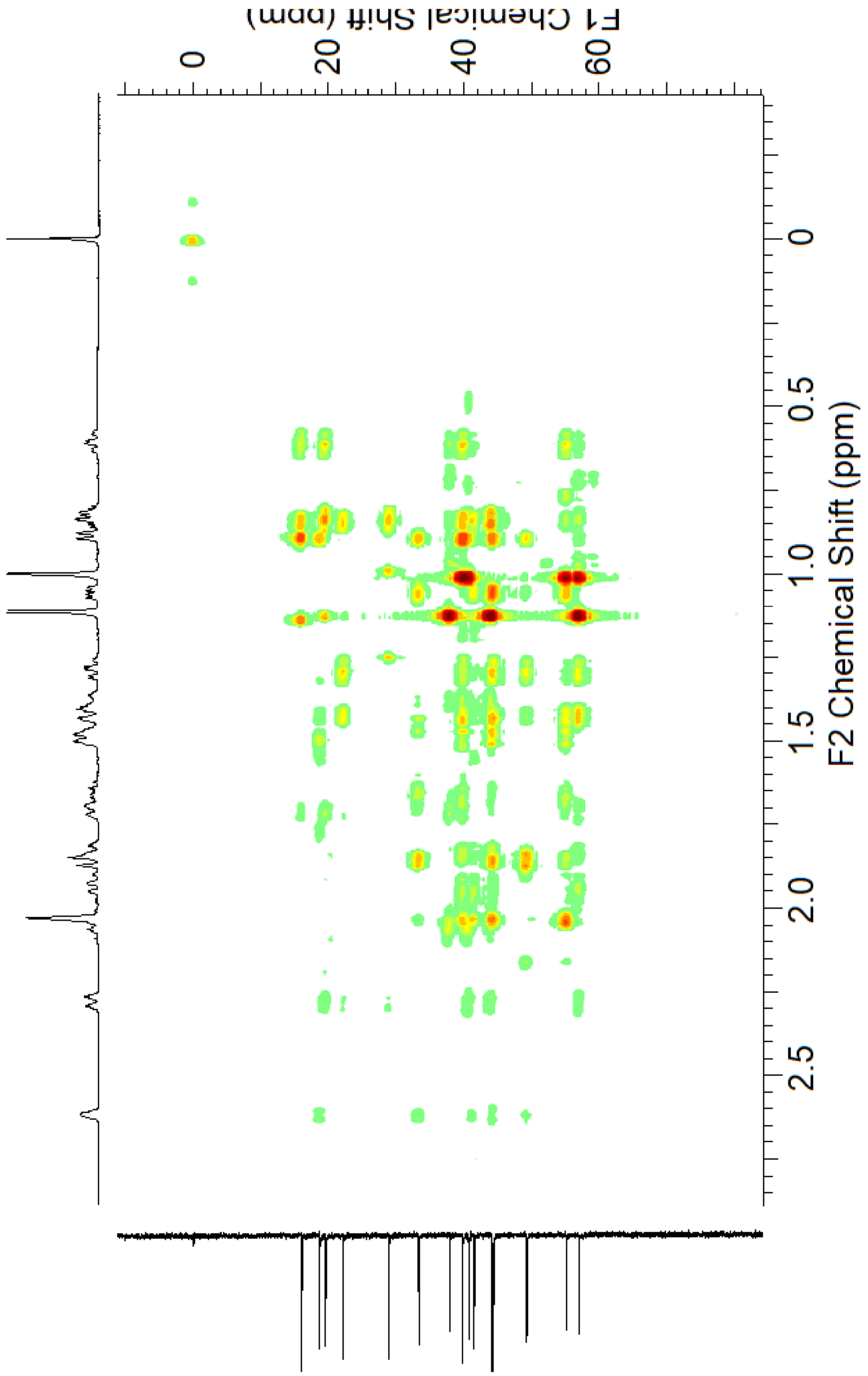

Figure S96. HMBC expansion spectrum of *ent*-kaurenoic acid – C_6D_6 (^{13}C: 125 MHz, ^{1}H: 500 MHz).

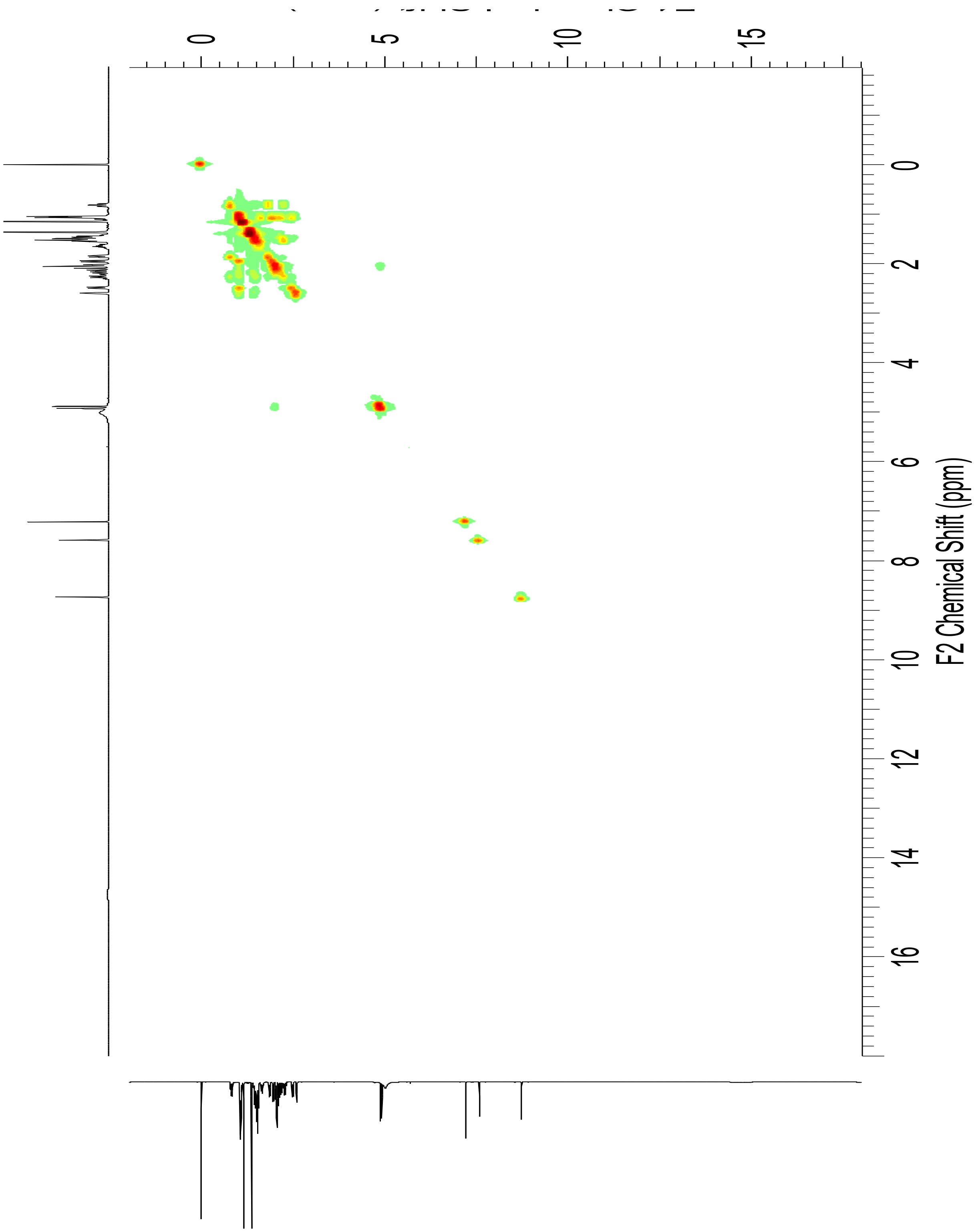

Figure S97. COSY spectrum of *ent*-kaurenoic acid – C_5D_5N (500 MHz).

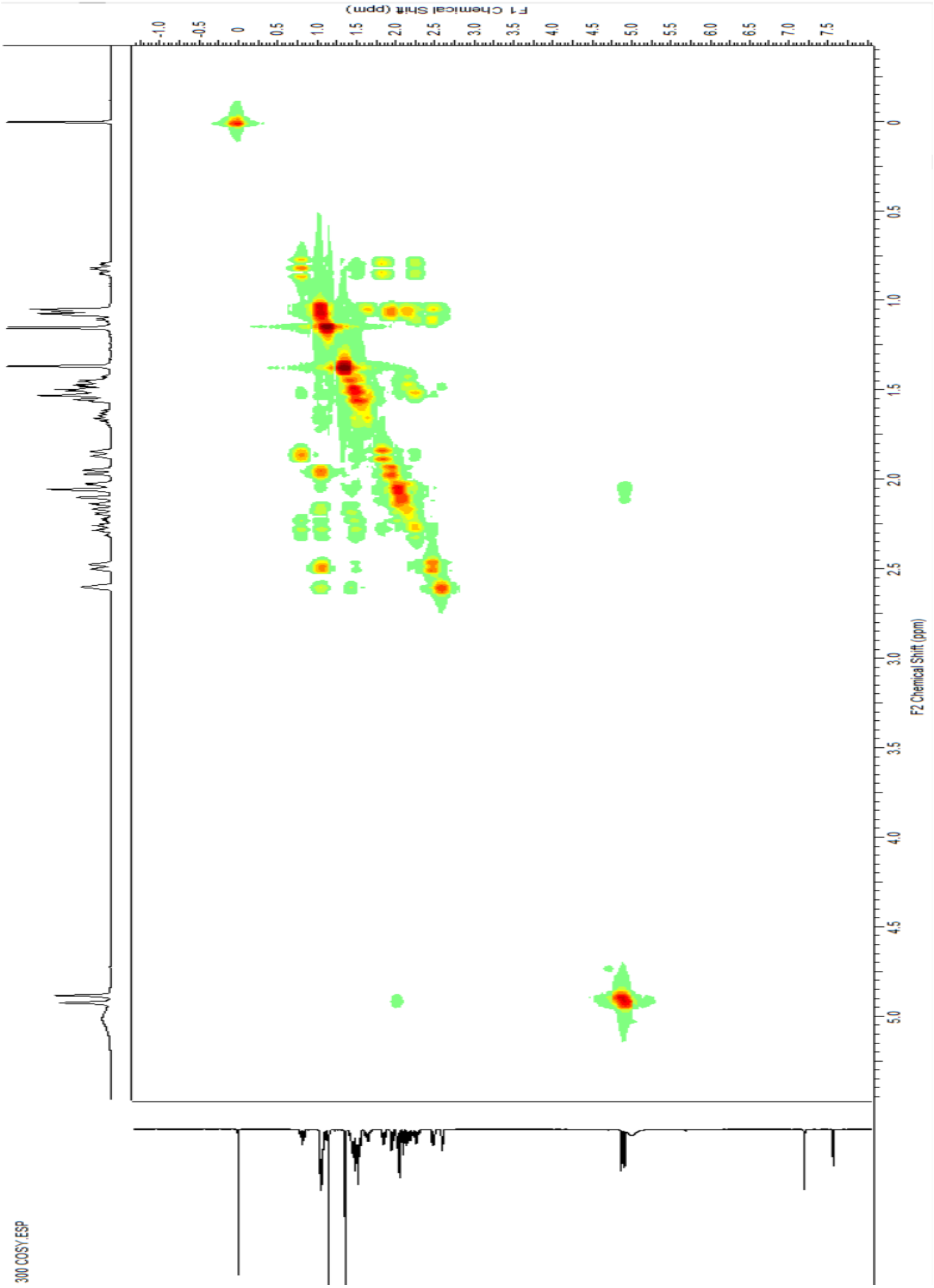

Figure S98. COSY expansion spectrum of *ent*-kaurenoic acid – C_5D_5N (500 MHz).

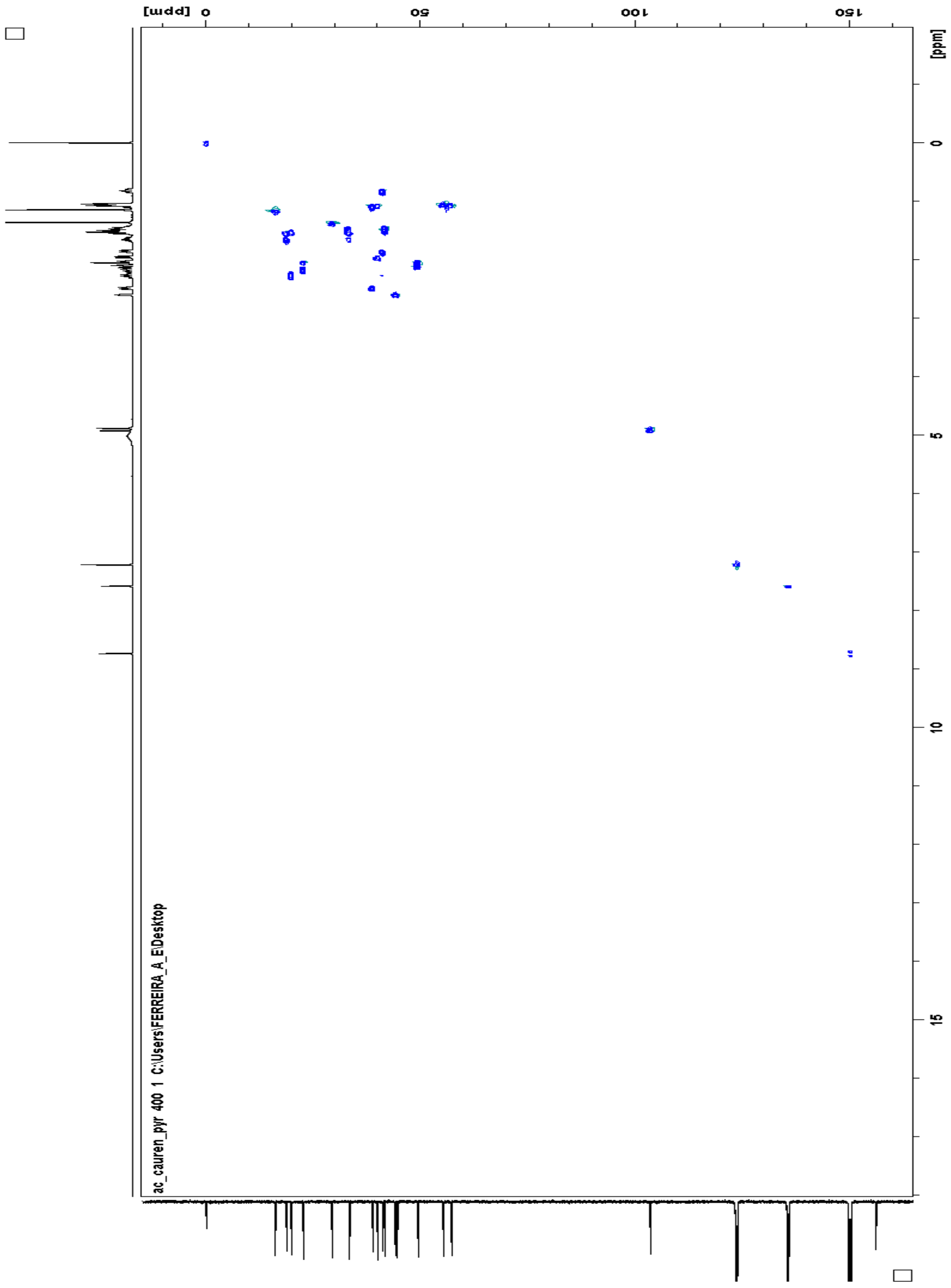

Figure S99. HSQC spectrum of *ent*-kaurenoic acid – C_5D_5N (^{13}C: 125 MHz, ^{1}H: 500 MHz).

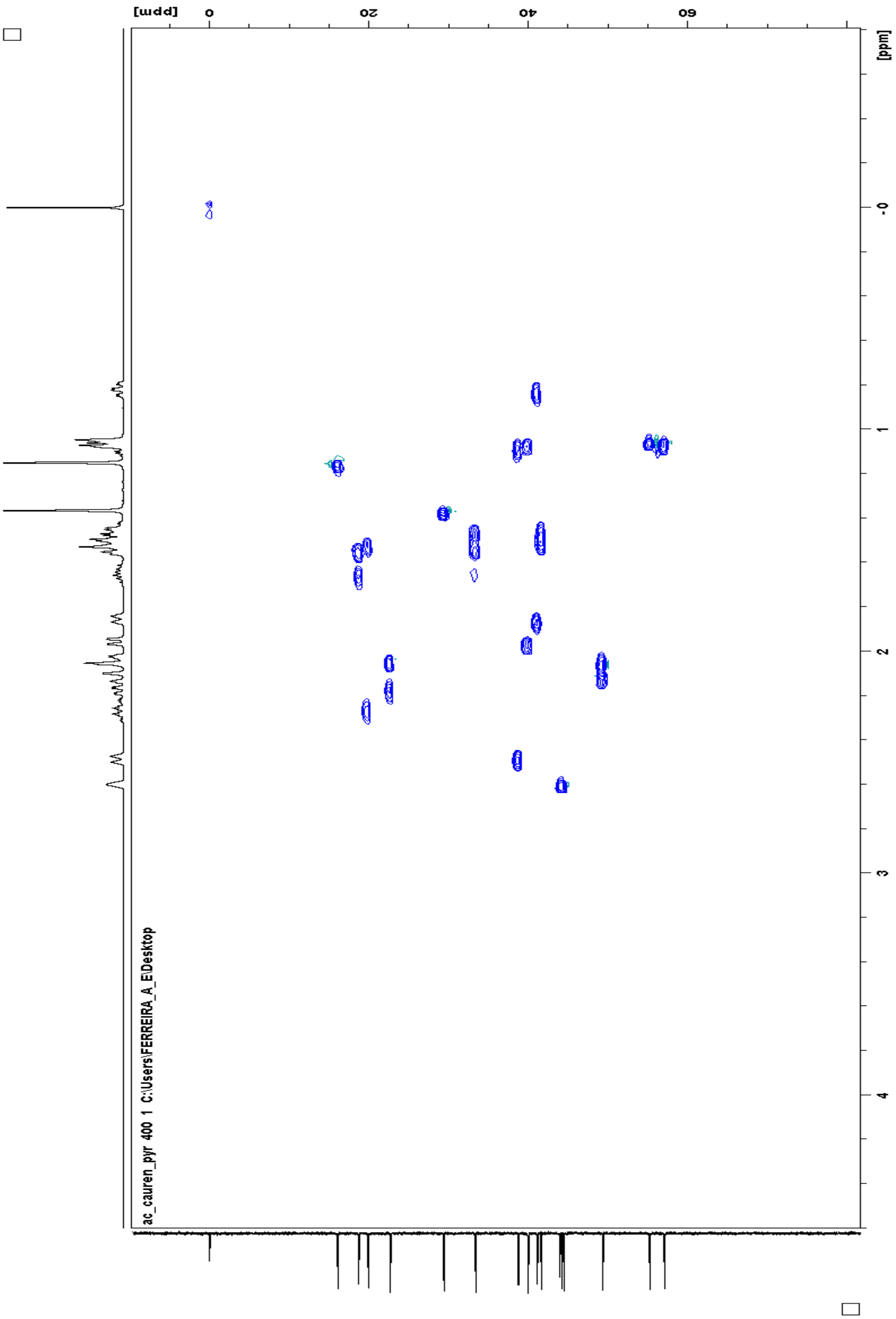

Figure S100. HSQC expansion spectrum of *ent*-kaurenoic acid – C_5D_5N (^{13}C: 125 MHz, ^{1}H: 500 MHz).

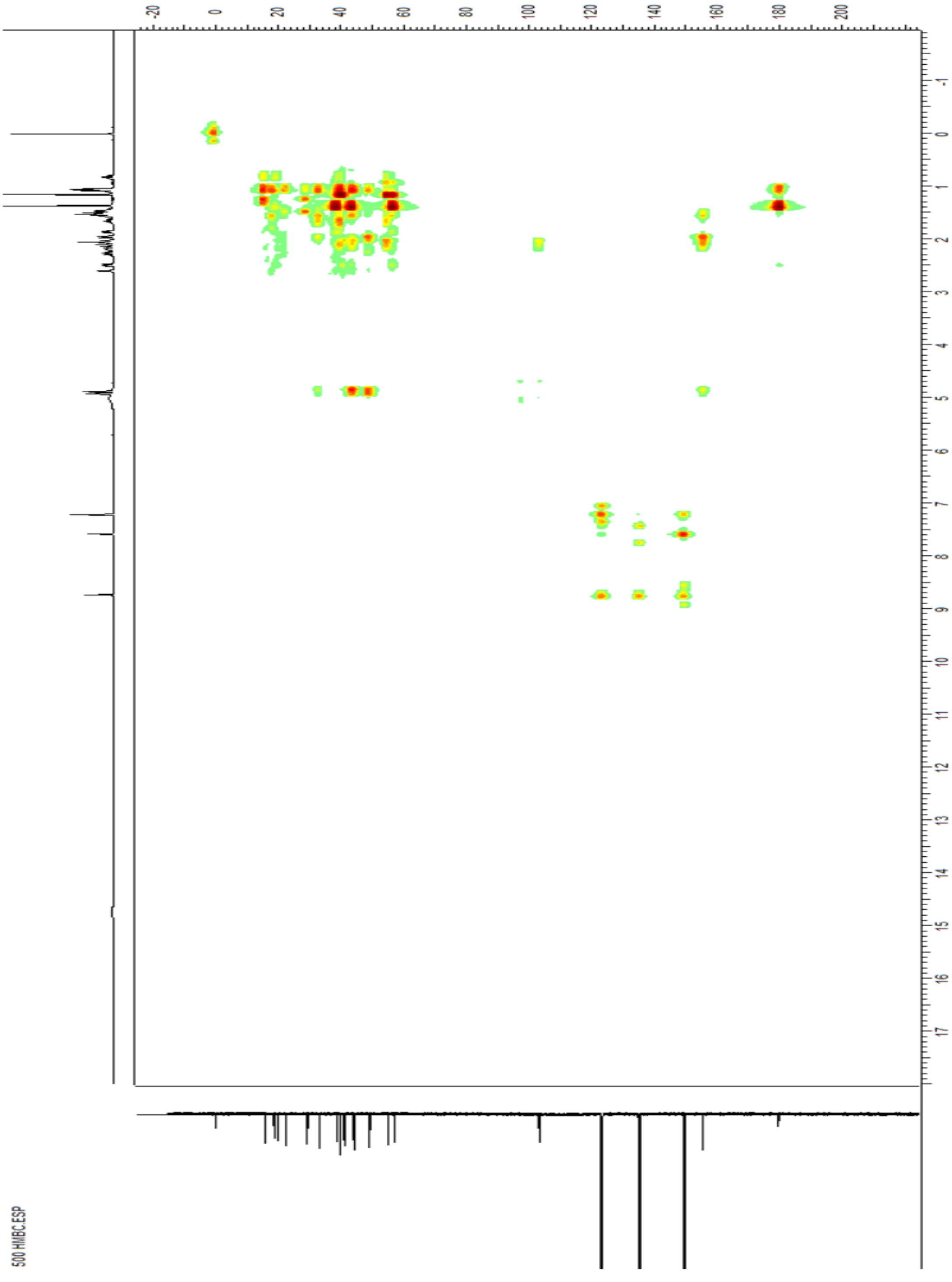

Figure S101. HMBC spectrum of *ent*-kaurenoic acid – C_5D_5N (^{13}C: 125 MHz, ^{1}H: 500 MHz).

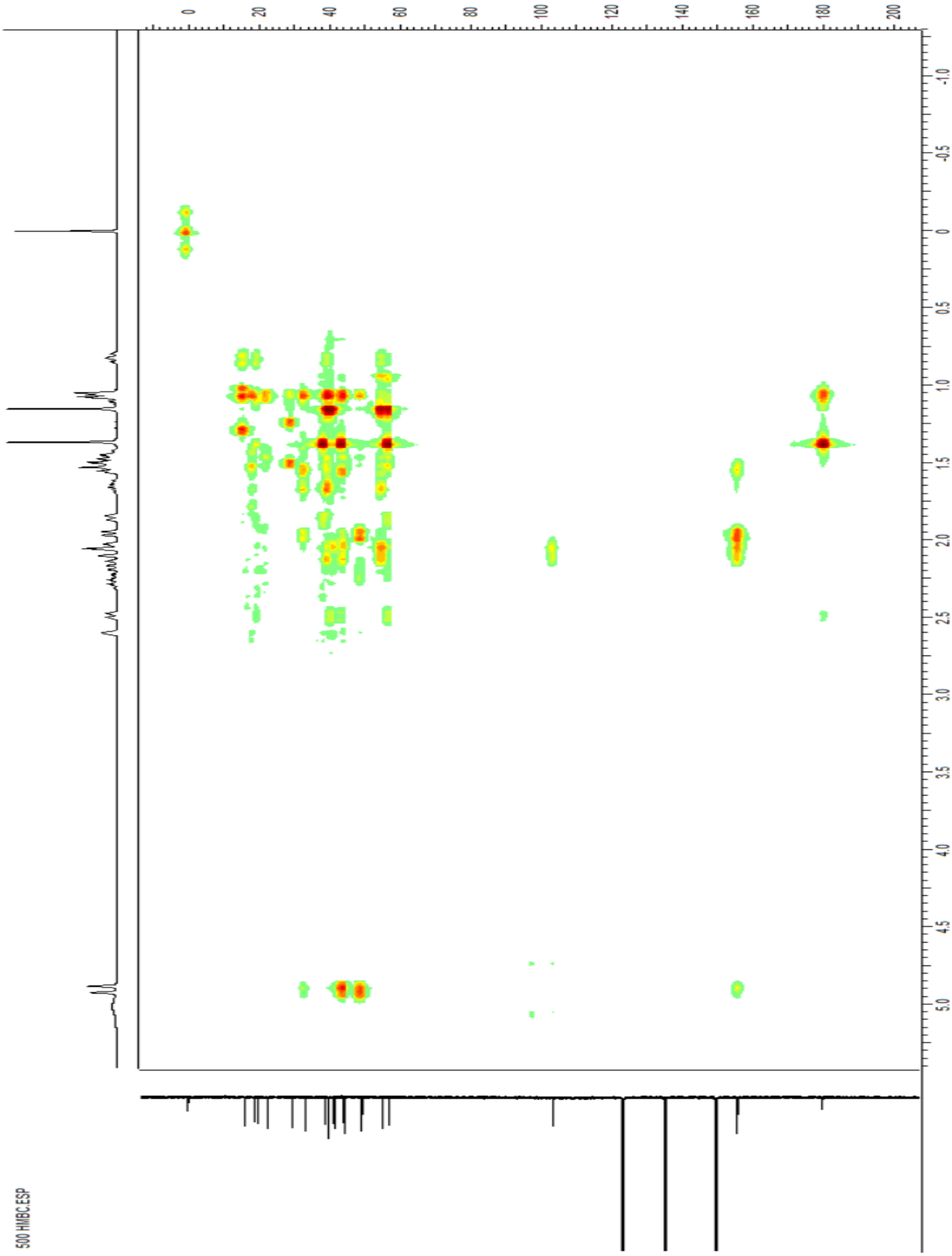

Figure S102. HMBC expansion spectrum of *ent*-kaurenoic acid – C_5D_5N (^{13}C: 125 MHz, ^1H: 500 MHz).

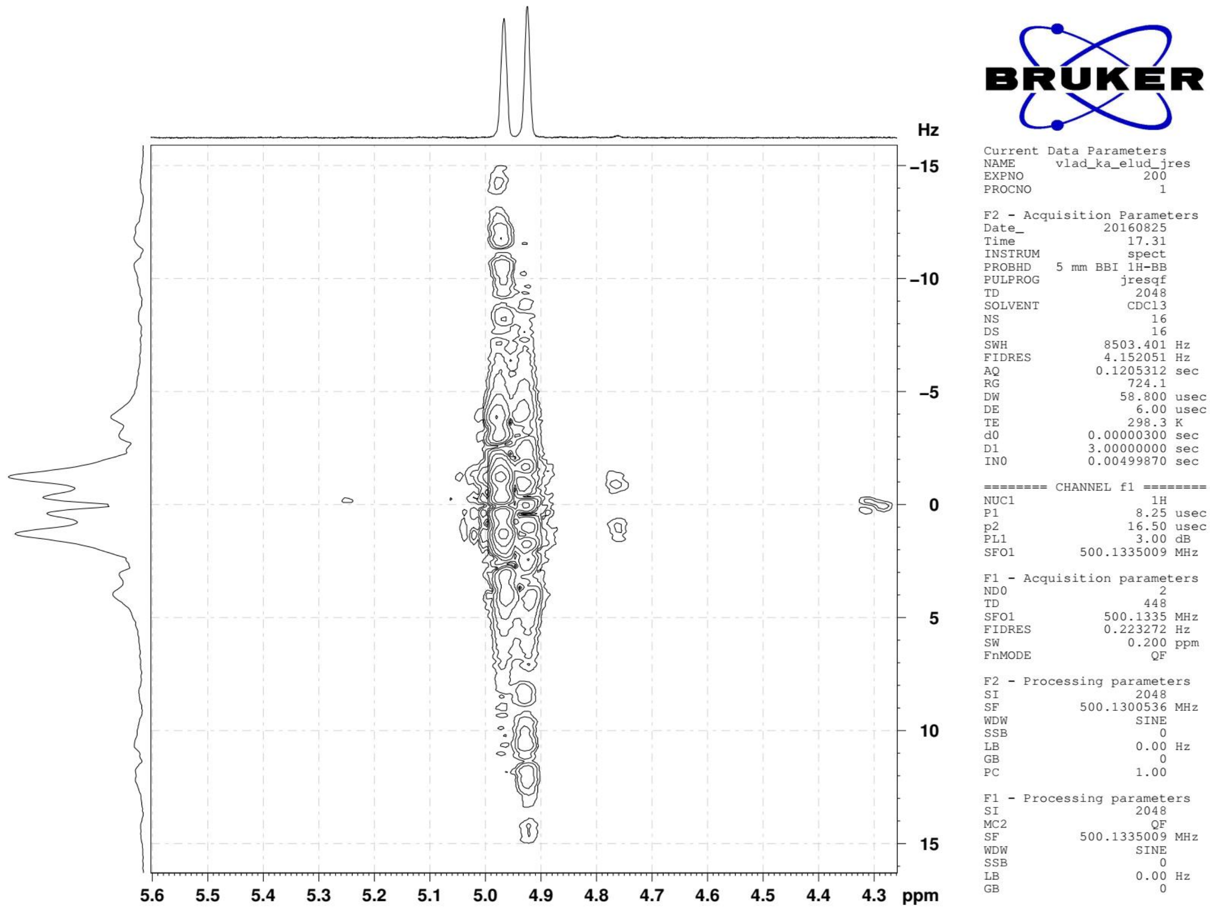

Figure S103. *J-resolved* spectrum for *ent*-kaurenoic acid - Expansion 1.

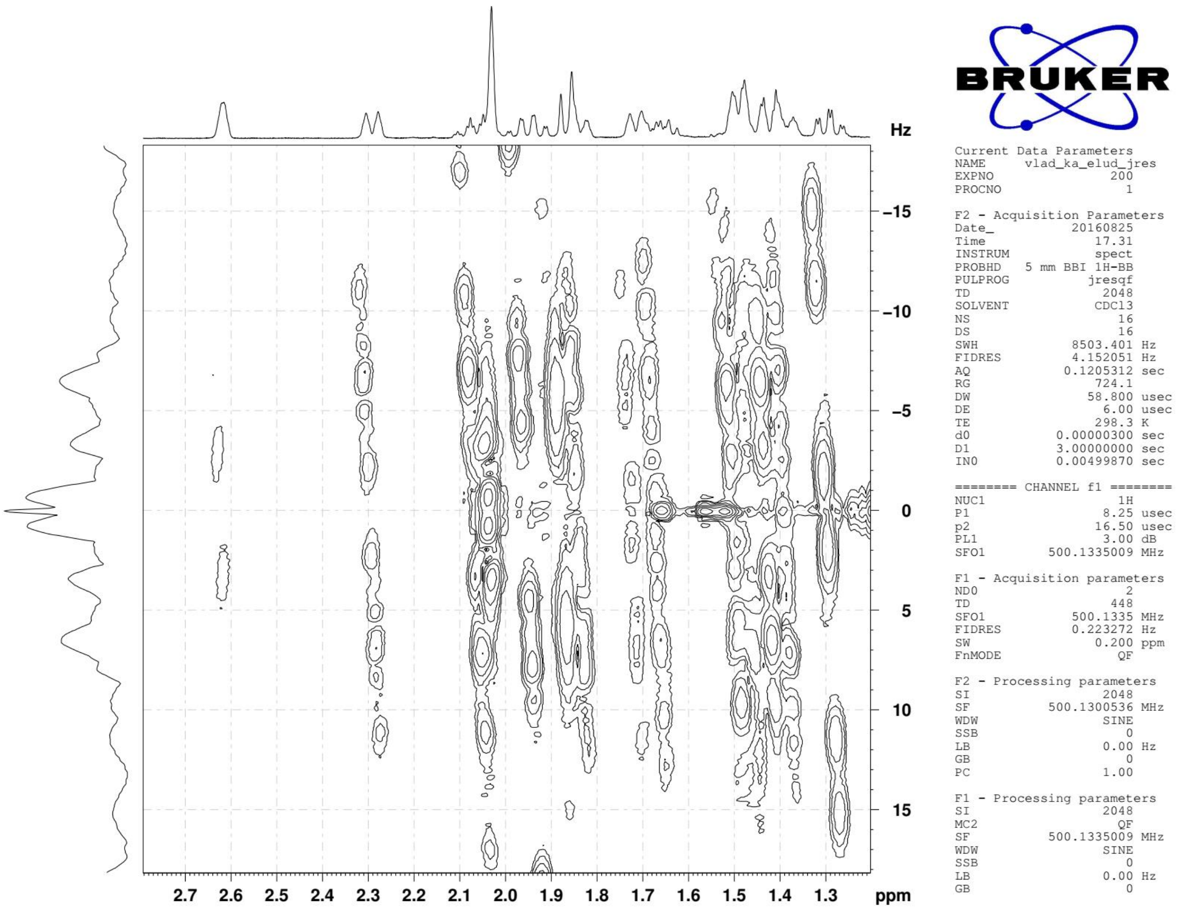

Figure S104. *J-resolved* spectrum for *ent*-kaurenoic acid - Expansion 2.

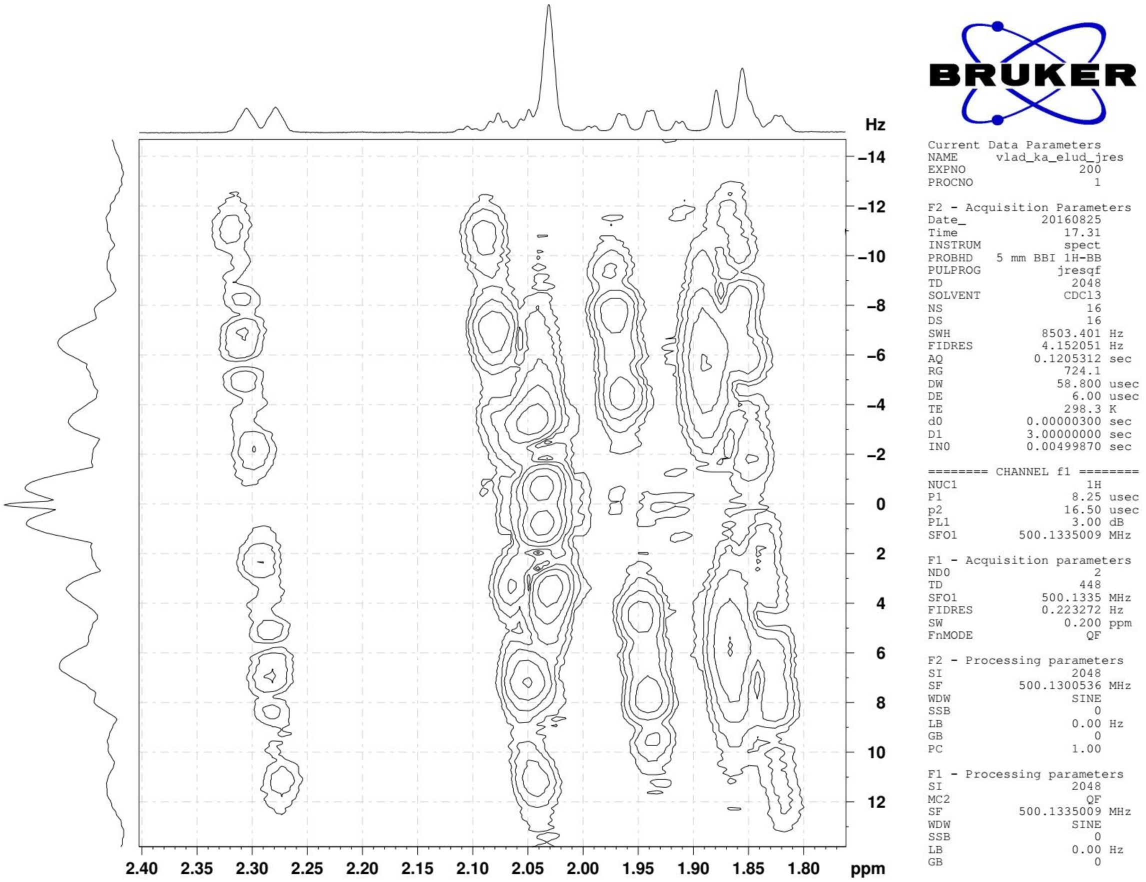

Figure S105. *J-resolved* spectrum for *ent*-kaurenoic acid - Expansion 3.

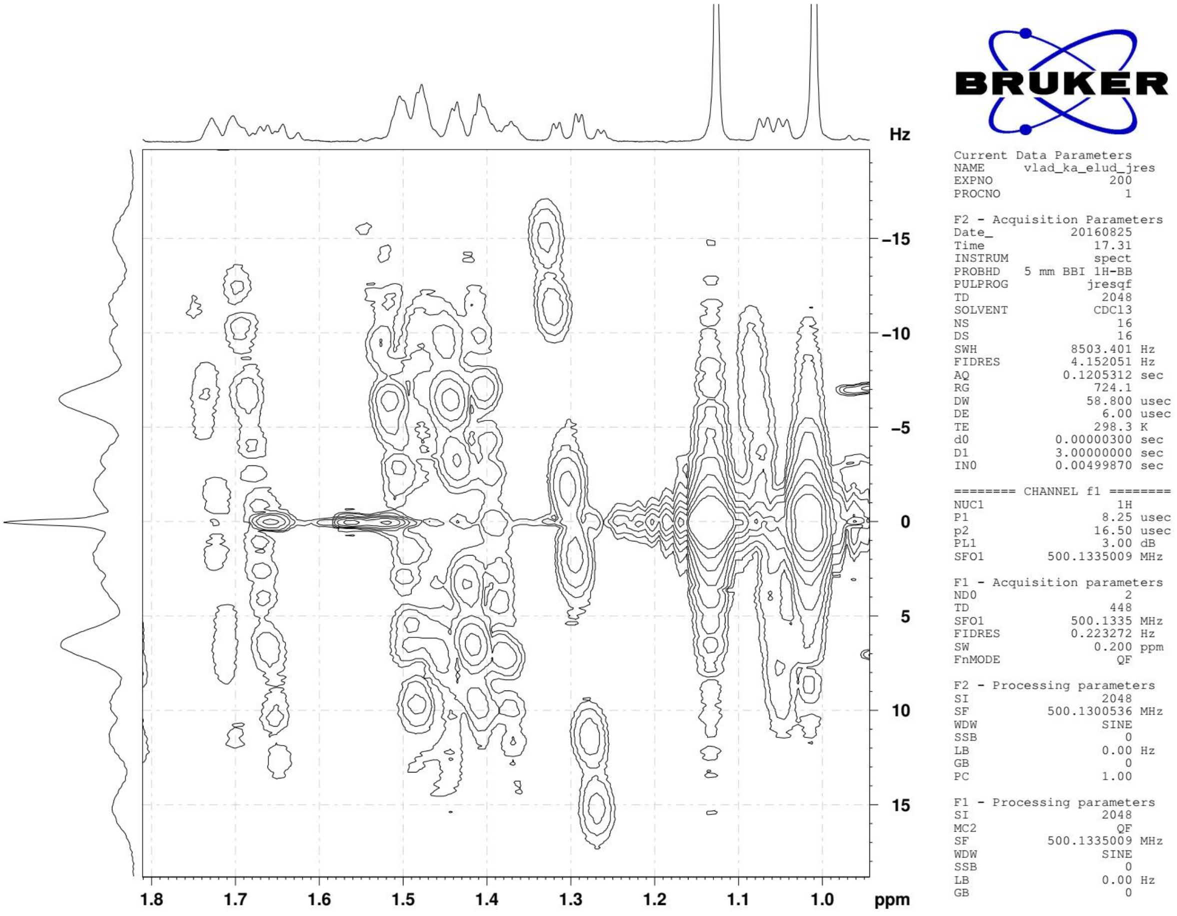

Figure S106. *J-resolved* spectrum for *ent*-kaurenoic acid - Expansion 4.

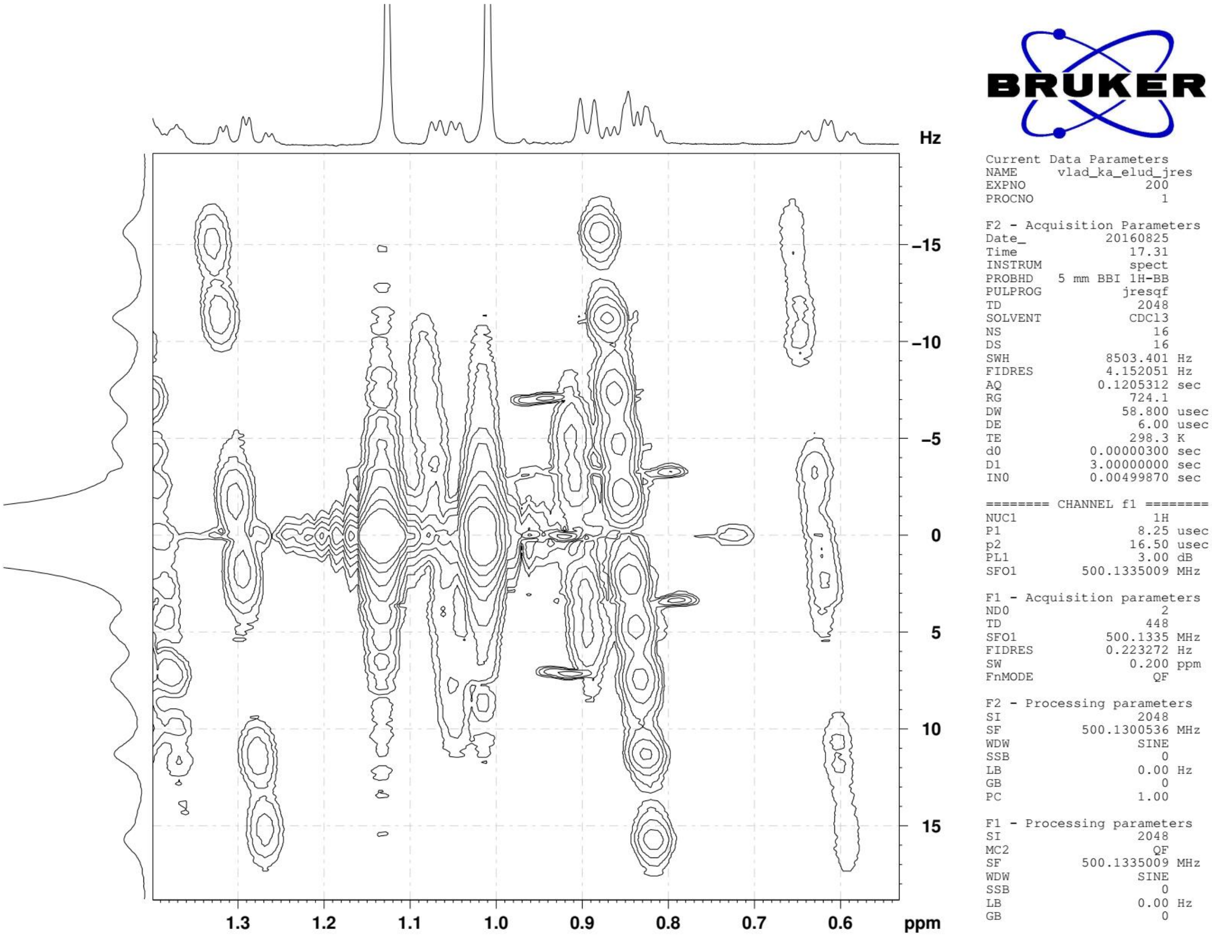

Figure S107. *J-resolved* spectrum for *ent*-kaurenoic acid - Expansion 5.

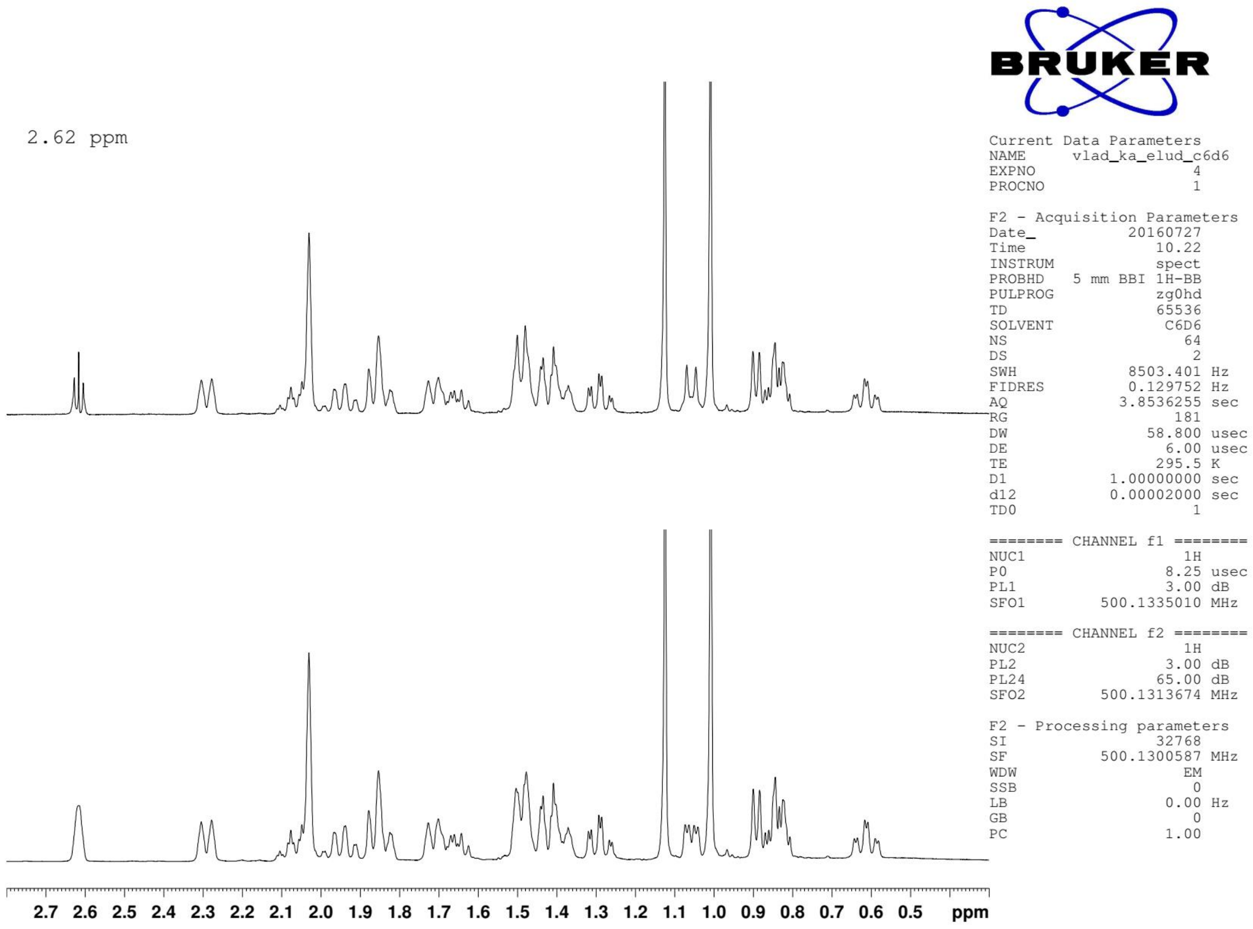

Figure S108. Double-Irradiation Methods in 2.62 ppm for *ent*-kaurenoic acid (C_6D_6 / ^{1}H: 500 MHz).

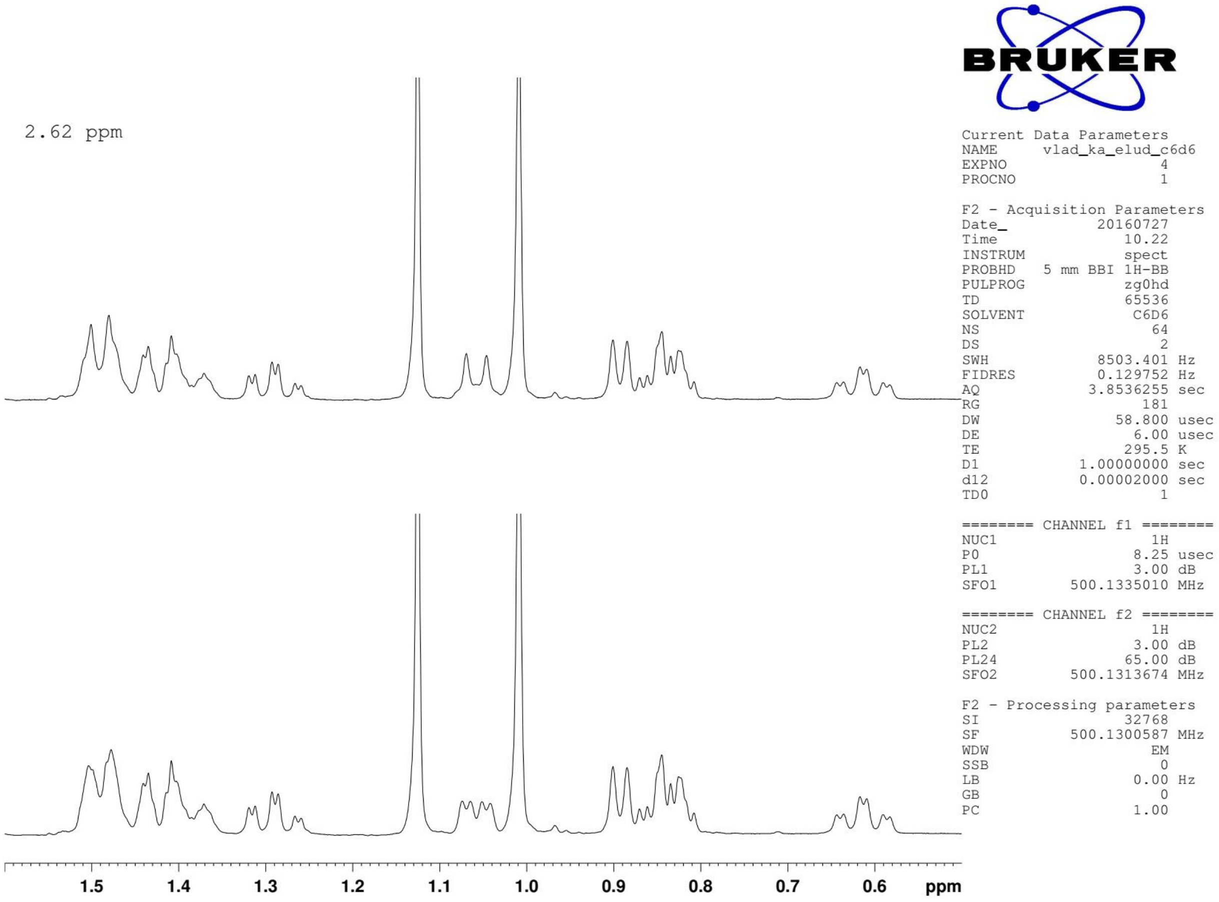

Figure S109. Double-Irradiation Methods in 2.62 ppm for *ent*-kaurenoic acid (C_6D_6 / ^1H: 500 MHz) - Expansion 1.

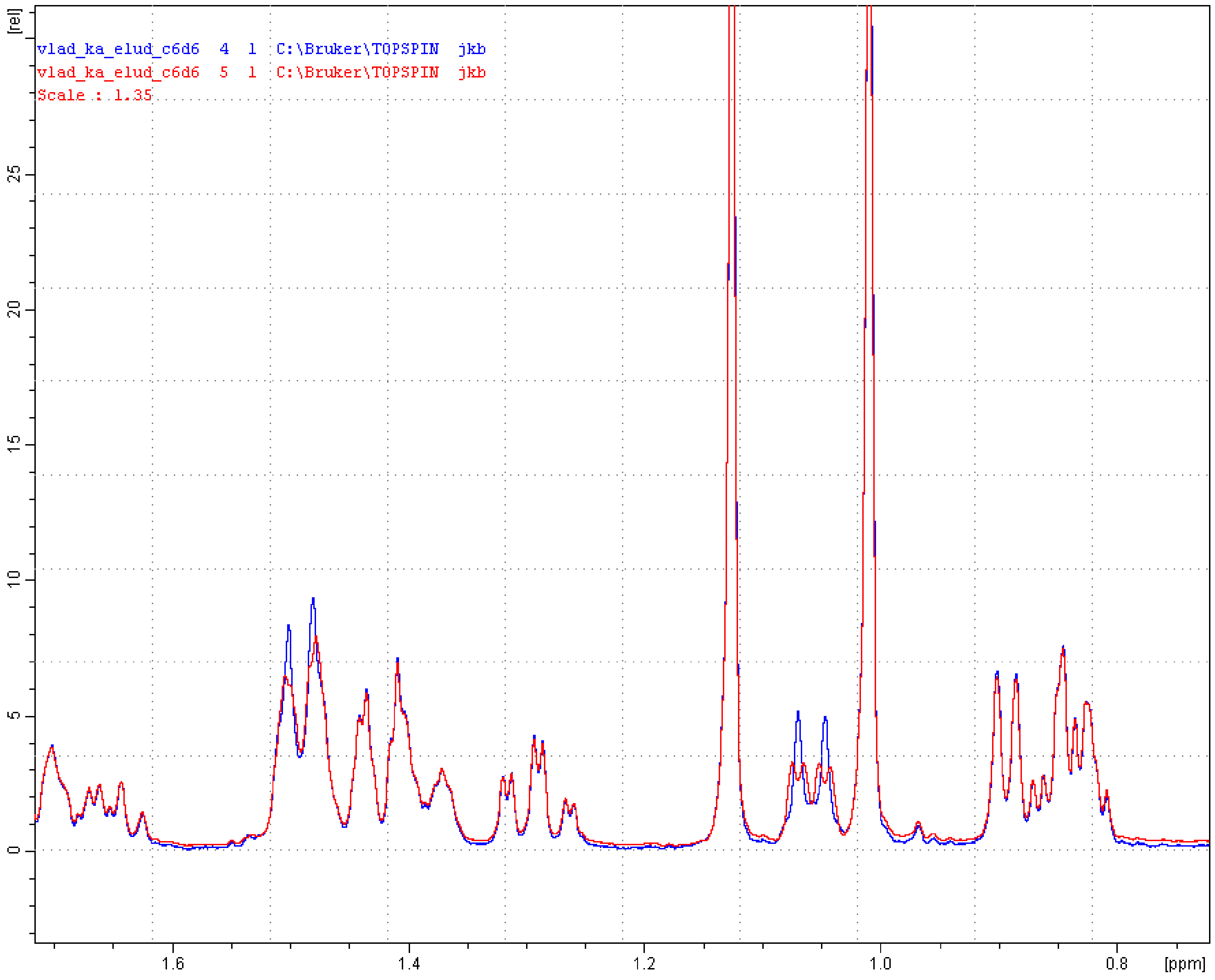

Figure S110. Double-Irradiation Methods in 2.62 ppm for *ent*-kaurenoic acid (C_6D_6 / ^1H: 500 MHz) - ***Comparative analysis***.

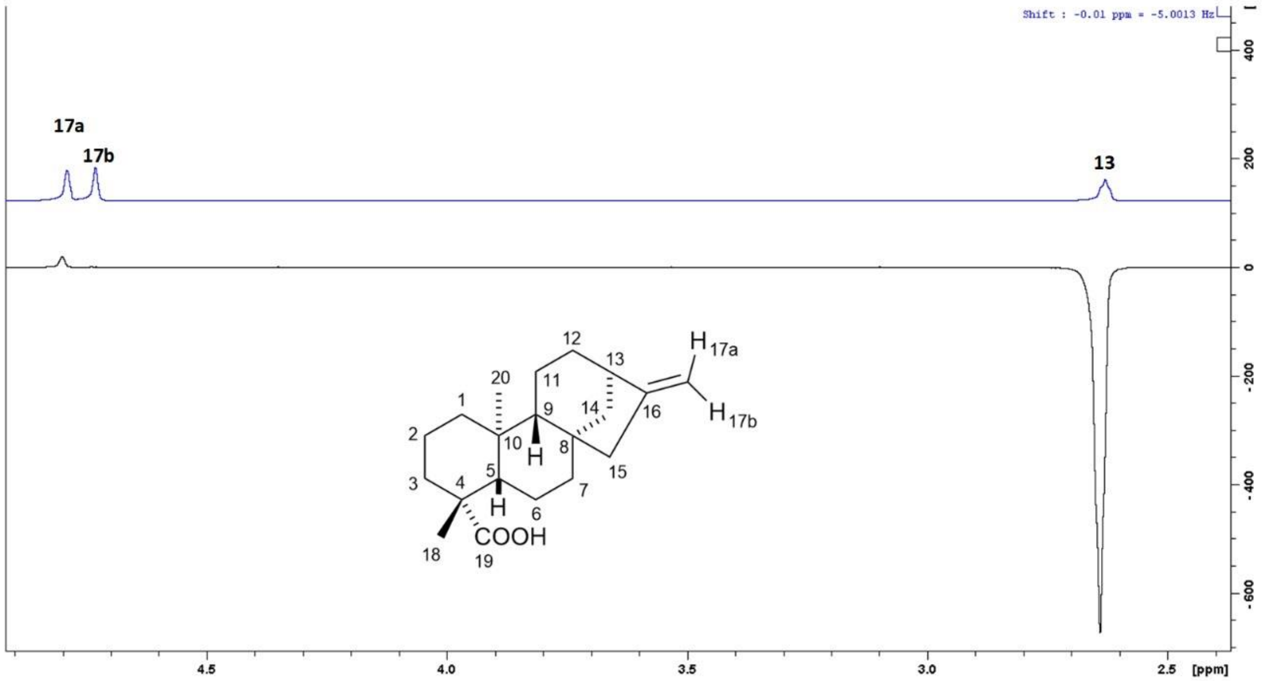

Figure S111: Results from NOESY spectra of *ent*-kaurenoic acid (KA) – $CDCl_3$, 500 MHz. Above, in blue, the original ^{1}H-NMR spectrum and below, in black, the NOE spectrum with irradiation in H13.

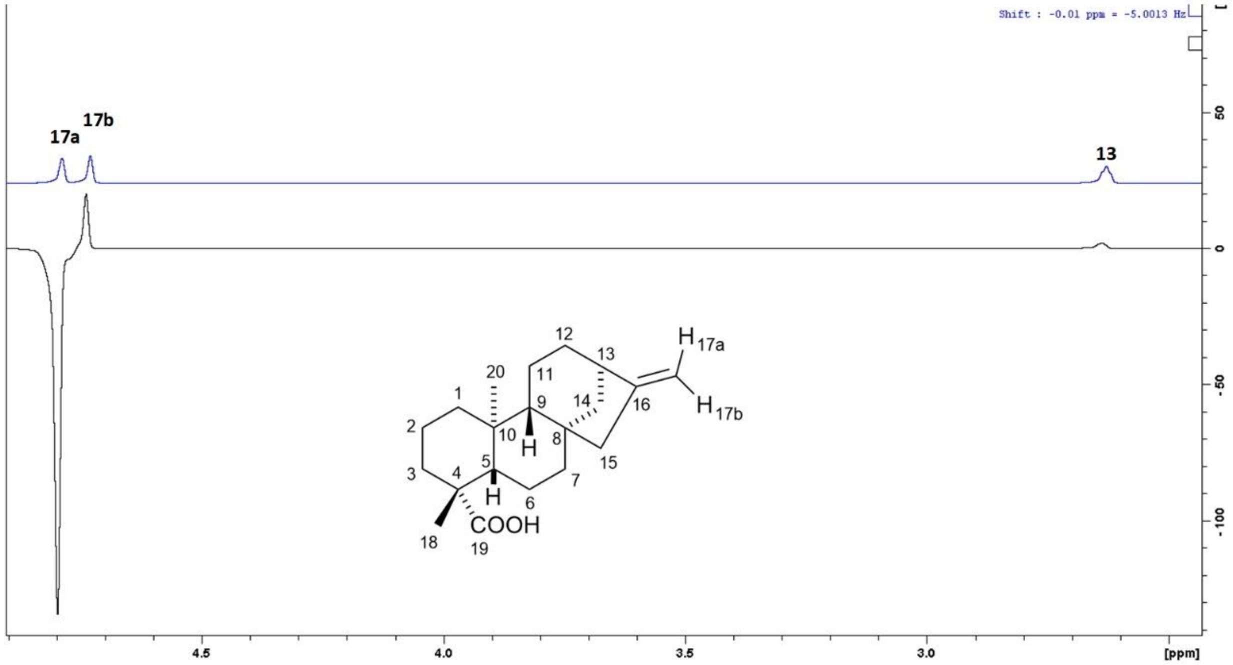

Figure S112: Results from NOESY spectra of *ent*-kaurenoic acid (KA) – $CDCl_3$, 500 MHz. Above, in blue, the original ^{1}H-NMR spectrum and below, in black, the NOE spectrum with irradiation in H17a.

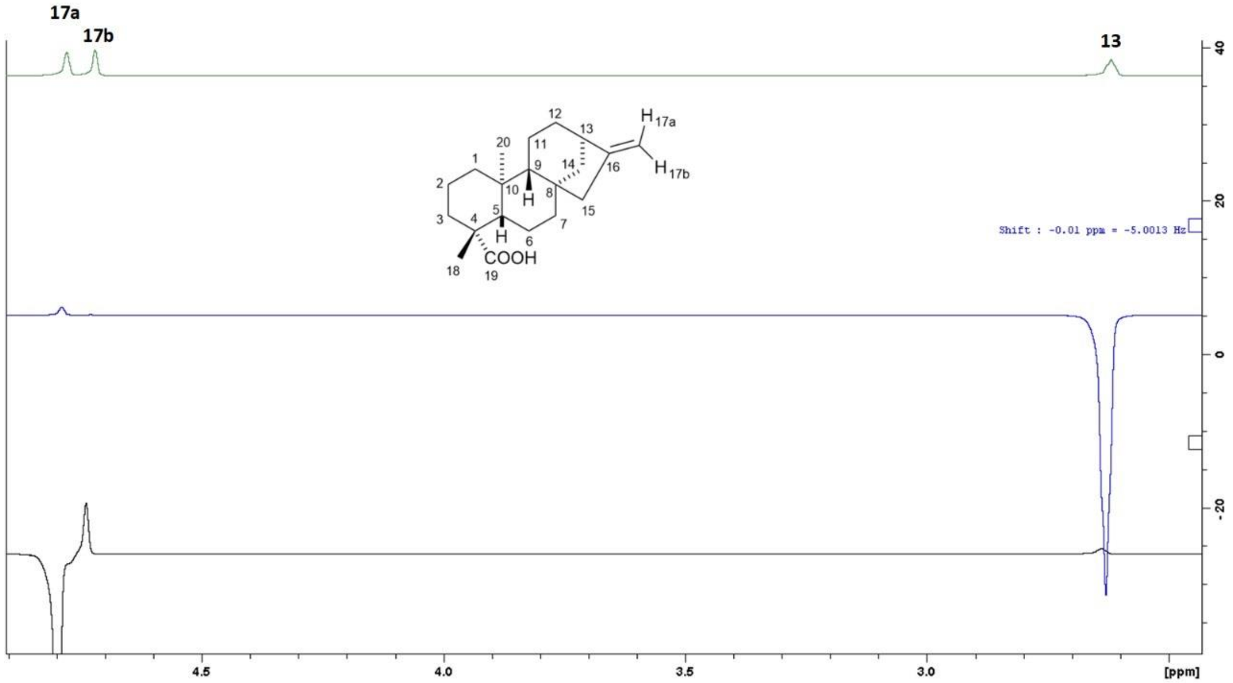

Figure S113: Results from NOESY spectra of *ent*-kaurenoic acid (KA) – $CDCl_3$, 500 MHz. Above, in green, the original ^{1}H-NMR spectrum, in the middle, in blue, NOE spectrum with irradiation at H13 and below, in black, the NOE spectrum with irradiation in H17a – together for comparison.

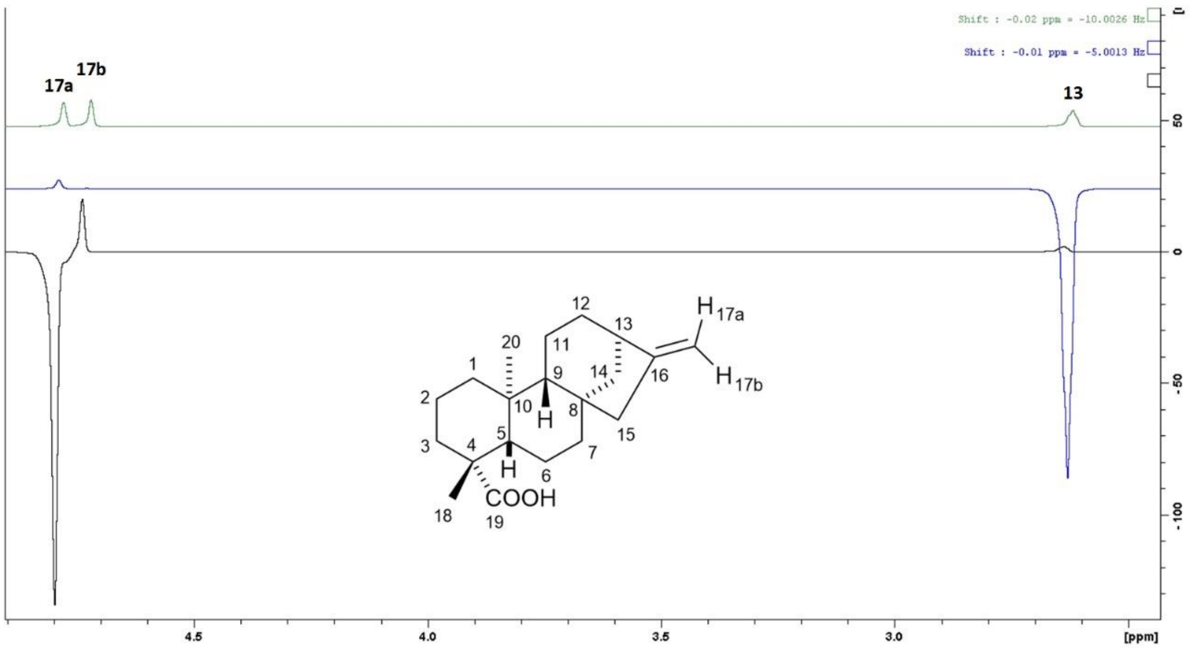

Figure S114: Results from NOESY spectra of *ent*-kaurenoic acid (KA) – $CDCl_3$, 500 MHz. Above, in green, the original ^{1}H-NMR spectrum, in the middle, in blue, NOE spectrum with irradiation at H13 and below, in black, the NOE spectrum with irradiation in H17a – together for comparison.

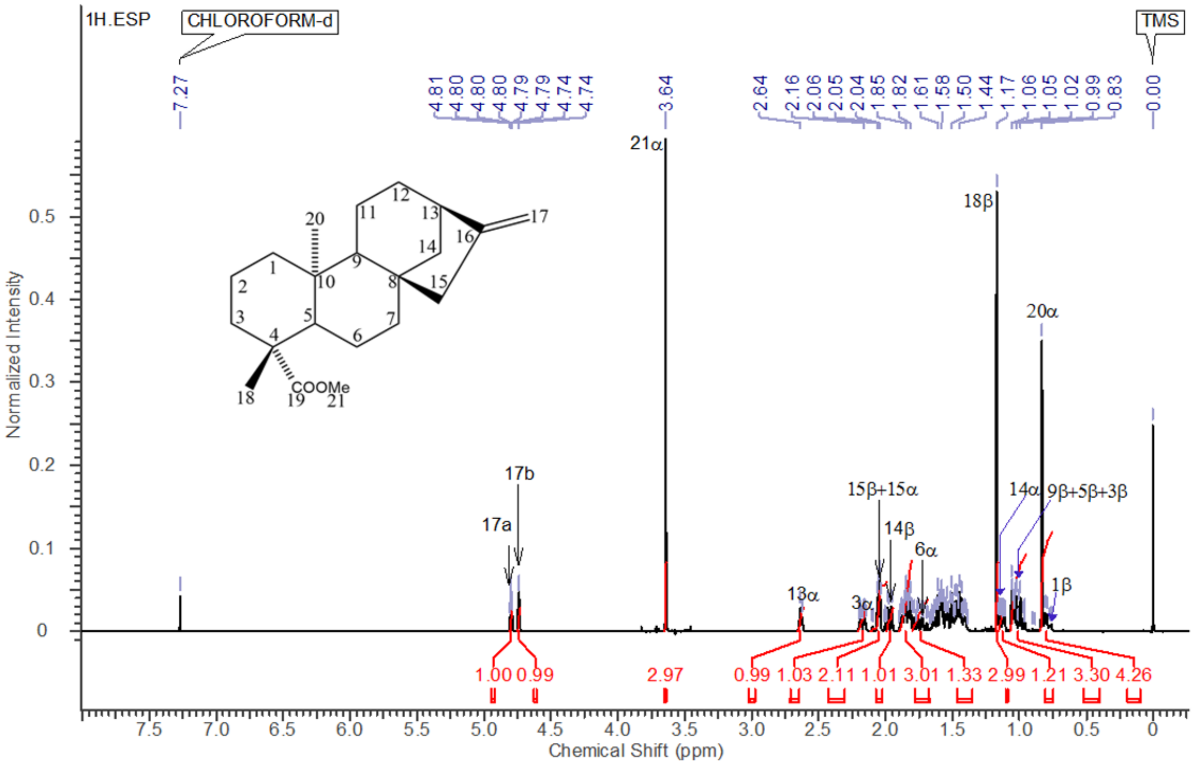

Figures S115. ^{1}H NMR spectrum of methyl ent-kaur-16-en-19-oate – $CDCl_3$, 400 MHz.

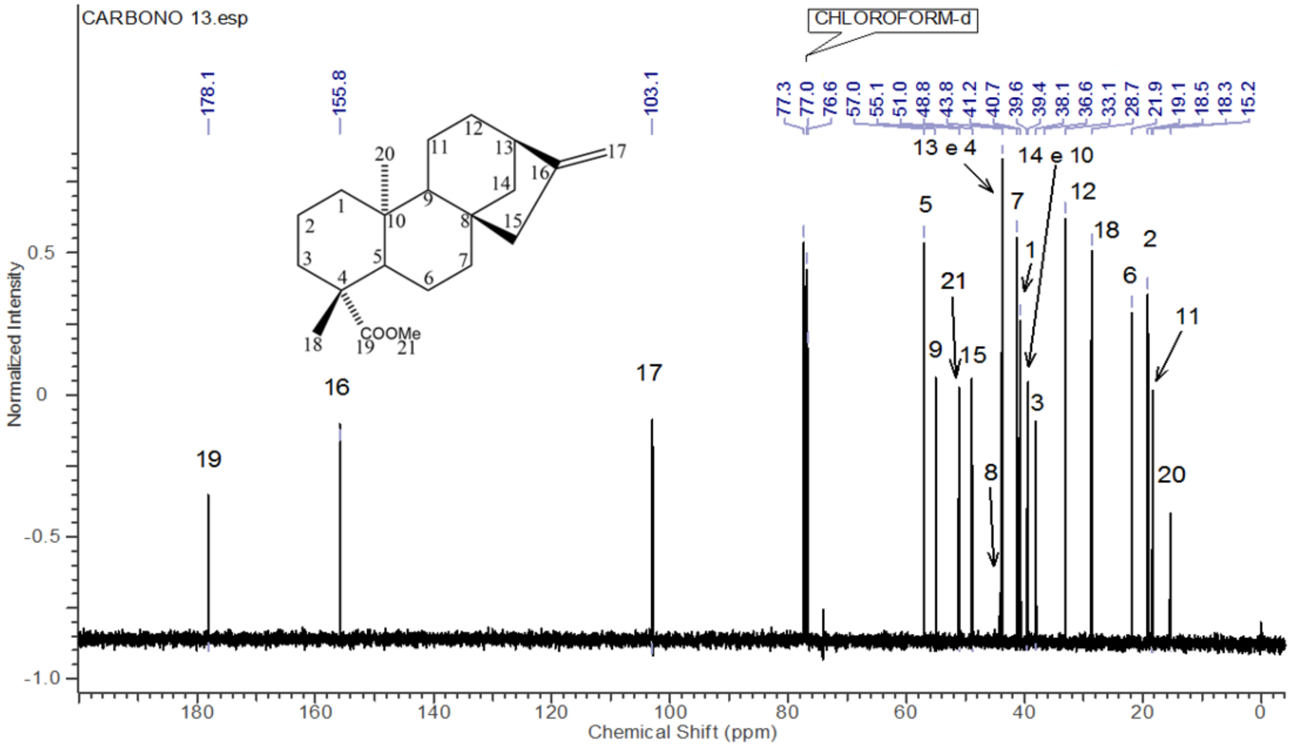

Figure S116. ^{13}C {^{1}H } NMR spectrum of methyl ent-kaur-16-en-19-oate – $CDCl_3$, 100 MHz.

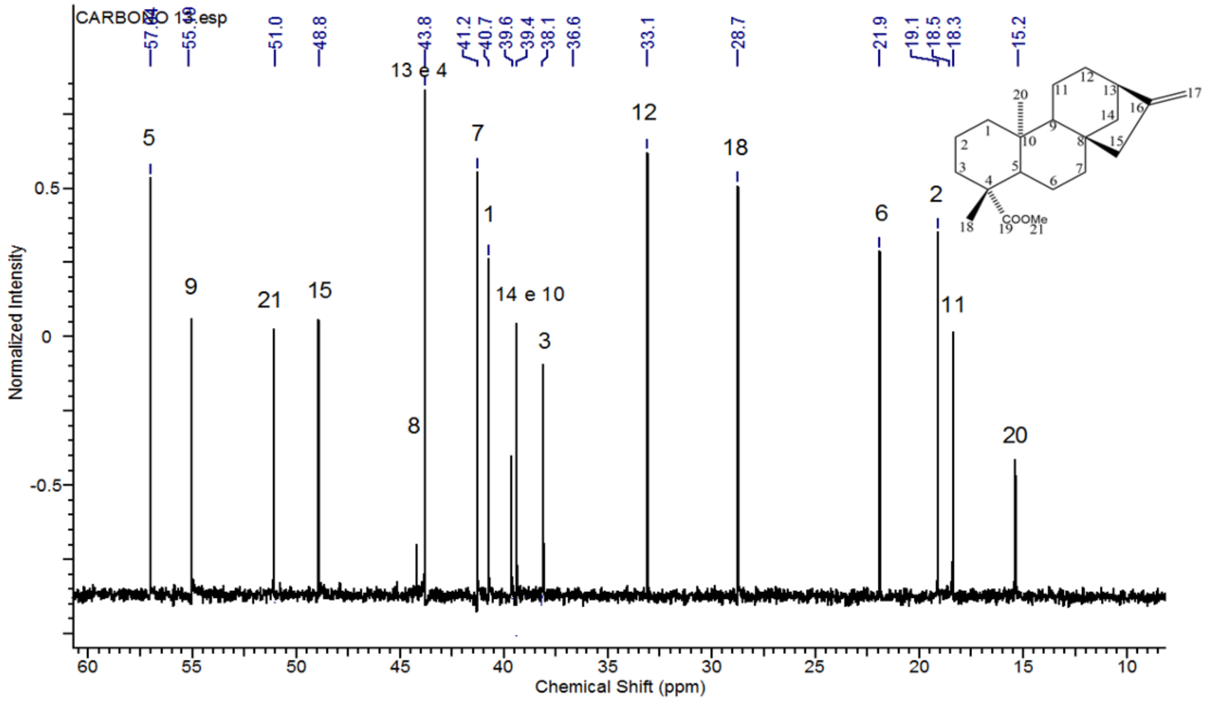

Figure S117. ^{13}C {^{1}H } NMR spectrum of methyl ent-kaur-16-en-19-oate – $CDCl_3$, 100 MHz – Expansion 1.

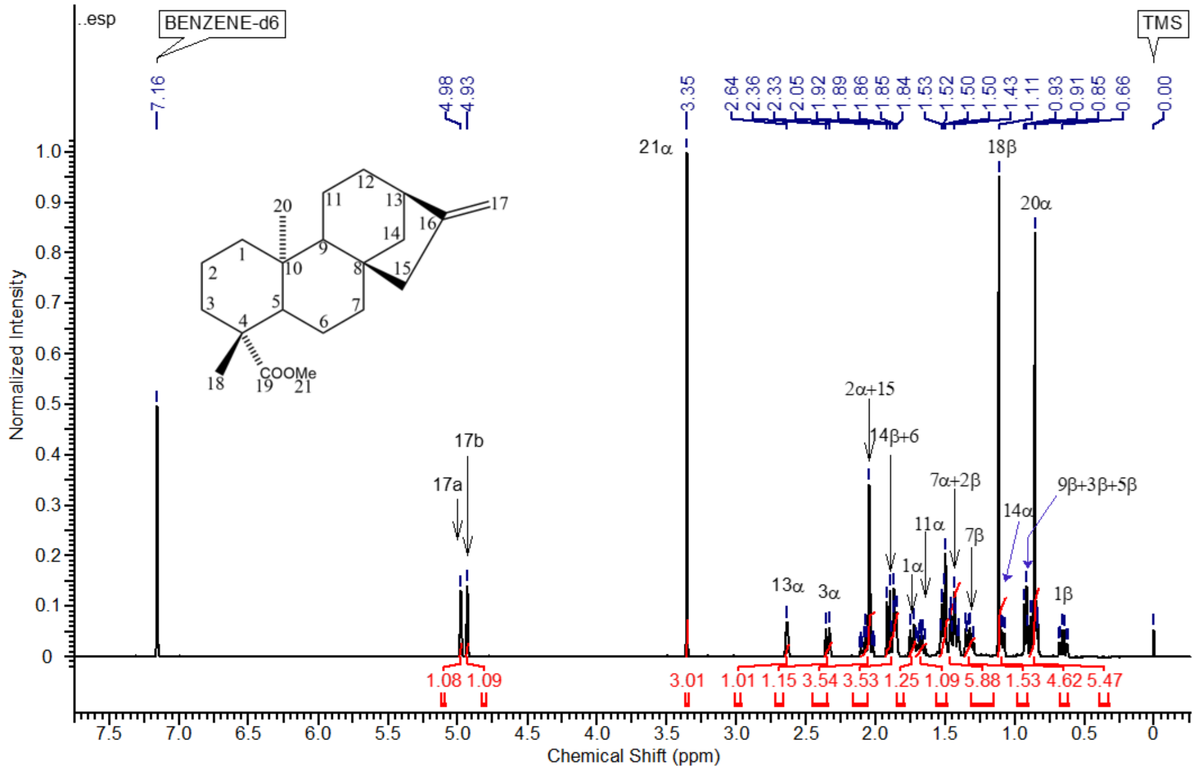

Figure S118. ^{1}H NMR spectrum of methyl ent-kaur-16-en-19-oate – C_6D_6, 500 MHz.

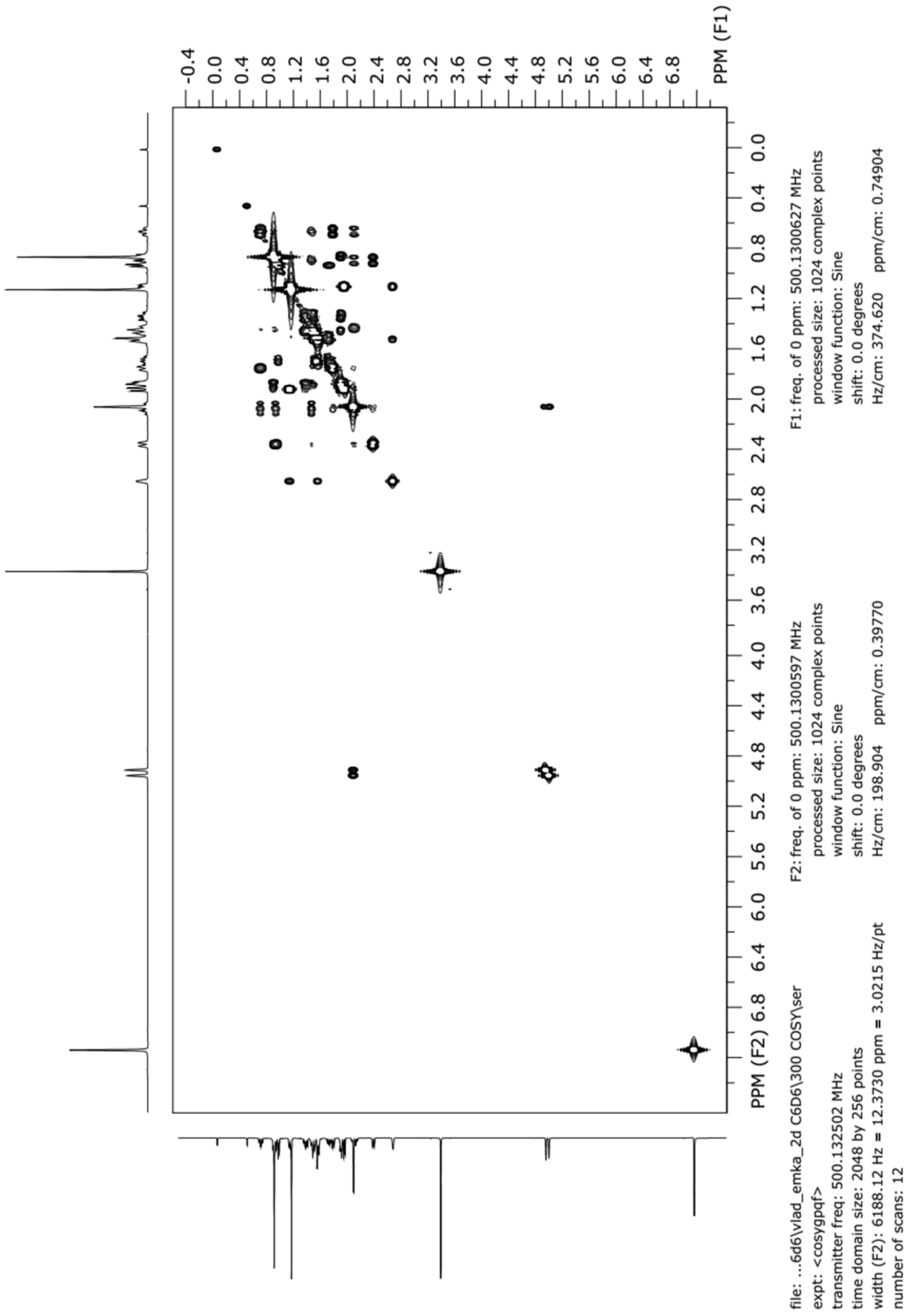

Figure S119. COSY spectrum of methyl ent-kaur-16-en-19-oate – C_6D_6 (^{1}H: 500 MHz).

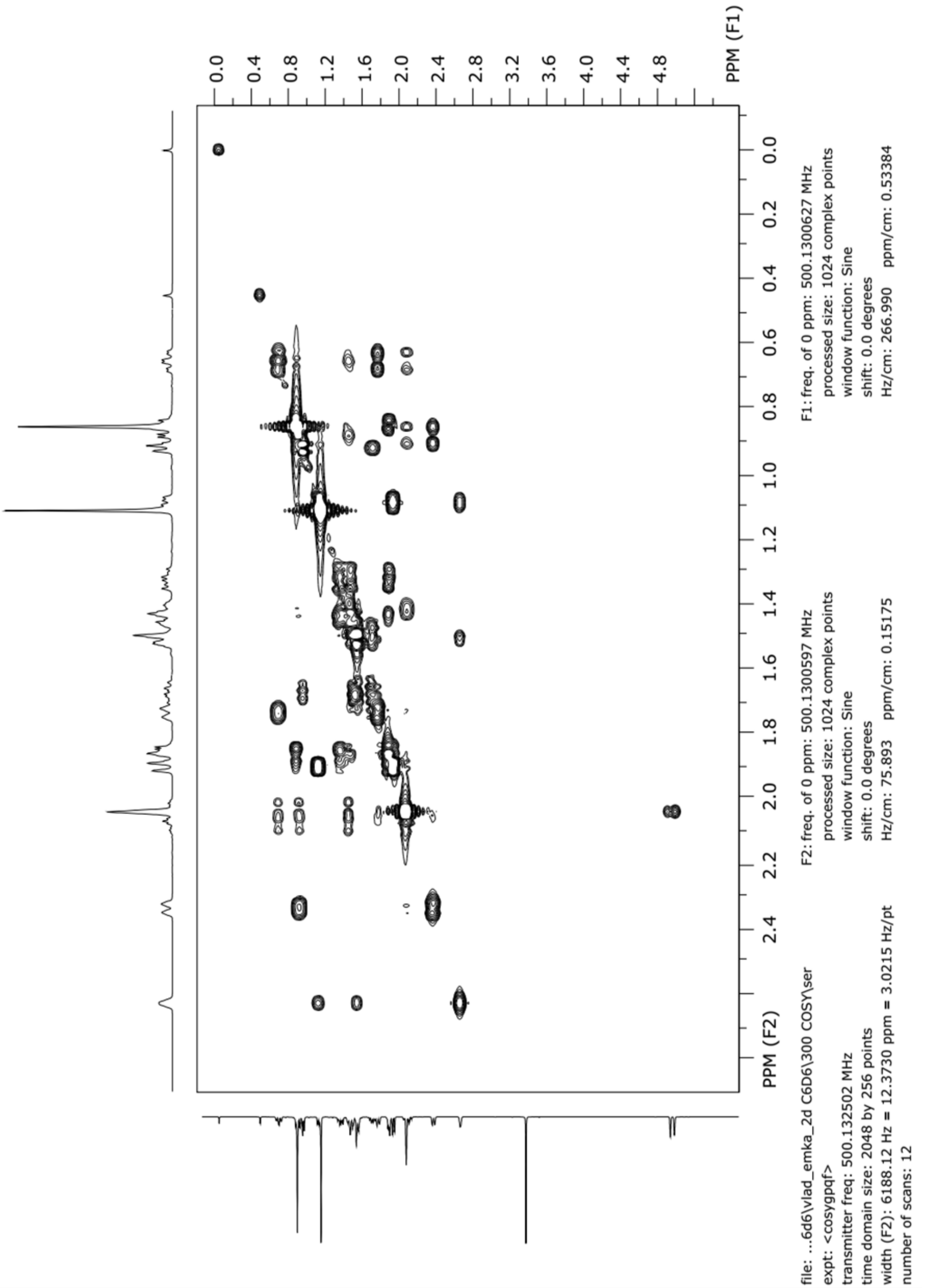

Figure S120. COSY expansion spectrum of methyl ent-kaur-16-en-19-oate – C_6D_6 (^1H: 500 MHz)

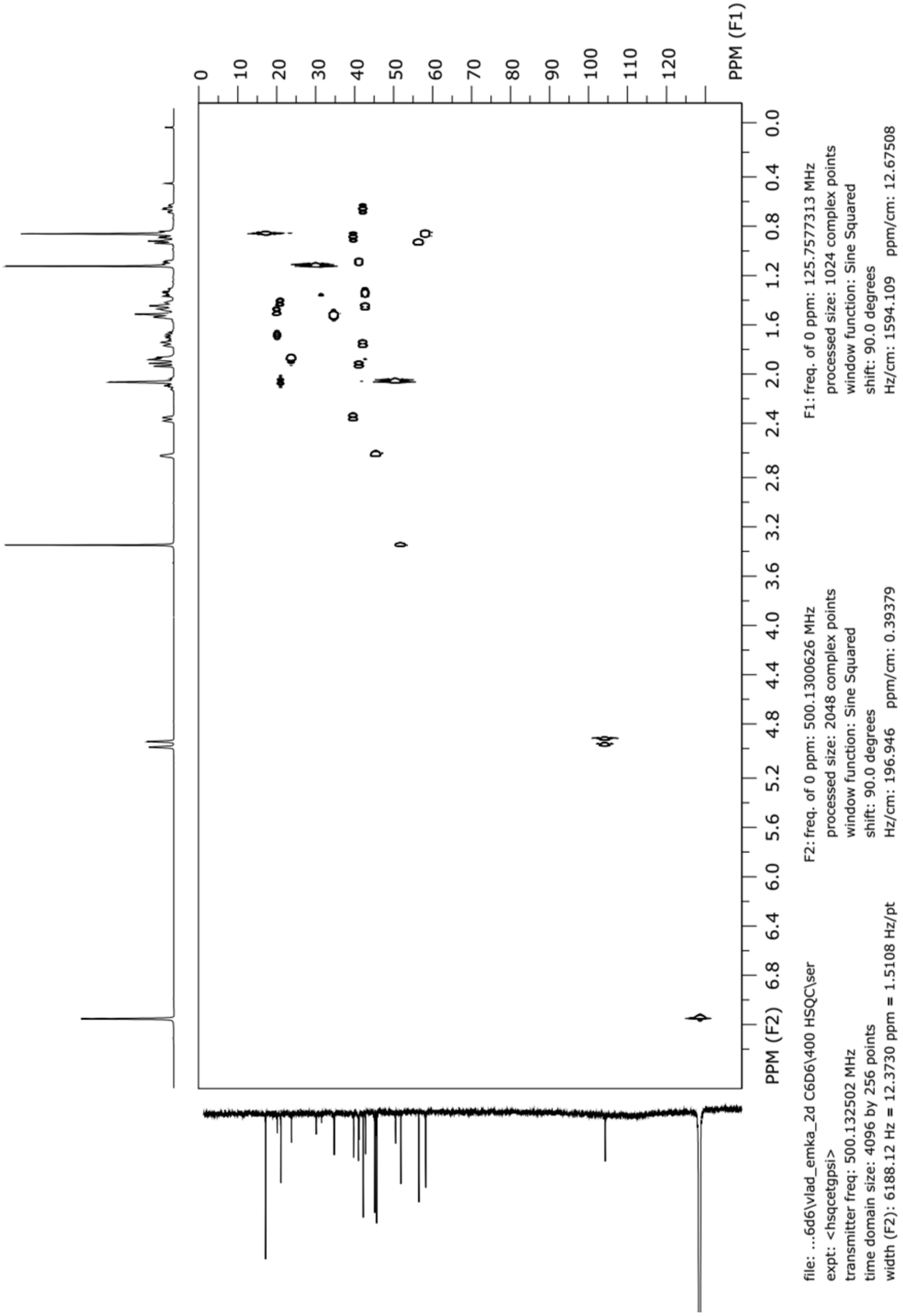

Figure S121. HSQC spectrum of methyl ent-kaur-16-en-19-oate – C_6D_6 (^{13}C: 125 MHz, ^{1}H: 500 MHz).

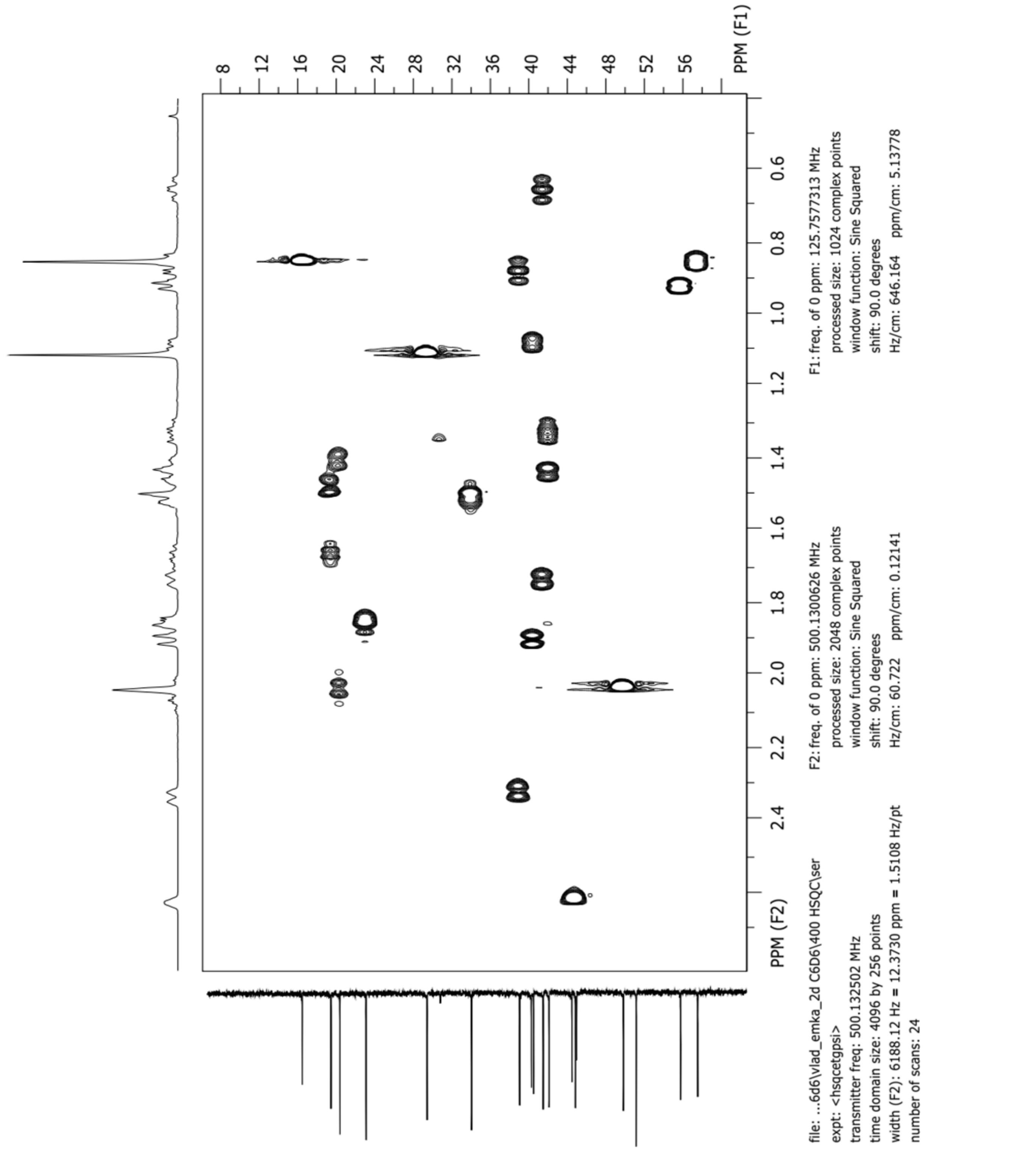

Figure S122. HSQC expansion spectrum of methyl ent-kaur-16-en-19-oate – C_6D_6 (^{13}C: 125 MHz, ^{1}H: 500 MHz).

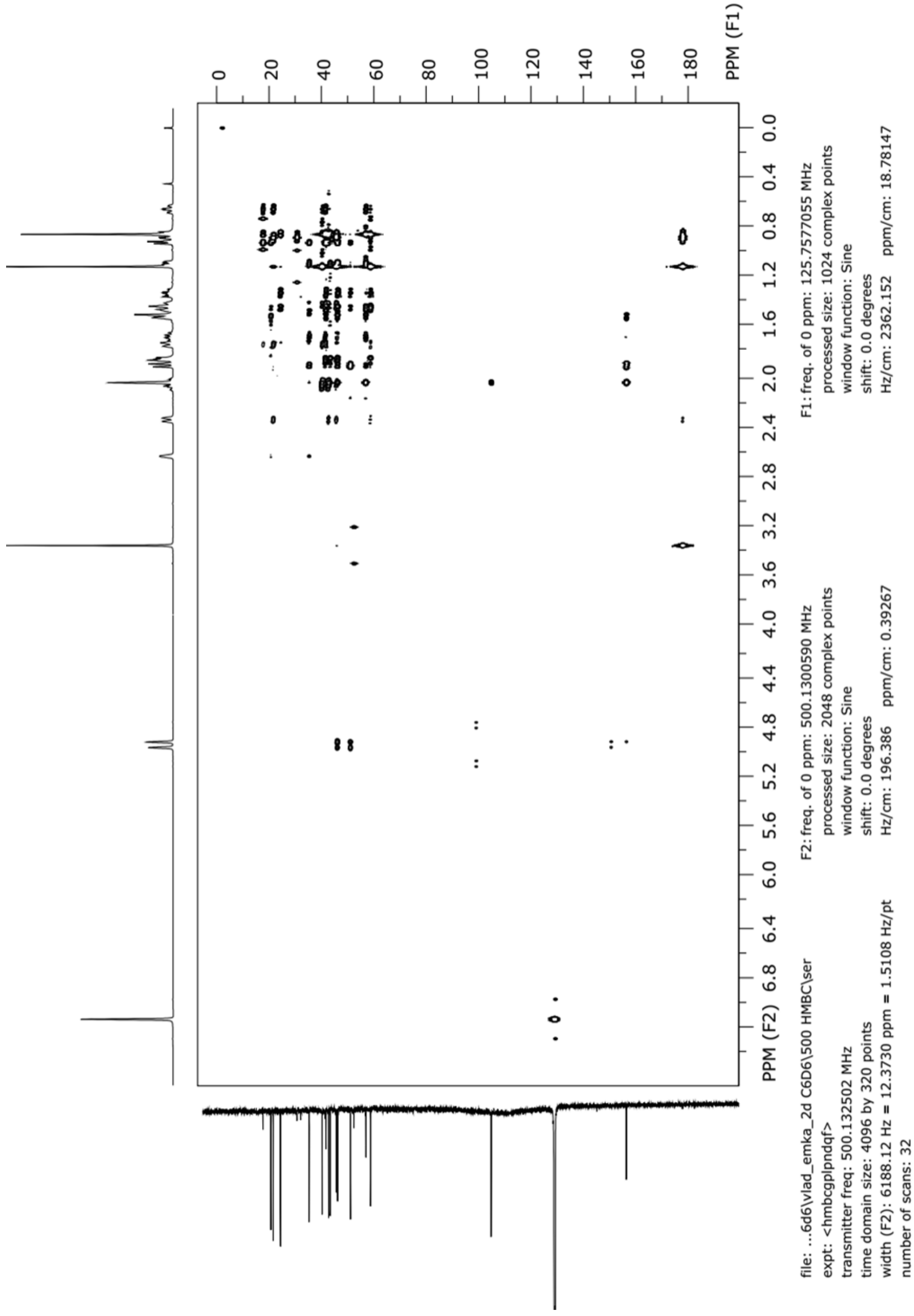

Figure S123. HMBC spectrum of methyl ent-kaur-16-en-19-oate – C_6D_6 (^{13}C: 125 MHz, ^{1}H: 500 MHz).

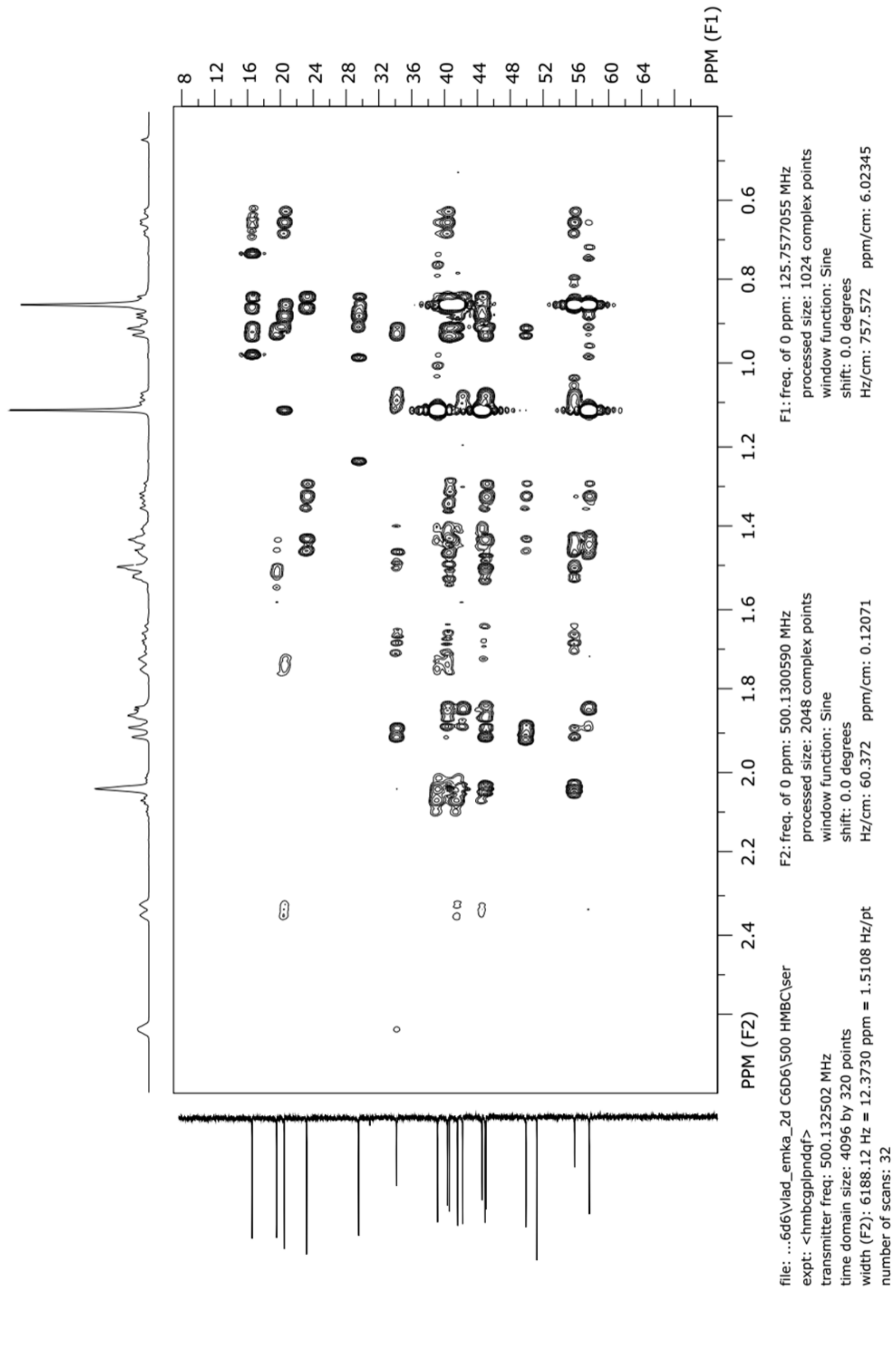

Figure S124. HMBC expansion spectrum of methyl ent-kaur-16-en-19-oate – C_6D_6 (^{13}C: 125 MHz, ^{1}H: 500 MHz).

IV. DATA COMPARISON SECTION

Table S5. ^{13}C-NMR data for *ent*-kaurenoic acid: $CDCl_3$; CD_3OD; C_6D_6; C_5D_5N and previously published literature [3] data for comparison.

C	$CDCl_3$ *δ $C^{Lit.}$(ppm)	$CDCl_3$ **δ$C^{Exp.}$(ppm)	CD_3OD **δ$C^{Exp.}$(ppm)	C_6D_6 **δ$C^{Exp.}$(ppm)	C_5D_5N **δ$C^{Exp.}$(ppm)
**1**	40.7	40.7	42.2	40.8	41.1
**2**	19.1	19.1	20.5	19.5	19.9
**3**	37.8	37.8	39.4	38.0	38.7
**4**	43.7	43.8	44.8	44.4	44.0
**5**	57.1	57.1	58.4	57.1	57.1
**6**	21.8	21.9	23.3	22.2	22.6
**7**	33.1	41.3	42.7	41.5	41.7
**8**	44.2	44.3	45.6	44.1	44.5
**9**	55.1	55.2	56.7	55.3	55.3
**10**	39.6	39.7	41.0	39.9	40.0
**11**	18.4	18.4	19.6	18.7	18.7
**12**	41.3	33.1	34.4	33.4	33.4
**13**	43.8	43.9	45.4	44.3	44.2
**14**	39.7	39.7	40.9	39.9	39.9
**15**	48.9	49.0	50.3	49.4	49.3
**16**	155.9	155.9	157.0	155.7	156.0
**17**	102.9	103.0	103.8	103.6	103.5
**18**	28.9	29.0	29.7	29.0	29.4
**19**	183.7	184.1	181.9	185.1	180.1
**20**	15.6	15.6	16.5	16.0	16.1

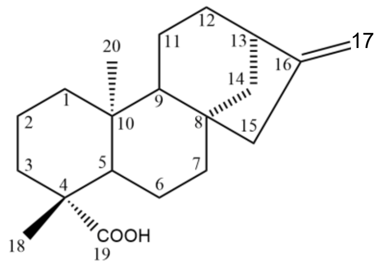

ent-kaurenoic acid

** Literature Data* [3]   *** This Work's Experimental Data*

Table S6. ^{13}C-NMR data for methyl ent-kaur-16-en-19-oate: $CDCl_3$ (100 MHz) e C_6D_6 (125 MHz); and previously published literature [2] data for comparison.

C	$CDCl_3$ *δ $C^{Lit.}$ (ppm)	$CDCl_3$ **δ $C^{Exp.}$ (ppm)	C_6D_6 **δ $C^{Exp.}$ (ppm)
**1**	40.8	40.7	41.3
**2**	19.2	19.1	20.0
**3**	38.2	38.1	38.7
**4**	43.9	43.9	44.3
**5**	57.1	57.1	57.5
**6**	21.9	21.9	22.8
**7**	41.3	41.2	41.9
**8**	44.2	44.2	44.7
**9**	55.1	55.1	55.7
**10**	39.4	39.4	40.0
**11**	18.4	18.3	19.1
**12**	33.1	33.1	33.8
**13**	43.8	43.8	44.8
**14**	39.7	39.6	40.3
**15**	48.9	48.8	49.7
**16**	155.9	155.8	156.0
**17**	102.9	103.1	103.9
**18**	28.9	28.7	29.1
**19**	178.1	178.1	177.7
**20**	15.4	15.2	16.1
**21**	51.1	51.0	51.0

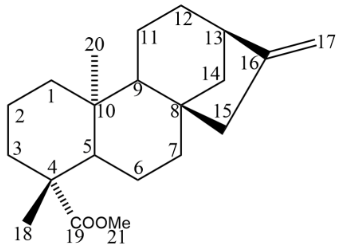

methyl ent-kaur-16-en-19-oate

* *Literature Data* [2]
** *This Work's Experimental Data*

Table S7. ^{1}H-NMR data for *ent*-kaurenoic acid: $CDCl_3$ (results in black); CD_3OD (results in red); C_6D_6 (results in blue) and C_5D_5N (results in green) - 500 MHz.

C	H	$CDCl_3$ *δH(ppm)	CD_3OD *δH(ppm)	C_6D_6 *δH(ppm)	C_5D_5N *δH(ppm)	*Coupling constants (Hz)	*Mult.
1	1α	1.88(1H)	1.89(1H)	1.71(1H)	1.85(1H)	J(1α,1β)=13.3;J(1α,2α)=3.9;J(1α,2β)=2.9; J(1α,3α)=1.8	*dddd*
	1β	0.81(1H)	0.84(1H)	0.60(1H)	0.82(1H)	J(1β,1α)=13.3;J(1β,2α)=13.3; J(1β,2β)=4.0	*td*
2	2α	1.84(1H)	1.93(1H)	2.05(1H)	2.27(1H)	J(2α,2β)=13.8;J(2α,1β)=13.3; J(2α,3β)=13.2;J(2α,1α)=3.9; J(2α,3α)=3.7	*ddddd*
	2β	1.44(1H)	1.46(1H)	1.38(1H)	1.52(1H)	J(2β,2α)=13.8;J(2β,3β)=4.4; J(2β,1β)=4.0;J(2β,1α)=2.9; J(2β,3α)=2.9	*dddt*
3	3α	2.16(1H)	2.13(1H)	2.28(1H)	2.48(1H)	J(3α,3β)=13.4;J(3α,2α)=3.7;J(3α,2β)=2.9; J(3α,1α)=1.8	*dddd*
	3β	1.01(1H)	1.01(1H)	0.83(1H)	1.08(1H)	J(3β,3α)=13.4;J(3β,2α)=13.2; J(3β,2β)=4.4	*ddd*
4	---	---	---	---	---		
5	5β	1.06(1H)	1.07(1H)	0.83 (1H)	1.06(1H)	J(5β,6α)=12.3; J(5β,6β)=2.2	*dd*
6	6α	1.81(1H)	1.85(1H)	1.94 (1H)	2.18(1H)	J(6α,6β)=13.8;J(6α,7β)=12.9;J(6α,5β)=12.3; J(6α,7α)=3.1	*dddd*
	6β	1.83(1H)	1.89(1H)	1.83 (1H)	2.04(1H)	J(6β,6α)=13.8;J(6β,7β)=3.7;J(6β,7α)=3.1; J(6β,5β)=2.2	*dddd*
7	7α	1.51(1H)	1.52(1H)	1.42(1H)	1.52(1H)	J(7α,7β)=13.1;J(7α,6α)=3.1;J(7α,6β)=3.1	*dt*
	7β	1.44(1H)	1.46(1H)	1.28(1H)	1.45(1H)	J(7β,7α)=13.1;J(7β,6α)=12.9;J(7β,6β)=3.7	*ddd*
8	---	---		---	---		
9	9β	1.07(1H)	1,08(1H)	0.89(1H)	1.05(1H)	J(9β,11α)=8.7;J(9β,14α)=1.4; J(9β,11β)=1.6	*ddd*
10	---	---	---	---	---		
11	11α	1.58(1H)	1.62(1H)	1.66(1H)	1.65(1H)	J(11α,11β)=13.5;J(11α;12α)=9.5; J(11α;12β)=8.7;J(11α;9β)=8.7	*ddt*
	11β	1.46(1H)	1.42(1H)	1.45(1H)	1.54(1H)	J(11β,11α)=13.5;*J*(11β,12β)=3.4; *J*(11β,12α)=2.6;*J*(11β,9β)=1.6	*dddd*
12	12α	1.60(1H)	1.64(1H)	1.50(1H)	1.53(1H)	J(12α,12β)=14.3; *J*(12α,11α)=9.5; *J*(12α,11β)=2.6;*J*(12α,13)=1.9	*dddd*
	12β	1.47(1H)	1.62(1H)	1.47(1H)	1.49(1H)	*J*(12β,12α)=14.3;*J*(12β,11α)=8.7; J(12β,11β)=3.4;*J*(12β,13α)=4.4;J(12β,14α)= 1.7	*ddddd*
13	13α	2.64(1H)	2.61(1H)	2.62(1H)	2.60(1H)	J(13α,14α)=5.0;J(13α,12α)=1.9 ;J(13α,12β)=4.4,J(13α,17a)=0.9; J(13α,17b)=0.9	*dddt*
14	14β	1.99(1H)	2.01(1H)	1.86(1H)	1.96(1H)	J(14β,14α)=11.4; J(14β,15β)=1.9	*dd*
	14α	1.14(1H)	1.12(1H)	1.06(1H)	1.06(1H)	J(14α,14β)=11.4;J(14α,13α)=5.0; J(14α,12β)= 1.7; J(14α, 9β)=1.4	*dddd*
15	15β	2.08(1H)	2.08(1H)	2.03(1H)	2.12(1H)	J(15β,15α)=16.8;J(15β,17a)=2.2; J(15β,17b) =2.2; J(15β,14β)=1.9	*dtd*
	15α	2.03(1H)	2,05(1H)	2.03(1H)	2.04(1H)	J(15α,15β)=16.8;J(15α,17a)=2.6; J(15α,17b)=2.6	*dt*
16	---	---	---	---	---		
17	17a	4.80(1H)	4.78(1H)	4.97(1H)	4.92(1H)	J(17a,15α)=2.6;J(17a,15β)=2.2; J(17a,17b)=1.5; J(17a,13α)=0.9	*dddd*
	17b	4.74(1H)	4.72(1H)	4.93(1H)	4.88(1H)	J(17b,15α)=2.6;J(17b,15β)=2.2; J(17b,17a)=1.5; J(17b,13α)=0.9	*dddd*
18	18β	1.24(3H)	1.18(3H)	1.11(3H)	1.36(3H)		*s*
19	---	---	---	---	---		
20	20α	0.95(3H)	0.98(3H)	1.00(3H)	1.15(3H)		*s*

** This Work's Experimental Data*

Table S8. ^{1}H-NMR data for methyl ent-kaur-16-en-19-oate: $CDCl_3$ – 400 MHz (results in black) and C_6D_6 (results in red) - 500 MHz.

C	H	$CDCl_3$ *δ H(ppm)	C_6D_6 *δ H(ppm)	Constantes de Acoplamento (Hz)	Multiplicidade
1	1α	1.88 (1H)	1.74 (1H)	J(1α,1β)=13.3;J(1α,2α)=3.9;J(1α,2β)=2.9; J(1α,3α)=1.8	*dddd*
	1β	0.80 (1H)	0.65 (1H)	J(1β,1α)=13.3;J(1β,2α)=13.3; J(1β,2β)=4.0	*td*
2	2α	1.85 (1H)	2.06 (1H)	J(2α,2β)=13.8;J(2α,1β)=13.3; J(2α,3β)=13.2;J(2α,1α)=3.9; J(2α,3α)=3.7	*ddddd*
	2β	1.43 (1H)	1.41 (1H)	J(2β,2α)=13.8;J(2β,3β)=4.4; J(2β,1β)=4.0;J(2β,1α)=2.9; J(2β,3α)=2.9	*dddt*
3	3α	2.17 (1H)	2.34 (1H)	J(3α,3β)=13.4;J(3α,2α)=3.7;J(3α,2β)=2.9; J(3α,1α)=1.8	*dddd*
	3β	1.00 (1H)	0.88 (1H)	J(3β,3α)=13.4;J(3β,2α)=13.2; J(3β,2β)=4.4	*ddd*
4	---				
5	5β	1.03 (1H)	0.85 (1H)	J(5β,6α)=12.3; J(5β,6β)=2.2	*dd*
6	6α	1.81 (1H)	1.86 (1H)	J(6α,6β)=13.8;J(6α,7β)=12.9;J(6α,5β)=12.3; J(6α,7α)=3.1	*dddd*
	6β	1.75 (1H)	1.84 (1H)		
7	7α	1.52 (1H)	1.45 (1H)	J(7α,7β)=13.1;J(7α,6α)=3.1; (7α,6β)=3.1	*dt*
	7β	1.45 (1H)	1.32 (1H)		
8	---				
9	9β	1.06 (1H)	0.92 (1H)	J(9β,11α)=8.7;J(9β,14α)=1.4; J(9β,11β)= 1.6	*ddd*
10	---				
11	11α	1.58 (1H)	1.68 (1H)	J(11α,11β)=13.5;J(11α;12α)=9.5; J(11α;12β)=8.7;J(11α;9β)=8.7	*ddt*
	11β		1.47 (1H)		
12	12α	1.63 (1H)	1.52 (1H)		
	12β	1.45 (1H)	1.49 (1H		
13	13α	2.64 (1H)	2.64 (1H)	J(13α,14α)=5.0;J(13α,12α)=1.9 ;J(13α,12β)=4.4,J(13α,17α)=0.9; J(13α,17β)=0.9	*dddt*
14	14β	1.97 (1H)	1.91 (1H)	J(14β,14α)=11.4; J(14β,15β)=1.9	*dd*
	14α	1.13 (1H)	1.09 (1H)	J(14α,14β)=11.4; J(14α,13α)= 5.0; J(14α, 12β)= 1,7; J(14α, 9β)= 1.4	*ddd*
15	15β	2.08 (1H)	2.07 (1H)	J(15β,15α)=16.8;J(15β,17a)=2.2; J(15β,17b)2.2; J(15β,14β)=1.9	*dtd*
	15α	2.02 (1H)	2.02 (1H)	J(15α,15β)=16.8;J(15α,17a)=2.6; J(15α,17b)=2.6	*dt*
16	---				
17	17a	4.79 (1H)	4.98 (1H)	J(17a,15α)=2.6;J(17a,15β)=2.2; J(17a,17b)=1.5; J(17a,13α)=0.9	*dddd*
	17b	4.74 (1H)	4.93 (1H)	J(17b,15α)=2.6;J(17b,15β)=2.2; J(17b,17a)=1.5; J(17b,13α)=0.9	*dddd*
18	18β	1.17 (3H)	1.11 (3H)		*s*
19	---				
20	20α	0.83 (3H)	0.85 (3H)		*s*
21	21α	3.64 (3H)	3.35 (3H)		*s*

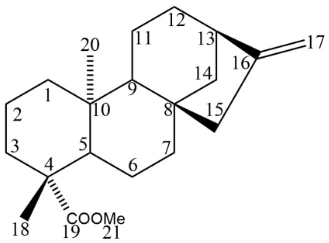

methyl ent-kaur-16-en-19-oate

** This Work's Experimental Data*

Table S9. Comparison of ^{1}H-NMR data presented in this work with the data found in literature for *ent*-kaurenoic acid.

Position	Hydrogen (500 MHz)	*δH ($CDCl_3$)	*δH (CD_3OD)	*δH (C_6D_6)	*δH (C_5D_5N)	Multiplicity and Coupling constant (Hz)	Ref. [4] (2020) $CDCl_3$ - 600 MHz
1	1α	1.88	1.89	1.71	1.85	dddd (13.3[1β]; 3.9[2α]; 2.9[2β]; 1.8[3α])	
	1β	0.81	0.84	0.60	0.82	td (13.3[1α]; 13.3[2α]; 4.0[2β])	
2	2α	1.84	1.93	2.05	2.27	ddddd (13.8[2β]; 13.3[1β]; 13.2[3β]; 3.9[1α]); 3.7 [3α])	
	2β	1.44	1.46	1.38	1.52	dddt (13.8[2α]; 4.4[3β]; 4.0[1β]; 2.9[1α]); 2.9[3α])	
3	3α	2.16	2.13	2.28	2.48	dddd (13.4[3β]; 3.7[2α]; 2.9[2β]; 1.8[1α])	
	3β	1.01	1.01	0.83	1.08	ddd (13.3[3α]; 13.2[2α]; 4.4[2β])	
4	---	-----	-----	-----	-----	-----	-----
5	5β	1.06	1.07	0.83	1.06	dd (12.3[6α]; 2.2[6β])	
6	6α	1.81	1.85	1.94	2.18	dddd (13.8[6β]; 12.9[7β]; 12.3[5β]; 3.1[7α])	
	6β	1.83	1.89	1.83	2.04	dddd (13.8[6α]; 3.7[7β]; 3.1[7α]; 2.2[5β])	
7	7α	1.51	1.52	1.42	1.52	dt (13.1[7β]; 3.1[6α]; 3.1[6β])	
	7β	1.44	1.46	1.28	1.45	ddd (13.1[7α]; 12.9[6α]; 3.7[6β])	
8	---	-----	-----	-----	-----	-----	-----
9	9β	1.07	1.08	0.89	1.05	ddd (8.7[11α]; 1.6[11β]; 1.4[14α])	
10	---	-----	-----	-----	-----	-----	-----
11	11α	1.58	1.62	1.66	1.65	ddt (13.5[11β]; 9.5[12α]; 8.7[12β]); 8.7[9β])	
	11β	1.46	1.42	1.45	1.54	dddd (13.5[11α]; 3.4[12β]; 2.6[12α]); 1.6[9β])	
12	12α	1.60	1.64	1.50	1.53	dddd (14.3[12β]; 9.5[11α]; 2.6[11β]); 1.9[13α])	
	12β	1.47	1.62	1.47	1.49	ddddd (14.3[12α]; 8.7[11α]; 3.4[11β]); 4.4[13α]; 1.7[14α])	
13	13α	2.64	2.61	2.62	2.60	dddt (5.0[14α]; 4.4[12β]); 1.9[12α]; 0.9[17a]; 0.9)[17b])	
14	14β	1.99	2.01	1.86	1.96	dd (11.4[14α]; 1.9[15β])	
	14α	1.14	1.12	1.06	1.06	dddd (11.4[14β]; 5.0[13α]; 1.7[12β]; 1.4[9β])	
15	15β	2.08	2.08	2.03	2.12	dtd (16.8[15α]; 2.2[17a]; 2.2[17b]; 1.9[14β])	
	15α	2.03	2.05	2.03	2.04	dt (16.8[15β]; 2.6[17a]; 2.6[17b])	
16	---	-----	-----	-----	------	-----	-----
17	17a	4.80	4.78	4.97	4.92	dddd (2.6[15α]; 2.2[15β]; 1.5[17b]; 0.9[13α])	4.81 brs
	17b	4.74	4.72	4.93	4.88	dddd (2.6[15α]; 2.2[15β]; 1.5[17a]; 0.9[13α])	4.75 brs
18	18β	1.24	1.18	1.11	1.36	s	1.25 s
19	---	-----	-----	-----	-----	-----	-----
20	20α	0.95	0.98	1.00	1.15	s	0.96 s

* *This Work's Experimental Data*

Table S10. Comparison of ^{1}H-NMR data presented in this work with the data found in literature for *ent*-kaurenoic acid -

Continuation

Posit.	^{1}H	Ref. [5] (2019) $CDCl_3$ - 300; 500 and 600 MHz	Ref. [6] (2018) $CDCl_3$ - 600 MHz	*Ref.* [7] (2018) CD_3OD - 600 MHz	Ref. [8] (2018) $CDCl_3$ - 400 MHz	Ref. [9] (2017) $CDCl_3$ - 300 MHz
**1**	**1α**			1.89 m		1.76 m
	**1β**	0.77 dt		0.84 m		0.75 ddd (13.0; 12.9;5.5)
**2**	**2α**			1.91 m		1.79 m
	**2β**			1.40 m		1.36 m
**3**	**3α**	2.13 d		2.13 brd (12.9)		2.06 m
	**3β**			1.01 m		0.94 m
**4**	**---**	-----	-----	-----	-----	-----
**5**	**5β**	1.07 m		1.07 m		1.03 m
**6**	**6α**			1.86 m		1.71 m
	**6β**			1.86 m		1.71 m
**7**	**7α**			1.50 m		
	**7β**			1.50 m		1.70 m
**8**	**---**	-----	-----	-----	-----	-----
**9**	**9β**	1.05 m		1.08 m		0,97 m
**10**	**---**	-----	-----	-----	-----	-----
**11**	**11α**			1.62 m		1.43 m
	**11β**			1.62 m		1.43 m
**12**	**12α**	1.59 m		1.63 m		1.38 m
	**12β**			1.46 m		1.47 m
**13**	**13α**	2.63 s	2.64 brs	2.61 m	2.63 br	2.56 brt
**14**	**14β**	1.97 dd		2.03 m		1.90 m
	**14α**	1.97 d		1.12 dd (11.6; 5.0)		1.09 m
**15**	**15β**	2.04 m		2.08 m		1.98 brs
	**15α**	2.04 m		2.01 m		1.98 brs
**16**	**---**	-----	-----	-----	-----	-----
**17**	**17a**	4.79 s	4.80 brs	4.78 brs	4.75 br	4.72 s
	**17b**	4.74 s	4,74 brs	4.72 brs	4.74 br	4.67 s
**18**	**18β**	1.24 s	1.24 s	1.18 s	1.24 s	1.25 s
**19**	**---**	-----	-----	-----	-----	-----
**20**	**20α**	0.95 s	0.95 s	0.98 s	0.95 s	0.92 s

Table S11. Comparison of ^{1}H-NMR data presented in this work with the data found in literature for *ent*-kaurenoic acid -

Continuation

Posit.	^{1}H	Ref. [10] (2016) $CDCl_3$ - 600 and 700 MHz	Ref. [11] (2015) $CDCl_3$ - 500 MHz	Ref. [12] (2013) $CDCl_3$ - 500	Ref. [13] (2012) $CDCl_3$ - 600 MHz
**1**	**1α**	1.88 m	1.88	1.54 m	1.87 m
	**1β**	0.81 m	0.82	0.82 m	0.80 dt (13.5; 4.0)
**2**	**2α**	1.87 m	1.86	1.42 m	1.87 m
	**2β**	1.45 m	1.42	1.42 m	1.42 m
**3**	**3α**	2.16 m	2.16	1.01 m	1.00 dt (13.5; 4.5)
	**3β**	1.01 m	1.02	2.17 m	2.15 d (14.5)
**4**	**---**	-----	-----	-----	-----
**5**	**5β**	1.06 m	1.06	1.07 m	1.06 m
**6**	**6α**	1.83 m	1.82	1.62 m	1.82 m
	**6β**	1.83 m	1.82	1.84 m	1.82 m
**7**	**7α**	1.52 m	1.47	1.44 m	1.52 dt (13.0; 3.5)
	**7β**	1.44 m	1.62	1.54 m	1.44 m
**8**	**---**	-----	-----	-----	-----
**9**	**9β**	1.06 m	1.04	1.05 m	1.04 d (7.0)
**10**	**---**	-----	-----	-----	-----
**11**	**11α**	1.56 m	1.56	1.60 m	1.59 m
	**11β**	1.56 m	1.60	1.88 m	1.55 m
**12**	**12α**	1.61 m	1.52	1.46 m	1.59 m
	**12β**	1.47 m	1.45	1.62 m	1.46 m
**13**	**13α**	2.63	2.63	2.61 brs	2.62 brs
**14**	**14β**	1.99 m	1.99	1.16	1.98 dd (11.5; 2.0)
	**14α**	1.14 m	1.13	2.02	1.13 dd (11.5; 5.0)
**15**	**15β**	2.06 m	2.05	2.06	2. 04 brs [H16]
	**15α**	2.04 m	2.05	2.06	2.04 brs [H16]
**16**	**---**	-----	-----	-----	-----
**17**	**17a**	4.80 m	4.81	4.72 s	4.78 brs
	**17b**	4.74 m	4.73	4.78 s	4.72 brs
**18**	**18β**	1.25 s	1.24	1.22 s	1.22 s [H19]
**19**	**---**	-----	-----	-----	-----
**20**	**20α**	0.95 s	0.95	0.93 s	0.93 s [H18]

Table S12. Comparison of ^{1}H-NMR data presented in this work with the data found in literature for *ent*-kaurenoic acid -

Continuation

Posit.	^{1}H	Ref. [14] (2001) $CDCl_3$ - 300MHz	Ref. [15] (2011) $CDCl_3$ - 600 MHz	Ref. [3] (1997) $CDCl_3$ - 400 and 500 MHz	Ref. [16] (1993) $CDCl_3$ - 300 MHz
**1**	**1α**		1.86	1.88	1.90
	**1β**		0.79	0.82	0.85
**2**	**2α**		1.87 m	1.86	1.90
	**2β**		1.41	1.42	1.45
**3**	**3α**		2.14 m	2.16	2.15
	**3β**		0,98	1.02	1.00
**4**	**---**	-----	-----	-----	-----
**5**	**5β**		1.04	1.06	1.05
**6**	**6α**		1.81	1.82	1.80
	**6β**		1.70 m	1.82	1.80
**7**	**7α**		1.48	1.47	1.50
	**7β**		1.40	1.62	1.50
**8**	**---**	-----	-----	-----	-----
**9**	**9β**		1.03	1.04	1.05
**10**	**---**	-----	-----	-----	-----
**11**	**11α**		1.60	1.60	1.60
	**11β**		1.54	1.56	1.60
**12**	**12α**		1.57	1.52	1.65
	**12β**		1.44	1.45	1.50
**13**	**13α**	2.62 brs	2.61 m	2.63	2.60
**14**	**14β**		1.97	1.99	1.95
	**14α**		1.11	1.13	1.15
**15**	**15β**	2.09 brs	2.03 m	2.05	2.00
	**15α**	2.09 brs	1.97	2.05	2.00
**16**	**---**	-----	-----	-----	-----
**17**	**17a**	4.79 brs	4.77 brs	4.80	4.90
	**17b**	4.79 brs	4.71 brs	4.73	4.80
**18**	**18β**	1.24 s	1.22 s	1.24	1.20
**19**	**---**	-----	-----	-----	-----
**20**	**20α**	0.95 s	0,93	0.95	0.95

Table S13. Comparison of ^{13}C-NMR data presented in this work with the data found in literature for *ent*-kaurenoic acid.

Carbon	*δC ($CDCl_3$)	*δC (CD_3OD)	*δC (C_6D_6)	*δC (C_5D_5N)	Ref. [4] (2020) $CDCl_3$ - 150 MHz	Ref. [5] (2019) $CDCl_3$ - 75; 125 and 150 MHz	Ref. [6] (2018) $CDCl_3$ - 150 MHz
**1**	40.7	42.2	40.8	41.1	40.7	40.9	41.3
**2**	19.1	20.5	19.5	19.9	19.1	19.3	19.1
**3**	37.8	39.4	38.0	38.7	37.8	37.9	37.8
**4**	43.8	44.8	44.4	44.0	43.7	43.9	44.2
**5**	57.1	58.4	57.1	57.1	57.0	57.2	57.0
**6**	21.9	23.3	22.2	22.6	21.8	21.9	21.8
**7**	41.3	42.7	41.5	41.7	41.2	41.4	40.7
**8**	44.3	45.6	44.1	44.5	44.2	44.4	39.6
**9**	55.2	56.7	55.3	55.3	55.1	55.3	55.9
**10**	39.7	41.0	39.9	40.0	39.6	39.9	43.7
**11**	18.4	19.6	18.7	18.7	18.4	18.6	18.4
**12**	33.1	34.4	33.4	33.4	33.1	33.3	**
**13**	43.9	45.4	44.3	44.2	43.8	44.0	43.8
**14**	39.7	40.9	39.9	39.9	39.7	39.8	39.7
**15**	49.0	50.3	49.4	49.3	48.9	49.2	48.9
**16**	155.9	157.0	155.7	156.0	155.8	156.0	155.9
**17**	103.0	103.8	103.6	103.5	102.9	103.2	103.0
**18**	29.0	29.7	29.0	29.4	28.9	29.1	29.0
**19**	184.1	181.9	185.1	180.1	184.2	184.7	184.7
**20**	15.6	16.5	16.0	16.1	15.5	15.8	15.6

** This Work's Experimental Data*
*** Not determined*

Table S14. Comparison of ^{13}C-NMR data presented in this work with the data found in literature for *ent*-kaurenoic acid – ***Continuation***

Carbon	Ref. [7] (2018) CD_3OD - 150 MHz	Ref. [8] (2018) $CDCl_3$ - 100 MHz	Ref. [9] (2017) $CDCl_3$ - 75 MHz	Ref. [10] (2016) $CDCl_3$ - 150 and 175 MHz
**1**	42.1	**	40.7	41.2
**2**	20.4	**	19.1	19.4
**3**	39.3	**	37.8	38.0
**4**	44.8	**	43.8	**
**5**	58.3	**	57.0	56.8
**6**	23.2	**	21.8	21.9
**7**	42.6	**	41.3	41.5
**8**	45.5	**	44.2	44.0
**9**	56.6	**	55.1	55.2
**10**	40.8	**	39.6	40.0
**11**	19.5	**	18.4	18.4
**12**	34.2	**	33.1	33.1
**13**	45.3	43.8	43.7	43.8
**14**	40.8	**	39.7	39.7
**15**	50.2	**	48.9	49.4
**16**	155.9	**	155.9	155.8
**17**	103.6	103.0	103.0	103.0
**18**	29.6	29.0	28.9	29.7
**19**	181.9	**	184.6	182.0
**20**	16.4	15.6	15.6	14.8

*** Not determined*

Table S15. Comparison of ^{13}C-NMR data presented in this work with the data found in literature for *ent*-kaurenoic acid – ***Continuation***

Carbon	Ref. [11] (2015) $CDCl_3$ - 125 MHz	Ref. [17] (2016) $CDCl_3$ - 200 MHz	Ref. [12] (2013) $CDCl_3$ - 150	Ref. [13] (2012) $CDCl_3$ - 150 MHz
**1**	40.7	40.9	41.4	40.7
**2**	19.1	19.3	19.3	19.1
**3**	37.8	38.1	37.9	37.8
**4**	43.7	43.9	43.9	43.7
**5**	56.9	57.2	57.3	57.0
**6**	21.8	22.0	22.0	21.8
**7**	33.1	41.5	40.9	41.3
**8**	44.2	44.4	44.1	44.2
**9**	55.1	55.3	55.3	55.1
**10**	39.7	39.9	39.9	39.6
**11**	18.4	18.6	18.7	18.4
**12**	41.3	33.3	33.9	33.1
**13**	43.9	44.1	43.9	43.8
**14**	39.8	39.9	39.9	39.7
**15**	48.9	49.2	48.5	155.9
**16**	155.9	156.1	156.1	48.9
**17**	103.1	103.2	103.2	102.9
**18**	183.8	183.8	29.2	15.6
**19**	28.9	29.2	184.9	28.9
**20**	15.6	15.8	15.8	183.9

Table S16. Comparison of ^{13}C-NMR data presented in this work with the data found in literature for *ent*-kaurenoic acid – ***Continuation***

**Carbon**	Ref. [15] (2011) $CDCl_3$ - 75 MHz	Ref. [3] (1997) $CDCl_3$ - 100 and 125 MHz	Ref. [16] (1993) $CDCl_3$ - 75 MHz
**1**	40.6	40.7	40.7
**2**	19.0	19.1	19.1
**3**	37.6	37.8	37.7
**4**	43.4	43.7	43.8
**5**	56.9	57.0	57.1
**6**	21.7	21.8	21.8
**7**	41.1	33.1	41.3
**8**	43.3	44.2	44.2
**9**	55.0	55.1	55.1
**10**	39.2	39.7	39.6
**11**	18.2	18.4	18.4
**12**	33.0	41.3	33.1
**13**	43.7	43.8	43.7
**14**	39.5	39.7	39.6
**15**	48.8	48.9	48.9
**16**	155.4	155.9	155.7
**17**	102.5	102.9	103.0
**18**	28.8	183.7	28.9
**19**	183.9	28.9	184.7
**20**	15.4	15.6	15.5

V. SUMMARY OF RESULTS

In this section, the ^{1}H NMR and ^{13}C {^{1}H} chemical shifts are summarized in the structural formulas of ent-kaurenoic acid and methyl ent-kaur-16-en-19-oate.

ent-kaurenoic acid -^{1}H RMN (500 MHz)

$CDCl_3$

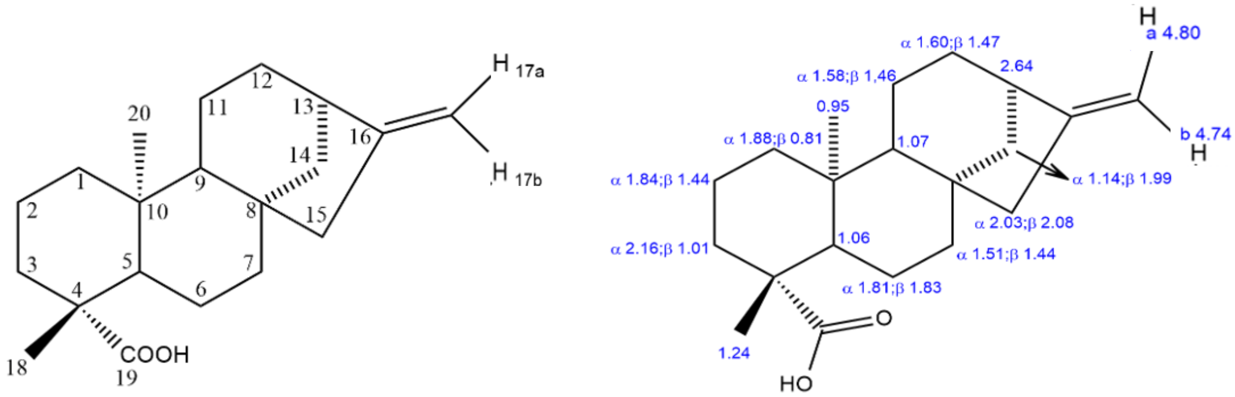

ent-kaurenoic acid -^{1}H RMN (500 MHz)

CD_3OD

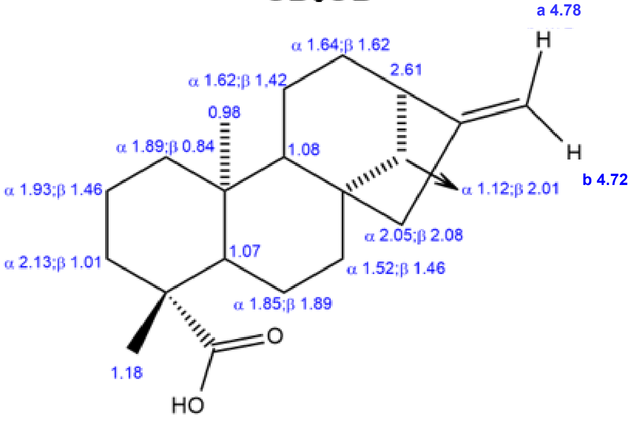

C_6D_6

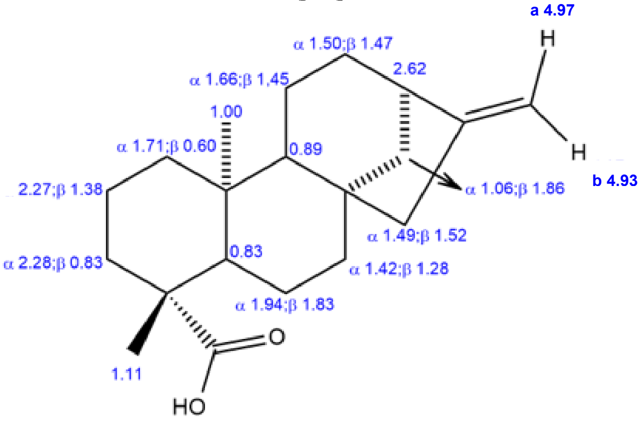

C_5D_5N

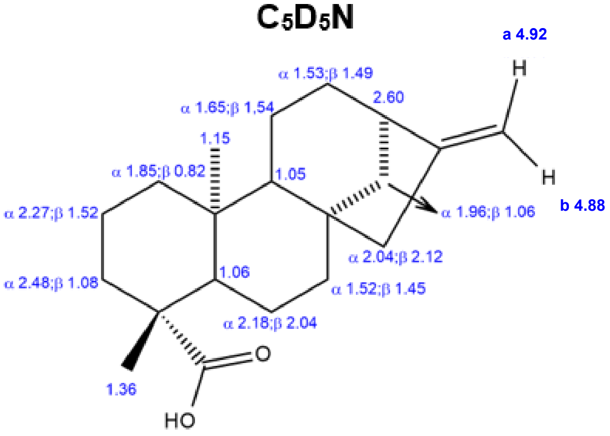

methyl ent-kaur-16-en-19-oate.

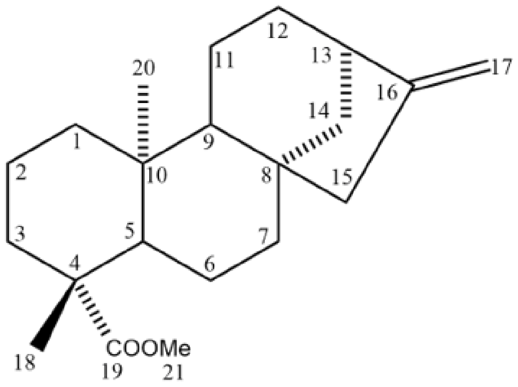

$CDCl_3$ ^{1}H-RMN (400 MHz)

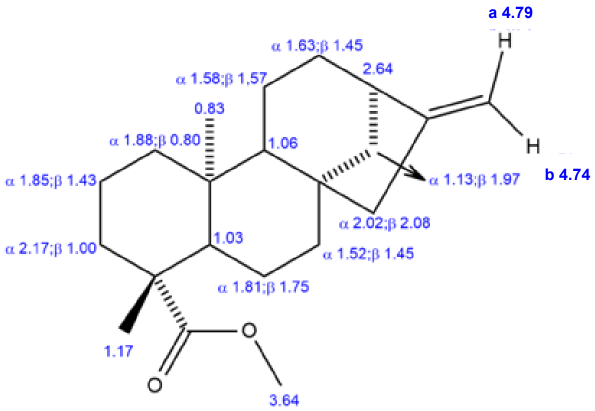

C_6D_6 ^{1}H-RMN (500 MHz)

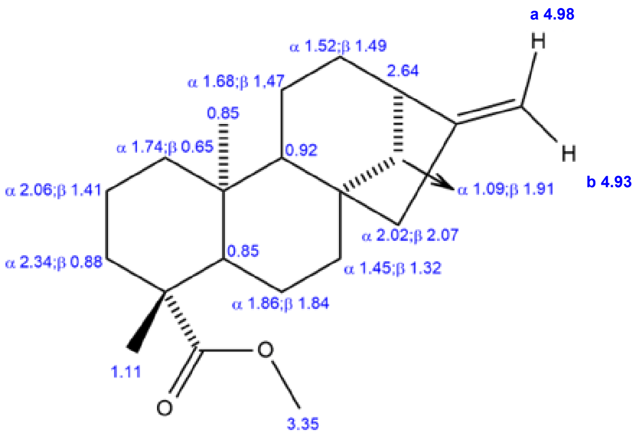

ent-kaurenoic acid ^{13}C {^{1}H} RMN (125 MHz)

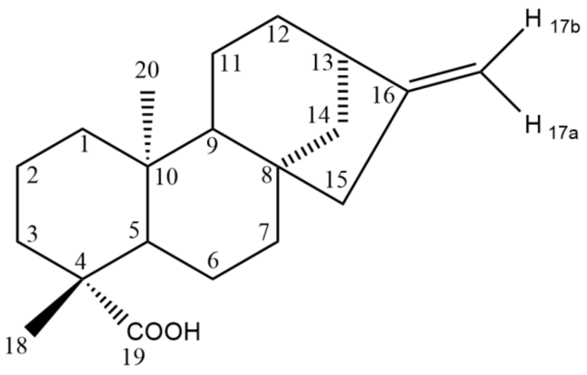

$CDCl_3$

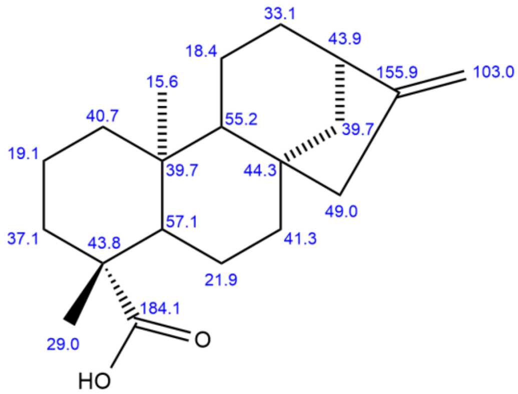

CD_3OD

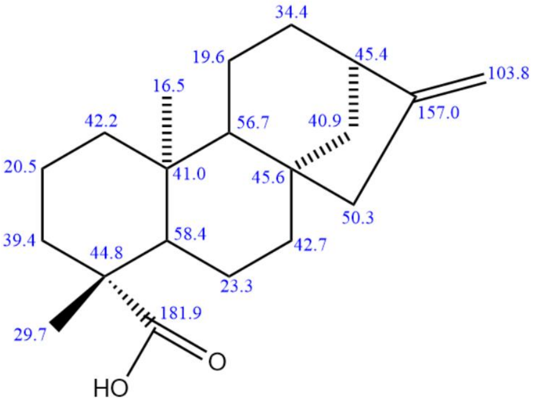

C_6D_6

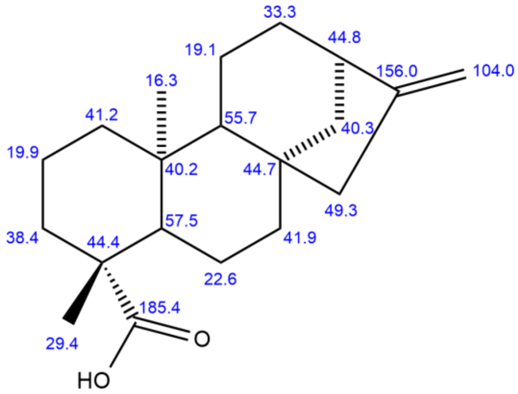

C_5D_5N

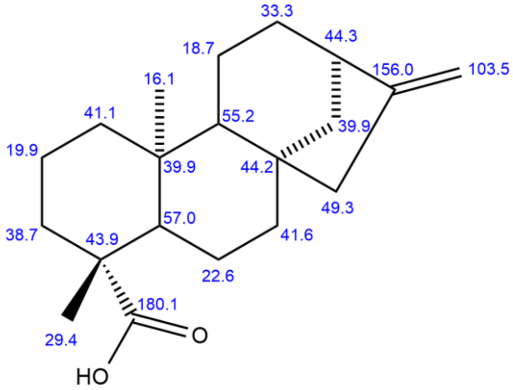

methyl ent-kaur-16-en-19-oate

$CDCl_3$ - ^{13}C-RMN (100 MHz)

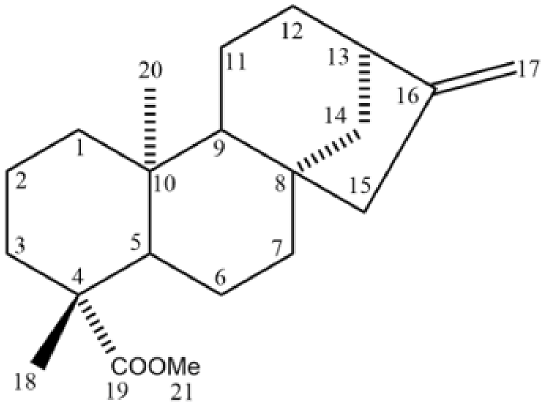

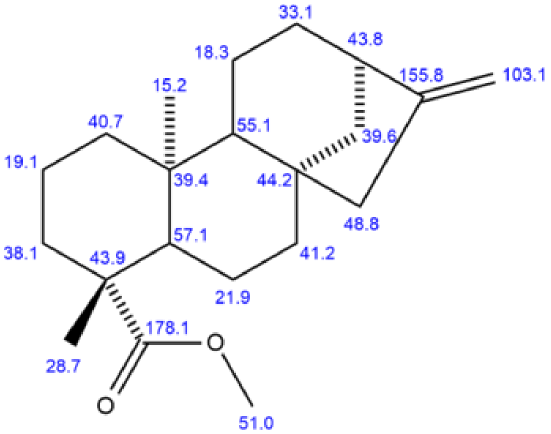

C_6D_6 - ^{13}C-RMN (125 MHz)

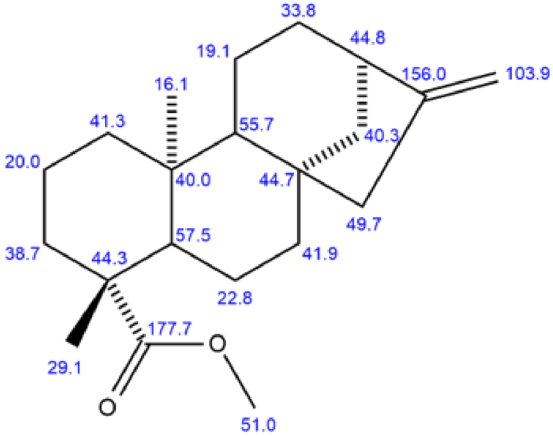

VI. TUTORIALS SECTION

In this section, a tutorial published by Ferreira and co-workers in an article titled “Software-assisted methodology for complete assignment of ^{1}H and ^{13}C NMR data of poorly functionalized molecules: The case of the chemical marker diterpene *ent*-copalic acid” will be made available. [18] *Ent*-copalic acid, or *ent*–labda-8(17)-13–dien–15-oic acid, (**Fig. 1**), is a perfect example of a poorly functionalized Natural Product displaying a highly overlapped ^{1}H NMR spectrum (**Fig. 1**).

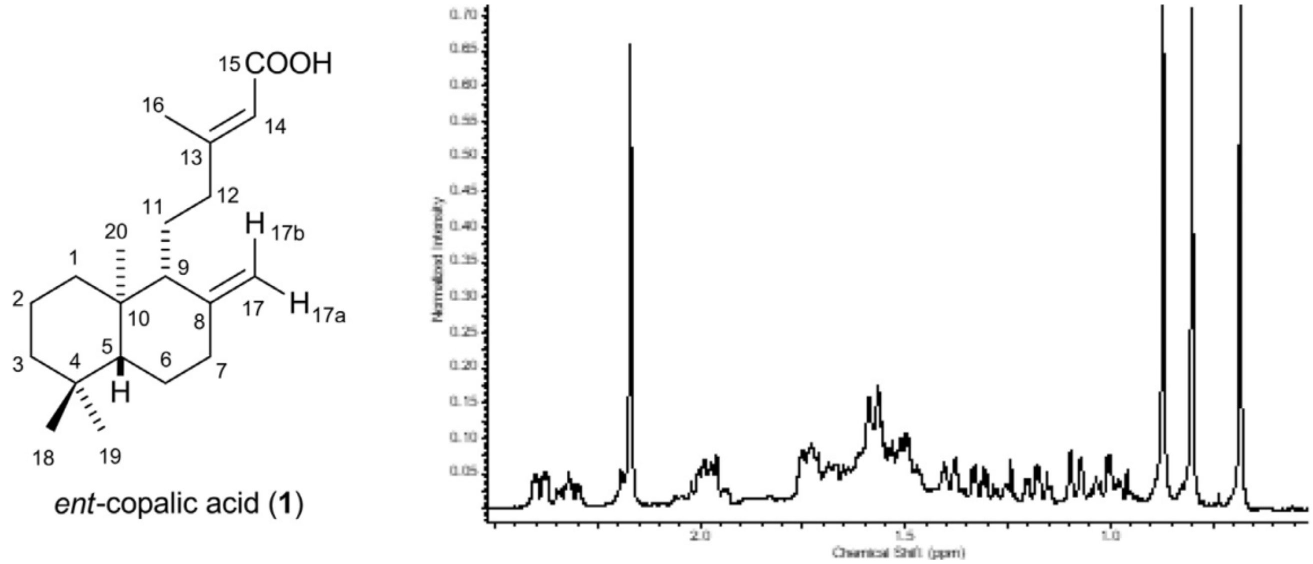

Fig. 1. *Ent*-copalic acid structure and the highly overlapped region in its ^{1}H NMR spectrum, between 0.9 and 2.42 ppm, where 19 hydrogen signals are involved. [18]

The TUTORIALS SECTION presents detailed tutorials of both programs, FOMSC3 and NMR_MultSim. A step-by-step usage guide is provided for each program to assist readers that might want to perform the software-assisted methodology here presented or want to use the calculation in some other problem. The two programs here presented were developed by Prof. Dr. Mauricio Gomes Constantino from FFCLRP – University of São Paulo (USP) – and are available at the webpage of the Synthetic Organic Chemistry Laboratory (LSO – Laboratório de Síntese Orgânica) of the Chemistry Department of FFCLRP - USP. The webpage http://artemis.ffclrp.usp.br/ includes several other available programs that were also developed for academic and scientific purposes, but not discussed here.

FOMSC3_rm_NB

This program, the most recent (2020) version of the original FOMSC3 (First-Order Multiplet Simulator/Checker), can calculate and plot NMR first order multiplets starting from information of chemical shifts (in Hertz) and coupling constant values. It can also plot the experimental spectrum on the same graph, thus providing a picture for visual comparison of calculated and experimental curves.

The initial screen of FOMSC3_rm_NB shows an example of a calculated signal, that of H7 of 15-deoxygoyazensolide, the signal for which this software was firstly designed (figure T1).

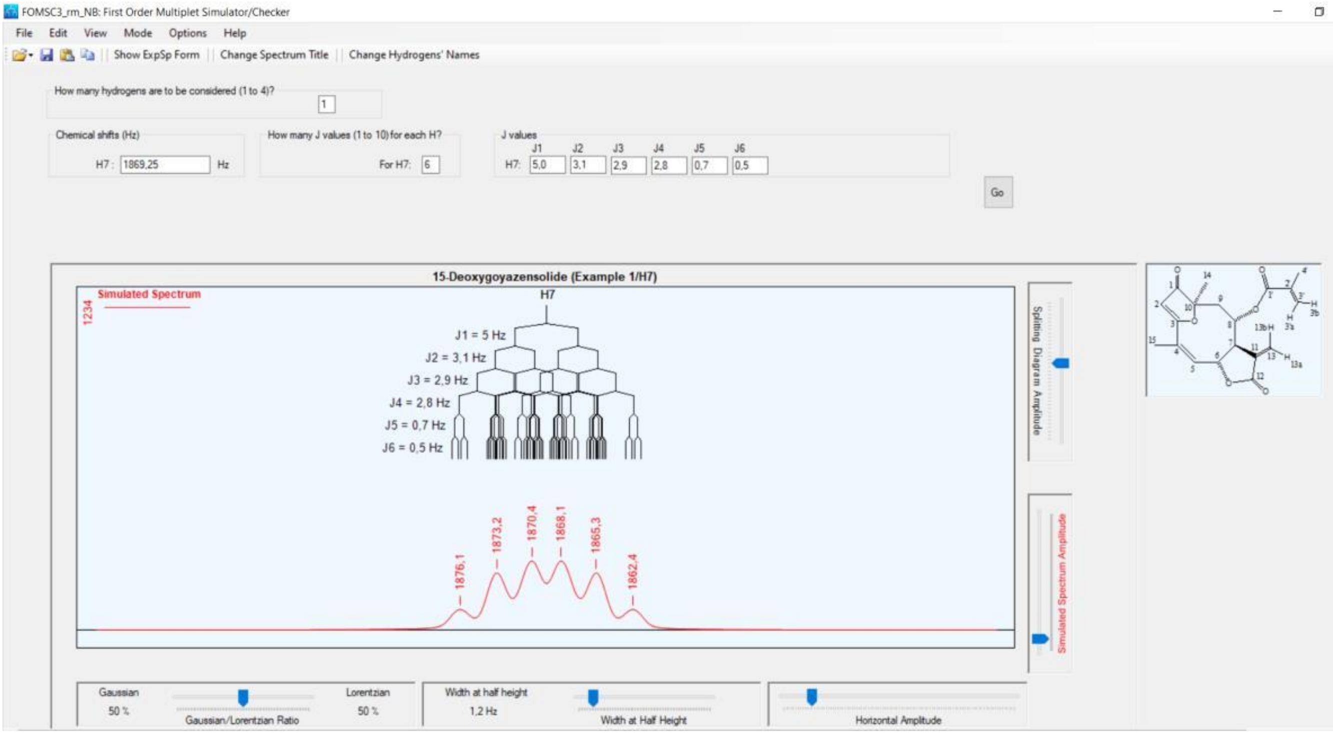

Figure T1: Initial screen of the FOMSC3_rm_NB.

When starting to use it, the user must exchange the data of the example that comes with the program with the information of the signals to be simulated. Firstly, the number of hydrogens that will be considered, which is up to four; followed by their chemical shift in Hz; then the number of *J* values for each hydrogen; and finally, all *J* values, as shown on figure T2. After filling the data, clicking on the “Go” button makes the software perform the calculation, immediately giving the result on screen, as done for H7a and H12a in our work (figure T3).

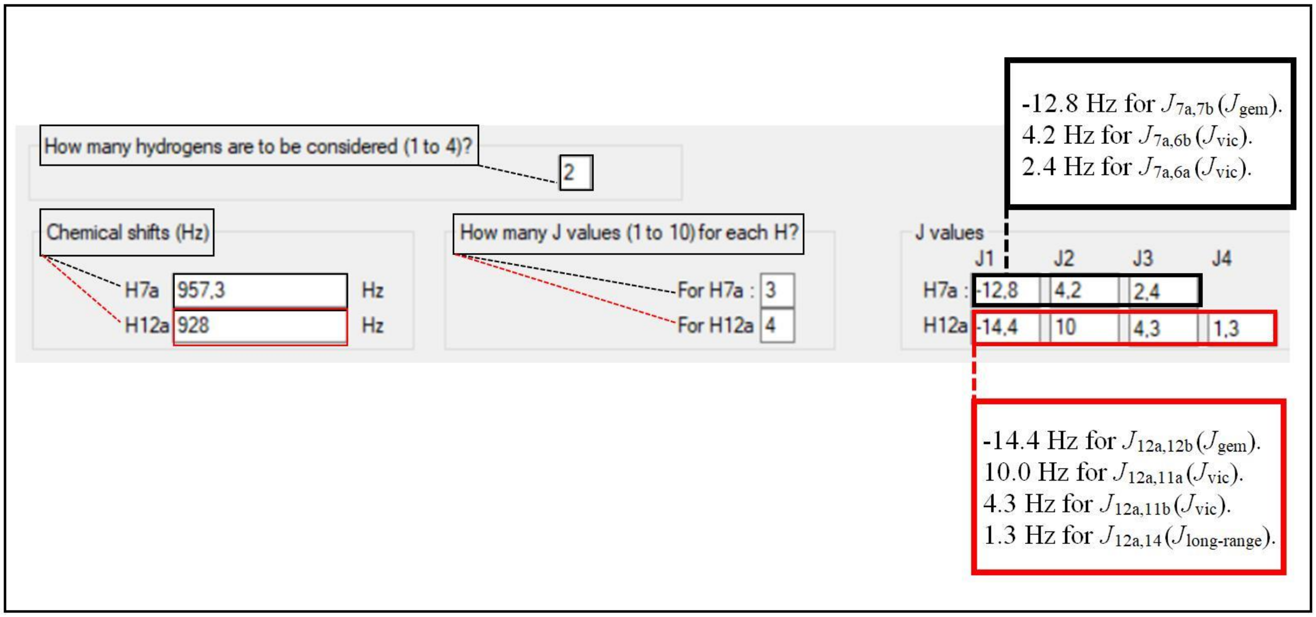

Figure T2 FOMSC3_rm_NB screen with highlights on what is requested.

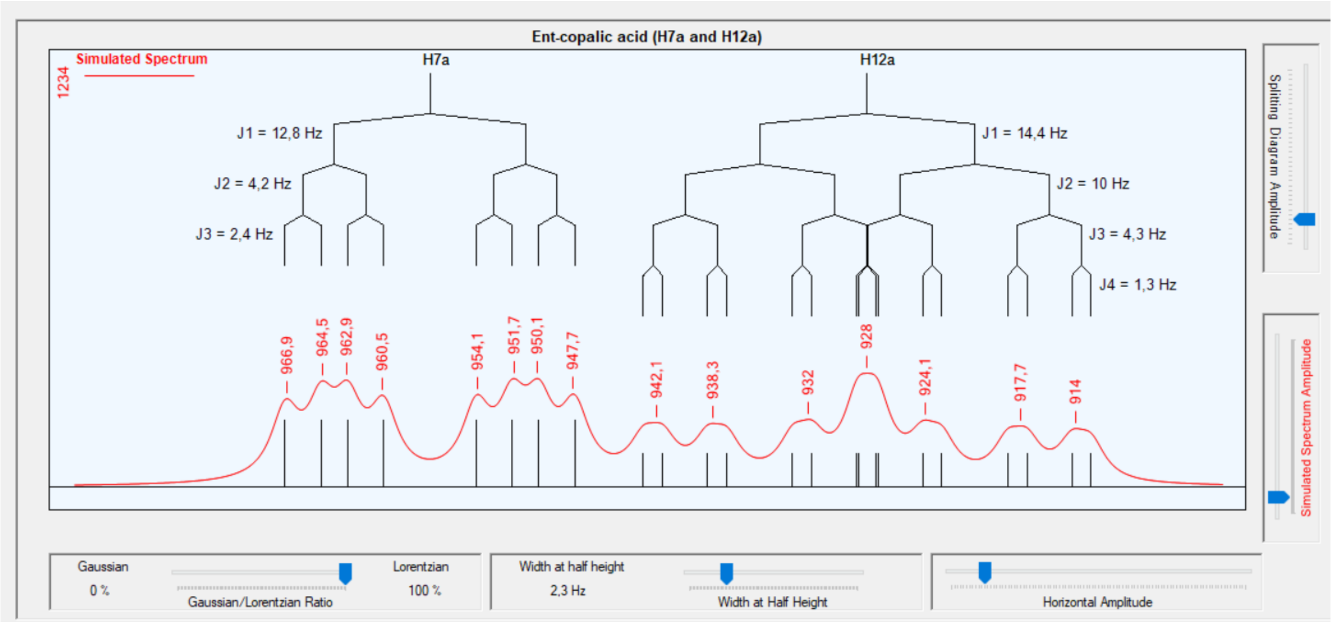

Figure T3: Screen of FOMSC3_rm_NB with the simulation for H7a and H12a signals in the ^{1}H-NMR spectrum for *ent*-copalic acid (**1**).

As it can be seen, the program prints, together with the signals, their splitting pattern diagrams also displaying the inserted *J* value in each case. In addition, H7a and H12a do not couple with each other, what turns their signals of first order and, thus, perfect to be calculated by this program.

Furthermore, for a better fitting between the experimental and calculated spectra appearance, the user is also allowed to control the shape of the envelope by changing the Lorentzian-Gaussian rate and/or the width at half height. Moreover, the user can also control horizontal and vertical amplitudes, separately for signal and splitting diagram, for better visualization of the results.

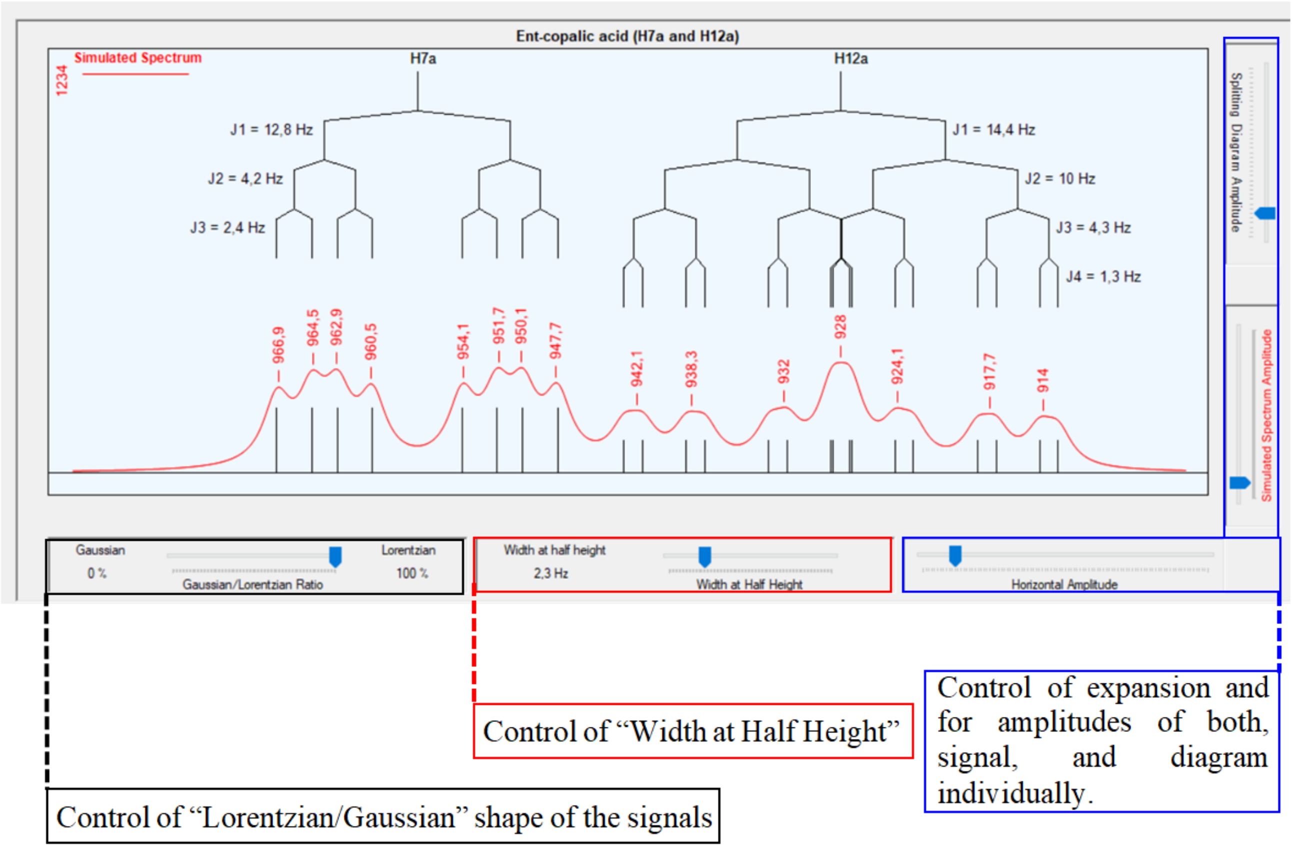

Figure T4: FOMSC3_rm_NB screen with results obtained and control of parameters.

When performing calculations with FOMSC3, the user is allowed to change the spectrum name and the hydrogens designations, by a fastly accessable menu on the top left side (figure T5- A). Furthermore, it is also possible to change the position of *J* lables in relation to the splitting diagram through the menu as shown on figure T5-B. The program is also full of options regarding what the user can view, as it can be seen on figure T5-D; the user can also easily copy the image of the results obtained (figure T5-C). After adjustments, clicking “Edit” and "Copy Simulated Spectrum Picture As Metafile" permits to copy the simulated spectra that can then be pasted into a new document for editing, as shown on figure T6.

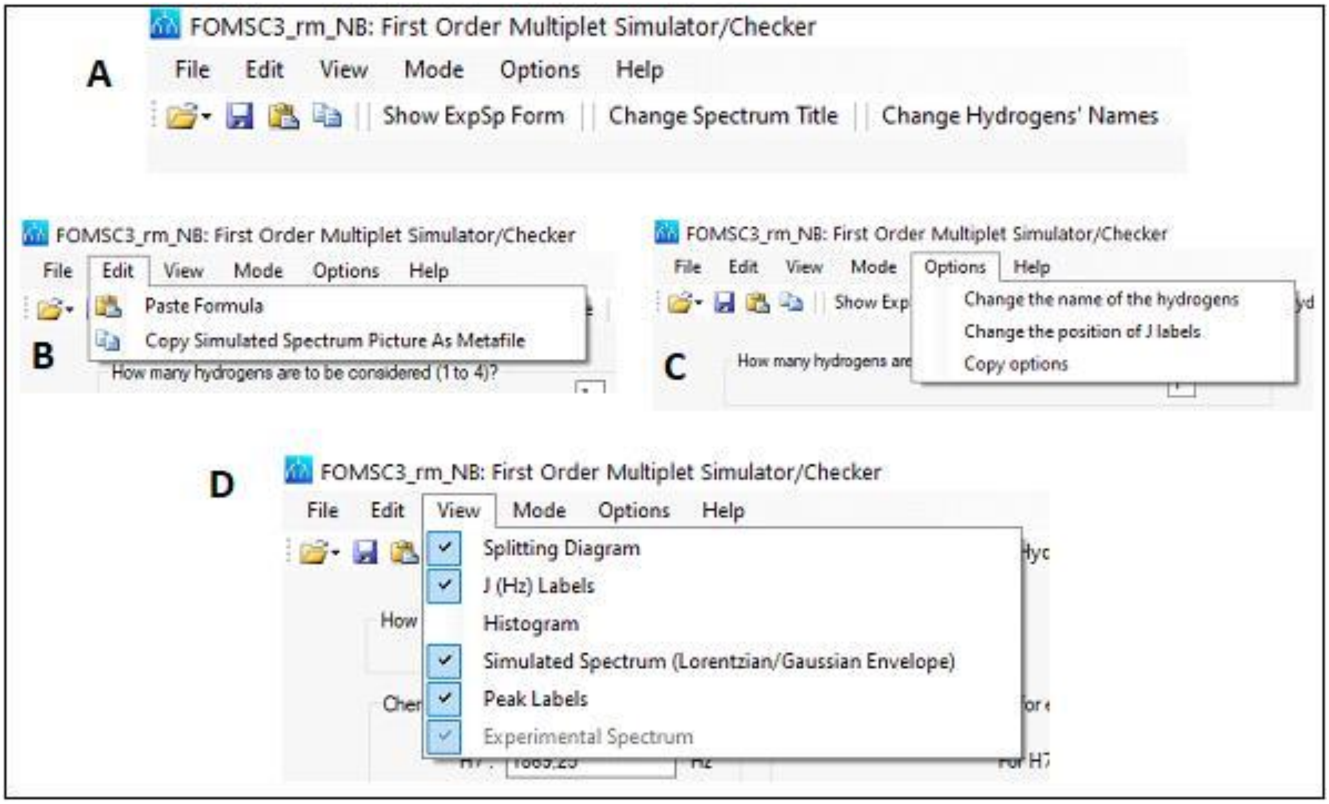

Figure T5: FOMSC3_rm_NB screen’s most important menus details.

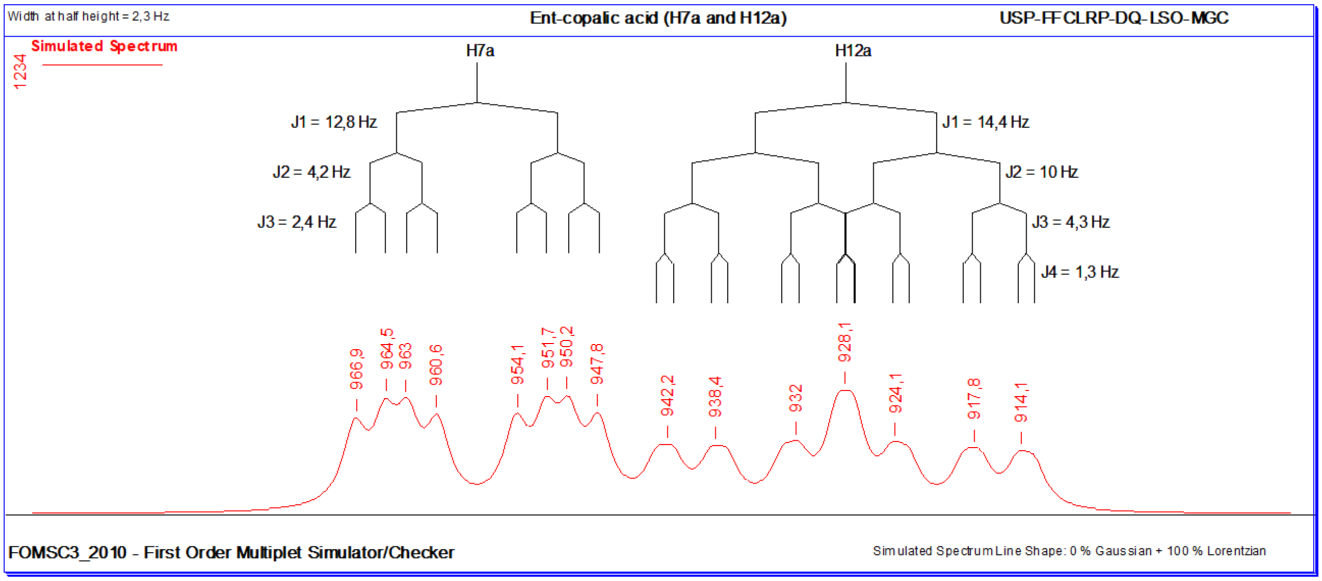

Figure T6: Image of the simulation for H7α and H12a pasted to Microsoft Word.

The pasted figure above (figure T6) shows the appearance of the signals, all individual peaks chemical shifts, and the coupling constant values together with the representation of the splitting pattern diagrams. After copying, the user can also transfer the image to an editing program together with experimental signals to generate a ploted figure of comparison, as what was made for hydrogens H7a and H12a from *ent*-copalic acid, shown on figure T7.

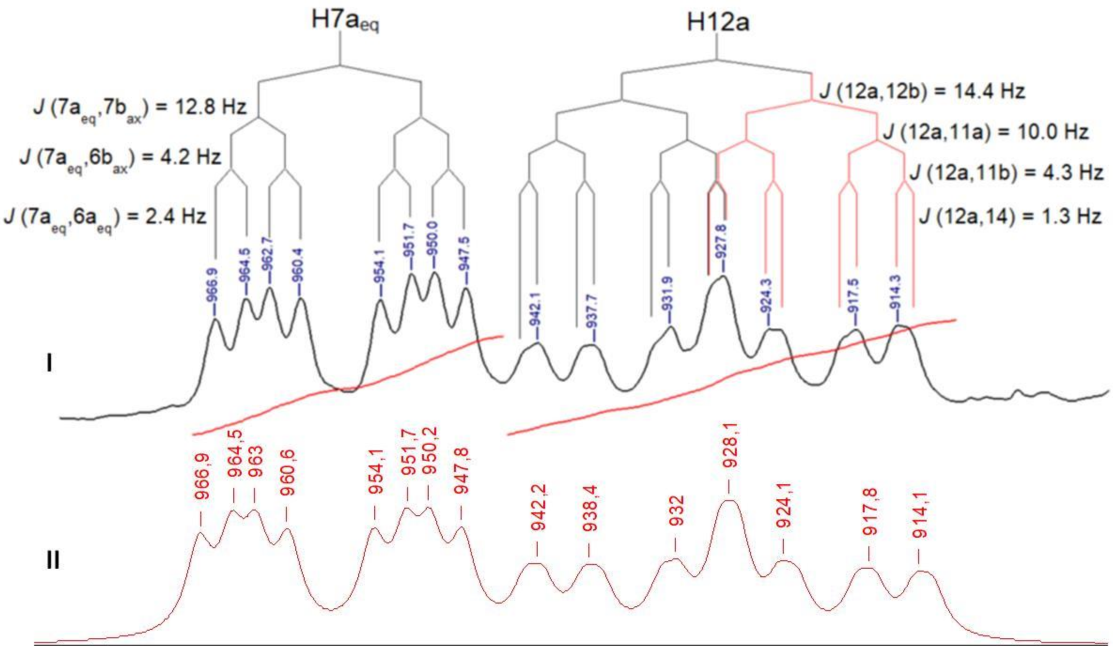

Figure T7: Experimental (**I**) and simulated signals (**II**) for H7α and H12a for *ent*-copalic acid.

The experimental/calculated comparison can also be done by FOMSC3_rm_NB, which offers the possibility of opening data from experimental NMR files (figure T8).

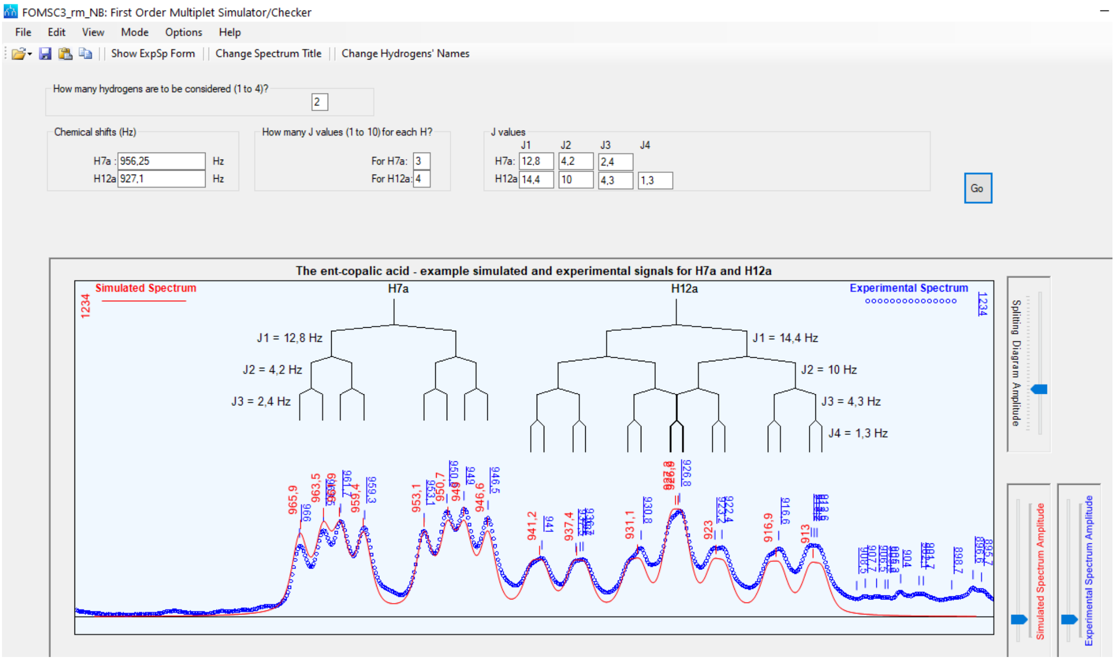

Figure T8: FOMSC3_rm_NB experimental (blue) and simulated (red) signals

comparison for H7α and H12a for *ent*-copalic acid.

This can be made through the “File” and “Open Experimental Spectrum” pathway. An excellent fitting between the simulated and the experimental spectra (figures T7 and T8), not only as a resemblance of visual appearance, but also due to high similarity between experimental and calculated individual peaks chemical shifts, can be seen. This proves that the *J* values used in the simulation are correct.

The best quality of this kind of simulation is the possibility to use the tool to confirm, refine and experimentally prove determined chemical shift values and coupling constants to help in laborious structural assignments.

Another kind of example is the H14 sign shown in figure T9, which is generated due to some long-range couplings, namely with H12 and H16. As a manner to state that all those couplings are really occurring and to determine their values, FOMSC3 can also be especially useful. *J*(14, 16) is easily measured in H16 signal, because it is a doublet. Moreover, due to the presence of a methyl group near H14 and considering the appearance of the H14 signal, it is easy to be tempted to consider the signal a low-resolution quartet (figure T9).

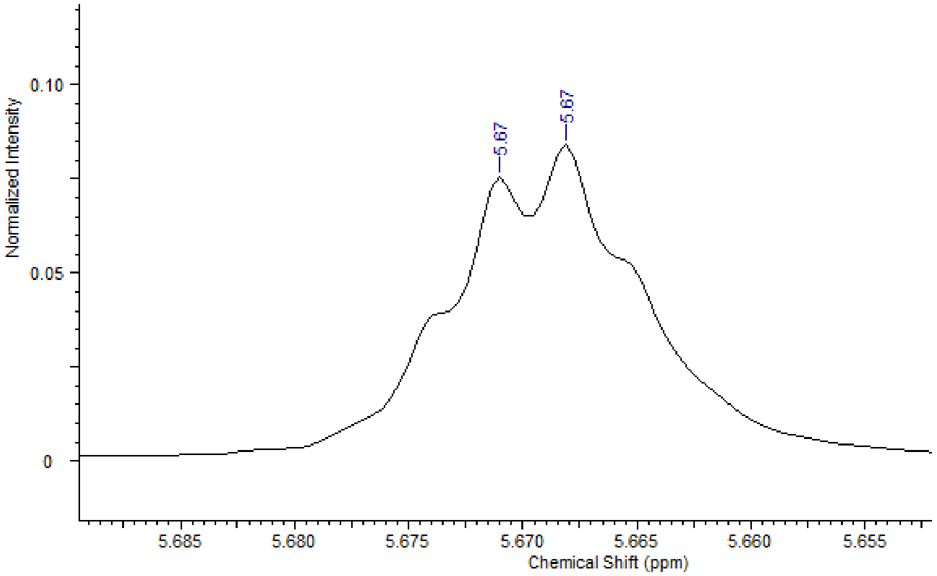

Figure T9: Experimental signal for H14 from *ent*-copalic acid.

For clarification of the H14 signal, *J*(14, 16) and *J*(14, 12a) measured before were used to estimate *J*(14, 12b). This was done using the difference between the *J* sum (signal width) and the sum of all known *J* values. Therewith, H14 signal started to be simulated performing some little adjustments. Total certainty was only reached after H12b studies, so the signal was simulated with the required information, as highlighted in figure T10. Following, simulated and experimental signals could be compared (figures T11 and T12).

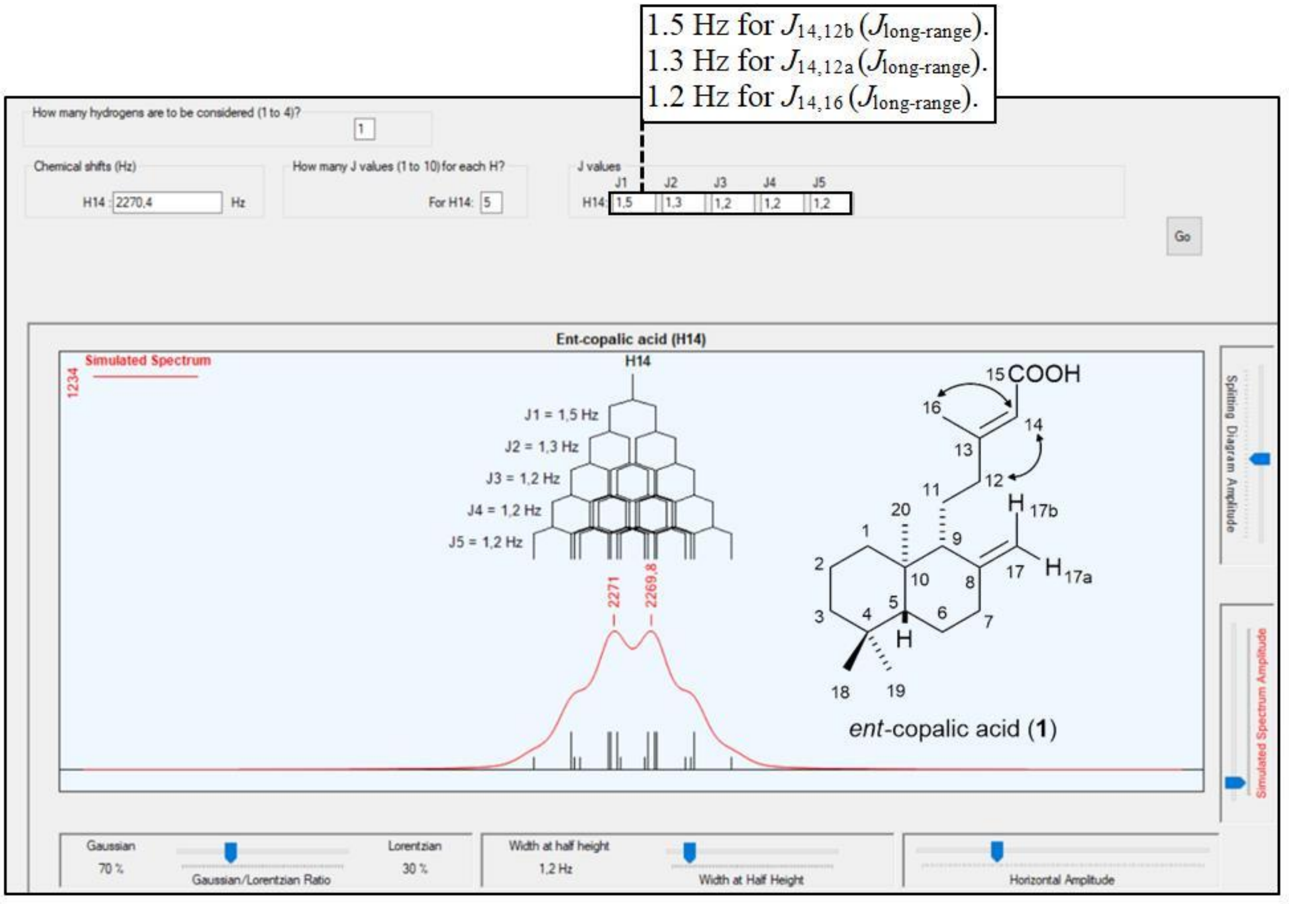

Figure T10: Simulated signal for H14 from *ent*-copalic acid.

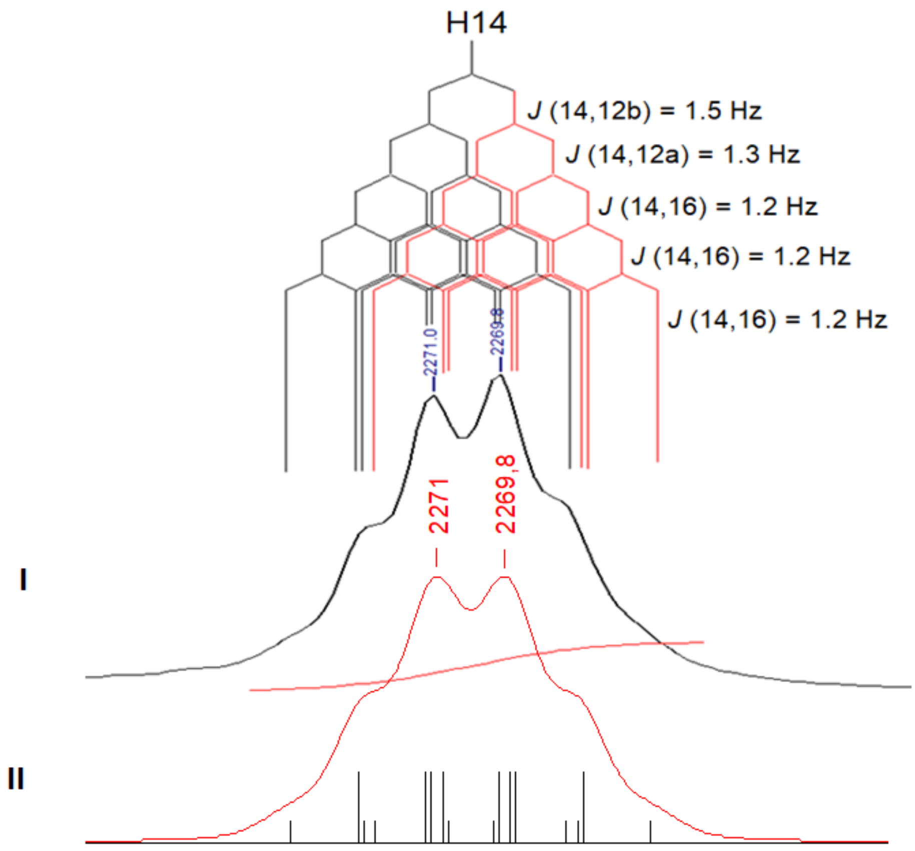

Figure T11: Comparison between experimental (I) and simulated (II) signals for H14.

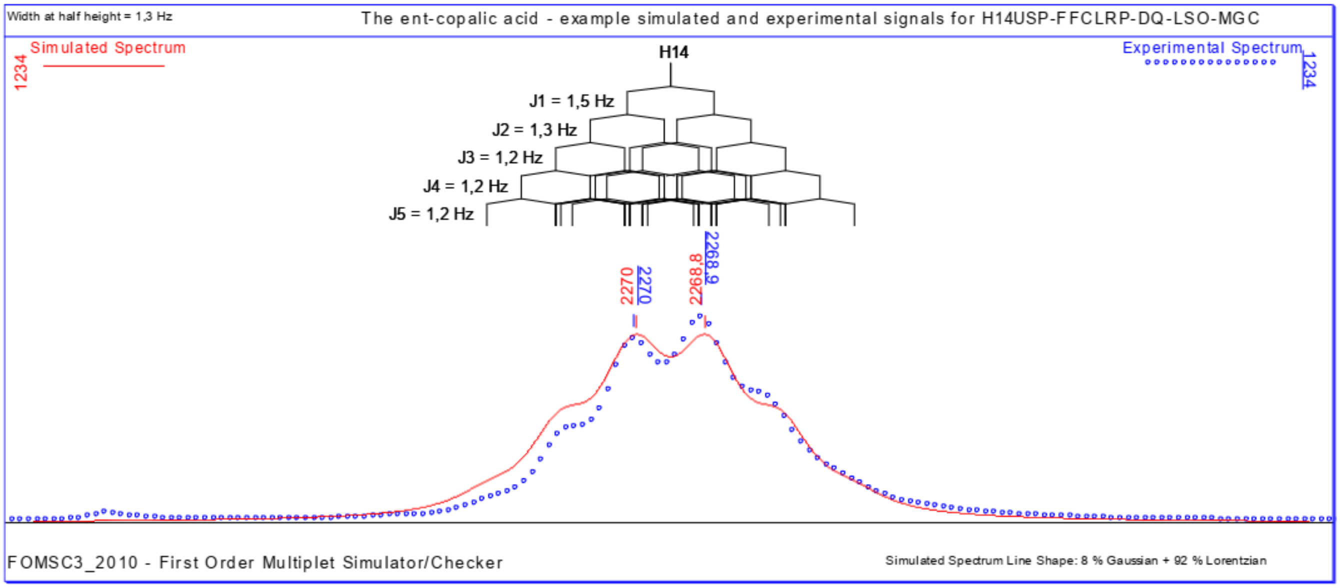

Figure T12: Comparison between experimental (blue) and simulated (red) signals for H14 in FOMSC3_rm_NB.

The almost perfect match between experimental and calculated spectra, including the individual peaks chemical shifts prove the reliability of determined *J* values. As it can be seen, the FOMSC3 program is an extremely helpful tool to study first-order spectrum signals; however, it is not appropiate for second order interactions cases, for which the NMR_ MultSim program is indicated.

NMR_MultSim_2011_1.4

The NMR_MultSim_2011_1.4 software is one of the versions of NMR_MultSim (Multiplet Simulator) and, as all other, can provide ^{1}H NMR subspectra (maximum 10 hydrogens) through quantum mechanical calculations, starting from arbitrary or experimentally determined values. These calculations are performed taking the chemical shift differences into account, to consider the phenomenon known as “second order distortions”, here designated as “second order modifications”. This latter determination is more suitable since these alterations

are not distortions but purely expected modifications due to high proximity of the chemical shifts from coupling hydrogens. On the other hand, the term “distortion” is more suitable to be used when the modification is caused by an unexpected problem and should not be observed in “normal” situations.

The program gives, after calculations, the resulting envelope curve according to distribution laws: either Gaussian or Lorentzian distribution, or even a variable combination of both, which can be selected by the user. Once all second order modifications are considered by the calculation methodology, NMR_MultiSim is perfectly suitable to allow accurate comparisons in second order signal cases. Nevertheless, NMR_MultiSim can also calculate first order signals through the same methodology, i.e. evaluating all chemical shifts distances. This has no implications to the result one can get with first order signals, and which is also good and reliable. On the other hand, the need of all coupled hydrogen chemical shifts to calculate one signal could turn the calculation a harder, or even impossible, task to accomplish. Sometimes, those chemical shifts are not possible to be measured, mainly in exceptionally overlapped and highly modified second-order spectra.

Here, a detailed tutorial of how to use this software to confirm and refine chemical-shift and coupling-constant values obtained from experimental spectra, applied to a poorly functionalized molecule, the *ent*-copalic acid, is described.

The same signal as the first example in FOMSC3_rm_NB will be used to show that NMR_MultSim also leads to good results in first order cases. The original signal of H7a and H12a is shown on figure T7-I.

In this signal of the *ent*-copalic acid spectrum, the integral values suggest that there are two hydrogens isolated from each other. The resolution of the signals in this spectrum

region made it possible to measure *J* values for simulation. The splitting diagram corresponding to the obtained data can also be seen on figure T7-I.

The insertion of data is slightly different in NMR_MultSim than in FOMSC3. The program also opens with an example and the information in forms must be changed to the information related to the signal that the user wants to calculate.

The initial screen shows the calculated spectrum in the lower half and the tables to insert data on the top higher half, as shown on figure T13.

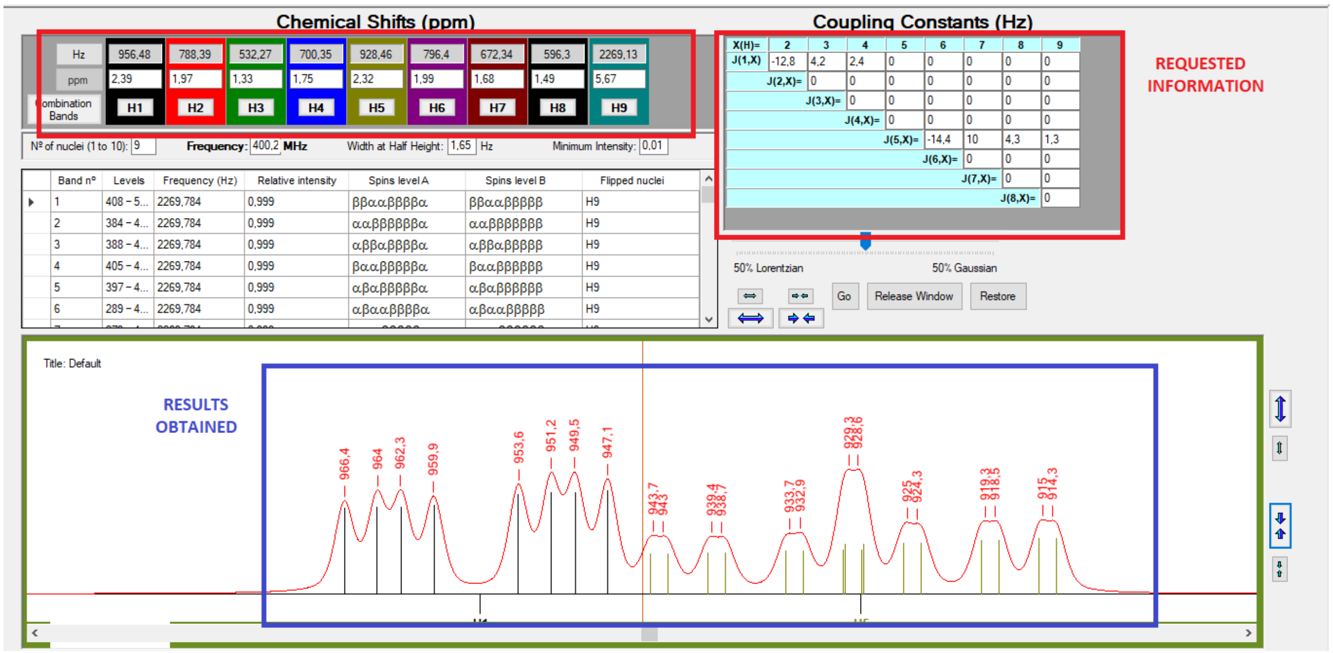

Figure T13: NMR_MultSim screenshot with highlights on what is requested and on what is obtained from the software, already with H7a and H12a data and simulated signals.

The figure above already shows the calculated H7a and H12a signals, and it will be now explained in detail how to get a desired simulation.

The first step is to insert the number of hydrogens involved, called “No of nuclei”, on the screen and which is limited up to 10 (figure T14). It must be stated that in NMR_MultSim,

the hydrogens considered involved are all simulated hydrogens, plus all hydrogens that couple with them. Thus, for simulation of H7a and H12a signals in FOMSC3, number 2 was inserted for hydrogens to be considered and in this software 9 was inserted as number of nuclei, because H7a couples with three other hydrogens and H12a with other four; total of nine hydrogens involved (see figure T14).

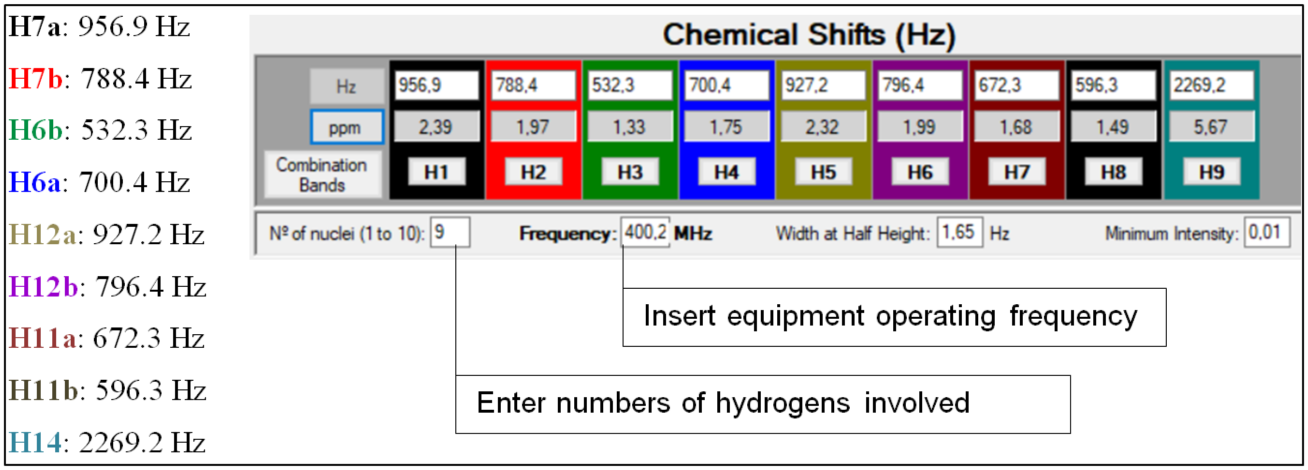

Figure T14: NMR_MultSim screen with initial data: chemical shifts, numbers of hydrogens and equipment operating frequency.

After that, it is necessary to insert the frequency of the equipment as well as all nine hydrogen chemical shifts, which can be in Hz or in ppm (figure T14).

The next set of information to be inserted consists of all the coupling constants for the signals to be calculated. This means, in this case, a total of seven *J* values: 3 for H7a and 4 for H12a, as shown on figure T15.

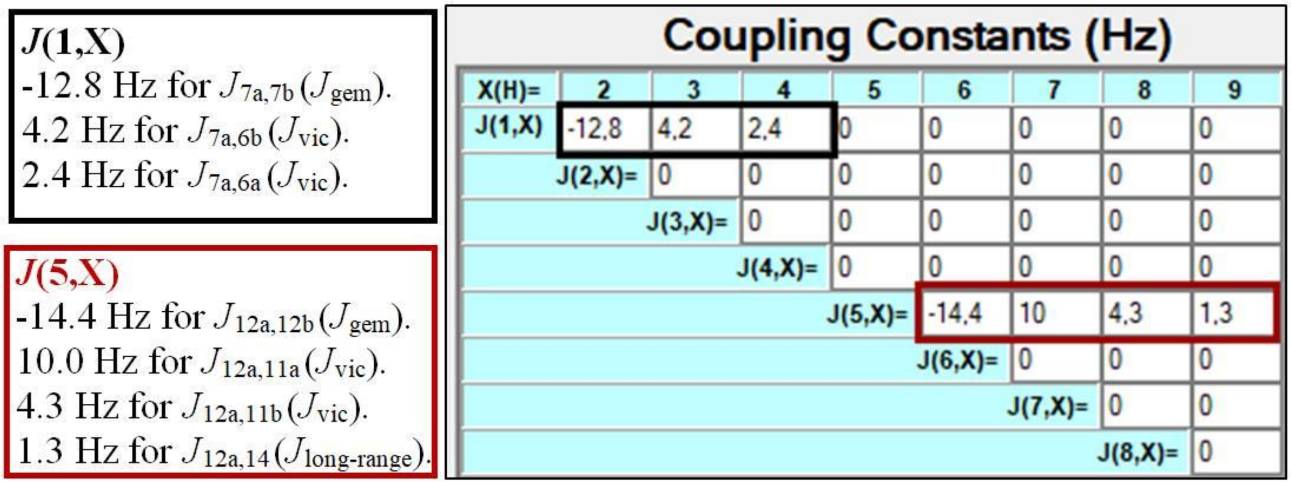

Figure T15: NMR_MultSim screen with H7a and H12a values of coupling constants.

With all this information inserted, the user can start the calculation by clicking the “Go” button. After a couple of seconds, the calculated spectrum is plotted on screen, just as shown on figure T13. The correct chemical shifts and *J* values are sufficient to get a simulated signal with a similar appearance to the experimental and practically identical individual peaks chemical shifts. Nevertheless, to get a more similar shape to the experimental envelope curve, it might be necessary to vary values like width at half height and minimum intensity (figure T14).

It is advisable to only make small changes, carefully observing the effect of each modification. The simulation shown on figure T13 was obtained after several modifications. Adjustments on chemical shifts and *J* values can be made by the same methodology. It is also possible to choose what to view in the simulated spectrum frame, such as peak labels, histogram, center reference line and other elements, as shown on figure T16.

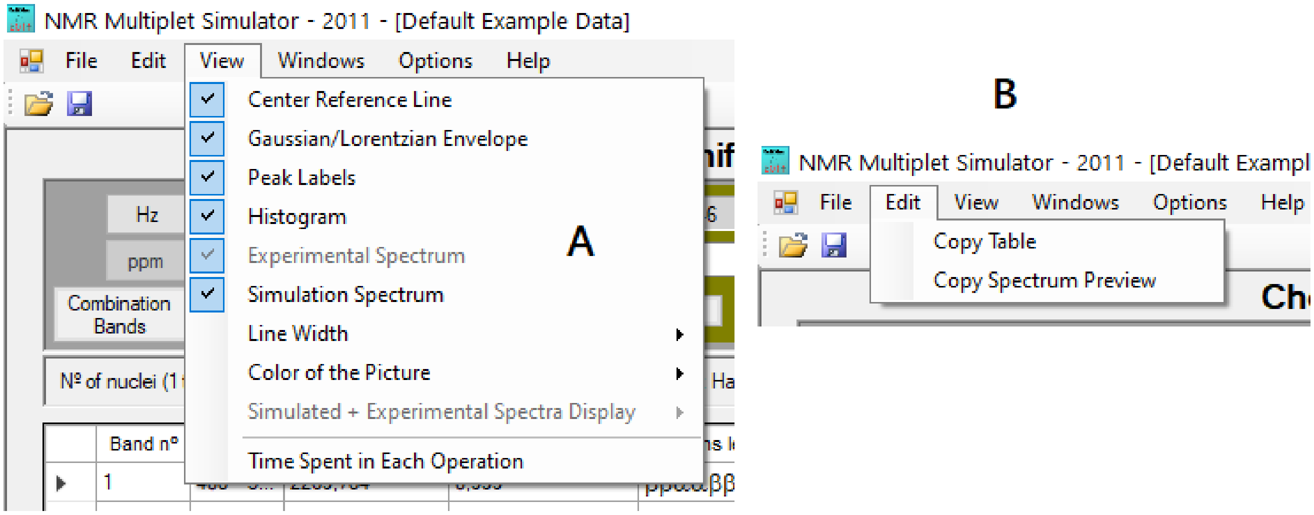

Figure T16: View and Edit menus in detail from NMR_MultSim.

To copy the simulation after several small adjustments, “Edit” and "Copy Spectrum Preview" must be clicked (figure T-13) and then pasted into a document for editing.

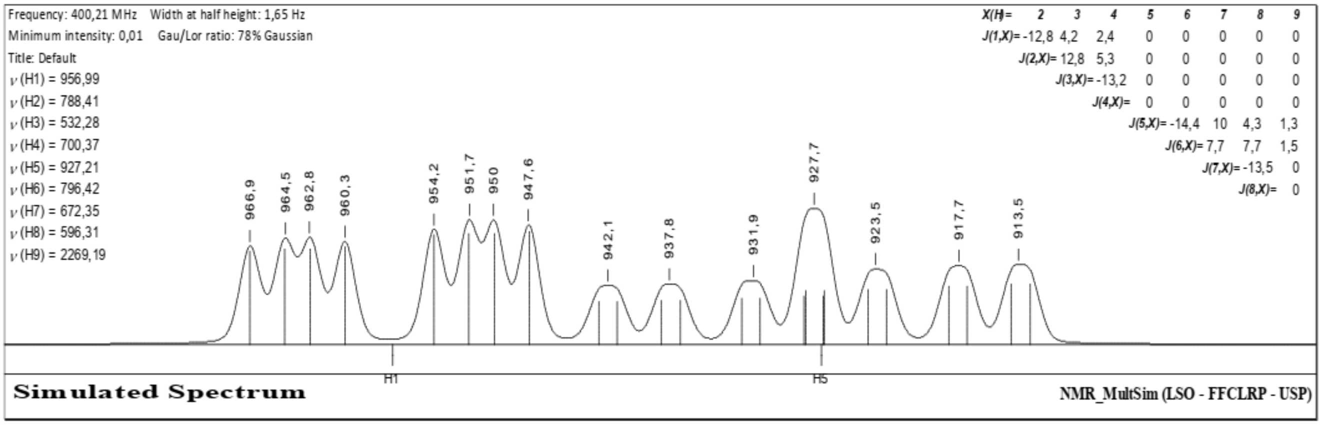

Figure T17: Simulation for H7α and H12a after several small adjustments, copied and pasted here.

Comparing the chemical shifts of this simulation with experimental values, it can be observed that H7α and H12a values are correct (FigureT18). The good correlation between the simulated and the experimental spectra (figure T18) shows that NMR_MultiSim is an appropriate tool to also simulate first order signals. However, these first order signals are more easily calculated with the FOMSC3-type programs (several versions).

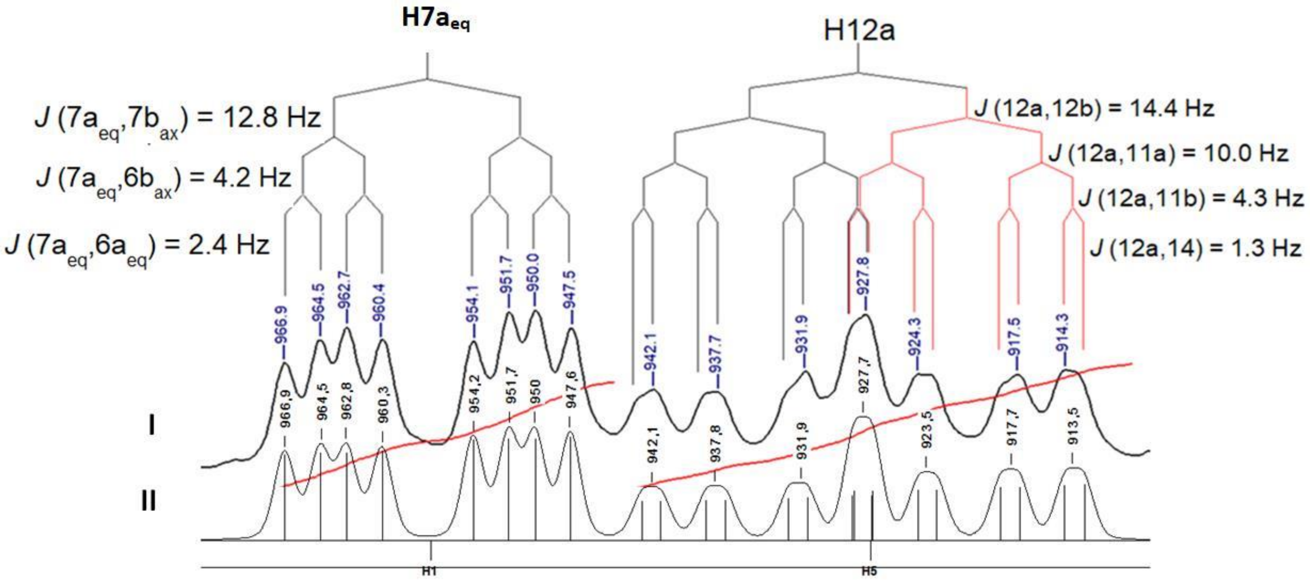

Figure T18: Experimental (**I**) and simulated signals (**II**) for H7α and H12a.

The NMR_MultSim is more necessary, and even indispensable, in other signals of our target molecule (*ent*-copalic acid) and in some other signals from poorly functionalized molecules, where a high level of signal overlapping and second order modifications are present.

As an example of those signal types, the simulation of H1a and H6a signals, which presents H1a, H6a and H11a overlapping and intense second-order interactions, will be shown here.

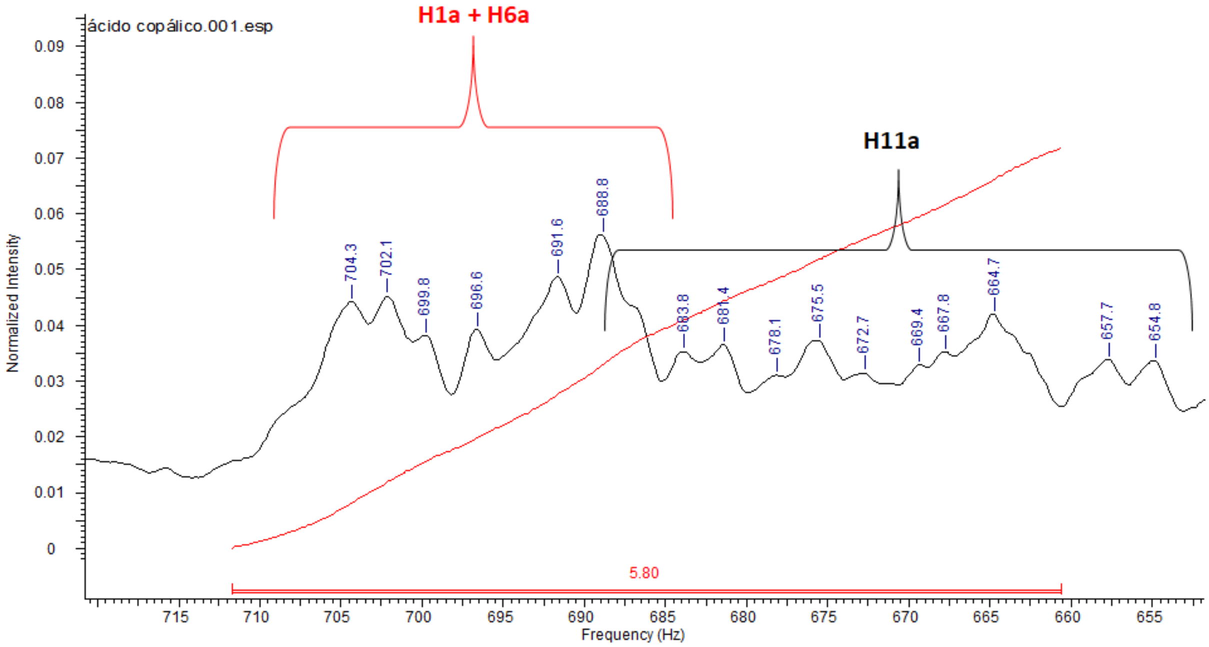

Figure T19: Experimental signals for H1a and H6a.

For simulation of this type of signal, as stated before, all involved chemical shifts are required. So, if H1a couples with H1b, H2a, H2b and H3a, while H6a couples with H6b, H5, H7a and H7b, the chemical shifts for those ten hydrogens (H1a, H6a and all eight hydrogens that couple with them) must be inserted into the program. For a previous calculation to verify the assigned coupling constants during the structural assignment process, the chemical shifts are enough. With all ten chemical shifts, in addition to the eight coupling constants involved with H1a and H6a, it is possible to calculate the signals. The obtained calculated signals, shown on figure T20, is close to the experimental, but not as good as that using all *J* values (figure T21).

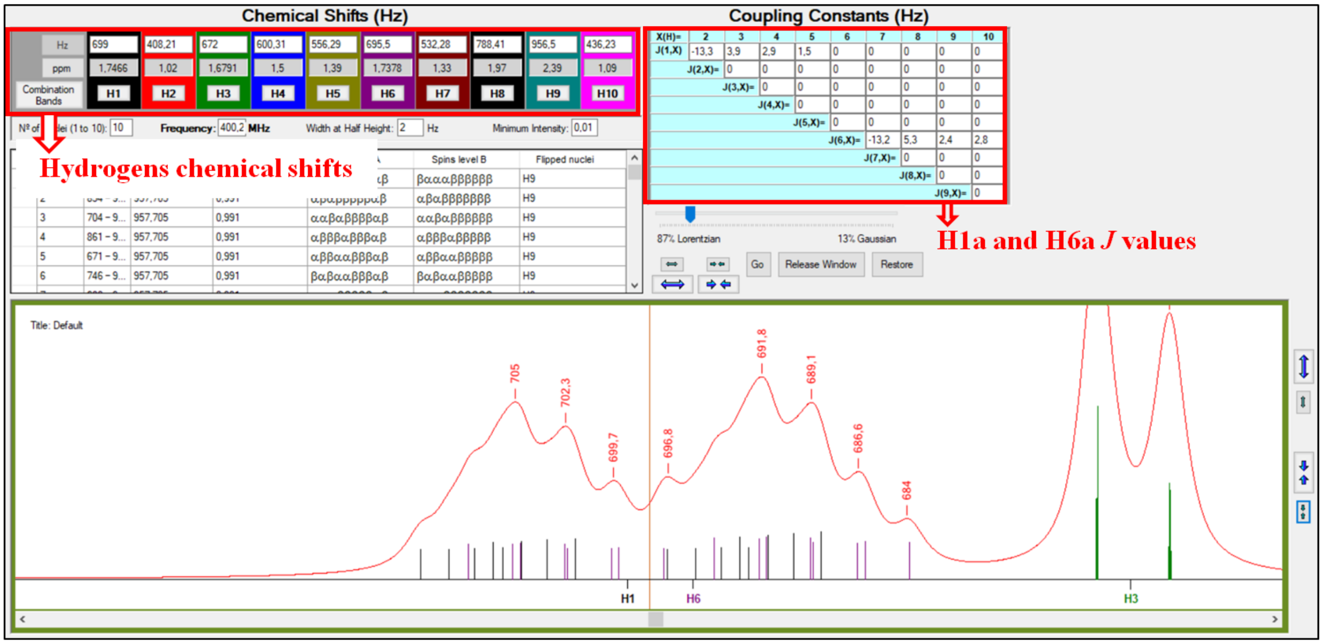

Figure T20: Calculated signals for H1a and H6a, NMR_MultiSim, with some *J* values.

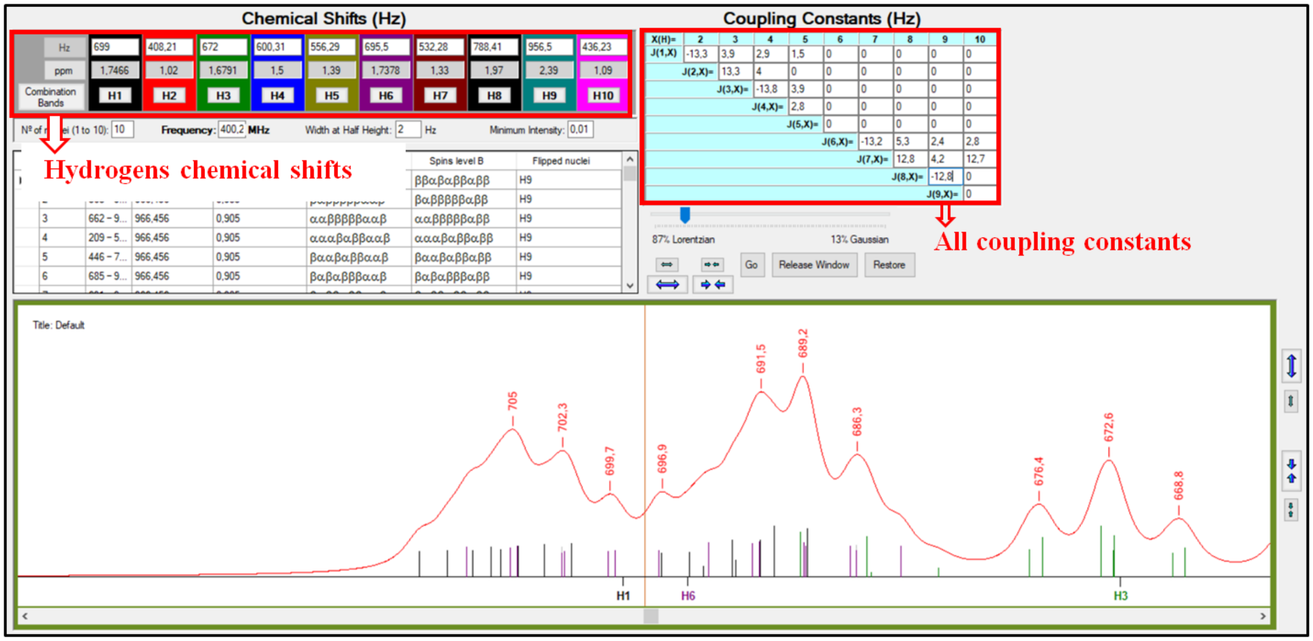

Figure T21: Calculated signals for H1a and H6a, NMR_MultiSim, with all *J* values.

Again, the “width at half height” and “minimum intensity” can be changed to verify

changes in spectrum appearance. Every time a value is changed, the software must calculate again, which is done by clicking the “Go” button. In addition, the signals of *J* values must also be verified. Only positive *J* values are used for first order spectra calculations. On the other hand, signals of *J*-values are considered for second order calculations. So, if there is a value of -12Hz for a coupling situation, the use of +12 Hz could lead to a different result; it is thus necessary to certify if the *J* value is positive or negative before using it in calculations with NMR Mult_Sim. Usually, geminal coupling constants are negative and vicinal are positive, but there can be different technical features [19–21].

Thus, experimental and calculated signals were compared by manual superposition (figure T22) and verification through the software resource, using the File-Open experimental spectrum menu, copying the *ent*-copalic acid experimental spectrum and comparing (figure T23).

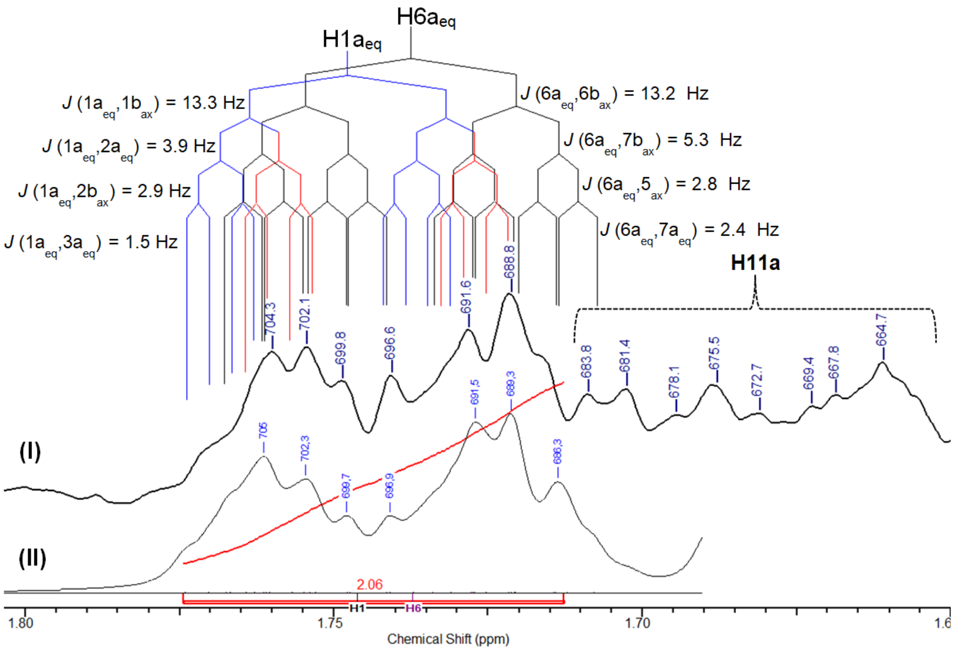

Figure T22: Comparison of experimental (A) and calculated (B) signals for H1a and H6a. Calculations done by NMR_MultiSim, and manual superpositions of both spectra.

Both comparisons, manual and through the NMR_MultiSim pathway, can be considered successful for determination of all coupling constants values for H1a and H6a hydrogens in *ent*copalic acid.

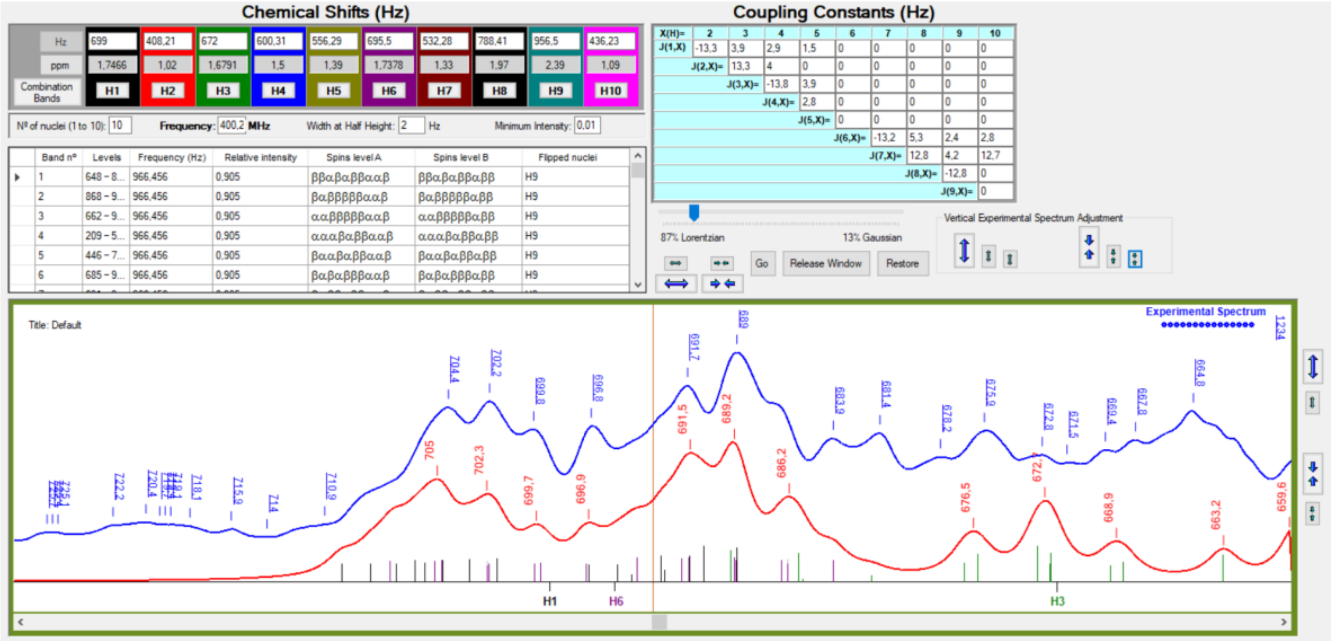

Figure T23: Comparison of calculated (red) and experimental (blue) signals for H1a and H6a by resources in NMR_MultiSim, of opening experimental spectra.

Another example of those signal types is simulation of H9 and H2a signals, which present high overlapping of H2a, H9, H11a and H2b, and intense second-order interactions. The methodology is the same as for the other signals presented above, starting with the original signal observation (figure T24) and measurement of chemical shifts and coupling constants.

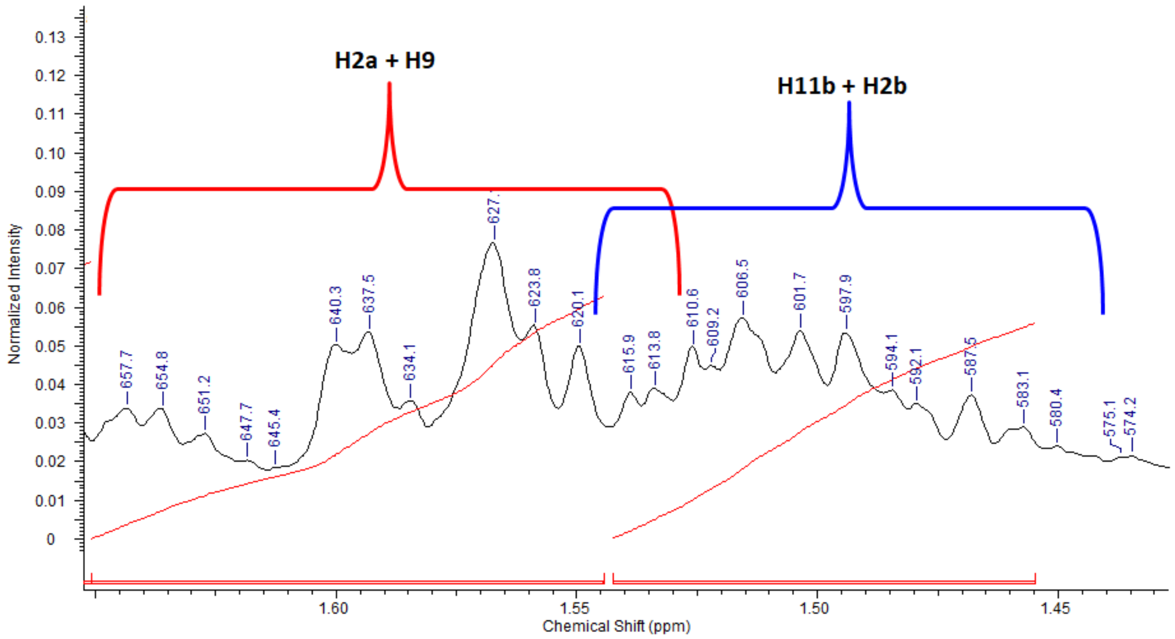

Figure T24: Experimental signals for H2a and H12a.

Filling all the required information obtained by the work with experimental spectra into NMR_MultSim forms leads to the obtention of the calculated signal. This latter can also be refined by slight adjustments, as stated before. The simulated signal, as shown on figure T25, can be now compared with the experimental or copied to generate superimposable figures for comparison (figures T26 and T27), as preferred by the users.

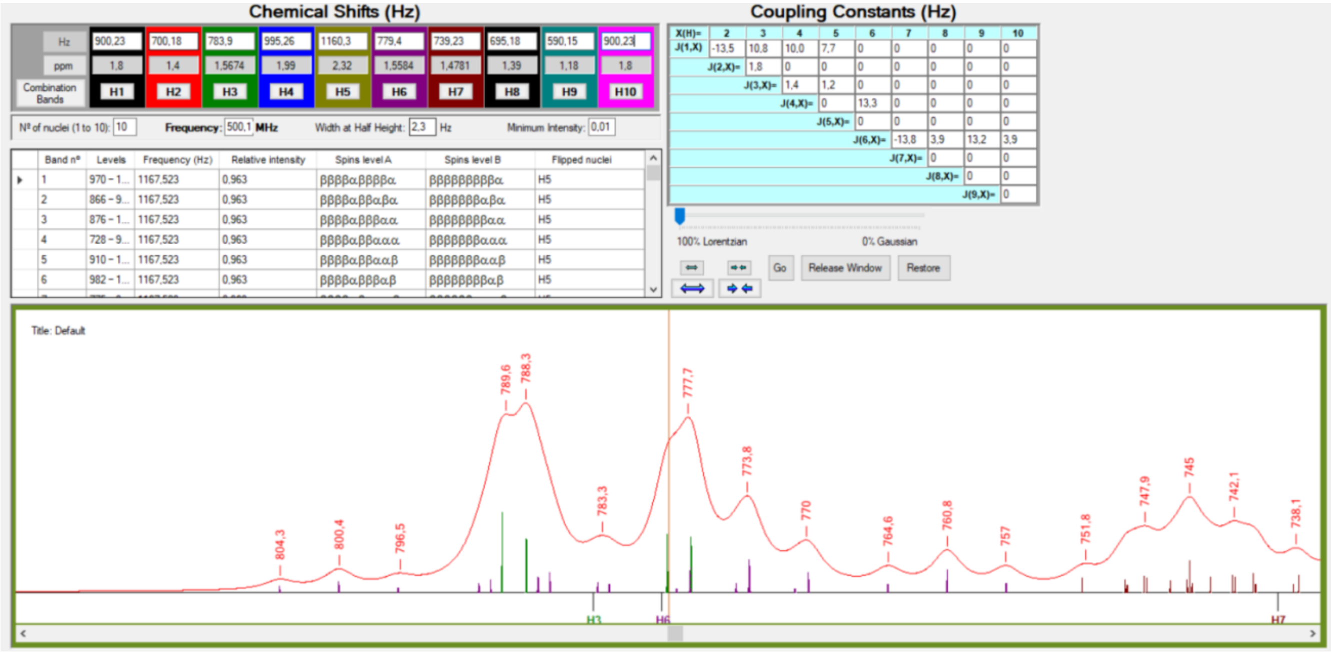

Figure T25: Screen of the program NMR_MultSim with the simulation of the H9 and H2α signals.

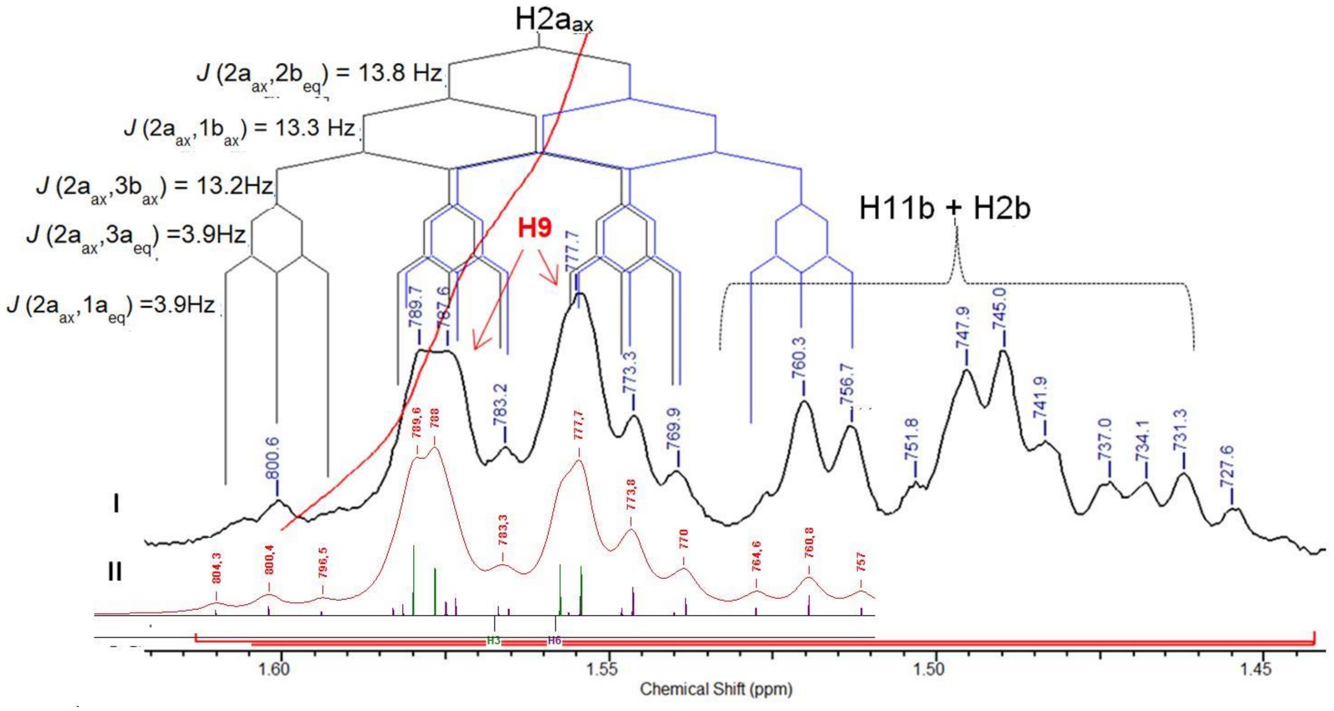

Figure T26: Experimental (**I**) and simulated (**II**) signals for H2α and H9β, obtained after several adjustments.

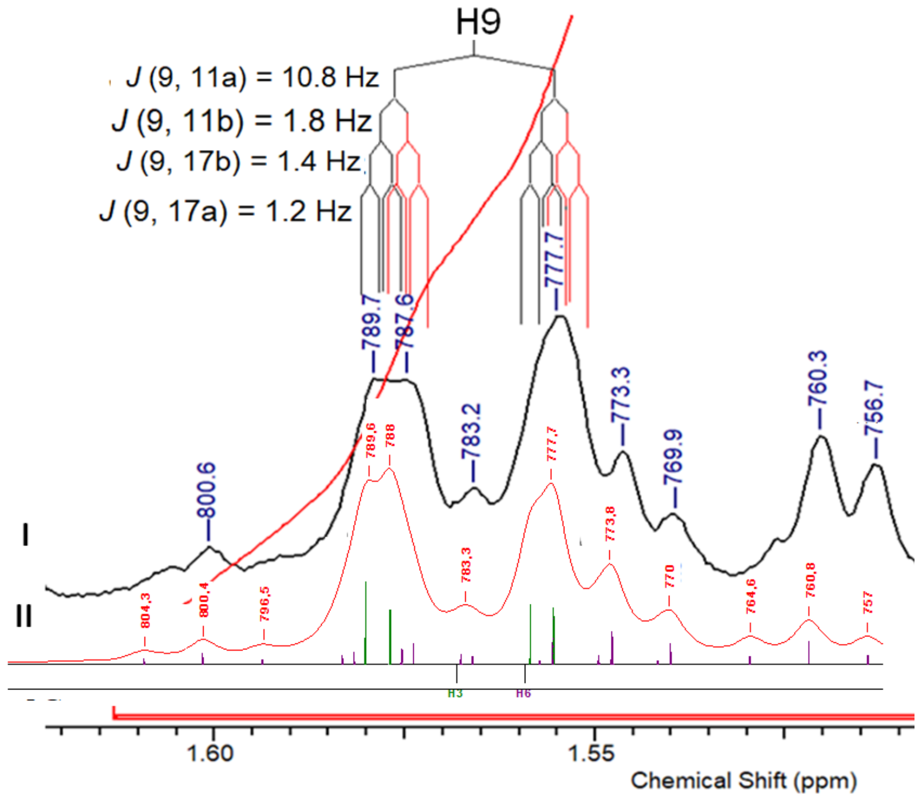

Figure T27: Experimental (**I**) and simulated (**II**) signals of the same region, highlighting H9β splitting pattern diagram.

The good similarity between the calculated and experimental signals confirms the assigned data. Such results, including their comparisons with experimental data showing good agreement in all cases, makes the value of those simulations clear. They can be considered as reliable tools to confirm and refine experimentally determined values of chemical shifts and coupling constants. Moreover, this can be performed even in cases of extreme overlapping and severe second-order modifications due to proximity of signals.

REFERENCES

1. Hipólito, U. V.; Rocha, J.T.; Palazzin, N.B.; Rodrigues, G.J.; Crestani, C.C.; Corrêa, F.M.; Bonaventura, D.; Ambrosio, S.R.; Bendhack, L.M.; Resstel, L.B.; et al. The Semi-Synthetic Kaurane Ent-16α-Methoxykauran-19-Oic Acid Induces Vascular Relaxation and Hypotension in Rats. *Eur. J. Pharmacol.* **2011**, *660*, 402–410, doi:10.1016/j.ejphar.2011.04.019.
2. Vieira, H.S.; Takahashi, J.A.; Oliveira, A.B. de; Chiari, E.; Boaventura, M.A.D. Novel Derivatives of Kaurenoic Acid: Preparation and Evaluation of Their Trypanocidal Activity. *J. Braz. Chem. Soc.* **2002**, *13*, 151–157, doi:10.1590/S0103-50532002000200004.
3. Enriquez, R.G.; Barajas, J.; Ortiz, B.; Lough, A.J.; Reynolds, W.F.; Yu, M.; Leon, I.; Gnecco, D. Comparison of Crystal and Solution Structures and 1 H and 13 C Chemical Shifts for Grandiflorenic Acid, Kaurenoic Acid, and Monoginoic Acid. *Can. J. Chem.* **1997**, *75*, 342–347, doi:10.1139/v97-039.
4. Montiel-Ruiz, R.M.; Córdova-de la Cruz, M.; González-Cortázar, M.; Zamilpa, A.; Gómez-Rivera, A.; López-Rodríguez, R.; Lobato-García, C.E.; Blé-González, E.A. Antinociceptive Effect of Hinokinin and Kaurenoic Acid Isolated from Aristolochia Odoratissima L. *Molecules* **2020**, *25*, 1454, doi:10.3390/molecules25061454.
5. Quintero-Rincón, P.; Fontal-Rivera; Contreras, R.; Fonseca; Velásquez-Gil PERIÓDICO TCHÊ QUÍMICA ARTIGO ORIGINAL SÍNTESE E CARACTERIZAÇÃO DE TRÊS CARBOXILATOS DE ORGANOTINA (IV) DO ÁCIDO ENT-CAURENÓICO: ATIVIDADE ANTIFÚNGICA CONTRA OS TRAMETES VERSICOLOR (L .: FR) PILÀT

SYNTHESIS AND CHARACTERIZATION OF THREE ORGANOTIN(IV) CARB. *Period. Tche Quim.* **2019**, *16*, 912–918.

6. Chen, Q.; Lin, H.; Wu, X.; Song, H.; Zhu, X. Preparative Separation of Six Terpenoids from Wedelia Prostrata Hemsl. by Two-Step High-Speed Counter-Current Chromatography. *J. Liq. Chromatogr. Relat. Technol.* **2018**, *41*, 408–414, doi:10.1080/10826076.2017.1412320.
7. Móricz, Á.M.; Ott, P.G.; Yüce, I.; Darcsi, A.; Béni, S.; Morlock, G.E. Effect-Directed Analysis via Hyphenated High-Performance Thin-Layer Chromatography for Bioanalytical Profiling of Sunflower Leaves. *J. Chromatogr. A* **2018**, *1533*, 213–220, doi:10.1016/j.chroma.2017.12.034.
8. Santos, J.S.; Escher, G.B.; da Silva Pereira, J.M.; Marinho, M.T.; Prado-Silva, L. do; Sant'Ana, A.S.; Dutra, L.M.; Barison, A.; Granato, D. 1H NMR Combined with Chemometrics Tools for Rapid Characterization of Edible Oils and Their Biological Properties. *Ind. Crops Prod.* **2018**, *116*, 191–200, doi:10.1016/j.indcrop.2018.02.063.
9. Guetchueng, S.T.; Nahar, L.; Ritchie, K.J.; Ismail, F.M.D.; Wansi, J.D.; Evans, A.; Sarker, S.D. Kaurane Diterpenes from the Fruits of Zanthoxylum Leprieurii (Rutaceae). *Rec. Nat. Prod.* **2017**, *11*, 304–309.
10. Elser, D.; Gilli, C.; Brecker, L.; Valant-Vetschera, K.M. Striking Diversification of Exudate Profiles in Selected Primula Lineages. *Nat. Prod. Commun.* **2016**, *11*, 1934578X1601100, doi:10.1177/1934578X1601100506.
11. Lee, K.J.; Song, K.H.; Choi, W.; Kim, Y.S. A Strategy for the Separation of Diterpenoid Isomers from the Root of Aralia Continentalis by Countercurrent Chromatography: The Distribution Ratio as a Substitute for the Partition Coefficient and a Three-Phase

Solvent System. *J. Chromatogr. A* **2015**, *1406*, 224–230, doi:10.1016/j.chroma.2015.06.038.

12. Okoye, T.C.; Akah, P.A.; Omeje, E.O.; Okoye, F.B.C.; Nworu, C.S. Anticonvulsant Effect of Kaurenoic Acid Isolated from the Root Bark of Annona Senegalensis. *Pharmacol. Biochem. Behav.* **2013**, *109*, 38–43, doi:10.1016/j.pbb.2013.05.001.
13. Padla, E.P.; Solis, L.T.; Ragasa, C.Y. Antibacterial and Antifungal Properties of Ent-Kaurenoic Acid from Smallanthus Sonchifolius. *Chin. J. Nat. Med.* **2012**, *10*, 408–414, doi:10.1016/S1875-5364(12)60080-6.
14. Nascimento, A.M. do; Oliveira, D.C.R. de Kaurene Diterpenes and Other Chemical Constituents from Mikania Stipulacea (M. Vahl) Willd. *J. Braz. Chem. Soc.* **2001**, *12*, 552–555, doi:10.1590/S0103-50532001000400019.
15. Safer, S.; Cicek, S.S.; Pieri, V.; Schwaiger, S.; Schneider, P.; Wissemann, V.; Stuppner, H. Metabolic Fingerprinting of Leontopodium Species (Asteraceae) by Means of 1H NMR and HPLC–ESI-MS. *Phytochemistry* **2011**, *72*, 1379–1389, doi:10.1016/j.phytochem.2011.04.006.
16. G. MUKHOPADHYAY, B.M.; Patra, A.; R. GHOSH, P.R.; P. LOWE Refined NMR and X-Ray Crystallographic Studies with a Diterpene from Annona Squamosa. *Fitoterapia* **1993**, *LXIV*.
17. Nett, R.S.; Dickschat, J.S.; Peters, R.J. Labeling Studies Clarify the Committed Step in Bacterial Gibberellin Biosynthesis. *Org. Lett.* **2016**, *18*, 5974–5977, doi:10.1021/acs.orglett.6b02569.
18. Ferreira, A.E.; Rocha, A.C.F.S.; Bastos, J.K.; Heleno, V.C.G. Software-Assisted Methodology for Complete Assignment of 1H and 13C NMR Data of Poorly

Functionalized Molecules: The Case of the Chemical Marker Diterpene Ent-copalic Acid. *J. Mol. Struct.* **2021**, *1228*, 129439, doi:10.1016/j.molstruc.2020.129439.

19. Pople, J.A.; Bothner-By, A.A. Nuclear Spin Coupling Between Geminal Hydrogen Atoms. *J. Chem. Phys.* **1965**, *42*, 1339–1349, doi:10.1063/1.1696119.

20. Cookson, R.C.; Crabb, T.A.; Frankel, J.J.; Hudec, J. Geminal Coupling Constants in Methylene Groups. *Tetrahedron* **1966**, *22*, 355–390, doi:10.1016/S0040-4020(01)99123-9.

21. Cahill, R.; Cookson, R.C.; Crabb, T.A. Geminal Coupling Constants in Methylene Groups—II. *Tetrahedron* **1969**, *25*, 4681–4709, doi:10.1016/S0040-4020(01)83012-X.
